# Identification of shared genetic architecture between non-alcoholic fatty liver disease and type 2 diabetes: A genome-wide analysis

**DOI:** 10.3389/fendo.2023.1050049

**Published:** 2023-03-22

**Authors:** Yajing Tan, Qian He, Kei Hang Katie Chan

**Affiliations:** ^1^ Department of Biomedical Sciences, City University of Hong Kong, Hong Kong, Hong Kong SAR, China; ^2^ Department of Electrical Engineering, City University of Hong Kong, Hong Kong, Hong Kong SAR, China; ^3^ Department of Epidemiology, Center for Global Cardiometabolic Health, Brown University, Providence, RI, United States

**Keywords:** non-alcoholic fatty liver disease, type 2 diabetes, GWAS, differential gene expression, shared genetics, mendelian randomization

## Abstract

**Background:**

The incidence of complications of non-alcoholic fatty liver disease (NAFLD) and type 2 diabetes (T2D) has been increasing.

**Method:**

In order to identify the shared genetic architecture of the two disease phenotypes of NAFLD and T2D, a European population-based GWAS summary and a cross-trait meta-analysis was used to identify significant shared genes for NAFLD and T2D. The enrichment of shared genes was then determined through the use of functional enrichment analysis to investigate the relationship between genes and phenotypes. Additionally, differential gene expression analysis was performed, significant differentially expressed genes in NAFLD and T2D were identified, genes that overlapped between those that were differentially expressed and cross-trait results were reported, and enrichment analysis was performed on the core genes that had been obtained in this way. Finally, the application of a bidirectional Mendelian randomization (MR) approach determined the causal link between NAFLD and T2D.

**Result:**

A total of 115 genes were discovered to be shared between NAFLD and T2D in the GWAS analysis. The enrichment analysis of these genes showed that some were involved in the processes such as the decomposition and metabolism of lipids, phospholipids, and glycerophospholipids. Additionally, through the use of differential gene expression analysis, 15 core genes were confirmed to be linked to both T2D and NAFLD. They were correlated with carcinoma cells and inflammation. Furthermore, the bidirectional MR identified a positive causal relationship between NAFLD and T2D.

**Conclusion:**

Our study determined the genetic structure shared between NAFLD and T2D, offering a new reference for the genetic pathogenesis and mechanism of NAFLD and T2D comorbidities.

## 1 Introduction

Non-alcoholic fatty liver disease (NAFLD) is a clinicopathological syndrome that is characterized by hepatic parenchymal steatosis and fat storage in the absence of a history of binge drinking. In simple terms, NAFLD is usually benign, but if linked with an unhealthy lifestyle, obesity, and other metabolic syndromes, it may develop from simple fat accumulation to non-alcoholic steatohepatitis (NASH), liver fibrosis and cirrhosis, and, in rare cases, liver cancer (1). It is a complex disease that results from the interaction of environmental and genetic factors, so it has multiple pathogenic factors such as insulin resistance (IR), lipid metabolism disorders, oxidative stress, and cytokine effects (2). In addition to being considered a manifestation of IR in the liver, NAFLD often coexists with metabolic syndromes such as obesity, type 2 diabetes (T2D), and hyperlipidemia (3). Among them, T2D is a typical endocrine and metabolic disease that is affected by multiple pathogenic factors, and its incidence is increasing rapidly worldwide. T2D, the most prevalent type of diabetes, is characterized by hyperglycemia, IR, and lipid metabolism disorder as the pathological basis (4). In recent years, it has been found that NAFLD and T2D show similar pathological characteristics and often coexist as common diseases that seriously endanger public health. On one hand, T2D can lead to dysfunction of glycolipid metabolism in the body through the development of factors such as IR, chronic inflammation and oxidative stress, which results in NAFLD and further liver damage and worsens the prognosis of NAFLD (5)? On the other hand, through fat deposition, inflammation, endoplasmic reticulum stress, and oxidative stress, NAFLD can also exacerbate hepatic IR and promote metabolic abnormalities including hyperglycemia, creating the ideal environment for the development of T2D (6).

Related studies have shown that NAFLD and T2D interact with each other, and that there is a complex two-way relationship between the two that can accelerate deterioration. Targher et al. (7) found that NAFLD was ubiquitous in patients with T2D (7). Similarly, Jarvis et al. (8), in a meta-analysis of population-based cohort studies, found that the occurrence of T2D was associated with a more than two-fold increase in the risk of severe liver disease events among those at risk of or diagnosed with NAFLD (8). This finding was the same as that found by Mantovani et al. (9) in a study of the impact of NAFLD on the risk of development of T2D (9). Also, Pinero et al. (10) reported that the global incidence of NASH had reached 3% to 5%. NASH occurs in 20% to 30% of patients with T2D and obesity, and NAFLD occurs in 69% to 87% of those patients (10). Hence, the incidence of NAFLD combined with T2D is higher than that of NAFLD alone or T2D alone (5). Thus, the identification of the shared genetic architecture of NAFLD and T2D has important implications for the prevention and treatment of these diseases.

In recent years, the development of next-generation sequencing and high-throughput genotyping arrays has led to the GWAS and exome-wide association studies (EWAS), which are methods for the identification of genetic factors for many complex diseases (11). The use of GWAS, which is larger-scale than EWAS, has led to the identification of many polymorphisms and genetic variants that are associated with NAFLD and T2D and the investigation of new therapeutic targets. In addition, differentially expressed genes (DEGs) are key to learn about gene activity. They have now become one of the most important tools for the discovery of biomarkers (12). This method can be used to find genes that exhibit notable variations in expression, to analyze statistically the findings to pinpoint particular genes that are associated with those conditions, and then to analyze the biological importance of those particular genes. More importantly, DEGs can complement the knowledge of important target tissues and cell types that the GWAS approach lacks in disease pathogenesis, thus realizing the transformation of relevant gene loci into mechanisms. Hence it can be seen that the integration of GWAS summary statistics and gene expression data can identify disease-related tissues and cell types without bias, increase the credibility of the analysis results, and provide a sufficient basis to explain the pathogenesis.

Current genetic studies that target NAFLD and T2D require the discovery of more significant genetic association signals to support and translate the research through various novel analytical methods into biological and potentially therapeutic knowledge. Therefore, this study first conducted a comprehensive genetic analysis through the use of GWAS to identify susceptibility genes for NAFLD combined with T2D. The core shared genes were further screened as this information was combined with the results of DEG analysis. Subsequently, functional annotation analysis was performed to identify the underlying biological pathways of these core, shared genes. At the same time, a two-sample MR analysis was conducted to explore the causal relationship between NAFLD and T2D. The above analysis provided a robust theoretical basis for the study of NAFLD and T2D complications and new ideas and opportunities for the further development of prevention and treatment strategies.

## 2 Methods

### 2.1 Data summary

To identify genetic variants in NAFLD combined with T2D, the GWAS summary statistics for this study were obtained from the US National Human Genome Research Institute GWAS catalog (https://www.ebi.ac.uk/gwas/), including NAFLD (1,483 cases and 17,781 controls) and T2D (4,040 cases and 113,735 controls). (13, 14). For the DEG analysis, the organism was *Homo sapiens*, and the experiment type was expression profiling by array, which set the screening conditions of the dataset. The related gene expression datasets GSE48452, GSE25724, GSE17470, and GSE20966 were downloaded from the Gene Expression Omnibus database (https://www.ncbi.nlm.nih.gov/geo/), which included human liver biopsy (18 NASH, 14 NAF, 27 obese, 14 controls), human islets, and NASH liver biopsy (6 case and 4 controls) and beta-cells from pancreatic tissue (n=10) (15–18). All data sources can be found in **Table 1**.

**Table 1:** Data source information summary.

Phenotype	Data Source	Population	Cases’ size	Controls’ size	Total’ size	Number (SNPs)	PubMed ID	Download Link
NAFLD	GWAS Catalog	European	1,483	17,781	19,264	6,797,908	32298765	https://www.ebi.ac.uk/gwas/studies/GCST90011885
T2D	GWAS Catalog	European	4,040	113,735	117,775	8,404,432	26961502	https://www.ebi.ac.uk/gwas/studies/GCST006801
NAFLD (Discovery set)	GEO database (GSE48452, GPL11532)	Germany	32	41	73	–	23931760	https://www.ncbi.nlm.nih.gov/geo/query/acc.cgi
NAFLD (Validation set)	GEO database (GSE17470, GPL2895)	USA	7	4	11	–	20221393	https://www.ncbi.nlm.nih.gov/geo/query/acc.cgi
T2D (Discovery set)	GEO database (GSE25724, GPL96)	Italy	6	7	13	–	21127054	https://www.ncbi.nlm.nih.gov/geo/query/acc.cgi
T2D (Validation set)	GEO database (GSE20966, GPL1352)	USA	10	10	20	–	20644627	https://www.ncbi.nlm.nih.gov/geo/query/acc.cgi

### 2.2 Study-level quality control

The “GWASInspector” R package was used to conduct harmonized quality control (QC) on the GWAS statistics of NAFLD and T2D phenotypes to ensure that false positive signals were eliminated and that low-quality data did not obscure actual signals (19, 20). NAFLD and T2D GWAS summary data were performed separately in the QC. The reference dataset was the 1000 genomes project reference panel (21), the specific genome build version was GRCh37, and we included the relevant information from the European population. The QC involved: the deletion of variants that contained missing fundamental values or were duplicated; the deletion of monomorphic variants; the checking of the consistency of allele frequencies with reference datasets; the alignment of alleles with reference datasets, and the comparison of those alleles to ensure that the resulting allele frequencies were correct; the removal of unverifiable mismatches and multi-allelic variants, and; the setting of a threshold plot_cut-off*_p* = 0.01 to exclude low-significance SNPs.

### 2.3 Cross-trait meta-analysis

Cross-trait meta-analysis was performed using the CPASSOC software package. CPASSOC is a method for studying cross-phenotypic (CP) associations by using summary statistics from GWAS of multiple phenotypes. It combines effect estimates and standard errors of GWAS summary statistics to test the hypothesis of an association between SNPs and traits (22). Cross-phenotype associations increase statistical power when the traits analyzed share common variants or common genetic pathways, which are often associated with pleiotropy (23). CPASSOC includes two tests, SHom and SHet. In this study, R v.4.1.3 was used to perform the SHet test considering the effect of trait heterogeneity, which can increase the power when the genetic effect size of different traits is different (24). At this time, the gamma distribution parameters are estimated by setting *N = 1E4* and calling the EstimateGamma function. Due to the many hypothesis tests that may be applied in GWAS studies, the threshold is strictly controlled to minimize the number of false positives reported. Currently, the most significant threshold is generally recognized as *p*<5×l0^-8^, and this threshold also applies to CPASSOC (24, 25). Then, hg19 was used as the reference genome, and the refGene database was used to annotate the SNPs that reached the threshold of significance level using the ANNOVAR software (http://www.openbioinformatics.org/annovar/). Finally, the shared genes of NAFLD combined with T2D were obtained.

### 2.4 Enrichment analysis

In this study, SNPs that showed significant variation in meta-analysis and the genes from which they came were used for functional enrichment analysis to explore the potential biological function of shared susceptibility genes between NAFLD and T2D. The online tool Metaspace (https://metascape.org/gp/index.html#/main/step1) was used to analyze comprehensively these susceptibility genes. Metaspace integrates more than 40 gene function annotation databases such as Gene Ontology (GO) and DisGeNET, providing multiple functional and diversified visualization methods such as gene enrichment analysis and protein interaction network analysis, which can be used for easy exploration and analysis of gene function (26). The GO enrichment analysis of candidate genes was focused on the use of the “clusterProfiler” package of R v.4.1.3 (https://www.r-project.org/). In addition, the online platform TissueEnrich (https://tissueenrich.gdcb.iastate.edu/) was used as a calculating input-gene centralized organization-specific enrichment tool to complete the tissue-specific expression analysis (27).

### 2.5 Differential gene expression analysis and enrichment

To confirm which of the chosen genes were the core shared genes in NAFLD and T2D, four GEO datasets were selected for DEG analysis. The specific dataset information is shown in **Table 1**. Of the four, GSE48452 and GSE25724 were used as discovery sets, while GSE17470 and GSE20966 were used as validation sets to verify the validity and disease association of the identified genes. In addition, each dataset was divided into two groups of samples, with NAFLD patients or T2D patients as the experimental group and healthy people as the control group. GEO data were processed through the application of the online analysis tool GEO2R (https://www.ncbi.nlm.nih.gov/geo/geo2r/) to identify DEGs. The visualization of overlapping genes in GEO datasets is realized through the online platform jvenn (http://www.bioinformatics.com.cn/static/others/jvenn/example.html) (28). After the discovery and validation sets were merged, the final DEGs were screened through use of adjust.P.Value, which is applied to adjust the *p*-value for multiple tests to control the false discovery rate (FDR). The FDR is calculated as expected rate x (false positive/(false positive + true positive)) (29). The value of *adj.p* was set at <*0.05* to screen out the DEGs of NAFLD and T2D. Then, the genes that overlapped with those found through the GWAS were identified as the shared core genes of NAFLD and T2D. Differential gene expression analysis is to identify shared genes through differential expression analysis between two phenotypes and to find overlapping genes with cross-trait analysis based on the GWAS summary statistics. Using multiple analytical methods to explore the reproducibility of our results between the two phenotypes can make the findings more reliable and robust. Next, the enrichment analysis described in section 2.4 was carried out for the genes that were found to overlap in the GWAS and DEG analyses.

### 2.6 Mendelian randomization analysis

The QC-processed GWAS data were used in the MR analysis. The potential causal effect between T2D and NAFLD was explored through the use of a bidirectional MR analysis, in which the two traits were evaluated alternately as exposure and outcome, and independent SNPs that were closely related to exposure and outcome traits were used as instrumental variables. Among them, the screening of exposure was essential. The parameters *p=*5×10^-8^ specified the *p*-value of the SNP in the exposure; that is, only SNPs with *p*-values of <5×10^-8^ were extracted (30). The NbDistribution simulation calculation was set to 1000 and the *p*-value threshold for judging whether the SNP was an outlier was set to 0.05 before the MR analysis was performed. Then, the calculation of MR pleiotropy residual sum and outlier (MR-PRESSO) was performed to identify the existence of the outliers (31). Once outliers were located, they were eliminated, and subsequent MR analysis was performed. MR and sensitivity analyses were performed through the use of the inverse variance weighted (IVW) method (32) with multiplicative random effects, supplemented by MR Egger (33, 34), weighted median (33), simple mode, and weighted mode methods (35). It is important to note that horizontal pleiotropy is a potential confounding factor in MR analysis; i.e., instrumental variable SNPs influence the outcome through a non-causal pathway, which may affect the measurement of the relationship between traits. To examine the impact of pleiotropy on the results of the MR analysis, MR-PRESSO was also used to test for horizontal pleiotropy for multiple instrumental variables. In addition, heterogeneity statistics and leave-one-out analyses were included in the MR analysis. Heterogeneity statistics mainly test the differences between individual SNPs, and leave-one-out analysis mainly tests the stability of MR results. The “TwoSampleMR” and “MRPRESSO” packages were used for MR analysis in R v.4.1.3.

## 3 Results

### 3.1 Study-level QC

QC was performed through the use of “GWASInspector”. 100% of NAFLD GWAS summary data (6,797,908 SNPs) and 99.7% of T2D GWAS summary data (8,380,746 SNPs) passed the QC procedure (**Table 2**). SNPs that passed the QC were included in the subsequent cross-trait meta-analysis and MR analysis.

**Table 2:** The number of SNPs after QC processing.

	NAFLD	T2D
Input variant count	6,797,908	8,404,432
Missing crucial variable	0	2
Duplicated variants	0	12,367
Monomorphic variants	0	0
Output variant count	6,797,908	8,380,746

### 3.2 Cross-trait meta-analysis

In total, CPASSOC identified 241 SNPs that were significantly associated (*p<5×10^-8^*) between NAFLD and T2D (**Supplementary Table 1**). In the results of the cross-trait meta-analysis, the SNP with the most significant p-value is rs73233361 (*p=6.78×10^-11^*), which is located on chromosome 12. Most of the remaining SNPs are located on chromosome 6, chromosome 2, and chromosome 3. 115 genes were obtained by ANNOVAR annotation (**Supplementary Table 1**).

### 3.3 Enrichment analysis

**Figure 1:**
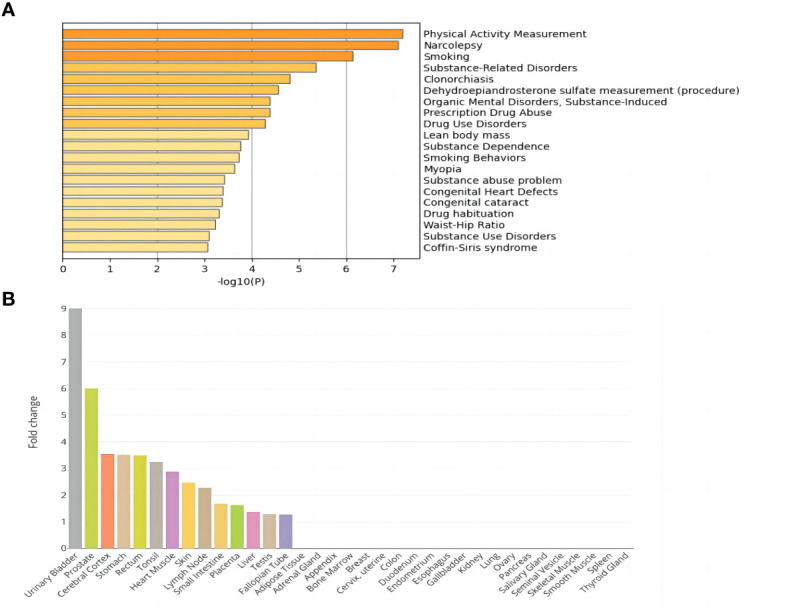
Enrichment analysis of shared genes in NAFLD and T2D. **(A)** DisGeNET enrichment analysis results. **(B)** Tissue enrichment results.

The DisGeNET enrichment analysis revealed that 115 shared genes were enriched in physical activity measurement, substance-related disorders, lean body mass, smoking behaviors, substance abuse problem, etc. (**Figure 1A**). The relevant results that were identified based on DisGeNET enrichment analysis are listed in **Supplementary Table 2**. GO enrichment analysis (**Supplementary Table 3**; **Supplementary Figure 1**) showed that the shared genes of NAFLD and T2D were enriched in the biological processes of processes of glycerophospholipids, phospholipids, lipid decomposition, glycerolipid metabolism, and they also participated in the enrichment in the molecular function of activity of various enzymes. Among them, the most were associated with sensory system development, a total of 7 genes (*FASLG, ISL1, TULP1, TBC1D32, ATP8A2, MAX*, and *ADAMTS18*). In addition, tissue enrichment analysis showed that NAFLD and T2D shared genes were mainly enriched in 14 tissues: the urinary bladder, prostate, cerebral cortex, stomach, rectum, tonsil, heart muscle, skin, lymph node, small intestine, placenta, liver, testis, and fallopian tube (**Figure 1B**). Among these tissues, the liver is closely related to the pathogenesis of NAFLD and T2D.

### 3.4 Differential gene expression analysis and enrichment

The DEG analysis results for each dataset are shown in **Supplementary Figures 2–5**. The discovery sets GSE48452 and GSE25724 contained 11,795 shared genes for NAFLD and T2D (**Supplementary Figures 6A, B**), whereas the validation sets GSE17470 and GSE20966 contained 15,557 shared genes for NAFLD and T2D (**Supplementary Figures 6C, D**). A combination of all discovery and validation sets yielded 9711 DEGs (**Supplementary Figure 6E**). Subsequently, 5545 DEGs shared by NAFLD and T2D were screened by adj.P (**Supplementary Table 4**). Consideration of these genes with the candidate genes that had been obtained through the GWAS produced fifteen core genes that were shared by NAFLD and T2D, namely *DNAJB9, VPS53, SCGN, CMAS, RGS6, FASLG, ABHD10, ATRN, PLA2G2F, ITIH2, ROBO1, SGCG, SH3GL2, CNR1*, and *FOXN3* (**Table 3**).

**Table 3:** Core genes after GWAS analysis and differential gene expression analysis combined.

	Gene	Cytogenetic region	Description	Remark
1	*VPS53*	17p13.3	VPS53 subunit of GARP complex	Not novel gene
2	*SCGN*	6p22.2	secretagogin, EF-hand calcium binding protein	Not novel gene
3	*RGS6*	14q24.2	regulator of G protein signaling 6	Not novel gene
4	*SGCG*	13q12.12	sarcoglycan gamma	Not novel gene
5	*FOXN3*	14q32.11	forkhead box N3	Not novel gene
6	*DNAJB9*	7q31.1	DnaJ heat shock protein family (Hsp40) member B9	Novel gene
7	*CMAS*	12p12.1	cytidine monophosphate N-acetylneuraminic acid synthetase	Novel gene
8	*FASLG*	1q24.3	Fas ligand	Novel gene
9	*ABHD10*	3q13.2	abhydrolase domain containing 10, depalmitoylase	Novel gene
10	*ATRN*	20p13	attractin	Novel gene
11	*PLA2G2F*	1p36.12	phospholipase A2 group IIF	Novel gene
12	*ITIH2*	10p14	inter-alpha-trypsin inhibitor heavy chain 2	Novel gene
13	*ROBO1*	3p12.3	roundabout guidance receptor 1	Novel gene
14	*SH3GL2*	9p22.2	SH3 domain containing GRB2 like 2, endophilin A1	Novel gene
15	*CNR1*	6q15	cannabinoid receptor 1	Novel gene

**Figure 2:**
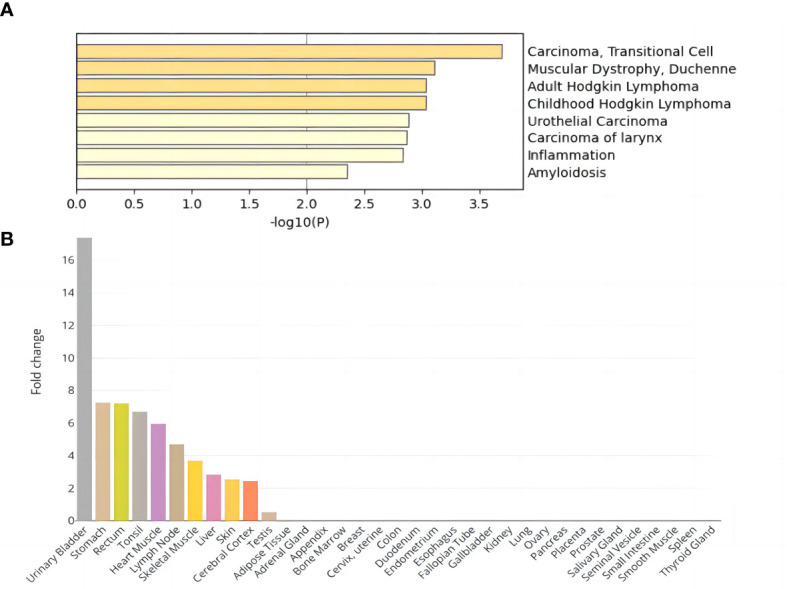
Enrichment analysis results of DEGs shared by NAFLD and T2D. **(A)** DisGeNET enrichment analysis results. **(B)** Tissue enrichment results.

DEG analysis of the above core genes (**Supplementary Table 5**) showed that seven genes were upregulated (*logFoldChange>0*) and eight genes were downregulated (*logFoldChange<0*) in disease. These fifteen core genes were subjected to enrichment analysis, and DisGeNET enrichment analysis revealed that they were enriched in carcinoma cells and inflammation (**Figure 2A**). Relevant findings from the DisGeNET enrichment analysis are provided in **Supplementary Table 6**.

GO enrichment analysis showed significant enrichment of several biological processes including regulation of endopeptidase and peptidase activity, lipid catabolic process, fatty acid metabolic process, response to lipopolysaccharide, and positive regulation of proteolysis; cellular components of the distal axon, endoplasmic reticulum lumen, and glutamatergic synapse; and molecular functions such as carboxylic ester hydrolase activity (**Supplementary Table 7**; **Supplementary Figure 7**). In addition, tissue enrichment analysis showed that NAFLD and T2D core shared genes were enriched in the urinary bladder, stomach, rectum, tonsil, heart muscle, lymph node, skeletal muscle, liver, skin, cerebral cortex, and testis (**Figure 2B**). The above enrichment results supported the earlier finding that the fifteen core shared genes, *DNAJB9, VPS53, SCGN, CMAS, RGS6, FASLG, ABHD10, ATRN, PLA2G2F, ITIH2, ROBO1, SGCG, SH3GL2, CNR1*, and *FOXN3*, were closely related to NAFLD and T2D.

### 3.5 Mendelian randomization analysis

**Figure 3:**
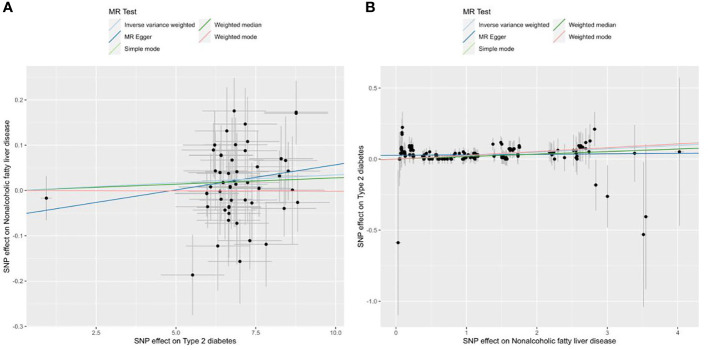
Scatter plots of the MR analysis. The light blue line shows the result of the IVW method, which has the most significant impact. The dark blue line shows the result of the MR Egger method; the light green is the result of the simple model method; the dark green is the result of the weighted median mode; and the red line represents the result of the weighted mode method. **(A)** Scatter plot of T2D as exposure and NAFLD as outcome. **(B)** Scatter plot of NAFLD as exposure and T2D as outcome.

No outliers were detected after processing with the “MRPRESSO” R package. The results of MR analysis in T2D and NAFLD are listed in **Table 4**. Among the results, regardless of whether NAFLD or T2D was used as exposure or outcome, the *p*-value obtained by the IVW method was less than 0.05, indicating a causal relationship between T2D and NAFLD; the related *beta*-value was more than zero, which indicated that the causal relationship between T2D and NAFLD was positive; this meant that increasing exposure (T2D) increased the risk of the outcome (NAFLD). From the scatter plot of the MR results (**Figure 3**), it can be seen that the IVW method yielded the most significant results among the five methods that were used for MR analysis. The plot also demonstrates the positive relationship between T2D and NAFLD, as did the forest plot (**Supplementary Figure 8**).

**Table 4:** Results of two-sample MR analysis of NAFLD and T2D.

Outcome	Exposure	Method	nsnp	b(exposure/outcome)	se	pval	OR (95%CI)
NAFLD	T2D	MR Egger	62	0.01109	0.00652	0.09406	1.01115 (0.99832-1.02415)
		Weighted median	62	0.00278	0.00184	0.13017	1.00278 (0.99918-1.00639)
		Inverse variance weighted	62	0.00343	0.00129	0.00801	1.00344 (1.00090-1.00599)
		Simple mode	62	-0.00016	0.00461	0.97296	0.99984 (0.99084-1.00892)
		Weighted mode	62	-0.00016	0.00359	0.96526	0.99984 (0.99283-1.00690)
T2D	NAFLD	MR Egger	217	0.00344	0.00573	0.54896	1.00344 (0.99224 -1.01477)
		Weighted median	217	0.01771	0.00389	5.36E-06	1.01787 (1.01013-1.02566)
		Inverse variance weighted	217	0.02414	0.00302	1.24E-15	1.02443 (1.01839-1.03051)
		Simple mode	217	0.02673	0.01887	0.15799	1.02709 (0.98980-1.06578)
		Weighted mode	217	0.02673	0.01909	0.16297	1.02709 (0.98936 -1.06625)

The statistical results of heterogeneity show that there was no heterogeneity between the instrumental variable SNPs (Q_pval was >0.05), which can be confirmed from the funnel plot (**Supplementary Figure 9**). The results of the pleiotropy test show that there was no statistical difference (*p>*0.05), which indicates that there was no horizontal pleiotropic effect. Through leave-one-out analysis (**Supplementary Table 8**; **Supplementary Figure 10**), it can be seen that no matter which SNP was removed, it would not have a fundamental impact on the results. So the MR results are robust.

## 4 Discussion

This study used GWAS summary data for 6,797,908 NAFLD and 8,404,432 T2D from European populations to determine the shared genetic architecture of these two phenotypes. A cross-trait meta-analysis identified 115 shared genes, and subsequent DEG analysis identified fifteen core shared genes: *DNAJB9, VPS53, SCGN, CMAS, RGS6, FASLG, ABHD10, ATRN, PLA2G2F, ITIH2, ROBO1, SGCG, SH3GL2, CNR1*, and *FOXN3*.

The liver is a vital organ that regulates glucose and lipid metabolism, and hepatic fat deposition is a critical factor in the pathogenesis of NAFLD and T2D (36). The twin-cycle hypothesis based on T2D explains that a gradual increase in the level of fat in the liver can lead to IR, which weakens the ability of insulin to suppress hepatic glucose production. This leads to an aggravation of hepatic gluconeogenesis and rises in blood sugar levels (37). The excess glucose is used to synthesize triglycerides, which results in increased levels of liver fat and reduced capacity to use glucose. These processes create a vicious circle between the liver and pancreas (38). At the same time, hepatic triglyceride synthesis is increased in NAFLD patients. When the level of free fatty acids (FFAs) produced by lipoprotein lipase exceeds the lipid storage capacity of adipose tissue, β-cells will take up many fatty acids and store them as triglycerides. This damages the β cells and causes IR (39). This may eventually promote the progression of liver damage to HCC.

Relevant studies to date have shown that the mechanism of action of the mechanisms mentioned above has become a tool for the conduct of research in clinical practice. Previous studies have suggested some potential links between these mechanisms and the identified, core shared genes. Forkhead box N3 (*FOXN3*), an important member of the FOX transcription factor family, is an important tumor suppressor gene that plays a crucial role in several cancers such as liver cancer, lung cancer, and colon cancer (40). The *FOXN3* gene locus is associated with fasting blood glucose levels. Hepatic *FOXN3* increases fasting blood glucose by inhibiting hepatic glucose utilization while also regulating the expression of amino acid transporters and catabolic enzymes (41, 42). Studies have shown that *FOXN3* suppresses the mRNA and protein expression of *E2F5* by inhibiting the promoter activity of potential oncogene *E2F5*, thereby inhibiting the proliferation of HCC cells *in vitro* and *in vivo* (43). Another tumor suppressor, regulator of G protein signaling 6 (*RGS6*), is upregulated in the liver of NAFLD patients, forms a complex with ATM in the liver, promotes ATM phosphorylation, and drives hepatic steatosis (44, 45). A study confirmed that hepatic *RGS6* increases oxidative stress and inflammation, which drive lipid deposition, fibrosis, and nonalcoholic fatty liver disease (46). In contrast, *RGS6* deficiency effectively ameliorated fat deposition, attenuated alcohol-dependent liver injury, and enhanced liver regeneration (47).

Other genes that may play a role in NAFLD and T2D include *SGCG*, a single-pass transmembrane glycoprotein implicated in the pathogenesis of obesity and T2D in humans (48). It has beneficial effects on glucose homeostasis, and elevated levels in diabetic patients may be compensatory for IR (49). Furthermore, *SCGN* is highly enriched in pancreatic β-cells and has pronounced effects on lipolysis and lipogenesis (50). It also regulates insulin expression and secretion, which is downregulated in type 2 diabetes (51, 52). Studies have shown that the *SCGN*-insulin interaction can stabilize insulin, enhance the hypoglycemic activity of insulin *in vivo*, and reduce hepatic steatosis and cholesterol metabolism disorders (51). In addition, the homologous gene *HCCS1* of *VPS53* also has a strong anti-tumor effect on liver cancer cells (53, 54).

Through a combination of the results of this study with the known mechanisms of action of NAFLD and T2D and related research findings, it can be shown that essential pathways affecting NAFLD and T2D include catabolism of lipids such as fatty acids, glycerides, and phospholipids. These biological processes affect lipid levels in tissues and hence affect hepatic fat accumulation and IR. Further research on this aspect of our findings should be considered.

In conclusion, the determination of triglyceride, FFA, and cholesterol levels can assist in the clinical observation of the dynamic changes in liver fat levels and IR and is of great significance in the prediction of comorbidities. At the same time, through the continuous deepening of genetic research, the development of targeted drugs to regulate the level of liver fat and the regulation of liver fat content is expected to become a key and effective treatment method for comorbidities. In addition, once the relevant mechanism of action is identified, specific gene therapy for NAFLD and T2D is expected to be realized.

One limitation of this study was that the shared genes were all screened from the results of GWAS studies in European populations, so other populations were not considered. Few replicated validation studies of the susceptibility loci associated with NAFLD and T2D have been conducted in other populations. Genetic and environmental factors influence the genetic backgrounds of populations and result in variations in allele frequencies, which affect illness incidence rates and the findings of GWAS analyses of susceptibility genes. Therefore it is uncertain whether the susceptibility genes identified in this study exist in other populations. However, the results of this study can provide a reference for research on NAFLD combined with T2D in other populations. GWAS research involves not only different populations but also different genders and different ages, and this richness of the data should be exploited for further exploration.

It is essential to note that this study cannot avoid the shortcomings of GWAS itself, such as the fact that the study is focused on the loci that achieve the significance threshold for genome-wide association, even though these loci account only partially for the complicated heredity of the disease (55). GWAS studies often overlook signals of mild or moderate association and ignore the effects of other variants such as gene deletions, copy number variations, etc. These neglected factors may involve underlying biological mechanisms that ultimately lead to the occurrence of disease. While NAFLD and T2D are complex diseases in which genetic and environmental factors interact, the pathogenesis is often caused by mutations or abnormalities of multiple genes, and each gene may play a part in a specific pathway but its role cannot explain the whole mechanism. Therefore, the study design can be effectively improved to make up for these issues with GWAS and the complexity of the disease. For example, the candidate gene method is used to find low-frequency variants, or the data from multiple studies can be combined in a meta-analysis to increase the sample size, and rare variants with substantial genetic effects can be found in this way (56, 57).

The strength of this study was that it involved the first comprehensive use of GWAS and DEG analysis to identify shared genes for NAFLD and T2D. During gene screening, strict thresholds were used to ensure the accuracy of the results, and significant shared genes were discovered efficiently. The study reconfirmed the association of the unveiled core genes, *VPS53, SCGN, RGS6, SGCG*, and *FOXN3*, with NAFLD and T2D, which had been reported in previous studies. The core genes *DNAJB9, CMAS, FASLG, ABHD10, ATRN, PLA2G2F, ITIH2, ROBO1, SH3GL2*, and *CNR1* were found to be related to NAFLD and T2D for the first time, and this provides a new research target for the precise treatment of NAFLD and T2D comorbidities.

## 5 Conclusion

In summary, this study found a causal relationship between NAFLD and T2D, which will be beneficial for the elucidation of the pathogenesis of NAFLD and T2D comorbidities. Fifteen core genes, *DNAJB9, VPS53, SCGN, CMAS, RGS6, FASLG, ABHD10, ATRN, PLA2G2F, ITIH2, ROBO1, SGCG, SH3GL2, CNR1, and FOXN3*, were identified as shared between NAFLD and T2D. This finding provided new ideas for the genetic study of NAFLD combined with T2D. Further gene expression verification and functional mechanism research should be carried out on these candidate genes in the future to explore the specific biological mechanisms of NAFLD and T2D comorbidities and to provide new drug-targeting sites for the prevention and treatment of comorbidities.

## Data availability statement

The datasets presented in this study can be found in online repositories. The names of the repository/repositories and accession number(s) can be found in the article/**Supplementary Material**.

## Author contributions

YT participated in data analysis, drafting, writing, and revising the paper. QH participated in the revision of the manuscript and carried out a strict review of the manuscript. KC conceived, designed, coordinated the study, and revised the manuscript. All authors contributed to the article and approved the submitted version.
