# Supplementary material for: Identification of shared genetic architecture between non-alcoholic fatty liver disease and type 2 diabetes: A genome-wide analysis
Source: Front Endocrinol (Lausanne). 2023 Mar 22;14:1050049. doi: 10.3389/fendo.2023.1050049 (PMC10073682; doi:10.3389/fendo.2023.1050049)
Supplement: Supplementary file 1 [file Table_1.docx]

Supplementary Material

**Supplementary Table 1** Cross-trait meta-analysis results between NAFLD and T2D (P<5×10^-8^)

| SNP | CHR | Genome position | allele_1 | allele_2 | p.shet | Gene |
| --- | --- | --- | --- | --- | --- | --- |
| rs77237404 | 3 | 3q13.2 | G | T | 4.44E-08 | ABHD10 |
| rs77133505 | 16 | 16q23.1 | C | A | 4.53E-08 | ADAMTS18 |
| rs208348 | 7 | 7p22.3 | C | G | 3.18E-08 | AMZ1;GNA12 |
| rs11978089 | 7 | 7q22.1 | C | T | 9.54E-09 | ARPC1B |
| rs2003499 | 7 | 7q22.1 | C | T | 1.75E-08 | ATP5MF-PTCD1;PTCD1 |
| rs9511911 | 13 | 13q12.13 | C | T | 4.88E-08 | ATP8A2 |
| rs671614 | 6 | 6q14.1 | A | G | 1.82E-08 | BCKDHB |
| rs640568 | 6 | 6q14.1 | T | C | 1.82E-08 |
| rs688442 | 6 | 6q14.1 | T | G | 1.81E-08 |
| rs649148 | 6 | 6q14.1 | A | G | 1.82E-08 |
| rs590686 | 6 | 6q14.1 | T | C | 1.83E-08 |
| rs587999 | 6 | 6q14.1 | T | C | 1.84E-08 |
| rs585506 | 6 | 6q14.1 | A | G | 1.05E-08 |
| rs651065 | 6 | 6q14.1 | A | T | 1.85E-08 |
| rs670119 | 6 | 6q14.1 | T | C | 1.83E-08 |
| rs652576 | 6 | 6q14.1 | A | G | 4.19E-09 |
| rs687702 | 6 | 6q14.1 | A | C | 1.72E-08 |
| rs685902 | 6 | 6q14.1 | C | G | 4.19E-09 |
| rs642943 | 6 | 6q14.1 | T | C | 1.85E-08 |
| rs641229 | 6 | 6q14.1 | A | T | 1.85E-08 |
| rs638921 | 6 | 6q14.1 | C | G | 8.22E-09 |
| rs677134 | 6 | 6q14.1 | T | G | 1.82E-08 |
| rs4706829 | 6 | 6q14.1 | A | G | 1.82E-08 |
| rs4706830 | 6 | 6q14.1 | A | G | 1.82E-08 |
| rs723308 | 6 | 6q14.1 | A | T | 1.82E-08 |
| rs6904257 | 6 | 6q14.1 | C | G | 1.80E-08 |
| rs806839 | 6 | 6q14.1 | A | G | 1.80E-08 |
| rs932463 | 6 | 6q14.1 | T | C | 1.81E-08 |
| rs78096436 | 20 | 20p13 | C | T | 1.71E-09 | C20orf194;ATRN |
| rs2591025 | 8 | 8q13.2 | G | A | 2.24E-08 | C8orf34 |
| rs6535224 | 4 | 4q21.21 | T | C | 3.76E-08 | CFAP299;BMP3 |
| rs1495644 | 4 | 4q21.21 | A | C | 2.23E-08 |
| rs6823242 | 4 | 4q21.21 | T | G | 4.01E-08 |
| rs12582259 | 12 | 12p12.1 | G | A | 3.75E-08 | CMAS;ST8SIA1 |
| rs12581049 | 12 | 12p12.1 | C | T | 3.75E-08 |
| rs2325118 | 6 | 6q15 | A | G | 2.29E-08 | CNR1;LOC101928936 |
| rs936495 | 6 | 6q15 | T | G | 2.30E-08 |
| rs9359785 | 6 | 6q15 | T | C | 2.12E-08 |
| rs1359226 | 6 | 6q15 | A | G | 2.12E-08 |
| rs748886 | 11 | 11q14.1 | T | A | 4.12E-08 | DLG2 |
| rs9357284 | 6 | 6p21.2 | G | C | 3.88E-08 | DNAH8 |
| rs2188599 | 7 | 7q31.1 | A | G | 9.44E-09 | DNAJB9;C7orf66 |
| rs1541342 | 7 | 7q31.1 | A | T | 1.15E-08 |
| rs11772153 | 7 | 7q31.1 | A | T | 1.69E-08 |
| rs6973471 | 7 | 7q31.1 | C | G | 1.71E-08 |
| rs720895 | 7 | 7q31.1 | T | G | 2.47E-09 |
| rs17278379 | 1 | 1q24.3 | C | T | 2.27E-08 | DNM3 |
| rs11054481 | 12 | 12p13.2 | G | C | 1.30E-08 | ETV6 |
| rs12335144 | 8 | 8q13.3 | C | T | 1.22E-09 | EYA1 |
| rs859668 | 1 | 1q24.3 | C | T | 4.69E-08 | FASLG;TNFSF18 |
| rs17061494 | 3 | 3p14.2 | C | T | 1.37E-09 | FHIT |
| rs34272009 | 3 | 3p14.2 | T | A | 9.59E-09 |
| rs62238352 | 3 | 3p14.2 | G | A | 7.85E-09 |
| rs7610652 | 3 | 3p14.2 | G | A | 2.91E-09 |
| rs62238354 | 3 | 3p14.2 | G | C | 2.41E-08 |
| rs12889270 | 14 | 14q32.11 | C | T | 4.99E-08 | FOXN3;EFCAB11 |
| rs62567098 | 9 | 9q21.11 | G | T | 2.08E-08 | FXN;TJP2 |
| rs36107264 | 9 | 9q22.2 | G | A | 2.67E-08 | GADD45G;UNQ6494 |
| rs3906867 | 13 | 13q31.3 | G | A | 4.57E-08 | GPC5;LINC00363 |
| rs1008720 | 10 | 10q26.13 | T | C | 1.21E-08 | GPR26 |
| rs10159973 | 10 | 10q25.2 | C | T | 2.48E-08 | HEAT2;GPAM |
| rs10160166 | 10 | 10q25.2 | G | A | 2.31E-08 |
| rs10017432 | 4 | 4q31.23 | A | G | 1.14E-08 | IQCM;DCLK2 |
| rs12523256 | 5 | 5p15.32 | T | A | 1.89E-08 | IRX1;LINC02114 |
| rs7187952 | 16 | 16q12.2 | G | A | 2.70E-08 | IRX5;IRX6 |
| rs57848528 | 5 | 5q11.2 | G | A | 9.53E-09 | ISL1;LINC02118 |
| rs56882549 | 5 | 5q11.2 | C | T | 1.19E-08 |
| rs16878909 | 5 | 5q11.2 | G | A | 4.62E-09 |
| rs1363999 | 5 | 5q11.2 | G | A | 1.43E-08 |
| rs80099492 | 5 | 5q11.2 | G | T | 2.57E-08 |
| rs11255309 | 10 | 10p14 | T | A | 1.45E-09 | ITIH2 |
| rs2597361 | 8 | 8q24.22 | T | C | 2.44E-08 | KCNQ3;HPYR1 |
| rs78930132 | 4 | 4q34.3 | C | A | 3.24E-09 | LINC00290;LINC02500 |
| rs1937503 | 13 | 13q21.32 | C | T | 1.37E-08 | LINC00364;LINC00550 |
| rs75434353 | 13 | 13q21.32 | C | T | 2.45E-08 |
| rs11620389 | 13 | 13q21.32 | G | A | 2.94E-08 |
| rs140209345 | 13 | 13q21.32 | C | T | 2.93E-08 |
| rs2325056 | 13 | 13q21.32 | A | G | 2.88E-08 |
| rs144242089 | 13 | 13q21.32 | G | A | 4.96E-08 |
| rs141040814 | 13 | 13q21.32 | C | T | 3.28E-08 |
| rs8000754 | 13 | 13q21.32 | A | C | 2.87E-08 |
| rs8000969 | 13 | 13q21.32 | T | C | 2.86E-08 |
| rs140400751 | 13 | 13q21.32 | G | A | 3.25E-08 |
| rs142490605 | 13 | 13q21.32 | C | T | 2.71E-08 |
| rs140329311 | 13 | 13q21.32 | G | A | 2.87E-08 |
| rs76764808 | 13 | 13q21.32 | C | A | 3.45E-08 |
| rs61273843 | 13 | 13q12.12 | G | T | 3.67E-08 | LINC00621;SGCG |
| rs61815777 | 1 | 1q32.2 | C | T | 7.36E-09 | LINC01717;LINC01774 |
| rs11119129 | 1 | 1q32.2 | G | T | 1.98E-08 |
| rs60982145 | 1 | 1q32.2 | G | T | 3.16E-08 |
| rs12410473 | 1 | 1q32.2 | C | A | 2.75E-08 | LINC01774;LINC01696 |
| rs13080791 | 3 | 3p12.2 | C | T | 4.73E-08 | LINC02008;NONE |
| rs73061925 | 3 | 3p22.3 | G | C | 3.48E-08 | LOC101928135 |
| rs60594133 | 3 | 3p22.3 | C | T | 3.36E-08 |
| rs9647406 | 3 | 3p22.3 | G | A | 3.48E-08 |
| rs11129623 | 3 | 3p22.3 | G | T | 3.49E-08 |
| rs11129625 | 3 | 3p22.3 | G | A | 3.49E-08 |
| rs885314 | 3 | 3p22.3 | C | T | 1.30E-08 |
| rs73061946 | 3 | 3p22.3 | G | A | 3.49E-08 |
| rs17032471 | 3 | 3p22.3 | C | T | 1.30E-08 |
| rs73061952 | 3 | 3p22.3 | C | T | 1.30E-08 |
| rs73061956 | 3 | 3p22.3 | C | T | 1.30E-08 |
| rs112741329 | 3 | 3p22.3 | G | A | 1.30E-08 |
| rs141713372 | 3 | 3p22.3 | G | A | 1.30E-08 |
| rs78734224 | 3 | 3p22.3 | C | T | 1.30E-08 |
| rs73061958 | 3 | 3p22.3 | C | T | 3.49E-08 |
| rs60839644 | 3 | 3p22.3 | C | T | 1.31E-08 |
| rs10514688 | 3 | 3p22.3 | C | T | 2.79E-08 |
| rs17032513 | 3 | 3p22.3 | C | T | 1.30E-08 |
| rs6795307 | 3 | 3p22.3 | A | G | 1.63E-08 |
| rs4461374 | 3 | 3p22.3 | A | T | 1.57E-08 |
| rs4267603 | 3 | 3p22.3 | T | C | 1.57E-08 |
| rs9835451 | 3 | 3p22.3 | A | G | 1.58E-08 |
| rs4292188 | 3 | 3p22.3 | T | C | 1.57E-08 |
| rs4305379 | 3 | 3p22.3 | T | C | 1.58E-08 |
| rs9843613 | 3 | 3p22.3 | A | T | 1.58E-08 |
| rs11129626 | 3 | 3p22.3 | A | G | 1.58E-08 |
| rs4678726 | 3 | 3p22.3 | A | G | 1.58E-08 |
| rs11676655 | 2 | 2q32.1 | G | C | 2.62E-08 | LOC105373782;FSIP2-AS2 |
| rs6731674 | 2 | 2q32.1 | C | A | 2.62E-08 |
| rs11675629 | 2 | 2q32.1 | T | A | 2.62E-08 |
| rs11681870 | 2 | 2q32.1 | C | A | 2.62E-08 |
| rs34087779 | 2 | 2q32.1 | C | T | 2.61E-08 |
| rs67432566 | 2 | 2q32.1 | T | A | 3.70E-08 |
| rs34571837 | 2 | 2q32.1 | C | T | 3.55E-08 |
| rs35847686 | 2 | 2q32.1 | G | C | 2.59E-08 |
| rs34877204 | 2 | 2q32.1 | G | A | 3.16E-08 |
| rs35289321 | 2 | 2q32.1 | G | A | 6.12E-09 |
| rs11691944 | 2 | 2q32.1 | G | C | 2.61E-09 |
| rs17206943 | 2 | 2q32.1 | G | A | 6.06E-09 |
| rs6704979 | 2 | 2q32.1 | G | A | 6.04E-09 |
| rs113020041 | 2 | 2q32.1 | T | A | 5.97E-09 |
| rs12991386 | 2 | 2q32.1 | C | T | 5.98E-09 |
| rs34539603 | 2 | 2q32.1 | C | T | 1.34E-08 |
| rs12989827 | 2 | 2q32.1 | T | A | 5.93E-09 |
| rs16827022 | 2 | 2q32.1 | G | A | 5.87E-09 |
| rs16827027 | 2 | 2q32.1 | G | A | 5.89E-09 |
| rs34338290 | 2 | 2q32.1 | C | T | 7.26E-09 |
| rs9789468 | 2 | 2q32.1 | C | T | 1.64E-08 |
| rs617867 | 22 | 22q11.21 | C | T | 2.62E-08 | LOC107987389 |
| rs482165 | 22 | 22q11.21 | G | A | 1.84E-08 |
| rs58708909 | 14 | 14q23.3 | G | A | 2.29E-08 | MAX;LINC02324 |
| rs7759891 | 6 | 6q14.1 | G | A | 3.48E-08 | MEI4;MIR10524 |
| rs74342700 | 6 | 6q22.31 | C | T | 3.00E-08 | MIR3144;TBC1D32 |
| rs9387878 | 6 | 6q22.31 | C | A | 1.46E-08 |
| rs17630649 | 6 | 6q22.31 | G | C | 4.45E-08 |
| rs2817943 | 6 | 6q22.31 | C | T | 3.62E-08 |
| rs2817941 | 6 | 6q22.31 | C | T | 3.69E-08 |
| rs10499099 | 6 | 6q22.31 | G | A | 3.17E-08 |
| rs17700692 | 16 | 16q23.1 | C | T | 3.26E-09 | MIR4719;MON1B |
| rs76536469 | 1 | 1p31.3 | G | T | 7.60E-09 | MIR6068;ALG6 |
| rs78527538 | 6 | 6p24.2 | C | T | 6.46E-10 | NEDD9;TMEM170B |
| rs73722961 | 6 | 6p24.2 | C | T | 3.62E-08 |
| rs12524463 | 6 | 6p24.2 | G | A | 3.57E-08 |
| rs7752886 | 6 | 6p24.2 | C | T | 4.41E-08 |
| rs78271362 | 6 | 6p24.2 | C | A | 2.10E-09 |
| rs28179 | 7 | 7p14.3 | A | T | 4.17E-08 | PDE1C |
| rs30589 | 7 | 7p14.3 | A | G | 2.97E-08 |
| rs3738126 | 1 | 1p36.12 | C | T | 1.38E-08 | PLA2G2F |
| rs7573051 | 2 | 2p23.2 | C | T | 2.35E-08 | PLB1 |
| rs35990269 | 2 | 2p23.2 | G | A | 2.40E-08 |
| rs11901746 | 2 | 2p23.2 | G | A | 3.37E-08 |
| rs11904757 | 2 | 2p23.2 | G | A | 3.06E-08 |
| rs138333841 | 16 | 16q23.3 | G | A | 3.31E-08 | PLCG2 |
| rs76574841 | 16 | 16q23.3 | G | A | 2.06E-08 |
| rs76424446 | 16 | 16q23.3 | G | C | 2.21E-08 |
| rs55901360 | 16 | 16q23.3 | C | T | 4.89E-08 |
| rs146473829 | 16 | 16q23.3 | G | C | 3.37E-08 |
| rs17105175 | 5 | 5q32 | G | C | 3.65E-08 | PPP2R2B |
| rs61828878 | 1 | 1q25.1 | G | A | 4.54E-09 | PRDX6;SLC9C2 |
| rs6574036 | 14 | 14q24.2 | A | G | 3.49E-08 | RGS6 |
| rs7161413 | 14 | 14q24.2 | A | G | 2.04E-08 |
| rs8011972 | 14 | 14q24.2 | A | G | 1.15E-09 |
| rs917392 | 14 | 14q24.2 | A | T | 4.76E-09 |
| rs8004427 | 14 | 14q24.2 | C | G | 5.55E-09 |
| rs4902968 | 14 | 14q24.2 | C | G | 5.62E-09 |
| rs4902970 | 14 | 14q24.2 | C | G | 5.82E-09 |
| rs80219778 | 5 | 5p13.1 | C | T | 3.78E-08 | RICTOR;FYB1 |
| rs10511124 | 3 | 3p12.3 | G | C | 1.24E-08 | ROBO1 |
| rs72950623 | 2 | 2q35 | G | T | 4.46E-09 | RPL37A;LINC01280 |
| rs72950645 | 2 | 2q35 | G | A | 4.57E-09 |
| rs72838866 | 6 | 6p22.2 | G | A | 4.32E-09 | SCGN |
| rs2383052 | 9 | 9p22.2 | A | G | 3.33E-08 | SH3GL2;ADAMTSL1 |
| rs10733345 | 9 | 9p22.2 | A | G | 2.75E-08 |
| rs10810875 | 9 | 9p22.2 | A | G | 3.98E-08 |
| rs13294429 | 9 | 9p22.2 | C | T | 3.12E-08 |
| rs34423194 | 9 | 9p22.2 | C | A | 2.88E-08 |
| rs13291916 | 9 | 9p22.2 | C | T | 3.53E-08 |
| rs71506851 | 9 | 9p22.2 | C | T | 4.57E-08 |
| rs34560846 | 9 | 9p22.2 | G | A | 6.36E-09 |
| rs12440434 | 15 | 15q21.1 | C | A | 3.71E-08 | SHC4 |
| rs11580117 | 1 | 1q25.1 | C | T | 5.59E-09 | SLC9C2 |
| rs61871478 | 10 | 10q24.31 | C | A | 1.77E-08 | SLF2 |
| rs17558354 | 2 | 2q35 | G | A | 1.17E-08 | SMARCAL1 |
| rs72948608 | 2 | 2q35 | G | C | 1.18E-08 |
| rs17486207 | 2 | 2q35 | G | A | 1.14E-08 |
| rs10498053 | 2 | 2q35 | G | C | 1.14E-08 |
| rs7571069 | 2 | 2q35 | C | T | 1.14E-08 |
| rs72948623 | 2 | 2q35 | G | A | 1.14E-08 |
| rs17558615 | 2 | 2q35 | G | C | 2.56E-08 |
| rs72948630 | 2 | 2q35 | T | A | 1.14E-08 |
| rs17558671 | 2 | 2q35 | C | T | 1.14E-08 |
| rs17486539 | 2 | 2q35 | G | A | 1.13E-08 |
| rs66931264 | 2 | 2q35 | G | A | 1.14E-08 |
| rs2066527 | 2 | 2q35 | C | T | 1.13E-08 |
| rs6744504 | 2 | 2q35 | G | A | 9.14E-09 |
| rs6744656 | 2 | 2q35 | G | A | 1.14E-08 |
| rs7575125 | 2 | 2q35 | G | C | 2.58E-08 |
| rs143290054 | 2 | 2q35 | G | C | 2.57E-08 |
| rs76052387 | 2 | 2q35 | C | T | 2.57E-08 |
| rs114821488 | 2 | 2q35 | T | A | 2.54E-08 |
| rs17559422 | 2 | 2q35 | C | T | 1.13E-08 |
| rs72948699 | 2 | 2q35 | G | T | 1.14E-08 | SMARCAL1;RPL37A |
| rs9444458 | 6 | 6q14.3 | G | A | 3.11E-08 | SNHG5;HTR1E |
| rs12756333 | 1 | 1p12 | G | T | 4.76E-08 | SPAG17;TBX15 |
| rs72643901 | 8 | 8q11.23 | C | T | 1.75E-08 | ST18;ALKAL1 |
| rs10914146 | 1 | 1q25.3 | C | T | 4.28E-08 | STX6 |
| rs243816 | 6 | 6p12.1 | C | T | 8.42E-09 | TINAG;FAM83B |
| rs514947 | 6 | 6p12.1 | G | A | 8.42E-09 |
| rs678451 | 6 | 6p12.1 | C | T | 1.06E-08 |
| rs189655 | 6 | 6p12.1 | C | T | 8.31E-09 |
| rs192158 | 6 | 6p12.1 | G | A | 1.05E-08 |
| rs750654 | 12 | 12q24.31 | G | C | 2.63E-09 | TMEM132B |
| rs2088708 | 12 | 12q24.31 | G | A | 2.53E-10 |
| rs73233354 | 12 | 12q24.31 | G | A | 4.52E-10 |
| rs12422423 | 12 | 12q24.31 | T | A | 9.42E-11 |
| rs73233358 | 12 | 12q24.31 | T | A | 9.39E-11 |
| rs73233359 | 12 | 12q24.31 | C | T | 9.35E-11 |
| rs2344687 | 12 | 12q24.31 | G | A | 6.88E-11 |
| rs73233361 | 12 | 12q24.31 | C | T | 6.78E-11 |
| rs1883636 | 6 | 6p21.31 | G | A | 1.62E-08 | TULP1 |
| rs6928735 | 6 | 6p21.31 | G | T | 1.10E-08 |
| rs2067997 | 6 | 6p21.31 | C | T | 1.10E-08 |
| rs7768844 | 6 | 6p21.31 | G | A | 1.58E-08 |
| rs1385677 | 4 | 4q22.3 | G | A | 4.01E-08 | UNC5C;PDHA2 |
| rs9942142 | 4 | 4q22.3 | C | T | 4.13E-08 |
| rs35233677 | 9 | 9q34.2 | G | T | 3.01E-08 | VAV2 |
| rs3744732 | 17 | 17p13.3 | G | A | 5.37E-10 | VPS53 |
| rs12633205 | 3 | 3q29 | C | T | 2.23E-08 | XXYLT1 |
| rs17828145 | 9 | 9q32 | G | A | 2.15E-08 | ZFP37;FAM225B |
| rs62117242 | 19 | 19q13.41 | C | T | 2.47E-08 | ZNF701 |

**Supplementary Table 2** DisGeNET enrichment results for genes shared by NAFLD and T2D

| GO | Description | Count | % | Hits | Log10(P) | Log10(q) |
| --- | --- | --- | --- | --- | --- | --- |
| C0027404 | Narcolepsy | 10 | 3 | DNAH8|GPC5|FHIT|FXN|VAV2|TMEM132B|C8orf34|PLB1|IQCM|SHC4 | -7.1 | -2.9 |
| C0037369 | Smoking | 10 | 3 | FHIT|GNA12|PDE1C|TBX15|PTCD1|DNM3|TMEM132B|XXYLT1|AMZ1|ATP5MF-PTCD1 | -6.1 | -2.1 |
| C4049938 | Physical Activity Measurement | 8 | 2.7 | BCKDHB|EYA1|FHIT|GNA12|TBX15|DNM3|VPS53|AMZ1 | -7.2 | -2.9 |
| C0027092 | Myopia | 8 | 2.7 | BMP3|DLG2|TULP1|TJP2|IRX5|SMARCAL1|ADAMTSL1|ADAMTS18 | -3.6 | -0.32 |
| C0205682 | Waist-Hip Ratio | 8 | 2.7 | BCKDHB|DLG2|EYA1|ROBO1|TBX15|DNM3|IRX1|C8orf34 | -3.2 | -0.14 |
| C1449563 | Cardiomyopathy, Familial Idiopathic | 8 | 2.7 | FASLG|CNR1|DNAH8|FYB1|ISL1|PDE1C|PLB1|ADAMTS18 | -2.4 | 0 |
| C0018798 | Congenital Heart Defects | 7 | 2.6 | FASLG|DNAH8|EYA1|FXN|ISL1|RGS6|IRX5 | -3.4 | -0.22 |
| C0027708 | Nephroblastoma | 7 | 2.6 | FASLG|DLG2|EYA1|MAX|PLCG2|RGS6|IRX5 | -2.5 | 0 |
| C0040034 | Thrombocytopenia | 7 | 2.6 | FASLG|ETV6|FYB1|PRDX6|ARPC1B|DNAAF9|SMARCAL1 | -2.4 | 0 |
| C0236969 | Substance-Related Disorders | 6 | 2.4 | FASLG|CNR1|DNAH8|ETV6|FHIT|PDE1C | -5.4 | -1.4 |
| C1519383 | Smoking Behaviors | 6 | 2.4 | GNA12|PDE1C|TBX15|DNM3|XXYLT1|AMZ1 | -3.7 | -0.36 |
| C0018790 | Cardiac Arrest | 6 | 2.4 | GPC5|FXN|HTR1E|PPP2R2B|DNAAF9|PLB1 | -2.6 | 0 |
| C4551683 | Adrenal Gland Pheochromocytoma | 6 | 2.4 | CNR1|GNA12|ISL1|MAX|RGS6|RICTOR | -2.6 | 0 |
| C0007758 | Cerebellar Ataxia | 6 | 2.4 | FXN|PPP2R2B|SGCG|RGS6|ATP8A2|SLC9C2 | -2.5 | 0 |
| C0029231 | Organic Mental Disorders, Substance-Induced | 5 | 2.2 | CNR1|DNAH8|ETV6|FHIT|PDE1C | -4.4 | -0.82 |
| C4316881 | Prescription Drug Abuse | 5 | 2.2 | CNR1|DNAH8|ETV6|FHIT|PDE1C | -4.4 | -0.82 |
| C0013222 | Drug Use Disorders | 5 | 2.2 | CNR1|DNAH8|ETV6|FHIT|PDE1C | -4.3 | -0.76 |
| C0424678 | Lean body mass | 5 | 2.2 | BCKDHB|ETV6|FHIT|GNA12|AMZ1 | -3.9 | -0.44 |
| C0038580 | Substance Dependence | 5 | 2.2 | CNR1|DNAH8|ETV6|FHIT|PDE1C | -3.8 | -0.36 |
| C0740858 | Substance abuse problem | 5 | 2.2 | CNR1|DNAH8|ETV6|FHIT|PDE1C | -3.4 | -0.22 |
| C0013170 | Drug habituation | 5 | 2.2 | CNR1|DNAH8|ETV6|FHIT|PDE1C | -3.3 | -0.17 |
| C0038586 | Substance Use Disorders | 5 | 2.2 | CNR1|DNAH8|ETV6|FHIT|PDE1C | -3.1 | -0.051 |
| C1510472 | Drug Dependence | 5 | 2.2 | CNR1|DNAH8|ETV6|FHIT|PDE1C | -2.8 | 0 |
| C0489786 | Height | 5 | 2.2 | BCKDHB|ETV6|GNA12|DNM3|AMZ1 | -2.8 | 0 |
| C0022665 | Kidney Neoplasm | 5 | 2.2 | DLG2|FHIT|MAX|PLCG2|DNM3 | -2.5 | 0 |
| C0740279 | Cerebellar atrophy | 5 | 2.2 | FXN|PPP2R2B|ATP8A2|VPS53|PLB1 | -2.4 | 0 |
| C0242422 | Parkinsonian Disorders | 5 | 2.2 | CNR1|PPP2R2B|ST8SIA1|DNM3|PLB1 | -2.1 | 0 |
| C0017658 | Glomerulonephritis | 5 | 2.2 | FASLG|ETV6|MAX|DNAJB9|PLB1 | -2 | 0 |
| C0009691 | Congenital cataract | 4 | 2 | EYA1|GPC5|RGS6|SMARCAL1 | -3.4 | -0.22 |
| C0009021 | Clonorchiasis | 3 | 1.7 | FASLG|PRDX6|PLB1 | -4.8 | -0.99 |
| C0201983 | Dehydroepiandrosterone sulfate measurement (procedure) | 3 | 1.7 | ARPC1B|PTCD1|ATP5MF-PTCD1 | -4.6 | -0.82 |
| C0265338 | Coffin-Siris syndrome | 3 | 1.7 | FASLG|SMARCAL1|CMAS | -3.1 | -0.038 |
| C1839780 | FRAGILE X TREMOR/ATAXIA SYNDROME | 3 | 1.7 | FXN|PPP2R2B|PLB1 | -2.9 | 0 |
| C1285498 | Vegetation | 3 | 1.7 | GNA12|MON1B|HEAT2 | -2.8 | 0 |
| C0016719 | Friedreich Ataxia | 3 | 1.7 | FXN|PPP2R2B|TJP2 | -2.5 | 0 |
| C0752120 | Spinocerebellar Ataxia Type 1 | 3 | 1.7 | ETV6|FXN|PPP2R2B | -2.3 | 0 |
| C3642346 | Luminal B Breast Carcinoma | 3 | 1.7 | EYA1|ISL1|RICTOR | -2.3 | 0 |
| C0239981 | Hypoalbuminemia | 3 | 1.7 | FHIT|FXN|PLB1 | -2.3 | 0 |
| C0162701 | Polysomnography | 3 | 1.7 | GPC5|C8orf34|RICTOR | -2.1 | 0 |
| C0239998 | Recurrent infections | 3 | 1.7 | PLCG2|ARPC1B|SMARCAL1 | -2.1 | 0 |

**Supplementary Table 3** GO enrichment results for genes shared by NAFLD and T2D

| ONTOLOGY | ID | Description | pvalue | p.adjust | qvalue | geneID | Count[[1]](#footnote-0) |
| --- | --- | --- | --- | --- | --- | --- | --- |
| BP | GO:0050731 | positive regulation of peptidyl-tyrosine phosphorylation | 0.000156935 | 0.137297913 | 0.129922371 | TNFSF18/RICTOR/ISL1/NEDD9/ALKAL1/PLCG2 | 6 |
| BP | GO:0040014 | regulation of multicellular organism growth | 0.000157633 | 0.137297913 | 0.129922371 | FXN/GPAM/ATP8A2/ATRN | 4 |
| BP | GO:0060914 | heart formation | 0.000417568 | 0.176413468 | 0.166936668 | ROBO1/ISL1/EYA1 | 3 |
| BP | GO:0046471 | phosphatidylglycerol metabolic process | 0.000454162 | 0.176413468 | 0.166936668 | PLA2G2F/PLB1/GPAM | 3 |
| BP | GO:0003266 | regulation of secondary heart field cardioblast proliferation | 0.000759376 | 0.176413468 | 0.166936668 | ISL1/EYA1 | 2 |
| BP | GO:0050730 | regulation of peptidyl-tyrosine phosphorylation | 0.000864224 | 0.176413468 | 0.166936668 | TNFSF18/RICTOR/ISL1/NEDD9/ALKAL1/PLCG2 | 6 |
| BP | GO:0046710 | GDP metabolic process | 0.000925612 | 0.176413468 | 0.166936668 | TJP2/DLG2 | 2 |
| BP | GO:0003263 | cardioblast proliferation | 0.001107725 | 0.176413468 | 0.166936668 | ISL1/EYA1 | 2 |
| BP | GO:0003264 | regulation of cardioblast proliferation | 0.001107725 | 0.176413468 | 0.166936668 | ISL1/EYA1 | 2 |
| BP | GO:0098840 | protein transport along microtubule | 0.001305585 | 0.176413468 | 0.166936668 | SPAG17/DLG2 | 2 |
| BP | GO:0099118 | microtubule-based protein transport | 0.001305585 | 0.176413468 | 0.166936668 | SPAG17/DLG2 | 2 |
| BP | GO:0048880 | sensory system development | 0.001371877 | 0.176413468 | 0.166936668 | FASLG/ISL1/TULP1/TBC1D32/ATP8A2/MAX/ADAMTS18 | 7 |
| BP | GO:0009395 | phospholipid catabolic process | 0.0014948 | 0.176413468 | 0.166936668 | PRDX6/PLB1/PLCG2 | 3 |
| BP | GO:0036151 | phosphatidylcholine acyl-chain remodeling | 0.00151906 | 0.176413468 | 0.166936668 | PLA2G2F/PLB1 | 2 |
| BP | GO:2001140 | positive regulation of phospholipid transport | 0.00151906 | 0.176413468 | 0.166936668 | FASLG/ATP8A2 | 2 |
| BP | GO:2001138 | regulation of phospholipid transport | 0.001748018 | 0.190315474 | 0.180091868 | FASLG/ATP8A2 | 2 |
| BP | GO:0003128 | heart field specification | 0.002526503 | 0.244509315 | 0.231374456 | ROBO1/ISL1 | 2 |
| BP | GO:0050884 | neuromuscular process controlling posture | 0.002526503 | 0.244509315 | 0.231374456 | FXN/ATP8A2 | 2 |
| BP | GO:2000136 | regulation of cell proliferation involved in heart morphogenesis | 0.002816106 | 0.25282252 | 0.239241082 | ISL1/EYA1 | 2 |
| BP | GO:0048645 | animal organ formation | 0.00301894 | 0.25282252 | 0.239241082 | ROBO1/ISL1/EYA1 | 3 |
| BP | GO:0061323 | cell proliferation involved in heart morphogenesis | 0.003120553 | 0.25282252 | 0.239241082 | ISL1/EYA1 | 2 |
| BP | GO:0035264 | multicellular organism growth | 0.003357948 | 0.25282252 | 0.239241082 | FXN/GPAM/ATP8A2/ATRN | 4 |
| BP | GO:1904888 | cranial skeletal system development | 0.003541171 | 0.25282252 | 0.239241082 | TBX15/FOXN3/IRX5 | 3 |
| BP | GO:0031346 | positive regulation of cell projection organization | 0.003547957 | 0.25282252 | 0.239241082 | DNM3/ROBO1/NEDD9/CNR1/ALKAL1/ATP8A2 | 6 |
| BP | GO:0050908 | detection of light stimulus involved in visual perception | 0.00377347 | 0.25282252 | 0.239241082 | TULP1/ATP8A2 | 2 |
| BP | GO:0050962 | detection of light stimulus involved in sensory perception | 0.00377347 | 0.25282252 | 0.239241082 | TULP1/ATP8A2 | 2 |
| BP | GO:0060041 | retina development in camera-type eye | 0.004440383 | 0.260800627 | 0.246790611 | TULP1/TBC1D32/ATP8A2/MAX | 4 |
| BP | GO:0010623 | programmed cell death involved in cell development | 0.004484257 | 0.260800627 | 0.246790611 | FASLG/UNC5C | 2 |
| BP | GO:0038095 | Fc-epsilon receptor signaling pathway | 0.004484257 | 0.260800627 | 0.246790611 | VAV2/PLCG2 | 2 |
| BP | GO:0003151 | outflow tract morphogenesis | 0.004581143 | 0.260800627 | 0.246790611 | ROBO1/ISL1/EYA1 | 3 |
| BP | GO:0046434 | organophosphate catabolic process | 0.004641113 | 0.260800627 | 0.246790611 | PRDX6/PLB1/FHIT/PLCG2 | 4 |
| BP | GO:0003148 | outflow tract septum morphogenesis | 0.004861043 | 0.262342224 | 0.248249394 | ROBO1/ISL1 | 2 |
| BP | GO:0018108 | peptidyl-tyrosine phosphorylation | 0.005139993 | 0.262342224 | 0.248249394 | TNFSF18/RICTOR/ISL1/NEDD9/ALKAL1/PLCG2 | 6 |
| BP | GO:0046339 | diacylglycerol metabolic process | 0.005251928 | 0.262342224 | 0.248249394 | PLB1/GPAM | 2 |
| BP | GO:0018212 | peptidyl-tyrosine modification | 0.00527094 | 0.262342224 | 0.248249394 | TNFSF18/RICTOR/ISL1/NEDD9/ALKAL1/PLCG2 | 6 |
| BP | GO:0046037 | GMP metabolic process | 0.00565679 | 0.267350934 | 0.25298904 | TJP2/DLG2 | 2 |
| BP | GO:0001654 | eye development | 0.005678522 | 0.267350934 | 0.25298904 | FASLG/TULP1/TBC1D32/ATP8A2/MAX/ADAMTS18 | 6 |
| BP | GO:0150063 | visual system development | 0.00596279 | 0.273346835 | 0.258662846 | FASLG/TULP1/TBC1D32/ATP8A2/MAX/ADAMTS18 | 6 |
| BP | GO:0048678 | response to axon injury | 0.00616464 | 0.275353902 | 0.260562095 | ISL1/MAX/PLCG2 | 3 |
| BP | GO:0060037 | pharyngeal system development | 0.006507963 | 0.28342177 | 0.268196563 | ISL1/EYA1 | 2 |
| BP | GO:0048704 | embryonic skeletal system morphogenesis | 0.007168894 | 0.300337266 | 0.284203371 | TBX15/EYA1/IRX5 | 3 |
| BP | GO:0043254 | regulation of protein-containing complex assembly | 0.007372331 | 0.300337266 | 0.284203371 | TNFSF18/RICTOR/ISL1/ARPC1B/SLF2/PLCG2 | 6 |
| BP | GO:0040018 | positive regulation of multicellular organism growth | 0.007413606 | 0.300337266 | 0.284203371 | GPAM/ATP8A2 | 2 |
| BP | GO:0048665 | neuron fate specification | 0.007886559 | 0.312236056 | 0.295462967 | ISL1/EYA1 | 2 |
| BP | GO:0046475 | glycerophospholipid catabolic process | 0.008872143 | 0.343450494 | 0.325000589 | PRDX6/PLB1 | 2 |
| BP | GO:0035633 | maintenance of blood-brain barrier | 0.009384541 | 0.355388485 | 0.336297279 | SH3GL2/TJP2 | 2 |
| BP | GO:0006650 | glycerophospholipid metabolic process | 0.009764644 | 0.361915085 | 0.342473276 | PLA2G2F/PRDX6/PLB1/GPAM/PLCG2 | 5 |
| BP | GO:0010092 | specification of animal organ identity | 0.010447978 | 0.379174519 | 0.358805545 | ROBO1/ISL1 | 2 |
| BP | GO:0001708 | cell fate specification | 0.0107383 | 0.381757532 | 0.361249801 | TBX15/ISL1/EYA1 | 3 |
| BP | GO:0003203 | endocardial cushion morphogenesis | 0.010998788 | 0.383197784 | 0.362612683 | ROBO1/ISL1 | 2 |
| BP | GO:0060249 | anatomical structure homeostasis | 0.012834831 | 0.41924467 | 0.396723157 | SCGN/TULP1/SH3GL2/FXN/TJP2 | 5 |
| BP | GO:0016042 | lipid catabolic process | 0.013298786 | 0.41924467 | 0.396723157 | PLA2G2F/PRDX6/PLB1/CNR1/PLCG2 | 5 |
| BP | GO:0009167 | purine ribonucleoside monophosphate metabolic process | 0.013326693 | 0.41924467 | 0.396723157 | TJP2/DLG2 | 2 |
| BP | GO:0032735 | positive regulation of interleukin-12 production | 0.013326693 | 0.41924467 | 0.396723157 | ISL1/PLCG2 | 2 |
| BP | GO:0050866 | negative regulation of cell activation | 0.013804691 | 0.41924467 | 0.396723157 | PLA2G2F/TNFSF18/CNR1/ADAMTS18 | 4 |
| BP | GO:0044242 | cellular lipid catabolic process | 0.014014343 | 0.41924467 | 0.396723157 | PRDX6/PLB1/CNR1/PLCG2 | 4 |
| BP | GO:0009126 | purine nucleoside monophosphate metabolic process | 0.014563892 | 0.41924467 | 0.396723157 | TJP2/DLG2 | 2 |
| BP | GO:0007596 | blood coagulation | 0.014655437 | 0.41924467 | 0.396723157 | GNA12/VAV2/ADAMTS18/PLCG2 | 4 |
| BP | GO:0048705 | skeletal system morphogenesis | 0.01509301 | 0.41924467 | 0.396723157 | TBX15/EYA1/FOXN3/IRX5 | 4 |
| BP | GO:0043280 | positive regulation of cysteine-type endopeptidase activity involved in apoptotic process | 0.015500802 | 0.41924467 | 0.396723157 | FASLG/ROBO1/ST18 | 3 |
| BP | GO:0008037 | cell recognition | 0.015764753 | 0.41924467 | 0.396723157 | ROBO1/NEDD9/TULP1/CNR1 | 4 |
| BP | GO:0050817 | coagulation | 0.015764753 | 0.41924467 | 0.396723157 | GNA12/VAV2/ADAMTS18/PLCG2 | 4 |
| BP | GO:0048701 | embryonic cranial skeleton morphogenesis | 0.015848756 | 0.41924467 | 0.396723157 | TBX15/IRX5 | 2 |
| BP | GO:0007599 | hemostasis | 0.015992793 | 0.41924467 | 0.396723157 | GNA12/VAV2/ADAMTS18/PLCG2 | 4 |
| BP | GO:0048706 | embryonic skeletal system development | 0.016506265 | 0.41924467 | 0.396723157 | TBX15/EYA1/IRX5 | 3 |
| BP | GO:0050919 | negative chemotaxis | 0.016508794 | 0.41924467 | 0.396723157 | ROBO1/UNC5C | 2 |
| BP | GO:0003197 | endocardial cushion development | 0.017180426 | 0.41924467 | 0.396723157 | ROBO1/ISL1 | 2 |
| BP | GO:0008038 | neuron recognition | 0.017180426 | 0.41924467 | 0.396723157 | ROBO1/CNR1 | 2 |
| BP | GO:0043114 | regulation of vascular permeability | 0.017863548 | 0.41924467 | 0.396723157 | SH3GL2/TJP2 | 2 |
| BP | GO:0030168 | platelet activation | 0.018261856 | 0.41924467 | 0.396723157 | VAV2/ADAMTS18/PLCG2 | 3 |
| BP | GO:0007188 | adenylate cyclase-modulating G protein-coupled receptor signaling pathway | 0.020190934 | 0.41924467 | 0.396723157 | HTR1E/CNR1/GNA12/GPR26 | 4 |
| BP | GO:0009584 | detection of visible light | 0.020708828 | 0.41924467 | 0.396723157 | TULP1/ATP8A2 | 2 |
| BP | GO:0045332 | phospholipid translocation | 0.020708828 | 0.41924467 | 0.396723157 | FASLG/ATP8A2 | 2 |
| BP | GO:2001056 | positive regulation of cysteine-type endopeptidase activity | 0.022073766 | 0.41924467 | 0.396723157 | FASLG/ROBO1/ST18 | 3 |
| BP | GO:0038093 | Fc receptor signaling pathway | 0.022197698 | 0.41924467 | 0.396723157 | VAV2/PLCG2 | 2 |
| BP | GO:0072132 | mesenchyme morphogenesis | 0.022197698 | 0.41924467 | 0.396723157 | ROBO1/ISL1 | 2 |
| BP | GO:0042733 | embryonic digit morphogenesis | 0.022958333 | 0.41924467 | 0.396723157 | TBC1D32/GNA12 | 2 |
| BP | GO:0006644 | phospholipid metabolic process | 0.023448842 | 0.41924467 | 0.396723157 | PLA2G2F/PRDX6/PLB1/GPAM/PLCG2 | 5 |
| BP | GO:0070231 | T cell apoptotic process | 0.023729634 | 0.41924467 | 0.396723157 | FASLG/GPAM | 2 |
| BP | GO:0045927 | positive regulation of growth | 0.023820695 | 0.41924467 | 0.396723157 | RICTOR/FXN/GPAM/ATP8A2 | 4 |
| BP | GO:0034204 | lipid translocation | 0.0245115 | 0.41924467 | 0.396723157 | FASLG/ATP8A2 | 2 |
| BP | GO:0009161 | ribonucleoside monophosphate metabolic process | 0.025303832 | 0.41924467 | 0.396723157 | TJP2/DLG2 | 2 |
| BP | GO:0061098 | positive regulation of protein tyrosine kinase activity | 0.025303832 | 0.41924467 | 0.396723157 | NEDD9/ALKAL1 | 2 |
| BP | GO:0046486 | glycerolipid metabolic process | 0.025803889 | 0.41924467 | 0.396723157 | PLA2G2F/PRDX6/PLB1/GPAM/PLCG2 | 5 |
| BP | GO:0050729 | positive regulation of inflammatory response | 0.026731297 | 0.41924467 | 0.396723157 | TNFSF18/CNR1/PLCG2 | 3 |
| BP | GO:0090596 | sensory organ morphogenesis | 0.027183288 | 0.41924467 | 0.396723157 | FASLG/TULP1/EYA1/ATP8A2 | 4 |
| BP | GO:0035904 | aorta development | 0.027742642 | 0.41924467 | 0.396723157 | ROBO1/EYA1 | 2 |
| BP | GO:0010976 | positive regulation of neuron projection development | 0.028085907 | 0.41924467 | 0.396723157 | CNR1/ALKAL1/ATP8A2 | 3 |
| BP | GO:0032615 | interleukin-12 production | 0.028575858 | 0.41924467 | 0.396723157 | ISL1/PLCG2 | 2 |
| BP | GO:0032655 | regulation of interleukin-12 production | 0.028575858 | 0.41924467 | 0.396723157 | ISL1/PLCG2 | 2 |
| BP | GO:0097035 | regulation of membrane lipid distribution | 0.028575858 | 0.41924467 | 0.396723157 | FASLG/ATP8A2 | 2 |
| BP | GO:0048592 | eye morphogenesis | 0.029009021 | 0.41924467 | 0.396723157 | FASLG/TULP1/ATP8A2 | 3 |
| BP | GO:0016925 | protein sumoylation | 0.029419053 | 0.41924467 | 0.396723157 | EYA1/SLF2 | 2 |
| BP | GO:0050727 | regulation of inflammatory response | 0.029613532 | 0.41924467 | 0.396723157 | TNFSF18/RICTOR/ISL1/CNR1/PLCG2 | 5 |
| BP | GO:0045165 | cell fate commitment | 0.030475642 | 0.41924467 | 0.396723157 | TBX15/TNFSF18/ISL1/EYA1 | 4 |
| BP | GO:0048488 | synaptic vesicle endocytosis | 0.031134996 | 0.41924467 | 0.396723157 | DNM3/SH3GL2 | 2 |
| BP | GO:0140238 | presynaptic endocytosis | 0.031134996 | 0.41924467 | 0.396723157 | DNM3/SH3GL2 | 2 |
| BP | GO:0007187 | G protein-coupled receptor signaling pathway, coupled to cyclic nucleotide second messenger | 0.032007555 | 0.41924467 | 0.396723157 | HTR1E/CNR1 | 2 |
| BP | GO:0042531 | positive regulation of tyrosine phosphorylation of STAT protein | 0.032007555 | 0.41924467 | 0.396723157 | TNFSF18/ISL1 | 2 |
| BP | GO:0009583 | detection of light stimulus | 0.032889713 | 0.41924467 | 0.396723157 | TULP1/ATP8A2 | 2 |
| BP | GO:0046503 | glycerolipid catabolic process | 0.032889713 | 0.41924467 | 0.396723157 | PRDX6/PLB1 | 2 |
| BP | GO:0048663 | neuron fate commitment | 0.032889713 | 0.41924467 | 0.396723157 | ISL1/EYA1 | 2 |
| BP | GO:0007568 | aging | 0.033864132 | 0.41924467 | 0.396723157 | CNR1/GNA12/ATP8A2 | 3 |
| BP | GO:0061512 | protein localization to cilium | 0.034682457 | 0.41924467 | 0.396723157 | TULP1/TBC1D32 | 2 |
| BP | GO:0048562 | embryonic organ morphogenesis | 0.034725902 | 0.41924467 | 0.396723157 | TBX15/EYA1/ATP8A2/IRX5 | 4 |
| BP | GO:0010950 | positive regulation of endopeptidase activity | 0.036440532 | 0.41924467 | 0.396723157 | FASLG/ROBO1/ST18 | 3 |
| BP | GO:0060411 | cardiac septum morphogenesis | 0.036512487 | 0.41924467 | 0.396723157 | ROBO1/ISL1 | 2 |
| BP | GO:0071677 | positive regulation of mononuclear cell migration | 0.037441257 | 0.41924467 | 0.396723157 | TNFSF18/NEDD9 | 2 |
| BP | GO:0050766 | positive regulation of phagocytosis | 0.038379075 | 0.41924467 | 0.396723157 | TULP1/PLCG2 | 2 |
| BP | GO:0031503 | protein-containing complex localization | 0.038572526 | 0.41924467 | 0.396723157 | SPAG17/DNM3/DLG2 | 3 |
| BP | GO:0010975 | regulation of neuron projection development | 0.03888574 | 0.41924467 | 0.396723157 | DNM3/ROBO1/CNR1/ALKAL1/ATP8A2 | 5 |
| BP | GO:0072078 | nephron tubule morphogenesis | 0.039325852 | 0.41924467 | 0.396723157 | IRX1/EYA1 | 2 |
| BP | GO:0042129 | regulation of T cell proliferation | 0.040767102 | 0.41924467 | 0.396723157 | PLA2G2F/TNFSF18/GPAM | 3 |
| BP | GO:0002084 | protein depalmitoylation | 0.041024619 | 0.41924467 | 0.396723157 | ABHD10 | 1 |
| BP | GO:0003129 | heart induction | 0.041024619 | 0.41924467 | 0.396723157 | ROBO1 | 1 |
| BP | GO:0006655 | phosphatidylglycerol biosynthetic process | 0.041024619 | 0.41924467 | 0.396723157 | GPAM | 1 |
| BP | GO:0038007 | netrin-activated signaling pathway | 0.041024619 | 0.41924467 | 0.396723157 | UNC5C | 1 |
| BP | GO:0038171 | cannabinoid signaling pathway | 0.041024619 | 0.41924467 | 0.396723157 | CNR1 | 1 |
| BP | GO:0060920 | cardiac pacemaker cell differentiation | 0.041024619 | 0.41924467 | 0.396723157 | ISL1 | 1 |
| BP | GO:0070099 | regulation of chemokine-mediated signaling pathway | 0.041024619 | 0.41924467 | 0.396723157 | ROBO1 | 1 |
| BP | GO:0071600 | otic vesicle morphogenesis | 0.041024619 | 0.41924467 | 0.396723157 | EYA1 | 1 |
| BP | GO:0090557 | establishment of endothelial intestinal barrier | 0.041024619 | 0.41924467 | 0.396723157 | TJP2 | 1 |
| BP | GO:1905383 | protein localization to presynapse | 0.041024619 | 0.41924467 | 0.396723157 | DLG2 | 1 |
| BP | GO:2001269 | positive regulation of cysteine-type endopeptidase activity involved in apoptotic signaling pathway | 0.041024619 | 0.41924467 | 0.396723157 | ST18 | 1 |
| BP | GO:0006919 | activation of cysteine-type endopeptidase activity involved in apoptotic process | 0.041245929 | 0.41924467 | 0.396723157 | FASLG/ROBO1 | 2 |
| BP | GO:0009123 | nucleoside monophosphate metabolic process | 0.041245929 | 0.41924467 | 0.396723157 | TJP2/DLG2 | 2 |
| BP | GO:0046470 | phosphatidylcholine metabolic process | 0.041245929 | 0.41924467 | 0.396723157 | PLA2G2F/PLB1 | 2 |
| BP | GO:0072088 | nephron epithelium morphogenesis | 0.041245929 | 0.41924467 | 0.396723157 | IRX1/EYA1 | 2 |
| BP | GO:0036465 | synaptic vesicle recycling | 0.043200779 | 0.41924467 | 0.396723157 | DNM3/SH3GL2 | 2 |
| BP | GO:0061333 | renal tubule morphogenesis | 0.043200779 | 0.41924467 | 0.396723157 | IRX1/EYA1 | 2 |
| BP | GO:0070227 | lymphocyte apoptotic process | 0.044191026 | 0.41924467 | 0.396723157 | FASLG/GPAM | 2 |
| BP | GO:0072028 | nephron morphogenesis | 0.044191026 | 0.41924467 | 0.396723157 | IRX1/EYA1 | 2 |
| BP | GO:0001660 | fever generation | 0.04503452 | 0.41924467 | 0.396723157 | CNR1 | 1 |
| BP | GO:0002732 | positive regulation of dendritic cell cytokine production | 0.04503452 | 0.41924467 | 0.396723157 | PLCG2 | 1 |
| BP | GO:0003139 | secondary heart field specification | 0.04503452 | 0.41924467 | 0.396723157 | ISL1 | 1 |
| BP | GO:0006054 | N-acetylneuraminate metabolic process | 0.04503452 | 0.41924467 | 0.396723157 | CMAS | 1 |
| BP | GO:0006086 | acetyl-CoA biosynthetic process from pyruvate | 0.04503452 | 0.41924467 | 0.396723157 | PDHA2 | 1 |
| BP | GO:0021559 | trigeminal nerve development | 0.04503452 | 0.41924467 | 0.396723157 | ISL1 | 1 |
| BP | GO:0021860 | pyramidal neuron development | 0.04503452 | 0.41924467 | 0.396723157 | DCLK2 | 1 |
| BP | GO:0021889 | olfactory bulb interneuron differentiation | 0.04503452 | 0.41924467 | 0.396723157 | ROBO1 | 1 |
| BP | GO:0030320 | cellular monovalent inorganic anion homeostasis | 0.04503452 | 0.41924467 | 0.396723157 | FASLG | 1 |
| BP | GO:0030644 | cellular chloride ion homeostasis | 0.04503452 | 0.41924467 | 0.396723157 | FASLG | 1 |
| BP | GO:0032610 | interleukin-1 alpha production | 0.04503452 | 0.41924467 | 0.396723157 | ISL1 | 1 |
| BP | GO:0032650 | regulation of interleukin-1 alpha production | 0.04503452 | 0.41924467 | 0.396723157 | ISL1 | 1 |
| BP | GO:0036152 | phosphatidylethanolamine acyl-chain remodeling | 0.04503452 | 0.41924467 | 0.396723157 | PLA2G2F | 1 |
| BP | GO:0043084 | penile erection | 0.04503452 | 0.41924467 | 0.396723157 | CNR1 | 1 |
| BP | GO:0052646 | alditol phosphate metabolic process | 0.04503452 | 0.41924467 | 0.396723157 | GPAM | 1 |
| BP | GO:0060081 | membrane hyperpolarization | 0.04503452 | 0.41924467 | 0.396723157 | KCNQ3 | 1 |
| BP | GO:0090527 | actin filament reorganization | 0.04503452 | 0.41924467 | 0.396723157 | NEDD9 | 1 |
| BP | GO:2000320 | negative regulation of T-helper 17 cell differentiation | 0.04503452 | 0.41924467 | 0.396723157 | TNFSF18 | 1 |
| BP | GO:2000826 | regulation of heart morphogenesis | 0.04503452 | 0.41924467 | 0.396723157 | ROBO1 | 1 |
| BP | GO:0097061 | dendritic spine organization | 0.045189706 | 0.41924467 | 0.396723157 | DNM3/NEDD9 | 2 |
| BP | GO:0042509 | regulation of tyrosine phosphorylation of STAT protein | 0.046196732 | 0.41924467 | 0.396723157 | TNFSF18/ISL1 | 2 |
| BP | GO:0031334 | positive regulation of protein-containing complex assembly | 0.04652445 | 0.41924467 | 0.396723157 | RICTOR/SLF2/PLCG2 | 3 |
| BP | GO:0099504 | synaptic vesicle cycle | 0.04652445 | 0.41924467 | 0.396723157 | DNM3/CNR1/SH3GL2 | 3 |
| BP | GO:0007389 | pattern specification process | 0.047599851 | 0.41924467 | 0.396723157 | ROBO1/IRX1/ISL1/TBC1D32/EYA1 | 5 |
| BP | GO:0010952 | positive regulation of peptidase activity | 0.047721874 | 0.41924467 | 0.396723157 | FASLG/ROBO1/ST18 | 3 |
| BP | GO:0003163 | sinoatrial node development | 0.049027865 | 0.41924467 | 0.396723157 | ISL1 | 1 |
| BP | GO:0010454 | negative regulation of cell fate commitment | 0.049027865 | 0.41924467 | 0.396723157 | TNFSF18 | 1 |
| BP | GO:0031650 | regulation of heat generation | 0.049027865 | 0.41924467 | 0.396723157 | CNR1 | 1 |
| BP | GO:0032957 | inositol trisphosphate metabolic process | 0.049027865 | 0.41924467 | 0.396723157 | PLCG2 | 1 |
| BP | GO:0034086 | maintenance of sister chromatid cohesion | 0.049027865 | 0.41924467 | 0.396723157 | SLF2 | 1 |
| BP | GO:0034088 | maintenance of mitotic sister chromatid cohesion | 0.049027865 | 0.41924467 | 0.396723157 | SLF2 | 1 |
| BP | GO:0040015 | negative regulation of multicellular organism growth | 0.049027865 | 0.41924467 | 0.396723157 | FXN | 1 |
| BP | GO:0051001 | negative regulation of nitric-oxide synthase activity | 0.049027865 | 0.41924467 | 0.396723157 | CNR1 | 1 |
| BP | GO:0060379 | cardiac muscle cell myoblast differentiation | 0.049027865 | 0.41924467 | 0.396723157 | ISL1 | 1 |
| BP | GO:0072070 | loop of Henle development | 0.049027865 | 0.41924467 | 0.396723157 | IRX1 | 1 |
| BP | GO:0090331 | negative regulation of platelet aggregation | 0.049027865 | 0.41924467 | 0.396723157 | ADAMTS18 | 1 |
| BP | GO:0099509 | regulation of presynaptic cytosolic calcium ion concentration | 0.049027865 | 0.41924467 | 0.396723157 | SCGN | 1 |
| BP | GO:1900227 | positive regulation of NLRP3 inflammasome complex assembly | 0.049027865 | 0.41924467 | 0.396723157 | PLCG2 | 1 |
| BP | GO:1902950 | regulation of dendritic spine maintenance | 0.049027865 | 0.41924467 | 0.396723157 | NEDD9 | 1 |
| BP | GO:2000317 | negative regulation of T-helper 17 type immune response | 0.049027865 | 0.41924467 | 0.396723157 | TNFSF18 | 1 |
| BP | GO:2000344 | positive regulation of acrosome reaction | 0.049027865 | 0.41924467 | 0.396723157 | PLB1 | 1 |
| BP | GO:2000508 | regulation of dendritic cell chemotaxis | 0.049027865 | 0.41924467 | 0.396723157 | TNFSF18 | 1 |
| BP | GO:0006163 | purine nucleotide metabolic process | 0.049035924 | 0.41924467 | 0.396723157 | FHIT/PDHA2/TJP2/GPAM/DLG2 | 5 |
| BP | GO:0051453 | regulation of intracellular pH | 0.049267044 | 0.41924467 | 0.396723157 | FASLG/SLC9C2 | 2 |
| BP | GO:0002695 | negative regulation of leukocyte activation | 0.050162146 | 0.41924467 | 0.396723157 | PLA2G2F/TNFSF18/CNR1 | 3 |
| BP | GO:0007260 | tyrosine phosphorylation of STAT protein | 0.050306611 | 0.41924467 | 0.396723157 | TNFSF18/ISL1 | 2 |
| BP | GO:0032456 | endocytic recycling | 0.050306611 | 0.41924467 | 0.396723157 | STX6/VPS53 | 2 |
| BP | GO:0048638 | regulation of developmental growth | 0.050362685 | 0.41924467 | 0.396723157 | FXN/GPAM/ATP8A2/ATRN | 4 |
| BP | GO:0099175 | regulation of postsynapse organization | 0.051354105 | 0.41924467 | 0.396723157 | DNM3/NEDD9 | 2 |
| BP | GO:2000243 | positive regulation of reproductive process | 0.051354105 | 0.41924467 | 0.396723157 | PLB1/UNC5C | 2 |
| BP | GO:0032370 | positive regulation of lipid transport | 0.052409442 | 0.41924467 | 0.396723157 | FASLG/ATP8A2 | 2 |
| BP | GO:0022408 | negative regulation of cell-cell adhesion | 0.052662505 | 0.41924467 | 0.396723157 | PLA2G2F/TNFSF18/ADAMTS18 | 3 |
| BP | GO:0002863 | positive regulation of inflammatory response to antigenic stimulus | 0.053004721 | 0.41924467 | 0.396723157 | CNR1 | 1 |
| BP | GO:0007501 | mesodermal cell fate specification | 0.053004721 | 0.41924467 | 0.396723157 | EYA1 | 1 |
| BP | GO:0016024 | CDP-diacylglycerol biosynthetic process | 0.053004721 | 0.41924467 | 0.396723157 | GPAM | 1 |
| BP | GO:0033604 | negative regulation of catecholamine secretion | 0.053004721 | 0.41924467 | 0.396723157 | CNR1 | 1 |
| BP | GO:0035865 | cellular response to potassium ion | 0.053004721 | 0.41924467 | 0.396723157 | DLG2 | 1 |
| BP | GO:0038203 | TORC2 signaling | 0.053004721 | 0.41924467 | 0.396723157 | RICTOR | 1 |
| BP | GO:0060100 | positive regulation of phagocytosis, engulfment | 0.053004721 | 0.41924467 | 0.396723157 | PLCG2 | 1 |
| BP | GO:0060831 | smoothened signaling pathway involved in dorsal/ventral neural tube patterning | 0.053004721 | 0.41924467 | 0.396723157 | TBC1D32 | 1 |
| BP | GO:0061000 | negative regulation of dendritic spine development | 0.053004721 | 0.41924467 | 0.396723157 | DNM3 | 1 |
| BP | GO:0097499 | protein localization to non-motile cilium | 0.053004721 | 0.41924467 | 0.396723157 | TULP1 | 1 |
| BP | GO:0099550 | trans-synaptic signaling, modulating synaptic transmission | 0.053004721 | 0.41924467 | 0.396723157 | CNR1 | 1 |
| BP | GO:1905155 | positive regulation of membrane invagination | 0.053004721 | 0.41924467 | 0.396723157 | PLCG2 | 1 |
| BP | GO:0043010 | camera-type eye development | 0.054076816 | 0.41924467 | 0.396723157 | TULP1/TBC1D32/ATP8A2/MAX | 4 |
| BP | GO:0061564 | axon development | 0.054263853 | 0.41924467 | 0.396723157 | ROBO1/UNC5C/ISL1/CNR1/ATP8A2 | 5 |
| BP | GO:0030641 | regulation of cellular pH | 0.05454332 | 0.41924467 | 0.396723157 | FASLG/SLC9C2 | 2 |
| BP | GO:0043281 | regulation of cysteine-type endopeptidase activity involved in apoptotic process | 0.054576847 | 0.41924467 | 0.396723157 | FASLG/ROBO1/ST18 | 3 |
| BP | GO:2000241 | regulation of reproductive process | 0.055222352 | 0.41924467 | 0.396723157 | PLB1/UNC5C/CNR1 | 3 |
| BP | GO:0106027 | neuron projection organization | 0.055621698 | 0.41924467 | 0.396723157 | DNM3/NEDD9 | 2 |
| BP | GO:0033674 | positive regulation of kinase activity | 0.056600583 | 0.41924467 | 0.396723157 | ROBO1/RICTOR/NEDD9/ALKAL1/VAV2 | 5 |
| BP | GO:0001656 | metanephros development | 0.056707596 | 0.41924467 | 0.396723157 | IRX1/EYA1 | 2 |
| BP | GO:0021859 | pyramidal neuron differentiation | 0.056965157 | 0.41924467 | 0.396723157 | DCLK2 | 1 |
| BP | GO:0033004 | negative regulation of mast cell activation | 0.056965157 | 0.41924467 | 0.396723157 | CNR1 | 1 |
| BP | GO:0034111 | negative regulation of homotypic cell-cell adhesion | 0.056965157 | 0.41924467 | 0.396723157 | ADAMTS18 | 1 |
| BP | GO:0046341 | CDP-diacylglycerol metabolic process | 0.056965157 | 0.41924467 | 0.396723157 | GPAM | 1 |
| BP | GO:0048388 | endosomal lumen acidification | 0.056965157 | 0.41924467 | 0.396723157 | FASLG | 1 |
| BP | GO:0072540 | T-helper 17 cell lineage commitment | 0.056965157 | 0.41924467 | 0.396723157 | TNFSF18 | 1 |
| BP | GO:0098734 | macromolecule depalmitoylation | 0.056965157 | 0.41924467 | 0.396723157 | ABHD10 | 1 |
| BP | GO:0099171 | presynaptic modulation of chemical synaptic transmission | 0.056965157 | 0.41924467 | 0.396723157 | CNR1 | 1 |
| BP | GO:1903894 | regulation of IRE1-mediated unfolded protein response | 0.056965157 | 0.41924467 | 0.396723157 | DNAJB9 | 1 |
| BP | GO:0030003 | cellular cation homeostasis | 0.057790604 | 0.41924467 | 0.396723157 | FASLG/SLC9C2/SCGN/FXN/PLCG2 | 5 |
| BP | GO:0035082 | axoneme assembly | 0.057800933 | 0.41924467 | 0.396723157 | SPAG17/DNAH8 | 2 |
| BP | GO:0042147 | retrograde transport, endosome to Golgi | 0.057800933 | 0.41924467 | 0.396723157 | STX6/VPS53 | 2 |
| BP | GO:0006637 | acyl-CoA metabolic process | 0.058901629 | 0.41924467 | 0.396723157 | PDHA2/GPAM | 2 |
| BP | GO:0035383 | thioester metabolic process | 0.058901629 | 0.41924467 | 0.396723157 | PDHA2/GPAM | 2 |
| BP | GO:0042098 | T cell proliferation | 0.059172282 | 0.41924467 | 0.396723157 | PLA2G2F/TNFSF18/GPAM | 3 |
| BP | GO:0099003 | vesicle-mediated transport in synapse | 0.059172282 | 0.41924467 | 0.396723157 | DNM3/CNR1/SH3GL2 | 3 |
| BP | GO:0034109 | homotypic cell-cell adhesion | 0.060009607 | 0.41924467 | 0.396723157 | TJP2/ADAMTS18 | 2 |
| BP | GO:0001325 | formation of extrachromosomal circular DNA | 0.060909238 | 0.41924467 | 0.396723157 | SMARCAL1 | 1 |
| BP | GO:0001771 | immunological synapse formation | 0.060909238 | 0.41924467 | 0.396723157 | NEDD9 | 1 |
| BP | GO:0002864 | regulation of acute inflammatory response to antigenic stimulus | 0.060909238 | 0.41924467 | 0.396723157 | CNR1 | 1 |
| BP | GO:0032725 | positive regulation of granulocyte macrophage colony-stimulating factor production | 0.060909238 | 0.41924467 | 0.396723157 | ISL1 | 1 |
| BP | GO:0033147 | negative regulation of intracellular estrogen receptor signaling pathway | 0.060909238 | 0.41924467 | 0.396723157 | ISL1 | 1 |
| BP | GO:0033599 | regulation of mammary gland epithelial cell proliferation | 0.060909238 | 0.41924467 | 0.396723157 | ROBO1 | 1 |
| BP | GO:0046322 | negative regulation of fatty acid oxidation | 0.060909238 | 0.41924467 | 0.396723157 | CNR1 | 1 |
| BP | GO:0048934 | peripheral nervous system neuron differentiation | 0.060909238 | 0.41924467 | 0.396723157 | ISL1 | 1 |
| BP | GO:0048935 | peripheral nervous system neuron development | 0.060909238 | 0.41924467 | 0.396723157 | ISL1 | 1 |
| BP | GO:0060099 | regulation of phagocytosis, engulfment | 0.060909238 | 0.41924467 | 0.396723157 | PLCG2 | 1 |
| BP | GO:0060413 | atrial septum morphogenesis | 0.060909238 | 0.41924467 | 0.396723157 | ISL1 | 1 |
| BP | GO:0071599 | otic vesicle development | 0.060909238 | 0.41924467 | 0.396723157 | EYA1 | 1 |
| BP | GO:0090656 | t-circle formation | 0.060909238 | 0.41924467 | 0.396723157 | SMARCAL1 | 1 |
| BP | GO:0090737 | telomere maintenance via telomere trimming | 0.060909238 | 0.41924467 | 0.396723157 | SMARCAL1 | 1 |
| BP | GO:0097152 | mesenchymal cell apoptotic process | 0.060909238 | 0.41924467 | 0.396723157 | ETV6 | 1 |
| BP | GO:1900102 | negative regulation of endoplasmic reticulum unfolded protein response | 0.060909238 | 0.41924467 | 0.396723157 | DNAJB9 | 1 |
| BP | GO:1905153 | regulation of membrane invagination | 0.060909238 | 0.41924467 | 0.396723157 | PLCG2 | 1 |
| BP | GO:2000104 | negative regulation of DNA-templated DNA replication | 0.060909238 | 0.41924467 | 0.396723157 | SMARCAL1 | 1 |
| BP | GO:0007032 | endosome organization | 0.061124787 | 0.41924467 | 0.396723157 | FASLG/STX6 | 2 |
| BP | GO:0061097 | regulation of protein tyrosine kinase activity | 0.061124787 | 0.41924467 | 0.396723157 | NEDD9/ALKAL1 | 2 |
| BP | GO:0072080 | nephron tubule development | 0.061124787 | 0.41924467 | 0.396723157 | IRX1/EYA1 | 2 |
| BP | GO:0002690 | positive regulation of leukocyte chemotaxis | 0.062247092 | 0.41924467 | 0.396723157 | TNFSF18/NEDD9 | 2 |
| BP | GO:0060993 | kidney morphogenesis | 0.062247092 | 0.41924467 | 0.396723157 | IRX1/EYA1 | 2 |
| BP | GO:1902414 | protein localization to cell junction | 0.063376445 | 0.41924467 | 0.396723157 | TJP2/DLG2 | 2 |
| BP | GO:0003002 | regionalization | 0.063998183 | 0.41924467 | 0.396723157 | ROBO1/IRX1/ISL1/TBC1D32 | 4 |
| BP | GO:0006885 | regulation of pH | 0.064512768 | 0.41924467 | 0.396723157 | FASLG/SLC9C2 | 2 |
| BP | GO:0032755 | positive regulation of interleukin-6 production | 0.064512768 | 0.41924467 | 0.396723157 | ISL1/PLCG2 | 2 |
| BP | GO:0061326 | renal tubule development | 0.064512768 | 0.41924467 | 0.396723157 | IRX1/EYA1 | 2 |
| BP | GO:0007601 | visual perception | 0.064640972 | 0.41924467 | 0.396723157 | TULP1/ATP8A2/IRX5 | 3 |
| BP | GO:0002730 | regulation of dendritic cell cytokine production | 0.064837032 | 0.41924467 | 0.396723157 | PLCG2 | 1 |
| BP | GO:0007028 | cytoplasm organization | 0.064837032 | 0.41924467 | 0.396723157 | ETV6 | 1 |
| BP | GO:0034638 | phosphatidylcholine catabolic process | 0.064837032 | 0.41924467 | 0.396723157 | PLB1 | 1 |
| BP | GO:0042159 | lipoprotein catabolic process | 0.064837032 | 0.41924467 | 0.396723157 | ABHD10 | 1 |
| BP | GO:0055091 | phospholipid homeostasis | 0.064837032 | 0.41924467 | 0.396723157 | GPAM | 1 |
| BP | GO:0060046 | regulation of acrosome reaction | 0.064837032 | 0.41924467 | 0.396723157 | PLB1 | 1 |
| BP | GO:0060911 | cardiac cell fate commitment | 0.064837032 | 0.41924467 | 0.396723157 | ISL1 | 1 |
| BP | GO:0050953 | sensory perception of light stimulus | 0.067460354 | 0.41924467 | 0.396723157 | TULP1/ATP8A2/IRX5 | 3 |
| BP | GO:0006486 | protein glycosylation | 0.068173934 | 0.41924467 | 0.396723157 | ALG6/XXYLT1/ST8SIA1 | 3 |
| BP | GO:0043413 | macromolecule glycosylation | 0.068173934 | 0.41924467 | 0.396723157 | ALG6/XXYLT1/ST8SIA1 | 3 |
| BP | GO:0002371 | dendritic cell cytokine production | 0.068748605 | 0.41924467 | 0.396723157 | PLCG2 | 1 |
| BP | GO:0003184 | pulmonary valve morphogenesis | 0.068748605 | 0.41924467 | 0.396723157 | ROBO1 | 1 |
| BP | GO:0007379 | segment specification | 0.068748605 | 0.41924467 | 0.396723157 | IRX1 | 1 |
| BP | GO:0031649 | heat generation | 0.068748605 | 0.41924467 | 0.396723157 | CNR1 | 1 |
| BP | GO:0055089 | fatty acid homeostasis | 0.068748605 | 0.41924467 | 0.396723157 | GPAM | 1 |
| BP | GO:0061548 | ganglion development | 0.068748605 | 0.41924467 | 0.396723157 | UNC5C | 1 |
| BP | GO:1903729 | regulation of plasma membrane organization | 0.068748605 | 0.41924467 | 0.396723157 | FASLG | 1 |
| BP | GO:2001267 | regulation of cysteine-type endopeptidase activity involved in apoptotic signaling pathway | 0.068748605 | 0.41924467 | 0.396723157 | ST18 | 1 |
| BP | GO:0042472 | inner ear morphogenesis | 0.069126252 | 0.41924467 | 0.396723157 | EYA1/ATP8A2 | 2 |
| BP | GO:0050764 | regulation of phagocytosis | 0.069126252 | 0.41924467 | 0.396723157 | TULP1/PLCG2 | 2 |
| BP | GO:0006641 | triglyceride metabolic process | 0.070296296 | 0.41924467 | 0.396723157 | PLB1/GPAM | 2 |
| BP | GO:0009135 | purine nucleoside diphosphate metabolic process | 0.070296296 | 0.41924467 | 0.396723157 | TJP2/DLG2 | 2 |
| BP | GO:0009179 | purine ribonucleoside diphosphate metabolic process | 0.070296296 | 0.41924467 | 0.396723157 | TJP2/DLG2 | 2 |
| BP | GO:0015914 | phospholipid transport | 0.070296296 | 0.41924467 | 0.396723157 | FASLG/ATP8A2 | 2 |
| BP | GO:0032760 | positive regulation of tumor necrosis factor production | 0.07147286 | 0.41924467 | 0.396723157 | ISL1/PLCG2 | 2 |
| BP | GO:0050920 | regulation of chemotaxis | 0.071793649 | 0.41924467 | 0.396723157 | TNFSF18/ROBO1/NEDD9 | 3 |
| BP | GO:0043087 | regulation of GTPase activity | 0.072580397 | 0.41924467 | 0.396723157 | RICTOR/NEDD9/VAV2/RGS6 | 4 |
| BP | GO:0001710 | mesodermal cell fate commitment | 0.072644023 | 0.41924467 | 0.396723157 | EYA1 | 1 |
| BP | GO:0001956 | positive regulation of neurotransmitter secretion | 0.072644023 | 0.41924467 | 0.396723157 | CNR1 | 1 |
| BP | GO:0002295 | T-helper cell lineage commitment | 0.072644023 | 0.41924467 | 0.396723157 | TNFSF18 | 1 |
| BP | GO:0006085 | acetyl-CoA biosynthetic process | 0.072644023 | 0.41924467 | 0.396723157 | PDHA2 | 1 |
| BP | GO:0006488 | dolichol-linked oligosaccharide biosynthetic process | 0.072644023 | 0.41924467 | 0.396723157 | ALG6 | 1 |
| BP | GO:0032604 | granulocyte macrophage colony-stimulating factor production | 0.072644023 | 0.41924467 | 0.396723157 | ISL1 | 1 |
| BP | GO:0032645 | regulation of granulocyte macrophage colony-stimulating factor production | 0.072644023 | 0.41924467 | 0.396723157 | ISL1 | 1 |
| BP | GO:0032769 | negative regulation of monooxygenase activity | 0.072644023 | 0.41924467 | 0.396723157 | CNR1 | 1 |
| BP | GO:0035234 | ectopic germ cell programmed cell death | 0.072644023 | 0.41924467 | 0.396723157 | UNC5C | 1 |
| BP | GO:0035864 | response to potassium ion | 0.072644023 | 0.41924467 | 0.396723157 | DLG2 | 1 |
| BP | GO:0043116 | negative regulation of vascular permeability | 0.072644023 | 0.41924467 | 0.396723157 | SH3GL2 | 1 |
| BP | GO:0043117 | positive regulation of vascular permeability | 0.072644023 | 0.41924467 | 0.396723157 | TJP2 | 1 |
| BP | GO:0045623 | negative regulation of T-helper cell differentiation | 0.072644023 | 0.41924467 | 0.396723157 | TNFSF18 | 1 |
| BP | GO:0097094 | craniofacial suture morphogenesis | 0.072644023 | 0.41924467 | 0.396723157 | FOXN3 | 1 |
| BP | GO:0150078 | positive regulation of neuroinflammatory response | 0.072644023 | 0.41924467 | 0.396723157 | PLCG2 | 1 |
| BP | GO:0003279 | cardiac septum development | 0.073845257 | 0.41924467 | 0.396723157 | ROBO1/ISL1 | 2 |
| BP | GO:0060840 | artery development | 0.073845257 | 0.41924467 | 0.396723157 | ROBO1/EYA1 | 2 |
| BP | GO:0007631 | feeding behavior | 0.075040946 | 0.41924467 | 0.396723157 | CNR1/ATP8A2 | 2 |
| BP | GO:0032006 | regulation of TOR signaling | 0.075040946 | 0.41924467 | 0.396723157 | RICTOR/GNA12 | 2 |
| BP | GO:0051341 | regulation of oxidoreductase activity | 0.075040946 | 0.41924467 | 0.396723157 | CNR1/FXN | 2 |
| BP | GO:2000116 | regulation of cysteine-type endopeptidase activity | 0.075498613 | 0.41924467 | 0.396723157 | FASLG/ROBO1/ST18 | 3 |
| BP | GO:0033209 | tumor necrosis factor-mediated signaling pathway | 0.076242864 | 0.41924467 | 0.396723157 | TNFSF18/ST18 | 2 |
| BP | GO:1903557 | positive regulation of tumor necrosis factor superfamily cytokine production | 0.076242864 | 0.41924467 | 0.396723157 | ISL1/PLCG2 | 2 |
| BP | GO:0007411 | axon guidance | 0.0762497 | 0.41924467 | 0.396723157 | ROBO1/UNC5C/ISL1 | 3 |
| BP | GO:0002281 | macrophage activation involved in immune response | 0.076523351 | 0.41924467 | 0.396723157 | PLCG2 | 1 |
| BP | GO:0006490 | oligosaccharide-lipid intermediate biosynthetic process | 0.076523351 | 0.41924467 | 0.396723157 | ALG6 | 1 |
| BP | GO:0006837 | serotonin transport | 0.076523351 | 0.41924467 | 0.396723157 | CNR1 | 1 |
| BP | GO:0010544 | negative regulation of platelet activation | 0.076523351 | 0.41924467 | 0.396723157 | ADAMTS18 | 1 |
| BP | GO:0010759 | positive regulation of macrophage chemotaxis | 0.076523351 | 0.41924467 | 0.396723157 | TNFSF18 | 1 |
| BP | GO:0098884 | postsynaptic neurotransmitter receptor internalization | 0.076523351 | 0.41924467 | 0.396723157 | DNM3 | 1 |
| BP | GO:0140239 | postsynaptic endocytosis | 0.076523351 | 0.41924467 | 0.396723157 | DNM3 | 1 |
| BP | GO:0097485 | neuron projection guidance | 0.077004122 | 0.41924467 | 0.396723157 | ROBO1/UNC5C/ISL1 | 3 |
| BP | GO:0007173 | epidermal growth factor receptor signaling pathway | 0.077450942 | 0.41924467 | 0.396723157 | FASLG/FAM83B | 2 |
| BP | GO:0050670 | regulation of lymphocyte proliferation | 0.078522923 | 0.41924467 | 0.396723157 | PLA2G2F/TNFSF18/GPAM | 3 |
| BP | GO:0031532 | actin cytoskeleton reorganization | 0.078665108 | 0.41924467 | 0.396723157 | RICTOR/NEDD9 | 2 |
| BP | GO:0034446 | substrate adhesion-dependent cell spreading | 0.078665108 | 0.41924467 | 0.396723157 | NEDD9/ATRN | 2 |
| BP | GO:0003215 | cardiac right ventricle morphogenesis | 0.080386657 | 0.41924467 | 0.396723157 | ISL1 | 1 |
| BP | GO:0006491 | N-glycan processing | 0.080386657 | 0.41924467 | 0.396723157 | ST8SIA1 | 1 |
| BP | GO:0030002 | cellular anion homeostasis | 0.080386657 | 0.41924467 | 0.396723157 | FASLG | 1 |
| BP | GO:0031998 | regulation of fatty acid beta-oxidation | 0.080386657 | 0.41924467 | 0.396723157 | CNR1 | 1 |
| BP | GO:0036498 | IRE1-mediated unfolded protein response | 0.080386657 | 0.41924467 | 0.396723157 | DNAJB9 | 1 |
| BP | GO:0042474 | middle ear morphogenesis | 0.080386657 | 0.41924467 | 0.396723157 | EYA1 | 1 |
| BP | GO:0055064 | chloride ion homeostasis | 0.080386657 | 0.41924467 | 0.396723157 | FASLG | 1 |
| BP | GO:0055083 | monovalent inorganic anion homeostasis | 0.080386657 | 0.41924467 | 0.396723157 | FASLG | 1 |
| BP | GO:0072079 | nephron tubule formation | 0.080386657 | 0.41924467 | 0.396723157 | IRX1 | 1 |
| BP | GO:1900225 | regulation of NLRP3 inflammasome complex assembly | 0.080386657 | 0.41924467 | 0.396723157 | PLCG2 | 1 |
| BP | GO:1903421 | regulation of synaptic vesicle recycling | 0.080386657 | 0.41924467 | 0.396723157 | DNM3 | 1 |
| BP | GO:0050878 | regulation of body fluid levels | 0.08055021 | 0.41924467 | 0.396723157 | GNA12/VAV2/ADAMTS18/PLCG2 | 4 |
| BP | GO:0007229 | integrin-mediated signaling pathway | 0.081111422 | 0.41924467 | 0.396723157 | FYB1/NEDD9 | 2 |
| BP | GO:1905954 | positive regulation of lipid localization | 0.081111422 | 0.41924467 | 0.396723157 | FASLG/ATP8A2 | 2 |
| BP | GO:0044782 | cilium organization | 0.081135657 | 0.41924467 | 0.396723157 | SPAG17/NEDD9/DNAH8/TBC1D32 | 4 |
| BP | GO:0032944 | regulation of mononuclear cell proliferation | 0.081599987 | 0.41924467 | 0.396723157 | PLA2G2F/TNFSF18/GPAM | 3 |
| BP | GO:0009185 | ribonucleoside diphosphate metabolic process | 0.082343431 | 0.41924467 | 0.396723157 | TJP2/DLG2 | 2 |
| BP | GO:0030004 | cellular monovalent inorganic cation homeostasis | 0.082343431 | 0.41924467 | 0.396723157 | FASLG/SLC9C2 | 2 |
| BP | GO:0070085 | glycosylation | 0.082377399 | 0.41924467 | 0.396723157 | ALG6/XXYLT1/ST8SIA1 | 3 |
| BP | GO:0003177 | pulmonary valve development | 0.084234004 | 0.41924467 | 0.396723157 | ROBO1 | 1 |
| BP | GO:0007063 | regulation of sister chromatid cohesion | 0.084234004 | 0.41924467 | 0.396723157 | SLF2 | 1 |
| BP | GO:0007252 | I-kappaB phosphorylation | 0.084234004 | 0.41924467 | 0.396723157 | PLCG2 | 1 |
| BP | GO:0007413 | axonal fasciculation | 0.084234004 | 0.41924467 | 0.396723157 | CNR1 | 1 |
| BP | GO:0008090 | retrograde axonal transport | 0.084234004 | 0.41924467 | 0.396723157 | DLG2 | 1 |
| BP | GO:0009083 | branched-chain amino acid catabolic process | 0.084234004 | 0.41924467 | 0.396723157 | BCKDHB | 1 |
| BP | GO:0010002 | cardioblast differentiation | 0.084234004 | 0.41924467 | 0.396723157 | ISL1 | 1 |
| BP | GO:0016137 | glycoside metabolic process | 0.084234004 | 0.41924467 | 0.396723157 | ABHD10 | 1 |
| BP | GO:0043373 | CD4-positive, alpha-beta T cell lineage commitment | 0.084234004 | 0.41924467 | 0.396723157 | TNFSF18 | 1 |
| BP | GO:0052695 | cellular glucuronidation | 0.084234004 | 0.41924467 | 0.396723157 | ABHD10 | 1 |
| BP | GO:0090026 | positive regulation of monocyte chemotaxis | 0.084234004 | 0.41924467 | 0.396723157 | TNFSF18 | 1 |
| BP | GO:0090201 | negative regulation of release of cytochrome c from mitochondria | 0.084234004 | 0.41924467 | 0.396723157 | FXN | 1 |
| BP | GO:0097062 | dendritic spine maintenance | 0.084234004 | 0.41924467 | 0.396723157 | NEDD9 | 1 |
| BP | GO:0097503 | sialylation | 0.084234004 | 0.41924467 | 0.396723157 | ST8SIA1 | 1 |
| BP | GO:0106030 | neuron projection fasciculation | 0.084234004 | 0.41924467 | 0.396723157 | CNR1 | 1 |
| BP | GO:0140131 | positive regulation of lymphocyte chemotaxis | 0.084234004 | 0.41924467 | 0.396723157 | NEDD9 | 1 |
| BP | GO:0150105 | protein localization to cell-cell junction | 0.084234004 | 0.41924467 | 0.396723157 | TJP2 | 1 |
| BP | GO:2000319 | regulation of T-helper 17 cell differentiation | 0.084234004 | 0.41924467 | 0.396723157 | TNFSF18 | 1 |
| BP | GO:0072009 | nephron epithelium development | 0.084824807 | 0.420170543 | 0.397599293 | IRX1/EYA1 | 2 |
| BP | GO:0071887 | leukocyte apoptotic process | 0.086074038 | 0.420170543 | 0.397599293 | FASLG/GPAM | 2 |
| BP | GO:0030326 | embryonic limb morphogenesis | 0.087328874 | 0.420170543 | 0.397599293 | TBC1D32/GNA12 | 2 |
| BP | GO:0035113 | embryonic appendage morphogenesis | 0.087328874 | 0.420170543 | 0.397599293 | TBC1D32/GNA12 | 2 |
| BP | GO:0002223 | stimulatory C-type lectin receptor signaling pathway | 0.088065458 | 0.420170543 | 0.397599293 | PLCG2 | 1 |
| BP | GO:0003228 | atrial cardiac muscle tissue development | 0.088065458 | 0.420170543 | 0.397599293 | ISL1 | 1 |
| BP | GO:0007620 | copulation | 0.088065458 | 0.420170543 | 0.397599293 | CNR1 | 1 |
| BP | GO:0031290 | retinal ganglion cell axon guidance | 0.088065458 | 0.420170543 | 0.397599293 | ISL1 | 1 |
| BP | GO:0032516 | positive regulation of phosphoprotein phosphatase activity | 0.088065458 | 0.420170543 | 0.397599293 | GNA12 | 1 |
| BP | GO:0043371 | negative regulation of CD4-positive, alpha-beta T cell differentiation | 0.088065458 | 0.420170543 | 0.397599293 | TNFSF18 | 1 |
| BP | GO:0044546 | NLRP3 inflammasome complex assembly | 0.088065458 | 0.420170543 | 0.397599293 | PLCG2 | 1 |
| BP | GO:1903428 | positive regulation of reactive oxygen species biosynthetic process | 0.088065458 | 0.420170543 | 0.397599293 | PLCG2 | 1 |
| BP | GO:1990840 | response to lectin | 0.088065458 | 0.420170543 | 0.397599293 | PLCG2 | 1 |
| BP | GO:1990858 | cellular response to lectin | 0.088065458 | 0.420170543 | 0.397599293 | PLCG2 | 1 |
| BP | GO:0031623 | receptor internalization | 0.09112634 | 0.420170543 | 0.397599293 | DNM3/PLCG2 | 2 |
| BP | GO:0051402 | neuron apoptotic process | 0.091139334 | 0.420170543 | 0.397599293 | FASLG/ISL1/MAX | 3 |
| BP | GO:0002363 | alpha-beta T cell lineage commitment | 0.091881083 | 0.420170543 | 0.397599293 | TNFSF18 | 1 |
| BP | GO:0003283 | atrial septum development | 0.091881083 | 0.420170543 | 0.397599293 | ISL1 | 1 |
| BP | GO:0021904 | dorsal/ventral neural tube patterning | 0.091881083 | 0.420170543 | 0.397599293 | TBC1D32 | 1 |
| BP | GO:0036158 | outer dynein arm assembly | 0.091881083 | 0.420170543 | 0.397599293 | DNAH8 | 1 |
| BP | GO:0061760 | antifungal innate immune response | 0.091881083 | 0.420170543 | 0.397599293 | PLCG2 | 1 |
| BP | GO:0014706 | striated muscle tissue development | 0.09277304 | 0.420170543 | 0.397599293 | ISL1/EYA1/SGCG | 3 |
| BP | GO:0003007 | heart morphogenesis | 0.093594485 | 0.420170543 | 0.397599293 | ROBO1/ISL1/EYA1 | 3 |
| BP | GO:0071675 | regulation of mononuclear cell migration | 0.093684788 | 0.420170543 | 0.397599293 | TNFSF18/NEDD9 | 2 |
| BP | GO:0001578 | microtubule bundle formation | 0.094971857 | 0.420170543 | 0.397599293 | SPAG17/DNAH8 | 2 |
| BP | GO:0042471 | ear morphogenesis | 0.094971857 | 0.420170543 | 0.397599293 | EYA1/ATP8A2 | 2 |
| BP | GO:0051209 | release of sequestered calcium ion into cytosol | 0.094971857 | 0.420170543 | 0.397599293 | FASLG/PLCG2 | 2 |
| BP | GO:0050678 | regulation of epithelial cell proliferation | 0.095182155 | 0.420170543 | 0.397599293 | ROBO1/ISL1/EYA1/ST8SIA1 | 4 |
| BP | GO:0060828 | regulation of canonical Wnt signaling pathway | 0.09524649 | 0.420170543 | 0.397599293 | ISL1/TMEM170B/GPC5 | 3 |
| BP | GO:0001759 | organ induction | 0.095680945 | 0.420170543 | 0.397599293 | ROBO1 | 1 |
| BP | GO:0006925 | inflammatory cell apoptotic process | 0.095680945 | 0.420170543 | 0.397599293 | FASLG | 1 |
| BP | GO:0021884 | forebrain neuron development | 0.095680945 | 0.420170543 | 0.397599293 | DCLK2 | 1 |
| BP | GO:0043369 | CD4-positive or CD8-positive, alpha-beta T cell lineage commitment | 0.095680945 | 0.420170543 | 0.397599293 | TNFSF18 | 1 |
| BP | GO:0051590 | positive regulation of neurotransmitter transport | 0.095680945 | 0.420170543 | 0.397599293 | CNR1 | 1 |
| BP | GO:0060907 | positive regulation of macrophage cytokine production | 0.095680945 | 0.420170543 | 0.397599293 | PLCG2 | 1 |
| BP | GO:0070233 | negative regulation of T cell apoptotic process | 0.095680945 | 0.420170543 | 0.397599293 | GPAM | 1 |
| BP | GO:0090103 | cochlea morphogenesis | 0.095680945 | 0.420170543 | 0.397599293 | EYA1 | 1 |
| BP | GO:0140632 | inflammasome complex assembly | 0.095680945 | 0.420170543 | 0.397599293 | PLCG2 | 1 |
| BP | GO:2000353 | positive regulation of endothelial cell apoptotic process | 0.095680945 | 0.420170543 | 0.397599293 | FASLG | 1 |
| BP | GO:0051283 | negative regulation of sequestering of calcium ion | 0.096264069 | 0.420170543 | 0.397599293 | FASLG/PLCG2 | 2 |
| BP | GO:0003206 | cardiac chamber morphogenesis | 0.097561361 | 0.420170543 | 0.397599293 | ROBO1/ISL1 | 2 |
| BP | GO:0003231 | cardiac ventricle development | 0.097561361 | 0.420170543 | 0.397599293 | ROBO1/ISL1 | 2 |
| BP | GO:0033865 | nucleoside bisphosphate metabolic process | 0.097561361 | 0.420170543 | 0.397599293 | PDHA2/GPAM | 2 |
| BP | GO:0033875 | ribonucleoside bisphosphate metabolic process | 0.097561361 | 0.420170543 | 0.397599293 | PDHA2/GPAM | 2 |
| BP | GO:0034032 | purine nucleoside bisphosphate metabolic process | 0.097561361 | 0.420170543 | 0.397599293 | PDHA2/GPAM | 2 |
| BP | GO:0038127 | ERBB signaling pathway | 0.097561361 | 0.420170543 | 0.397599293 | FASLG/FAM83B | 2 |
| BP | GO:0002688 | regulation of leukocyte chemotaxis | 0.098863668 | 0.420170543 | 0.397599293 | TNFSF18/NEDD9 | 2 |
| BP | GO:0051282 | regulation of sequestering of calcium ion | 0.098863668 | 0.420170543 | 0.397599293 | FASLG/PLCG2 | 2 |
| BP | GO:0006875 | cellular metal ion homeostasis | 0.099025813 | 0.420170543 | 0.397599293 | FASLG/SCGN/FXN/PLCG2 | 4 |
| BP | GO:0045860 | positive regulation of protein kinase activity | 0.099025813 | 0.420170543 | 0.397599293 | ROBO1/RICTOR/NEDD9/ALKAL1 | 4 |
| BP | GO:0006658 | phosphatidylserine metabolic process | 0.099465108 | 0.420170543 | 0.397599293 | PLA2G2F | 1 |
| BP | GO:0017121 | plasma membrane phospholipid scrambling | 0.099465108 | 0.420170543 | 0.397599293 | FASLG | 1 |
| BP | GO:0046716 | muscle cell cellular homeostasis | 0.099465108 | 0.420170543 | 0.397599293 | FXN | 1 |
| BP | GO:0098719 | sodium ion import across plasma membrane | 0.099465108 | 0.420170543 | 0.397599293 | SLC9C2 | 1 |
| BP | GO:2000114 | regulation of establishment of cell polarity | 0.099465108 | 0.420170543 | 0.397599293 | RICTOR | 1 |
| BP | GO:0045739 | positive regulation of DNA repair | 0.100170929 | 0.420170543 | 0.397599293 | EYA1/SLF2 | 2 |
| BP | GO:0050868 | negative regulation of T cell activation | 0.100170929 | 0.420170543 | 0.397599293 | PLA2G2F/TNFSF18 | 2 |
| BP | GO:0031929 | TOR signaling | 0.102800057 | 0.420170543 | 0.397599293 | RICTOR/GNA12 | 2 |
| BP | GO:0070663 | regulation of leukocyte proliferation | 0.102828149 | 0.420170543 | 0.397599293 | PLA2G2F/TNFSF18/GPAM | 3 |
| BP | GO:0002433 | immune response-regulating cell surface receptor signaling pathway involved in phagocytosis | 0.103233635 | 0.420170543 | 0.397599293 | VAV2 | 1 |
| BP | GO:0002438 | acute inflammatory response to antigenic stimulus | 0.103233635 | 0.420170543 | 0.397599293 | CNR1 | 1 |
| BP | GO:0006063 | uronic acid metabolic process | 0.103233635 | 0.420170543 | 0.397599293 | ABHD10 | 1 |
| BP | GO:0006929 | substrate-dependent cell migration | 0.103233635 | 0.420170543 | 0.397599293 | ROBO1 | 1 |
| BP | GO:0007064 | mitotic sister chromatid cohesion | 0.103233635 | 0.420170543 | 0.397599293 | SLF2 | 1 |
| BP | GO:0010259 | multicellular organism aging | 0.103233635 | 0.420170543 | 0.397599293 | GNA12 | 1 |
| BP | GO:0010842 | retina layer formation | 0.103233635 | 0.420170543 | 0.397599293 | ATP8A2 | 1 |
| BP | GO:0019585 | glucuronate metabolic process | 0.103233635 | 0.420170543 | 0.397599293 | ABHD10 | 1 |
| BP | GO:0033598 | mammary gland epithelial cell proliferation | 0.103233635 | 0.420170543 | 0.397599293 | ROBO1 | 1 |
| BP | GO:0038096 | Fc-gamma receptor signaling pathway involved in phagocytosis | 0.103233635 | 0.420170543 | 0.397599293 | VAV2 | 1 |
| BP | GO:0045672 | positive regulation of osteoclast differentiation | 0.103233635 | 0.420170543 | 0.397599293 | NEDD9 | 1 |
| BP | GO:0046639 | negative regulation of alpha-beta T cell differentiation | 0.103233635 | 0.420170543 | 0.397599293 | TNFSF18 | 1 |
| BP | GO:0048026 | positive regulation of mRNA splicing, via spliceosome | 0.103233635 | 0.420170543 | 0.397599293 | PRDX6 | 1 |
| BP | GO:0050995 | negative regulation of lipid catabolic process | 0.103233635 | 0.420170543 | 0.397599293 | CNR1 | 1 |
| BP | GO:0060384 | innervation | 0.103233635 | 0.420170543 | 0.397599293 | ISL1 | 1 |
| BP | GO:0070102 | interleukin-6-mediated signaling pathway | 0.103233635 | 0.420170543 | 0.397599293 | ST18 | 1 |
| BP | GO:0099590 | neurotransmitter receptor internalization | 0.103233635 | 0.420170543 | 0.397599293 | DNM3 | 1 |
| BP | GO:1904753 | negative regulation of vascular associated smooth muscle cell migration | 0.103233635 | 0.420170543 | 0.397599293 | GNA12 | 1 |
| BP | GO:1905523 | positive regulation of macrophage migration | 0.103233635 | 0.420170543 | 0.397599293 | TNFSF18 | 1 |
| BP | GO:0051208 | sequestering of calcium ion | 0.1041218 | 0.421653034 | 0.399002146 | FASLG/PLCG2 | 2 |
| BP | GO:0052548 | regulation of endopeptidase activity | 0.104262844 | 0.421653034 | 0.399002146 | FASLG/ROBO1/ST18/ITIH2 | 4 |
| BP | GO:0006639 | acylglycerol metabolic process | 0.105448247 | 0.421653034 | 0.399002146 | PLB1/GPAM | 2 |
| BP | GO:0009132 | nucleoside diphosphate metabolic process | 0.105448247 | 0.421653034 | 0.399002146 | TJP2/DLG2 | 2 |
| BP | GO:0002702 | positive regulation of production of molecular mediator of immune response | 0.106779337 | 0.421653034 | 0.399002146 | DNAJB9/PLCG2 | 2 |
| BP | GO:0006638 | neutral lipid metabolic process | 0.106779337 | 0.421653034 | 0.399002146 | PLB1/GPAM | 2 |
| BP | GO:0002092 | positive regulation of receptor internalization | 0.106986591 | 0.421653034 | 0.399002146 | PLCG2 | 1 |
| BP | GO:0002407 | dendritic cell chemotaxis | 0.106986591 | 0.421653034 | 0.399002146 | TNFSF18 | 1 |
| BP | GO:0009081 | branched-chain amino acid metabolic process | 0.106986591 | 0.421653034 | 0.399002146 | BCKDHB | 1 |
| BP | GO:0016540 | protein autoprocessing | 0.106986591 | 0.421653034 | 0.399002146 | FXN | 1 |
| BP | GO:0051953 | negative regulation of amine transport | 0.106986591 | 0.421653034 | 0.399002146 | CNR1 | 1 |
| BP | GO:0060259 | regulation of feeding behavior | 0.106986591 | 0.421653034 | 0.399002146 | CNR1 | 1 |
| BP | GO:1900746 | regulation of vascular endothelial growth factor signaling pathway | 0.106986591 | 0.421653034 | 0.399002146 | ROBO1 | 1 |
| BP | GO:1901623 | regulation of lymphocyte chemotaxis | 0.106986591 | 0.421653034 | 0.399002146 | NEDD9 | 1 |
| BP | GO:0001894 | tissue homeostasis | 0.108013197 | 0.424738126 | 0.401921509 | TULP1/SH3GL2/TJP2 | 3 |
| BP | GO:0002675 | positive regulation of acute inflammatory response | 0.110724038 | 0.425645131 | 0.40277979 | CNR1 | 1 |
| BP | GO:0003272 | endocardial cushion formation | 0.110724038 | 0.425645131 | 0.40277979 | ROBO1 | 1 |
| BP | GO:0010758 | regulation of macrophage chemotaxis | 0.110724038 | 0.425645131 | 0.40277979 | TNFSF18 | 1 |
| BP | GO:0010996 | response to auditory stimulus | 0.110724038 | 0.425645131 | 0.40277979 | ATP8A2 | 1 |
| BP | GO:0014072 | response to isoquinoline alkaloid | 0.110724038 | 0.425645131 | 0.40277979 | CNR1 | 1 |
| BP | GO:0032878 | regulation of establishment or maintenance of cell polarity | 0.110724038 | 0.425645131 | 0.40277979 | RICTOR | 1 |
| BP | GO:0032958 | inositol phosphate biosynthetic process | 0.110724038 | 0.425645131 | 0.40277979 | PLCG2 | 1 |
| BP | GO:0043278 | response to morphine | 0.110724038 | 0.425645131 | 0.40277979 | CNR1 | 1 |
| BP | GO:0046337 | phosphatidylethanolamine metabolic process | 0.110724038 | 0.425645131 | 0.40277979 | PLB1 | 1 |
| BP | GO:0007409 | axonogenesis | 0.110985672 | 0.425645131 | 0.40277979 | ROBO1/UNC5C/ISL1/ATP8A2 | 4 |
| BP | GO:0009581 | detection of external stimulus | 0.112148917 | 0.425645131 | 0.40277979 | TULP1/ATP8A2 | 2 |
| BP | GO:0042060 | wound healing | 0.113728626 | 0.425645131 | 0.40277979 | GNA12/VAV2/ADAMTS18/PLCG2 | 4 |
| BP | GO:0003156 | regulation of animal organ formation | 0.114446041 | 0.425645131 | 0.40277979 | ROBO1 | 1 |
| BP | GO:0003209 | cardiac atrium morphogenesis | 0.114446041 | 0.425645131 | 0.40277979 | ISL1 | 1 |
| BP | GO:0010575 | positive regulation of vascular endothelial growth factor production | 0.114446041 | 0.425645131 | 0.40277979 | ISL1 | 1 |
| BP | GO:0042104 | positive regulation of activated T cell proliferation | 0.114446041 | 0.425645131 | 0.40277979 | GPAM | 1 |
| BP | GO:0090025 | regulation of monocyte chemotaxis | 0.114446041 | 0.425645131 | 0.40277979 | TNFSF18 | 1 |
| BP | GO:1902547 | regulation of cellular response to vascular endothelial growth factor stimulus | 0.114446041 | 0.425645131 | 0.40277979 | ROBO1 | 1 |
| BP | GO:0050852 | T cell receptor signaling pathway | 0.114860005 | 0.425645131 | 0.40277979 | FYB1/PLCG2 | 2 |
| BP | GO:0006874 | cellular calcium ion homeostasis | 0.115978934 | 0.425645131 | 0.40277979 | FASLG/SCGN/PLCG2 | 3 |
| BP | GO:0007266 | Rho protein signal transduction | 0.116221919 | 0.425645131 | 0.40277979 | ROBO1/GNA12 | 2 |
| BP | GO:0009582 | detection of abiotic stimulus | 0.116221919 | 0.425645131 | 0.40277979 | TULP1/ATP8A2 | 2 |
| BP | GO:0030833 | regulation of actin filament polymerization | 0.116221919 | 0.425645131 | 0.40277979 | RICTOR/ARPC1B | 2 |
| BP | GO:0009150 | purine ribonucleotide metabolic process | 0.117199779 | 0.425645131 | 0.40277979 | PDHA2/TJP2/GPAM/DLG2 | 4 |
| BP | GO:0032434 | regulation of proteasomal ubiquitin-dependent protein catabolic process | 0.117588 | 0.425645131 | 0.40277979 | FHIT/GNA12 | 2 |
| BP | GO:0090090 | negative regulation of canonical Wnt signaling pathway | 0.117588 | 0.425645131 | 0.40277979 | ISL1/TMEM170B | 2 |
| BP | GO:0002360 | T cell lineage commitment | 0.118152662 | 0.425645131 | 0.40277979 | TNFSF18 | 1 |
| BP | GO:0016226 | iron-sulfur cluster assembly | 0.118152662 | 0.425645131 | 0.40277979 | FXN | 1 |
| BP | GO:0021522 | spinal cord motor neuron differentiation | 0.118152662 | 0.425645131 | 0.40277979 | ISL1 | 1 |
| BP | GO:0031163 | metallo-sulfur cluster assembly | 0.118152662 | 0.425645131 | 0.40277979 | FXN | 1 |
| BP | GO:0070498 | interleukin-1-mediated signaling pathway | 0.118152662 | 0.425645131 | 0.40277979 | ST18 | 1 |
| BP | GO:0072539 | T-helper 17 cell differentiation | 0.118152662 | 0.425645131 | 0.40277979 | TNFSF18 | 1 |
| BP | GO:0090330 | regulation of platelet aggregation | 0.118152662 | 0.425645131 | 0.40277979 | ADAMTS18 | 1 |
| BP | GO:1900101 | regulation of endoplasmic reticulum unfolded protein response | 0.118152662 | 0.425645131 | 0.40277979 | DNAJB9 | 1 |
| BP | GO:0002683 | negative regulation of immune system process | 0.118601303 | 0.425645131 | 0.40277979 | PLA2G2F/TNFSF18/CNR1/GPAM | 4 |
| BP | GO:0048568 | embryonic organ development | 0.118601303 | 0.425645131 | 0.40277979 | TBX15/EYA1/ATP8A2/IRX5 | 4 |
| BP | GO:0035107 | appendage morphogenesis | 0.118958193 | 0.425645131 | 0.40277979 | TBC1D32/GNA12 | 2 |
| BP | GO:0035108 | limb morphogenesis | 0.118958193 | 0.425645131 | 0.40277979 | TBC1D32/GNA12 | 2 |
| BP | GO:0051384 | response to glucocorticoid | 0.118958193 | 0.425645131 | 0.40277979 | ISL1/BCKDHB | 2 |
| BP | GO:0050921 | positive regulation of chemotaxis | 0.121710685 | 0.425645131 | 0.40277979 | TNFSF18/NEDD9 | 2 |
| BP | GO:0002220 | innate immune response activating cell surface receptor signaling pathway | 0.121843964 | 0.425645131 | 0.40277979 | PLCG2 | 1 |
| BP | GO:0002313 | mature B cell differentiation involved in immune response | 0.121843964 | 0.425645131 | 0.40277979 | PLCG2 | 1 |
| BP | GO:0006099 | tricarboxylic acid cycle | 0.121843964 | 0.425645131 | 0.40277979 | PDHA2 | 1 |
| BP | GO:0006783 | heme biosynthetic process | 0.121843964 | 0.425645131 | 0.40277979 | FXN | 1 |
| BP | GO:0010039 | response to iron ion | 0.121843964 | 0.425645131 | 0.40277979 | FXN | 1 |
| BP | GO:0019433 | triglyceride catabolic process | 0.121843964 | 0.425645131 | 0.40277979 | PLB1 | 1 |
| BP | GO:0051491 | positive regulation of filopodium assembly | 0.121843964 | 0.425645131 | 0.40277979 | DNM3 | 1 |
| BP | GO:1900745 | positive regulation of p38MAPK cascade | 0.121843964 | 0.425645131 | 0.40277979 | GADD45G | 1 |
| BP | GO:2000316 | regulation of T-helper 17 type immune response | 0.121843964 | 0.425645131 | 0.40277979 | TNFSF18 | 1 |
| BP | GO:0016055 | Wnt signaling pathway | 0.123564573 | 0.425645131 | 0.40277979 | ISL1/TMEM170B/GPC5/PLCG2 | 4 |
| BP | GO:0072073 | kidney epithelium development | 0.124478942 | 0.425645131 | 0.40277979 | IRX1/EYA1 | 2 |
| BP | GO:0198738 | cell-cell signaling by wnt | 0.124999008 | 0.425645131 | 0.40277979 | ISL1/TMEM170B/GPC5/PLCG2 | 4 |
| BP | GO:0002758 | innate immune response-activating signal transduction | 0.125520009 | 0.425645131 | 0.40277979 | PLCG2 | 1 |
| BP | GO:0003180 | aortic valve morphogenesis | 0.125520009 | 0.425645131 | 0.40277979 | ROBO1 | 1 |
| BP | GO:0007628 | adult walking behavior | 0.125520009 | 0.425645131 | 0.40277979 | FXN | 1 |
| BP | GO:0008156 | negative regulation of DNA replication | 0.125520009 | 0.425645131 | 0.40277979 | SMARCAL1 | 1 |
| BP | GO:0043552 | positive regulation of phosphatidylinositol 3-kinase activity | 0.125520009 | 0.425645131 | 0.40277979 | VAV2 | 1 |
| BP | GO:0050482 | arachidonic acid secretion | 0.125520009 | 0.425645131 | 0.40277979 | PLA2G2F | 1 |
| BP | GO:1903963 | arachidonate transport | 0.125520009 | 0.425645131 | 0.40277979 | PLA2G2F | 1 |
| BP | GO:0052547 | regulation of peptidase activity | 0.125718922 | 0.425645131 | 0.40277979 | FASLG/ROBO1/ST18/ITIH2 | 4 |
| BP | GO:1903038 | negative regulation of leukocyte cell-cell adhesion | 0.125868844 | 0.425645131 | 0.40277979 | PLA2G2F/TNFSF18 | 2 |
| BP | GO:0002687 | positive regulation of leukocyte migration | 0.12726252 | 0.425645131 | 0.40277979 | TNFSF18/NEDD9 | 2 |
| BP | GO:0015748 | organophosphate ester transport | 0.12726252 | 0.425645131 | 0.40277979 | FASLG/ATP8A2 | 2 |
| BP | GO:0050796 | regulation of insulin secretion | 0.128659915 | 0.425645131 | 0.40277979 | ISL1/CNR1 | 2 |
| BP | GO:0002431 | Fc receptor mediated stimulatory signaling pathway | 0.12918086 | 0.425645131 | 0.40277979 | VAV2 | 1 |
| BP | GO:0006084 | acetyl-CoA metabolic process | 0.12918086 | 0.425645131 | 0.40277979 | PDHA2 | 1 |
| BP | GO:0006654 | phosphatidic acid biosynthetic process | 0.12918086 | 0.425645131 | 0.40277979 | GPAM | 1 |
| BP | GO:0021772 | olfactory bulb development | 0.12918086 | 0.425645131 | 0.40277979 | ROBO1 | 1 |
| BP | GO:0038094 | Fc-gamma receptor signaling pathway | 0.12918086 | 0.425645131 | 0.40277979 | VAV2 | 1 |
| BP | GO:0060795 | cell fate commitment involved in formation of primary germ layer | 0.12918086 | 0.425645131 | 0.40277979 | EYA1 | 1 |
| BP | GO:0090659 | walking behavior | 0.12918086 | 0.425645131 | 0.40277979 | FXN | 1 |
| BP | GO:0032103 | positive regulation of response to external stimulus | 0.129345213 | 0.425645131 | 0.40277979 | TNFSF18/NEDD9/CNR1/PLCG2 | 4 |
| BP | GO:0098876 | vesicle-mediated transport to the plasma membrane | 0.130060977 | 0.425645131 | 0.40277979 | STX6/VPS53 | 2 |
| BP | GO:0006302 | double-strand break repair | 0.13066759 | 0.425645131 | 0.40277979 | SMARCAL1/EYA1/SLF2 | 3 |
| BP | GO:0009259 | ribonucleotide metabolic process | 0.130808086 | 0.425645131 | 0.40277979 | PDHA2/TJP2/GPAM/DLG2 | 4 |
| BP | GO:0006688 | glycosphingolipid biosynthetic process | 0.132826579 | 0.425645131 | 0.40277979 | ST8SIA1 | 1 |
| BP | GO:0007095 | mitotic G2 DNA damage checkpoint signaling | 0.132826579 | 0.425645131 | 0.40277979 | FOXN3 | 1 |
| BP | GO:0009954 | proximal/distal pattern formation | 0.132826579 | 0.425645131 | 0.40277979 | IRX1 | 1 |
| BP | GO:0032743 | positive regulation of interleukin-2 production | 0.132826579 | 0.425645131 | 0.40277979 | PLCG2 | 1 |
| BP | GO:0035025 | positive regulation of Rho protein signal transduction | 0.132826579 | 0.425645131 | 0.40277979 | ROBO1 | 1 |
| BP | GO:0035066 | positive regulation of histone acetylation | 0.132826579 | 0.425645131 | 0.40277979 | ISL1 | 1 |
| BP | GO:0035335 | peptidyl-tyrosine dephosphorylation | 0.132826579 | 0.425645131 | 0.40277979 | EYA1 | 1 |
| BP | GO:0036336 | dendritic cell migration | 0.132826579 | 0.425645131 | 0.40277979 | TNFSF18 | 1 |
| BP | GO:0048333 | mesodermal cell differentiation | 0.132826579 | 0.425645131 | 0.40277979 | EYA1 | 1 |
| BP | GO:0061081 | positive regulation of myeloid leukocyte cytokine production involved in immune response | 0.132826579 | 0.425645131 | 0.40277979 | PLCG2 | 1 |
| BP | GO:0110110 | positive regulation of animal organ morphogenesis | 0.132826579 | 0.425645131 | 0.40277979 | ROBO1 | 1 |
| BP | GO:1900181 | negative regulation of protein localization to nucleus | 0.132826579 | 0.425645131 | 0.40277979 | DCLK2 | 1 |
| BP | GO:0030183 | B cell differentiation | 0.132873882 | 0.425645131 | 0.40277979 | DNAJB9/PLCG2 | 2 |
| BP | GO:0072006 | nephron development | 0.132873882 | 0.425645131 | 0.40277979 | IRX1/EYA1 | 2 |
| BP | GO:0046651 | lymphocyte proliferation | 0.135388473 | 0.425645131 | 0.40277979 | PLA2G2F/TNFSF18/GPAM | 3 |
| BP | GO:0000723 | telomere maintenance | 0.135700807 | 0.425645131 | 0.40277979 | SMARCAL1/SLF2 | 2 |
| BP | GO:0007584 | response to nutrient | 0.135700807 | 0.425645131 | 0.40277979 | BCKDHB/CNR1 | 2 |
| BP | GO:0050905 | neuromuscular process | 0.135700807 | 0.425645131 | 0.40277979 | FXN/ATP8A2 | 2 |
| BP | GO:0051092 | positive regulation of NF-kappaB transcription factor activity | 0.135700807 | 0.425645131 | 0.40277979 | TNFSF18/PLCG2 | 2 |
| BP | GO:0006779 | porphyrin-containing compound biosynthetic process | 0.136457227 | 0.425645131 | 0.40277979 | FXN | 1 |
| BP | GO:0021988 | olfactory lobe development | 0.136457227 | 0.425645131 | 0.40277979 | ROBO1 | 1 |
| BP | GO:0030212 | hyaluronan metabolic process | 0.136457227 | 0.425645131 | 0.40277979 | ITIH2 | 1 |
| BP | GO:0031128 | developmental induction | 0.136457227 | 0.425645131 | 0.40277979 | ROBO1 | 1 |
| BP | GO:0033014 | tetrapyrrole biosynthetic process | 0.136457227 | 0.425645131 | 0.40277979 | FXN | 1 |
| BP | GO:0033146 | regulation of intracellular estrogen receptor signaling pathway | 0.136457227 | 0.425645131 | 0.40277979 | ISL1 | 1 |
| BP | GO:0042462 | eye photoreceptor cell development | 0.136457227 | 0.425645131 | 0.40277979 | TULP1 | 1 |
| BP | GO:0050685 | positive regulation of mRNA processing | 0.136457227 | 0.425645131 | 0.40277979 | PRDX6 | 1 |
| BP | GO:0051354 | negative regulation of oxidoreductase activity | 0.136457227 | 0.425645131 | 0.40277979 | CNR1 | 1 |
| BP | GO:0071425 | hematopoietic stem cell proliferation | 0.136457227 | 0.425645131 | 0.40277979 | ETV6 | 1 |
| BP | GO:0098664 | G protein-coupled serotonin receptor signaling pathway | 0.136457227 | 0.425645131 | 0.40277979 | HTR1E | 1 |
| BP | GO:1901099 | negative regulation of signal transduction in absence of ligand | 0.136457227 | 0.425645131 | 0.40277979 | EYA1 | 1 |
| BP | GO:1901658 | glycosyl compound catabolic process | 0.136457227 | 0.425645131 | 0.40277979 | ABHD10 | 1 |
| BP | GO:2000515 | negative regulation of CD4-positive, alpha-beta T cell activation | 0.136457227 | 0.425645131 | 0.40277979 | TNFSF18 | 1 |
| BP | GO:2001240 | negative regulation of extrinsic apoptotic signaling pathway in absence of ligand | 0.136457227 | 0.425645131 | 0.40277979 | EYA1 | 1 |
| BP | GO:0031667 | response to nutrient levels | 0.136728948 | 0.425645131 | 0.40277979 | RICTOR/BCKDHB/CNR1/MAX | 4 |
| BP | GO:0032368 | regulation of lipid transport | 0.137119395 | 0.425645131 | 0.40277979 | FASLG/ATP8A2 | 2 |
| BP | GO:0031349 | positive regulation of defense response | 0.137293453 | 0.425645131 | 0.40277979 | TNFSF18/CNR1/PLCG2 | 3 |
| BP | GO:0019693 | ribose phosphate metabolic process | 0.137476756 | 0.425645131 | 0.40277979 | PDHA2/TJP2/GPAM/DLG2 | 4 |
| BP | GO:0007162 | negative regulation of cell adhesion | 0.138249445 | 0.425645131 | 0.40277979 | PLA2G2F/TNFSF18/ADAMTS18 | 3 |
| BP | GO:0001822 | kidney development | 0.139207754 | 0.425645131 | 0.40277979 | IRX1/TBC1D32/EYA1 | 3 |
| BP | GO:0055074 | calcium ion homeostasis | 0.139207754 | 0.425645131 | 0.40277979 | FASLG/SCGN/PLCG2 | 3 |
| BP | GO:0007189 | adenylate cyclase-activating G protein-coupled receptor signaling pathway | 0.139966562 | 0.425645131 | 0.40277979 | CNR1/GPR26 | 2 |
| BP | GO:0010922 | positive regulation of phosphatase activity | 0.140072866 | 0.425645131 | 0.40277979 | GNA12 | 1 |
| BP | GO:0032228 | regulation of synaptic transmission, GABAergic | 0.140072866 | 0.425645131 | 0.40277979 | CNR1 | 1 |
| BP | GO:0035909 | aorta morphogenesis | 0.140072866 | 0.425645131 | 0.40277979 | EYA1 | 1 |
| BP | GO:0045777 | positive regulation of blood pressure | 0.140072866 | 0.425645131 | 0.40277979 | CNR1 | 1 |
| BP | GO:0046320 | regulation of fatty acid oxidation | 0.140072866 | 0.425645131 | 0.40277979 | CNR1 | 1 |
| BP | GO:0090218 | positive regulation of lipid kinase activity | 0.140072866 | 0.425645131 | 0.40277979 | VAV2 | 1 |
| BP | GO:0097484 | dendrite extension | 0.140072866 | 0.425645131 | 0.40277979 | SH3GL2 | 1 |
| BP | GO:1903514 | release of sequestered calcium ion into cytosol by endoplasmic reticulum | 0.140072866 | 0.425645131 | 0.40277979 | FASLG | 1 |
| BP | GO:0060070 | canonical Wnt signaling pathway | 0.140168363 | 0.425645131 | 0.40277979 | ISL1/TMEM170B/GPC5 | 3 |
| BP | GO:0050673 | epithelial cell proliferation | 0.141998876 | 0.425645131 | 0.40277979 | ROBO1/ISL1/EYA1/ST8SIA1 | 4 |
| BP | GO:0002429 | immune response-activating cell surface receptor signaling pathway | 0.142096423 | 0.425645131 | 0.40277979 | FYB1/VAV2/PLCG2 | 3 |
| BP | GO:0002757 | immune response-activating signal transduction | 0.142096423 | 0.425645131 | 0.40277979 | FYB1/VAV2/PLCG2 | 3 |
| BP | GO:0032943 | mononuclear cell proliferation | 0.142096423 | 0.425645131 | 0.40277979 | PLA2G2F/TNFSF18/GPAM | 3 |
| BP | GO:0007292 | female gamete generation | 0.142826706 | 0.425645131 | 0.40277979 | MEI4/ETV6 | 2 |
| BP | GO:0008064 | regulation of actin polymerization or depolymerization | 0.142826706 | 0.425645131 | 0.40277979 | RICTOR/ARPC1B | 2 |
| BP | GO:0002335 | mature B cell differentiation | 0.143673557 | 0.425645131 | 0.40277979 | PLCG2 | 1 |
| BP | GO:0003161 | cardiac conduction system development | 0.143673557 | 0.425645131 | 0.40277979 | ISL1 | 1 |
| BP | GO:0003176 | aortic valve development | 0.143673557 | 0.425645131 | 0.40277979 | ROBO1 | 1 |
| BP | GO:0003230 | cardiac atrium development | 0.143673557 | 0.425645131 | 0.40277979 | ISL1 | 1 |
| BP | GO:0010934 | macrophage cytokine production | 0.143673557 | 0.425645131 | 0.40277979 | PLCG2 | 1 |
| BP | GO:0010935 | regulation of macrophage cytokine production | 0.143673557 | 0.425645131 | 0.40277979 | PLCG2 | 1 |
| BP | GO:0014046 | dopamine secretion | 0.143673557 | 0.425645131 | 0.40277979 | CNR1 | 1 |
| BP | GO:0014059 | regulation of dopamine secretion | 0.143673557 | 0.425645131 | 0.40277979 | CNR1 | 1 |
| BP | GO:0032435 | negative regulation of proteasomal ubiquitin-dependent protein catabolic process | 0.143673557 | 0.425645131 | 0.40277979 | FHIT | 1 |
| BP | GO:0033144 | negative regulation of intracellular steroid hormone receptor signaling pathway | 0.143673557 | 0.425645131 | 0.40277979 | ISL1 | 1 |
| BP | GO:0034110 | regulation of homotypic cell-cell adhesion | 0.143673557 | 0.425645131 | 0.40277979 | ADAMTS18 | 1 |
| BP | GO:0046473 | phosphatidic acid metabolic process | 0.143673557 | 0.425645131 | 0.40277979 | GPAM | 1 |
| BP | GO:0070229 | negative regulation of lymphocyte apoptotic process | 0.143673557 | 0.425645131 | 0.40277979 | GPAM | 1 |
| BP | GO:0006909 | phagocytosis | 0.144033506 | 0.425938244 | 0.403057158 | TULP1/VAV2/PLCG2 | 3 |
| BP | GO:0120254 | olefinic compound metabolic process | 0.144261518 | 0.425938244 | 0.403057158 | PLA2G2F/PLB1 | 2 |
| BP | GO:0007034 | vacuolar transport | 0.147140369 | 0.431135812 | 0.407975516 | MON1B/VPS53 | 2 |
| BP | GO:0030832 | regulation of actin filament length | 0.147140369 | 0.431135812 | 0.407975516 | RICTOR/ARPC1B | 2 |
| BP | GO:0007617 | mating behavior | 0.147259362 | 0.431135812 | 0.407975516 | CNR1 | 1 |
| BP | GO:0043368 | positive T cell selection | 0.147259362 | 0.431135812 | 0.407975516 | TNFSF18 | 1 |
| BP | GO:0045742 | positive regulation of epidermal growth factor receptor signaling pathway | 0.147259362 | 0.431135812 | 0.407975516 | FASLG | 1 |
| BP | GO:0072001 | renal system development | 0.147934265 | 0.431371898 | 0.40819892 | IRX1/TBC1D32/EYA1 | 3 |
| BP | GO:0022407 | regulation of cell-cell adhesion | 0.148893101 | 0.431371898 | 0.40819892 | PLA2G2F/TNFSF18/GPAM/ADAMTS18 | 4 |
| BP | GO:0055067 | monovalent inorganic cation homeostasis | 0.150031191 | 0.431371898 | 0.40819892 | FASLG/SLC9C2 | 2 |
| BP | GO:0001667 | ameboidal-type cell migration | 0.15044284 | 0.431371898 | 0.40819892 | ROBO1/ISL1/GNA12/PLCG2 | 4 |
| BP | GO:0007340 | acrosome reaction | 0.15083034 | 0.431371898 | 0.40819892 | PLB1 | 1 |
| BP | GO:0021532 | neural tube patterning | 0.15083034 | 0.431371898 | 0.40819892 | TBC1D32 | 1 |
| BP | GO:0042755 | eating behavior | 0.15083034 | 0.431371898 | 0.40819892 | ATP8A2 | 1 |
| BP | GO:0045922 | negative regulation of fatty acid metabolic process | 0.15083034 | 0.431371898 | 0.40819892 | CNR1 | 1 |
| BP | GO:0046621 | negative regulation of organ growth | 0.15083034 | 0.431371898 | 0.40819892 | FXN | 1 |
| BP | GO:0070286 | axonemal dynein complex assembly | 0.15083034 | 0.431371898 | 0.40819892 | DNAH8 | 1 |
| BP | GO:0009101 | glycoprotein biosynthetic process | 0.150882633 | 0.431371898 | 0.40819892 | ALG6/XXYLT1/ST8SIA1 | 3 |
| BP | GO:0031960 | response to corticosteroid | 0.151480969 | 0.431371898 | 0.40819892 | ISL1/BCKDHB | 2 |
| BP | GO:0072503 | cellular divalent inorganic cation homeostasis | 0.152858843 | 0.431371898 | 0.40819892 | FASLG/SCGN/PLCG2 | 3 |
| BP | GO:0051250 | negative regulation of lymphocyte activation | 0.152933593 | 0.431371898 | 0.40819892 | PLA2G2F/TNFSF18 | 2 |
| BP | GO:0099173 | postsynapse organization | 0.152933593 | 0.431371898 | 0.40819892 | DNM3/NEDD9 | 2 |
| BP | GO:2001022 | positive regulation of response to DNA damage stimulus | 0.152933593 | 0.431371898 | 0.40819892 | EYA1/SLF2 | 2 |
| BP | GO:0043410 | positive regulation of MAPK cascade | 0.153561218 | 0.431371898 | 0.40819892 | ROBO1/ALKAL1/GADD45G/PLCG2 | 4 |
| BP | GO:0007210 | serotonin receptor signaling pathway | 0.154386553 | 0.431371898 | 0.40819892 | HTR1E | 1 |
| BP | GO:0010762 | regulation of fibroblast migration | 0.154386553 | 0.431371898 | 0.40819892 | GNA12 | 1 |
| BP | GO:0033120 | positive regulation of RNA splicing | 0.154386553 | 0.431371898 | 0.40819892 | PRDX6 | 1 |
| BP | GO:0048246 | macrophage chemotaxis | 0.154386553 | 0.431371898 | 0.40819892 | TNFSF18 | 1 |
| BP | GO:1901186 | positive regulation of ERBB signaling pathway | 0.154386553 | 0.431371898 | 0.40819892 | FASLG | 1 |
| BP | GO:2000758 | positive regulation of peptidyl-lysine acetylation | 0.154386553 | 0.431371898 | 0.40819892 | ISL1 | 1 |
| BP | GO:0003205 | cardiac chamber development | 0.154389015 | 0.431371898 | 0.40819892 | ROBO1/ISL1 | 2 |
| BP | GO:0030307 | positive regulation of cell growth | 0.154389015 | 0.431371898 | 0.40819892 | RICTOR/FXN | 2 |
| BP | GO:0044089 | positive regulation of cellular component biogenesis | 0.155129751 | 0.431371898 | 0.40819892 | DNM3/RICTOR/SLF2/PLCG2 | 4 |
| BP | GO:0010970 | transport along microtubule | 0.155847187 | 0.431371898 | 0.40819892 | SPAG17/DLG2 | 2 |
| BP | GO:0048639 | positive regulation of developmental growth | 0.155847187 | 0.431371898 | 0.40819892 | GPAM/ATP8A2 | 2 |
| BP | GO:0006040 | amino sugar metabolic process | 0.157928061 | 0.431371898 | 0.40819892 | CMAS | 1 |
| BP | GO:0014912 | negative regulation of smooth muscle cell migration | 0.157928061 | 0.431371898 | 0.40819892 | GNA12 | 1 |
| BP | GO:0019432 | triglyceride biosynthetic process | 0.157928061 | 0.431371898 | 0.40819892 | GPAM | 1 |
| BP | GO:0032733 | positive regulation of interleukin-10 production | 0.157928061 | 0.431371898 | 0.40819892 | PLCG2 | 1 |
| BP | GO:0045454 | cell redox homeostasis | 0.157928061 | 0.431371898 | 0.40819892 | PRDX6 | 1 |
| BP | GO:0046461 | neutral lipid catabolic process | 0.157928061 | 0.431371898 | 0.40819892 | PLB1 | 1 |
| BP | GO:0046464 | acylglycerol catabolic process | 0.157928061 | 0.431371898 | 0.40819892 | PLB1 | 1 |
| BP | GO:0050850 | positive regulation of calcium-mediated signaling | 0.157928061 | 0.431371898 | 0.40819892 | PLCG2 | 1 |
| BP | GO:0070050 | neuron cellular homeostasis | 0.157928061 | 0.431371898 | 0.40819892 | SCGN | 1 |
| BP | GO:0070232 | regulation of T cell apoptotic process | 0.157928061 | 0.431371898 | 0.40819892 | GPAM | 1 |
| BP | GO:0070884 | regulation of calcineurin-NFAT signaling cascade | 0.157928061 | 0.431371898 | 0.40819892 | PLCG2 | 1 |
| BP | GO:0072210 | metanephric nephron development | 0.157928061 | 0.431371898 | 0.40819892 | IRX1 | 1 |
| BP | GO:0150077 | regulation of neuroinflammatory response | 0.157928061 | 0.431371898 | 0.40819892 | PLCG2 | 1 |
| BP | GO:1905314 | semi-lunar valve development | 0.157928061 | 0.431371898 | 0.40819892 | ROBO1 | 1 |
| BP | GO:0030041 | actin filament polymerization | 0.158771593 | 0.431371898 | 0.40819892 | RICTOR/ARPC1B | 2 |
| BP | GO:0050806 | positive regulation of synaptic transmission | 0.158771593 | 0.431371898 | 0.40819892 | SCGN/CNR1 | 2 |
| BP | GO:2000058 | regulation of ubiquitin-dependent protein catabolic process | 0.158771593 | 0.431371898 | 0.40819892 | FHIT/GNA12 | 2 |
| BP | GO:0016482 | cytosolic transport | 0.160237732 | 0.431371898 | 0.40819892 | STX6/VPS53 | 2 |
| BP | GO:0003351 | epithelial cilium movement involved in extracellular fluid movement | 0.161454924 | 0.431371898 | 0.40819892 | SPAG17 | 1 |
| BP | GO:0006623 | protein targeting to vacuole | 0.161454924 | 0.431371898 | 0.40819892 | MON1B | 1 |
| BP | GO:0010453 | regulation of cell fate commitment | 0.161454924 | 0.431371898 | 0.40819892 | TNFSF18 | 1 |
| BP | GO:0021983 | pituitary gland development | 0.161454924 | 0.431371898 | 0.40819892 | ISL1 | 1 |
| BP | GO:0043029 | T cell homeostasis | 0.161454924 | 0.431371898 | 0.40819892 | GPAM | 1 |
| BP | GO:0050892 | intestinal absorption | 0.161454924 | 0.431371898 | 0.40819892 | TJP2 | 1 |
| BP | GO:0050999 | regulation of nitric-oxide synthase activity | 0.161454924 | 0.431371898 | 0.40819892 | CNR1 | 1 |
| BP | GO:0071354 | cellular response to interleukin-6 | 0.161454924 | 0.431371898 | 0.40819892 | ST18 | 1 |
| BP | GO:0086091 | regulation of heart rate by cardiac conduction | 0.161454924 | 0.431371898 | 0.40819892 | ISL1 | 1 |
| BP | GO:0106056 | regulation of calcineurin-mediated signaling | 0.161454924 | 0.431371898 | 0.40819892 | PLCG2 | 1 |
| BP | GO:2000403 | positive regulation of lymphocyte migration | 0.161454924 | 0.431371898 | 0.40819892 | NEDD9 | 1 |
| BP | GO:0007259 | receptor signaling pathway via JAK-STAT | 0.161706433 | 0.431382246 | 0.408208711 | TNFSF18/ISL1 | 2 |
| BP | GO:0021543 | pallium development | 0.164651338 | 0.433748537 | 0.410447888 | ROBO1/DCLK2 | 2 |
| BP | GO:0021953 | central nervous system neuron differentiation | 0.164651338 | 0.433748537 | 0.410447888 | DCLK2/ISL1 | 2 |
| BP | GO:0030178 | negative regulation of Wnt signaling pathway | 0.164651338 | 0.433748537 | 0.410447888 | ISL1/TMEM170B | 2 |
| BP | GO:0016266 | O-glycan processing | 0.164967201 | 0.433748537 | 0.410447888 | XXYLT1 | 1 |
| BP | GO:0021879 | forebrain neuron differentiation | 0.164967201 | 0.433748537 | 0.410447888 | DCLK2 | 1 |
| BP | GO:0045494 | photoreceptor cell maintenance | 0.164967201 | 0.433748537 | 0.410447888 | TULP1 | 1 |
| BP | GO:0045622 | regulation of T-helper cell differentiation | 0.164967201 | 0.433748537 | 0.410447888 | TNFSF18 | 1 |
| BP | GO:0060412 | ventricular septum morphogenesis | 0.164967201 | 0.433748537 | 0.410447888 | ROBO1 | 1 |
| BP | GO:1904037 | positive regulation of epithelial cell apoptotic process | 0.164967201 | 0.433748537 | 0.410447888 | FASLG | 1 |
| BP | GO:0030111 | regulation of Wnt signaling pathway | 0.165902834 | 0.433748537 | 0.410447888 | ISL1/TMEM170B/GPC5 | 3 |
| BP | GO:0032635 | interleukin-6 production | 0.167605941 | 0.433748537 | 0.410447888 | ISL1/PLCG2 | 2 |
| BP | GO:0032675 | regulation of interleukin-6 production | 0.167605941 | 0.433748537 | 0.410447888 | ISL1/PLCG2 | 2 |
| BP | GO:0050680 | negative regulation of epithelial cell proliferation | 0.167605941 | 0.433748537 | 0.410447888 | ROBO1/ISL1 | 2 |
| BP | GO:0006790 | sulfur compound metabolic process | 0.167938896 | 0.433748537 | 0.410447888 | PDHA2/FXN/GPAM | 3 |
| BP | GO:0001709 | cell fate determination | 0.168464953 | 0.433748537 | 0.410447888 | ISL1 | 1 |
| BP | GO:0003009 | skeletal muscle contraction | 0.168464953 | 0.433748537 | 0.410447888 | ATP8A2 | 1 |
| BP | GO:0044275 | cellular carbohydrate catabolic process | 0.168464953 | 0.433748537 | 0.410447888 | ABHD10 | 1 |
| BP | GO:0061001 | regulation of dendritic spine morphogenesis | 0.168464953 | 0.433748537 | 0.410447888 | DNM3 | 1 |
| BP | GO:0072538 | T-helper 17 type immune response | 0.168464953 | 0.433748537 | 0.410447888 | TNFSF18 | 1 |
| BP | GO:1900026 | positive regulation of substrate adhesion-dependent cell spreading | 0.168464953 | 0.433748537 | 0.410447888 | NEDD9 | 1 |
| BP | GO:1903573 | negative regulation of response to endoplasmic reticulum stress | 0.168464953 | 0.433748537 | 0.410447888 | DNAJB9 | 1 |
| BP | GO:1904706 | negative regulation of vascular associated smooth muscle cell proliferation | 0.168464953 | 0.433748537 | 0.410447888 | GNA12 | 1 |
| BP | GO:1905521 | regulation of macrophage migration | 0.168464953 | 0.433748537 | 0.410447888 | TNFSF18 | 1 |
| BP | GO:0032496 | response to lipopolysaccharide | 0.168959739 | 0.433748537 | 0.410447888 | FASLG/CNR1/PLCG2 | 3 |
| BP | GO:0001659 | temperature homeostasis | 0.170569884 | 0.433748537 | 0.410447888 | CNR1/GADD45G | 2 |
| BP | GO:0048736 | appendage development | 0.170569884 | 0.433748537 | 0.410447888 | TBC1D32/GNA12 | 2 |
| BP | GO:0060173 | limb development | 0.170569884 | 0.433748537 | 0.410447888 | TBC1D32/GNA12 | 2 |
| BP | GO:0002768 | immune response-regulating cell surface receptor signaling pathway | 0.171006971 | 0.433748537 | 0.410447888 | FYB1/VAV2/PLCG2 | 3 |
| BP | GO:0006858 | extracellular transport | 0.171948237 | 0.433748537 | 0.410447888 | SPAG17 | 1 |
| BP | GO:0019098 | reproductive behavior | 0.171948237 | 0.433748537 | 0.410447888 | CNR1 | 1 |
| BP | GO:0033003 | regulation of mast cell activation | 0.171948237 | 0.433748537 | 0.410447888 | CNR1 | 1 |
| BP | GO:0035384 | thioester biosynthetic process | 0.171948237 | 0.433748537 | 0.410447888 | PDHA2 | 1 |
| BP | GO:0046636 | negative regulation of alpha-beta T cell activation | 0.171948237 | 0.433748537 | 0.410447888 | TNFSF18 | 1 |
| BP | GO:0070741 | response to interleukin-6 | 0.171948237 | 0.433748537 | 0.410447888 | ST18 | 1 |
| BP | GO:0071616 | acyl-CoA biosynthetic process | 0.171948237 | 0.433748537 | 0.410447888 | PDHA2 | 1 |
| BP | GO:1900744 | regulation of p38MAPK cascade | 0.171948237 | 0.433748537 | 0.410447888 | GADD45G | 1 |
| BP | GO:1901021 | positive regulation of calcium ion transmembrane transporter activity | 0.171948237 | 0.433748537 | 0.410447888 | PLCG2 | 1 |
| BP | GO:0090276 | regulation of peptide hormone secretion | 0.172055246 | 0.433748537 | 0.410447888 | ISL1/CNR1 | 2 |
| BP | GO:0021517 | ventral spinal cord development | 0.175417115 | 0.435051094 | 0.411680472 | ISL1 | 1 |
| BP | GO:0021795 | cerebral cortex cell migration | 0.175417115 | 0.435051094 | 0.411680472 | ROBO1 | 1 |
| BP | GO:0033173 | calcineurin-NFAT signaling cascade | 0.175417115 | 0.435051094 | 0.411680472 | PLCG2 | 1 |
| BP | GO:0042168 | heme metabolic process | 0.175417115 | 0.435051094 | 0.411680472 | FXN | 1 |
| BP | GO:0046006 | regulation of activated T cell proliferation | 0.175417115 | 0.435051094 | 0.411680472 | GPAM | 1 |
| BP | GO:0055010 | ventricular cardiac muscle tissue morphogenesis | 0.175417115 | 0.435051094 | 0.411680472 | ISL1 | 1 |
| BP | GO:0070266 | necroptotic process | 0.175417115 | 0.435051094 | 0.411680472 | FASLG | 1 |
| BP | GO:1900271 | regulation of long-term synaptic potentiation | 0.175417115 | 0.435051094 | 0.411680472 | SCGN | 1 |
| BP | GO:1904752 | regulation of vascular associated smooth muscle cell migration | 0.175417115 | 0.435051094 | 0.411680472 | GNA12 | 1 |
| BP | GO:1990090 | cellular response to nerve growth factor stimulus | 0.175417115 | 0.435051094 | 0.411680472 | SH3GL2 | 1 |
| BP | GO:2001239 | regulation of extrinsic apoptotic signaling pathway in absence of ligand | 0.175417115 | 0.435051094 | 0.411680472 | EYA1 | 1 |
| BP | GO:0002791 | regulation of peptide secretion | 0.176524372 | 0.435051094 | 0.411680472 | ISL1/CNR1 | 2 |
| BP | GO:0097553 | calcium ion transmembrane import into cytosol | 0.176524372 | 0.435051094 | 0.411680472 | FASLG/PLCG2 | 2 |
| BP | GO:0019216 | regulation of lipid metabolic process | 0.177191997 | 0.435051094 | 0.411680472 | CNR1/VAV2/PLCG2 | 3 |
| BP | GO:0030073 | insulin secretion | 0.178018282 | 0.435051094 | 0.411680472 | ISL1/CNR1 | 2 |
| BP | GO:0097696 | receptor signaling pathway via STAT | 0.178018282 | 0.435051094 | 0.411680472 | TNFSF18/ISL1 | 2 |
| BP | GO:0070661 | leukocyte proliferation | 0.178228998 | 0.435051094 | 0.411680472 | PLA2G2F/TNFSF18/GPAM | 3 |
| BP | GO:0002861 | regulation of inflammatory response to antigenic stimulus | 0.178871643 | 0.435051094 | 0.411680472 | CNR1 | 1 |
| BP | GO:0034314 | Arp2/3 complex-mediated actin nucleation | 0.178871643 | 0.435051094 | 0.411680472 | ARPC1B | 1 |
| BP | GO:0090199 | regulation of release of cytochrome c from mitochondria | 0.178871643 | 0.435051094 | 0.411680472 | FXN | 1 |
| BP | GO:2001222 | regulation of neuron migration | 0.178871643 | 0.435051094 | 0.411680472 | UNC5C | 1 |
| BP | GO:0032640 | tumor necrosis factor production | 0.179514222 | 0.435051094 | 0.411680472 | ISL1/PLCG2 | 2 |
| BP | GO:0032680 | regulation of tumor necrosis factor production | 0.179514222 | 0.435051094 | 0.411680472 | ISL1/PLCG2 | 2 |
| BP | GO:0090087 | regulation of peptide transport | 0.179514222 | 0.435051094 | 0.411680472 | ISL1/CNR1 | 2 |
| BP | GO:0032200 | telomere organization | 0.18101215 | 0.435051094 | 0.411680472 | SMARCAL1/SLF2 | 2 |
| BP | GO:1905952 | regulation of lipid localization | 0.18101215 | 0.435051094 | 0.411680472 | FASLG/ATP8A2 | 2 |
| BP | GO:0001754 | eye photoreceptor cell differentiation | 0.182311882 | 0.435051094 | 0.411680472 | TULP1 | 1 |
| BP | GO:0002673 | regulation of acute inflammatory response | 0.182311882 | 0.435051094 | 0.411680472 | CNR1 | 1 |
| BP | GO:0006953 | acute-phase response | 0.182311882 | 0.435051094 | 0.411680472 | CNR1 | 1 |
| BP | GO:0031297 | replication fork processing | 0.182311882 | 0.435051094 | 0.411680472 | SMARCAL1 | 1 |
| BP | GO:0032008 | positive regulation of TOR signaling | 0.182311882 | 0.435051094 | 0.411680472 | RICTOR | 1 |
| BP | GO:0032309 | icosanoid secretion | 0.182311882 | 0.435051094 | 0.411680472 | PLA2G2F | 1 |
| BP | GO:0042220 | response to cocaine | 0.182311882 | 0.435051094 | 0.411680472 | CNR1 | 1 |
| BP | GO:0045581 | negative regulation of T cell differentiation | 0.182311882 | 0.435051094 | 0.411680472 | TNFSF18 | 1 |
| BP | GO:0045747 | positive regulation of Notch signaling pathway | 0.182311882 | 0.435051094 | 0.411680472 | ROBO1 | 1 |
| BP | GO:0045776 | negative regulation of blood pressure | 0.182311882 | 0.435051094 | 0.411680472 | CNR1 | 1 |
| BP | GO:0061028 | establishment of endothelial barrier | 0.182311882 | 0.435051094 | 0.411680472 | TJP2 | 1 |
| BP | GO:1904738 | vascular associated smooth muscle cell migration | 0.182311882 | 0.435051094 | 0.411680472 | GNA12 | 1 |
| BP | GO:1990089 | response to nerve growth factor | 0.182311882 | 0.435051094 | 0.411680472 | SH3GL2 | 1 |
| BP | GO:0002700 | regulation of production of molecular mediator of immune response | 0.1840138 | 0.437237031 | 0.413748982 | DNAJB9/PLCG2 | 2 |
| BP | GO:0008089 | anterograde axonal transport | 0.185737889 | 0.437237031 | 0.413748982 | DLG2 | 1 |
| BP | GO:0010862 | positive regulation of pathway-restricted SMAD protein phosphorylation | 0.185737889 | 0.437237031 | 0.413748982 | BMP3 | 1 |
| BP | GO:0035094 | response to nicotine | 0.185737889 | 0.437237031 | 0.413748982 | CNR1 | 1 |
| BP | GO:0035307 | positive regulation of protein dephosphorylation | 0.185737889 | 0.437237031 | 0.413748982 | GNA12 | 1 |
| BP | GO:0046460 | neutral lipid biosynthetic process | 0.185737889 | 0.437237031 | 0.413748982 | GPAM | 1 |
| BP | GO:0046463 | acylglycerol biosynthetic process | 0.185737889 | 0.437237031 | 0.413748982 | GPAM | 1 |
| BP | GO:0050798 | activated T cell proliferation | 0.185737889 | 0.437237031 | 0.413748982 | GPAM | 1 |
| BP | GO:0055081 | anion homeostasis | 0.185737889 | 0.437237031 | 0.413748982 | FASLG | 1 |
| BP | GO:1903426 | regulation of reactive oxygen species biosynthetic process | 0.185737889 | 0.437237031 | 0.413748982 | PLCG2 | 1 |
| BP | GO:0072507 | divalent inorganic cation homeostasis | 0.186585788 | 0.437882038 | 0.41435934 | FASLG/SCGN/PLCG2 | 3 |
| BP | GO:0071706 | tumor necrosis factor superfamily cytokine production | 0.187022899 | 0.437882038 | 0.41435934 | ISL1/PLCG2 | 2 |
| BP | GO:1903555 | regulation of tumor necrosis factor superfamily cytokine production | 0.187022899 | 0.437882038 | 0.41435934 | ISL1/PLCG2 | 2 |
| BP | GO:0021515 | cell differentiation in spinal cord | 0.189149722 | 0.437882038 | 0.41435934 | ISL1 | 1 |
| BP | GO:0030195 | negative regulation of blood coagulation | 0.189149722 | 0.437882038 | 0.41435934 | ADAMTS18 | 1 |
| BP | GO:0038084 | vascular endothelial growth factor signaling pathway | 0.189149722 | 0.437882038 | 0.41435934 | ROBO1 | 1 |
| BP | GO:0042461 | photoreceptor cell development | 0.189149722 | 0.437882038 | 0.41435934 | TULP1 | 1 |
| BP | GO:0044818 | mitotic G2/M transition checkpoint | 0.189149722 | 0.437882038 | 0.41435934 | FOXN3 | 1 |
| BP | GO:0051349 | positive regulation of lyase activity | 0.189149722 | 0.437882038 | 0.41435934 | FXN | 1 |
| BP | GO:0061082 | myeloid leukocyte cytokine production | 0.189149722 | 0.437882038 | 0.41435934 | PLCG2 | 1 |
| BP | GO:0090102 | cochlea development | 0.189149722 | 0.437882038 | 0.41435934 | EYA1 | 1 |
| BP | GO:2000059 | negative regulation of ubiquitin-dependent protein catabolic process | 0.189149722 | 0.437882038 | 0.41435934 | FHIT | 1 |
| BP | GO:0008217 | regulation of blood pressure | 0.190039121 | 0.437882038 | 0.41435934 | CNR1/GNA12 | 2 |
| BP | GO:0001655 | urogenital system development | 0.190803188 | 0.437882038 | 0.41435934 | IRX1/TBC1D32/EYA1 | 3 |
| BP | GO:0002237 | response to molecule of bacterial origin | 0.190803188 | 0.437882038 | 0.41435934 | FASLG/CNR1/PLCG2 | 3 |
| BP | GO:0010038 | response to metal ion | 0.190803188 | 0.437882038 | 0.41435934 | FXN/DLG2/PLCG2 | 3 |
| BP | GO:0015872 | dopamine transport | 0.19254744 | 0.437882038 | 0.41435934 | CNR1 | 1 |
| BP | GO:0019083 | viral transcription | 0.19254744 | 0.437882038 | 0.41435934 | MON1B | 1 |
| BP | GO:0045058 | T cell selection | 0.19254744 | 0.437882038 | 0.41435934 | TNFSF18 | 1 |
| BP | GO:0045197 | establishment or maintenance of epithelial cell apical/basal polarity | 0.19254744 | 0.437882038 | 0.41435934 | DLG2 | 1 |
| BP | GO:0051489 | regulation of filopodium assembly | 0.19254744 | 0.437882038 | 0.41435934 | DNM3 | 1 |
| BP | GO:0097720 | calcineurin-mediated signaling | 0.19254744 | 0.437882038 | 0.41435934 | PLCG2 | 1 |
| BP | GO:1900047 | negative regulation of hemostasis | 0.19254744 | 0.437882038 | 0.41435934 | ADAMTS18 | 1 |
| BP | GO:1901799 | negative regulation of proteasomal protein catabolic process | 0.19254744 | 0.437882038 | 0.41435934 | FHIT | 1 |
| BP | GO:1901985 | positive regulation of protein acetylation | 0.19254744 | 0.437882038 | 0.41435934 | ISL1 | 1 |
| BP | GO:1903307 | positive regulation of regulated secretory pathway | 0.19254744 | 0.437882038 | 0.41435934 | CNR1 | 1 |
| BP | GO:0060271 | cilium assembly | 0.192921235 | 0.437909182 | 0.414385026 | SPAG17/DNAH8/TBC1D32 | 3 |
| BP | GO:0048839 | inner ear development | 0.193062142 | 0.437909182 | 0.414385026 | EYA1/ATP8A2 | 2 |
| BP | GO:0009060 | aerobic respiration | 0.194576103 | 0.439669289 | 0.416050581 | PDHA2/FXN | 2 |
| BP | GO:0061136 | regulation of proteasomal protein catabolic process | 0.194576103 | 0.439669289 | 0.416050581 | FHIT/GNA12 | 2 |
| BP | GO:0007129 | homologous chromosome pairing at meiosis | 0.1959311 | 0.439669289 | 0.416050581 | MEI4 | 1 |
| BP | GO:0010543 | regulation of platelet activation | 0.1959311 | 0.439669289 | 0.416050581 | ADAMTS18 | 1 |
| BP | GO:0010823 | negative regulation of mitochondrion organization | 0.1959311 | 0.439669289 | 0.416050581 | FXN | 1 |
| BP | GO:0021872 | forebrain generation of neurons | 0.1959311 | 0.439669289 | 0.416050581 | DCLK2 | 1 |
| BP | GO:0051452 | intracellular pH reduction | 0.1959311 | 0.439669289 | 0.416050581 | FASLG | 1 |
| BP | GO:0051480 | regulation of cytosolic calcium ion concentration | 0.1959311 | 0.439669289 | 0.416050581 | SCGN | 1 |
| BP | GO:0032956 | regulation of actin cytoskeleton organization | 0.196109666 | 0.439669289 | 0.416050581 | RICTOR/NEDD9/ARPC1B | 3 |
| BP | GO:0045664 | regulation of neuron differentiation | 0.19760873 | 0.441169548 | 0.417470247 | ISL1/EYA1 | 2 |
| BP | GO:0032535 | regulation of cellular component size | 0.198242693 | 0.441169548 | 0.417470247 | RICTOR/ARPC1B/VAV2 | 3 |
| BP | GO:0002639 | positive regulation of immunoglobulin production | 0.199300759 | 0.441169548 | 0.417470247 | DNAJB9 | 1 |
| BP | GO:0006778 | porphyrin-containing compound metabolic process | 0.199300759 | 0.441169548 | 0.417470247 | FXN | 1 |
| BP | GO:0030838 | positive regulation of actin filament polymerization | 0.199300759 | 0.441169548 | 0.417470247 | RICTOR | 1 |
| BP | GO:0048260 | positive regulation of receptor-mediated endocytosis | 0.199300759 | 0.441169548 | 0.417470247 | PLCG2 | 1 |
| BP | GO:0071622 | regulation of granulocyte chemotaxis | 0.199300759 | 0.441169548 | 0.417470247 | TNFSF18 | 1 |
| BP | GO:0097300 | programmed necrotic cell death | 0.199300759 | 0.441169548 | 0.417470247 | FASLG | 1 |
| BP | GO:0030336 | negative regulation of cell migration | 0.199311386 | 0.441169548 | 0.417470247 | ROBO1/NEDD9/GNA12 | 3 |
| BP | GO:0070997 | neuron death | 0.199311386 | 0.441169548 | 0.417470247 | FASLG/ISL1/MAX | 3 |
| BP | GO:0071248 | cellular response to metal ion | 0.200647371 | 0.443563096 | 0.419735216 | DLG2/PLCG2 | 2 |
| BP | GO:0030520 | intracellular estrogen receptor signaling pathway | 0.202656475 | 0.446305409 | 0.422330214 | ISL1 | 1 |
| BP | GO:0042572 | retinol metabolic process | 0.202656475 | 0.446305409 | 0.422330214 | PLB1 | 1 |
| BP | GO:0050819 | negative regulation of coagulation | 0.202656475 | 0.446305409 | 0.422330214 | ADAMTS18 | 1 |
| BP | GO:0045862 | positive regulation of proteolysis | 0.203600355 | 0.446329658 | 0.42235316 | FASLG/ROBO1/ST18 | 3 |
| BP | GO:0008154 | actin polymerization or depolymerization | 0.203691719 | 0.446329658 | 0.42235316 | RICTOR/ARPC1B | 2 |
| BP | GO:0032271 | regulation of protein polymerization | 0.205215939 | 0.446329658 | 0.42235316 | RICTOR/ARPC1B | 2 |
| BP | GO:0003179 | heart valve morphogenesis | 0.205998304 | 0.446329658 | 0.42235316 | ROBO1 | 1 |
| BP | GO:0003229 | ventricular cardiac muscle tissue development | 0.205998304 | 0.446329658 | 0.42235316 | ISL1 | 1 |
| BP | GO:0010761 | fibroblast migration | 0.205998304 | 0.446329658 | 0.42235316 | GNA12 | 1 |
| BP | GO:0031103 | axon regeneration | 0.205998304 | 0.446329658 | 0.42235316 | ISL1 | 1 |
| BP | GO:0035088 | establishment or maintenance of apical/basal cell polarity | 0.205998304 | 0.446329658 | 0.42235316 | DLG2 | 1 |
| BP | GO:0043525 | positive regulation of neuron apoptotic process | 0.205998304 | 0.446329658 | 0.42235316 | FASLG | 1 |
| BP | GO:0043647 | inositol phosphate metabolic process | 0.205998304 | 0.446329658 | 0.42235316 | PLCG2 | 1 |
| BP | GO:0061245 | establishment or maintenance of bipolar cell polarity | 0.205998304 | 0.446329658 | 0.42235316 | DLG2 | 1 |
| BP | GO:0071385 | cellular response to glucocorticoid stimulus | 0.205998304 | 0.446329658 | 0.42235316 | ISL1 | 1 |
| BP | GO:2000107 | negative regulation of leukocyte apoptotic process | 0.205998304 | 0.446329658 | 0.42235316 | GPAM | 1 |
| BP | GO:0050863 | regulation of T cell activation | 0.207911361 | 0.447280074 | 0.423252521 | PLA2G2F/TNFSF18/GPAM | 3 |
| BP | GO:1903037 | regulation of leukocyte cell-cell adhesion | 0.208992451 | 0.447280074 | 0.423252521 | PLA2G2F/TNFSF18/GPAM | 3 |
| BP | GO:0001836 | release of cytochrome c from mitochondria | 0.209326303 | 0.447280074 | 0.423252521 | FXN | 1 |
| BP | GO:0010718 | positive regulation of epithelial to mesenchymal transition | 0.209326303 | 0.447280074 | 0.423252521 | ISL1 | 1 |
| BP | GO:0021545 | cranial nerve development | 0.209326303 | 0.447280074 | 0.423252521 | ISL1 | 1 |
| BP | GO:0043370 | regulation of CD4-positive, alpha-beta T cell differentiation | 0.209326303 | 0.447280074 | 0.423252521 | TNFSF18 | 1 |
| BP | GO:0043551 | regulation of phosphatidylinositol 3-kinase activity | 0.209326303 | 0.447280074 | 0.423252521 | VAV2 | 1 |
| BP | GO:0050832 | defense response to fungus | 0.209326303 | 0.447280074 | 0.423252521 | PLCG2 | 1 |
| BP | GO:0060998 | regulation of dendritic spine development | 0.209326303 | 0.447280074 | 0.423252521 | DNM3 | 1 |
| BP | GO:0071715 | icosanoid transport | 0.209326303 | 0.447280074 | 0.423252521 | PLA2G2F | 1 |
| BP | GO:0097120 | receptor localization to synapse | 0.209326303 | 0.447280074 | 0.423252521 | DLG2 | 1 |
| BP | GO:0002697 | regulation of immune effector process | 0.211158537 | 0.447280074 | 0.423252521 | TNFSF18/DNAJB9/PLCG2 | 3 |
| BP | GO:0002823 | negative regulation of adaptive immune response based on somatic recombination of immune receptors built from immunoglobulin superfamily domains | 0.21264053 | 0.447280074 | 0.423252521 | TNFSF18 | 1 |
| BP | GO:0032768 | regulation of monooxygenase activity | 0.21264053 | 0.447280074 | 0.423252521 | CNR1 | 1 |
| BP | GO:0038066 | p38MAPK cascade | 0.21264053 | 0.447280074 | 0.423252521 | GADD45G | 1 |
| BP | GO:0043113 | receptor clustering | 0.21264053 | 0.447280074 | 0.423252521 | DLG2 | 1 |
| BP | GO:0043388 | positive regulation of DNA binding | 0.21264053 | 0.447280074 | 0.423252521 | ISL1 | 1 |
| BP | GO:0045005 | DNA-templated DNA replication maintenance of fidelity | 0.21264053 | 0.447280074 | 0.423252521 | SMARCAL1 | 1 |
| BP | GO:0046148 | pigment biosynthetic process | 0.21264053 | 0.447280074 | 0.423252521 | FXN | 1 |
| BP | GO:0050433 | regulation of catecholamine secretion | 0.21264053 | 0.447280074 | 0.423252521 | CNR1 | 1 |
| BP | GO:0050879 | multicellular organismal movement | 0.21264053 | 0.447280074 | 0.423252521 | ATP8A2 | 1 |
| BP | GO:0050881 | musculoskeletal movement | 0.21264053 | 0.447280074 | 0.423252521 | ATP8A2 | 1 |
| BP | GO:0051932 | synaptic transmission, GABAergic | 0.21264053 | 0.447280074 | 0.423252521 | CNR1 | 1 |
| BP | GO:0090329 | regulation of DNA-templated DNA replication | 0.21264053 | 0.447280074 | 0.423252521 | SMARCAL1 | 1 |
| BP | GO:0071674 | mononuclear cell migration | 0.212856017 | 0.447280074 | 0.423252521 | TNFSF18/NEDD9 | 2 |
| BP | GO:0002285 | lymphocyte activation involved in immune response | 0.214387573 | 0.448354338 | 0.424269076 | TNFSF18/PLCG2 | 2 |
| BP | GO:0030900 | forebrain development | 0.21550595 | 0.448354338 | 0.424269076 | ROBO1/DCLK2/ISL1 | 3 |
| BP | GO:2000146 | negative regulation of cell motility | 0.21550595 | 0.448354338 | 0.424269076 | ROBO1/NEDD9/GNA12 | 3 |
| BP | GO:0030705 | cytoskeleton-dependent intracellular transport | 0.215920225 | 0.448354338 | 0.424269076 | SPAG17/DLG2 | 2 |
| BP | GO:0019369 | arachidonic acid metabolic process | 0.215941039 | 0.448354338 | 0.424269076 | PLA2G2F | 1 |
| BP | GO:0045620 | negative regulation of lymphocyte differentiation | 0.215941039 | 0.448354338 | 0.424269076 | TNFSF18 | 1 |
| BP | GO:0048010 | vascular endothelial growth factor receptor signaling pathway | 0.215941039 | 0.448354338 | 0.424269076 | VAV2 | 1 |
| BP | GO:0048016 | inositol phosphate-mediated signaling | 0.215941039 | 0.448354338 | 0.424269076 | PLCG2 | 1 |
| BP | GO:0050432 | catecholamine secretion | 0.215941039 | 0.448354338 | 0.424269076 | CNR1 | 1 |
| BP | GO:0060997 | dendritic spine morphogenesis | 0.215941039 | 0.448354338 | 0.424269076 | DNM3 | 1 |
| BP | GO:0050807 | regulation of synapse organization | 0.217453938 | 0.450347853 | 0.426155501 | DNM3/NEDD9 | 2 |
| BP | GO:0099111 | microtubule-based transport | 0.217453938 | 0.450347853 | 0.426155501 | SPAG17/DLG2 | 2 |
| BP | GO:0001755 | neural crest cell migration | 0.219227887 | 0.450347853 | 0.426155501 | ISL1 | 1 |
| BP | GO:0002090 | regulation of receptor internalization | 0.219227887 | 0.450347853 | 0.426155501 | PLCG2 | 1 |
| BP | GO:0002763 | positive regulation of myeloid leukocyte differentiation | 0.219227887 | 0.450347853 | 0.426155501 | NEDD9 | 1 |
| BP | GO:0010574 | regulation of vascular endothelial growth factor production | 0.219227887 | 0.450347853 | 0.426155501 | ISL1 | 1 |
| BP | GO:0043666 | regulation of phosphoprotein phosphatase activity | 0.219227887 | 0.450347853 | 0.426155501 | GNA12 | 1 |
| BP | GO:0045010 | actin nucleation | 0.219227887 | 0.450347853 | 0.426155501 | ARPC1B | 1 |
| BP | GO:0072666 | establishment of protein localization to vacuole | 0.219227887 | 0.450347853 | 0.426155501 | MON1B | 1 |
| BP | GO:0006282 | regulation of DNA repair | 0.222061095 | 0.451218823 | 0.426979683 | EYA1/SLF2 | 2 |
| BP | GO:0001658 | branching involved in ureteric bud morphogenesis | 0.222501131 | 0.451218823 | 0.426979683 | EYA1 | 1 |
| BP | GO:0022029 | telencephalon cell migration | 0.222501131 | 0.451218823 | 0.426979683 | ROBO1 | 1 |
| BP | GO:0033866 | nucleoside bisphosphate biosynthetic process | 0.222501131 | 0.451218823 | 0.426979683 | PDHA2 | 1 |
| BP | GO:0034030 | ribonucleoside bisphosphate biosynthetic process | 0.222501131 | 0.451218823 | 0.426979683 | PDHA2 | 1 |
| BP | GO:0034033 | purine nucleoside bisphosphate biosynthetic process | 0.222501131 | 0.451218823 | 0.426979683 | PDHA2 | 1 |
| BP | GO:0051353 | positive regulation of oxidoreductase activity | 0.222501131 | 0.451218823 | 0.426979683 | FXN | 1 |
| BP | GO:0055008 | cardiac muscle tissue morphogenesis | 0.222501131 | 0.451218823 | 0.426979683 | ISL1 | 1 |
| BP | GO:1905517 | macrophage migration | 0.222501131 | 0.451218823 | 0.426979683 | TNFSF18 | 1 |
| BP | GO:2000300 | regulation of synaptic vesicle exocytosis | 0.222501131 | 0.451218823 | 0.426979683 | CNR1 | 1 |
| BP | GO:2000351 | regulation of endothelial cell apoptotic process | 0.222501131 | 0.451218823 | 0.426979683 | FASLG | 1 |
| BP | GO:0046474 | glycerophospholipid biosynthetic process | 0.225137202 | 0.454653591 | 0.430229937 | GPAM/PLCG2 | 2 |
| BP | GO:0044458 | motile cilium assembly | 0.225760824 | 0.454653591 | 0.430229937 | SPAG17 | 1 |
| BP | GO:0060135 | maternal process involved in female pregnancy | 0.225760824 | 0.454653591 | 0.430229937 | CNR1 | 1 |
| BP | GO:0090497 | mesenchymal cell migration | 0.225760824 | 0.454653591 | 0.430229937 | ISL1 | 1 |
| BP | GO:0098900 | regulation of action potential | 0.225760824 | 0.454653591 | 0.430229937 | CNR1 | 1 |
| BP | GO:1900024 | regulation of substrate adhesion-dependent cell spreading | 0.225760824 | 0.454653591 | 0.430229937 | NEDD9 | 1 |
| BP | GO:0050803 | regulation of synapse structure or activity | 0.226676554 | 0.455587506 | 0.431113683 | DNM3/NEDD9 | 2 |
| BP | GO:0009100 | glycoprotein metabolic process | 0.228662175 | 0.455587506 | 0.431113683 | ALG6/XXYLT1/ST8SIA1 | 3 |
| BP | GO:0002820 | negative regulation of adaptive immune response | 0.229007024 | 0.455587506 | 0.431113683 | TNFSF18 | 1 |
| BP | GO:0006687 | glycosphingolipid metabolic process | 0.229007024 | 0.455587506 | 0.431113683 | ST8SIA1 | 1 |
| BP | GO:0031102 | neuron projection regeneration | 0.229007024 | 0.455587506 | 0.431113683 | ISL1 | 1 |
| BP | GO:0060393 | regulation of pathway-restricted SMAD protein phosphorylation | 0.229007024 | 0.455587506 | 0.431113683 | BMP3 | 1 |
| BP | GO:0070228 | regulation of lymphocyte apoptotic process | 0.229007024 | 0.455587506 | 0.431113683 | GPAM | 1 |
| BP | GO:1903409 | reactive oxygen species biosynthetic process | 0.229007024 | 0.455587506 | 0.431113683 | PLCG2 | 1 |
| BP | GO:0002253 | activation of immune response | 0.230870357 | 0.455587506 | 0.431113683 | FYB1/VAV2/PLCG2 | 3 |
| BP | GO:0043523 | regulation of neuron apoptotic process | 0.231299403 | 0.455587506 | 0.431113683 | FASLG/ISL1 | 2 |
| BP | GO:0007062 | sister chromatid cohesion | 0.232239785 | 0.455587506 | 0.431113683 | SLF2 | 1 |
| BP | GO:0010573 | vascular endothelial growth factor production | 0.232239785 | 0.455587506 | 0.431113683 | ISL1 | 1 |
| BP | GO:0021885 | forebrain cell migration | 0.232239785 | 0.455587506 | 0.431113683 | ROBO1 | 1 |
| BP | GO:0031663 | lipopolysaccharide-mediated signaling pathway | 0.232239785 | 0.455587506 | 0.431113683 | PLCG2 | 1 |
| BP | GO:0032481 | positive regulation of type I interferon production | 0.232239785 | 0.455587506 | 0.431113683 | PLCG2 | 1 |
| BP | GO:0032623 | interleukin-2 production | 0.232239785 | 0.455587506 | 0.431113683 | PLCG2 | 1 |
| BP | GO:0032663 | regulation of interleukin-2 production | 0.232239785 | 0.455587506 | 0.431113683 | PLCG2 | 1 |
| BP | GO:0032731 | positive regulation of interleukin-1 beta production | 0.232239785 | 0.455587506 | 0.431113683 | ISL1 | 1 |
| BP | GO:0046173 | polyol biosynthetic process | 0.232239785 | 0.455587506 | 0.431113683 | PLCG2 | 1 |
| BP | GO:0046579 | positive regulation of Ras protein signal transduction | 0.232239785 | 0.455587506 | 0.431113683 | ROBO1 | 1 |
| BP | GO:0050994 | regulation of lipid catabolic process | 0.232239785 | 0.455587506 | 0.431113683 | CNR1 | 1 |
| BP | GO:0060042 | retina morphogenesis in camera-type eye | 0.232239785 | 0.455587506 | 0.431113683 | ATP8A2 | 1 |
| BP | GO:0099072 | regulation of postsynaptic membrane neurotransmitter receptor levels | 0.232239785 | 0.455587506 | 0.431113683 | DNM3 | 1 |
| BP | GO:0006631 | fatty acid metabolic process | 0.234190348 | 0.457268516 | 0.432704391 | PLA2G2F/CNR1/GPAM | 3 |
| BP | GO:0002218 | activation of innate immune response | 0.235459161 | 0.457268516 | 0.432704391 | PLCG2 | 1 |
| BP | GO:0009311 | oligosaccharide metabolic process | 0.235459161 | 0.457268516 | 0.432704391 | ST8SIA1 | 1 |
| BP | GO:0010972 | negative regulation of G2/M transition of mitotic cell cycle | 0.235459161 | 0.457268516 | 0.432704391 | FOXN3 | 1 |
| BP | GO:0035306 | positive regulation of dephosphorylation | 0.235459161 | 0.457268516 | 0.432704391 | GNA12 | 1 |
| BP | GO:0045143 | homologous chromosome segregation | 0.235459161 | 0.457268516 | 0.432704391 | MEI4 | 1 |
| BP | GO:0046530 | photoreceptor cell differentiation | 0.235459161 | 0.457268516 | 0.432704391 | TULP1 | 1 |
| BP | GO:0071384 | cellular response to corticosteroid stimulus | 0.235459161 | 0.457268516 | 0.432704391 | ISL1 | 1 |
| BP | GO:0098930 | axonal transport | 0.235459161 | 0.457268516 | 0.432704391 | DLG2 | 1 |
| BP | GO:0043583 | ear development | 0.235928752 | 0.457670253 | 0.433084546 | EYA1/ATP8A2 | 2 |
| BP | GO:0001885 | endothelial cell development | 0.238665209 | 0.458890501 | 0.434239244 | TJP2 | 1 |
| BP | GO:0003170 | heart valve development | 0.238665209 | 0.458890501 | 0.434239244 | ROBO1 | 1 |
| BP | GO:0032613 | interleukin-10 production | 0.238665209 | 0.458890501 | 0.434239244 | PLCG2 | 1 |
| BP | GO:0032653 | regulation of interleukin-10 production | 0.238665209 | 0.458890501 | 0.434239244 | PLCG2 | 1 |
| BP | GO:0033013 | tetrapyrrole metabolic process | 0.238665209 | 0.458890501 | 0.434239244 | FXN | 1 |
| BP | GO:0035065 | regulation of histone acetylation | 0.238665209 | 0.458890501 | 0.434239244 | ISL1 | 1 |
| BP | GO:0048247 | lymphocyte chemotaxis | 0.238665209 | 0.458890501 | 0.434239244 | NEDD9 | 1 |
| BP | GO:1905515 | non-motile cilium assembly | 0.238665209 | 0.458890501 | 0.434239244 | TBC1D32 | 1 |
| BP | GO:0071805 | potassium ion transmembrane transport | 0.239018166 | 0.458928533 | 0.434275233 | SLC9C2/KCNQ3 | 2 |
| BP | GO:0050679 | positive regulation of epithelial cell proliferation | 0.240563732 | 0.458928533 | 0.434275233 | EYA1/ST8SIA1 | 2 |
| BP | GO:0097191 | extrinsic apoptotic signaling pathway | 0.240563732 | 0.458928533 | 0.434275233 | FASLG/EYA1 | 2 |
| BP | GO:0032970 | regulation of actin filament-based process | 0.240856802 | 0.458928533 | 0.434275233 | RICTOR/NEDD9/ARPC1B | 3 |
| BP | GO:0006879 | cellular iron ion homeostasis | 0.241857981 | 0.458928533 | 0.434275233 | FXN | 1 |
| BP | GO:0034605 | cellular response to heat | 0.241857981 | 0.458928533 | 0.434275233 | ST8SIA1 | 1 |
| BP | GO:0048278 | vesicle docking | 0.241857981 | 0.458928533 | 0.434275233 | STX6 | 1 |
| BP | GO:0060389 | pathway-restricted SMAD protein phosphorylation | 0.241857981 | 0.458928533 | 0.434275233 | BMP3 | 1 |
| BP | GO:0060675 | ureteric bud morphogenesis | 0.241857981 | 0.458928533 | 0.434275233 | EYA1 | 1 |
| BP | GO:0072577 | endothelial cell apoptotic process | 0.241857981 | 0.458928533 | 0.434275233 | FASLG | 1 |
| BP | GO:1902750 | negative regulation of cell cycle G2/M phase transition | 0.241857981 | 0.458928533 | 0.434275233 | FOXN3 | 1 |
| BP | GO:1903051 | negative regulation of proteolysis involved in protein catabolic process | 0.241857981 | 0.458928533 | 0.434275233 | FHIT | 1 |
| BP | GO:0030072 | peptide hormone secretion | 0.242109829 | 0.458928533 | 0.434275233 | ISL1/CNR1 | 2 |
| BP | GO:0051651 | maintenance of location in cell | 0.243656426 | 0.460781535 | 0.436028693 | FASLG/PLCG2 | 2 |
| BP | GO:0006303 | double-strand break repair via nonhomologous end joining | 0.245037533 | 0.460781535 | 0.436028693 | SMARCAL1 | 1 |
| BP | GO:0009620 | response to fungus | 0.245037533 | 0.460781535 | 0.436028693 | PLCG2 | 1 |
| BP | GO:0015909 | long-chain fatty acid transport | 0.245037533 | 0.460781535 | 0.436028693 | PLA2G2F | 1 |
| BP | GO:0043954 | cellular component maintenance | 0.245037533 | 0.460781535 | 0.436028693 | NEDD9 | 1 |
| BP | GO:0045576 | mast cell activation | 0.245037533 | 0.460781535 | 0.436028693 | CNR1 | 1 |
| BP | GO:0072171 | mesonephric tubule morphogenesis | 0.245037533 | 0.460781535 | 0.436028693 | EYA1 | 1 |
| BP | GO:1903050 | regulation of proteolysis involved in protein catabolic process | 0.245203492 | 0.460781535 | 0.436028693 | FHIT/GNA12 | 2 |
| BP | GO:0032024 | positive regulation of insulin secretion | 0.248203919 | 0.461953119 | 0.43713734 | ISL1 | 1 |
| BP | GO:0038034 | signal transduction in absence of ligand | 0.248203919 | 0.461953119 | 0.43713734 | EYA1 | 1 |
| BP | GO:0042093 | T-helper cell differentiation | 0.248203919 | 0.461953119 | 0.43713734 | TNFSF18 | 1 |
| BP | GO:0043550 | regulation of lipid kinase activity | 0.248203919 | 0.461953119 | 0.43713734 | VAV2 | 1 |
| BP | GO:0045670 | regulation of osteoclast differentiation | 0.248203919 | 0.461953119 | 0.43713734 | NEDD9 | 1 |
| BP | GO:0097192 | extrinsic apoptotic signaling pathway in absence of ligand | 0.248203919 | 0.461953119 | 0.43713734 | EYA1 | 1 |
| BP | GO:1902808 | positive regulation of cell cycle G1/S phase transition | 0.248203919 | 0.461953119 | 0.43713734 | PLCG2 | 1 |
| BP | GO:0071241 | cellular response to inorganic substance | 0.24829891 | 0.461953119 | 0.43713734 | DLG2/PLCG2 | 2 |
| BP | GO:0071902 | positive regulation of protein serine/threonine kinase activity | 0.24829891 | 0.461953119 | 0.43713734 | ROBO1/RICTOR | 2 |
| BP | GO:0007159 | leukocyte cell-cell adhesion | 0.249796142 | 0.461953119 | 0.43713734 | PLA2G2F/TNFSF18/GPAM | 3 |
| BP | GO:0018205 | peptidyl-lysine modification | 0.249796142 | 0.461953119 | 0.43713734 | ISL1/EYA1/SLF2 | 3 |
| BP | GO:0002790 | peptide secretion | 0.249847202 | 0.461953119 | 0.43713734 | ISL1/CNR1 | 2 |
| BP | GO:0007018 | microtubule-based movement | 0.250917344 | 0.461953119 | 0.43713734 | SPAG17/DNAH8/DLG2 | 3 |
| BP | GO:0002260 | lymphocyte homeostasis | 0.251357191 | 0.461953119 | 0.43713734 | GPAM | 1 |
| BP | GO:0035418 | protein localization to synapse | 0.251357191 | 0.461953119 | 0.43713734 | DLG2 | 1 |
| BP | GO:0046847 | filopodium assembly | 0.251357191 | 0.461953119 | 0.43713734 | DNM3 | 1 |
| BP | GO:0050922 | negative regulation of chemotaxis | 0.251357191 | 0.461953119 | 0.43713734 | ROBO1 | 1 |
| BP | GO:0061180 | mammary gland epithelium development | 0.251357191 | 0.461953119 | 0.43713734 | ROBO1 | 1 |
| BP | GO:2000401 | regulation of lymphocyte migration | 0.251357191 | 0.461953119 | 0.43713734 | NEDD9 | 1 |
| BP | GO:0002685 | regulation of leukocyte migration | 0.251395842 | 0.461953119 | 0.43713734 | TNFSF18/NEDD9 | 2 |
| BP | GO:0031669 | cellular response to nutrient levels | 0.251395842 | 0.461953119 | 0.43713734 | RICTOR/MAX | 2 |
| BP | GO:2001234 | negative regulation of apoptotic signaling pathway | 0.254494051 | 0.464097286 | 0.439166324 | EYA1/FXN | 2 |
| BP | GO:0002294 | CD4-positive, alpha-beta T cell differentiation involved in immune response | 0.254497405 | 0.464097286 | 0.439166324 | TNFSF18 | 1 |
| BP | GO:0030193 | regulation of blood coagulation | 0.254497405 | 0.464097286 | 0.439166324 | ADAMTS18 | 1 |
| BP | GO:0042130 | negative regulation of T cell proliferation | 0.254497405 | 0.464097286 | 0.439166324 | PLA2G2F | 1 |
| BP | GO:0043627 | response to estrogen | 0.254497405 | 0.464097286 | 0.439166324 | ARPC1B | 1 |
| BP | GO:0030098 | lymphocyte differentiation | 0.255410008 | 0.464097286 | 0.439166324 | TNFSF18/DNAJB9/PLCG2 | 3 |
| BP | GO:0040013 | negative regulation of locomotion | 0.255410008 | 0.464097286 | 0.439166324 | ROBO1/NEDD9/GNA12 | 3 |
| BP | GO:0007163 | establishment or maintenance of cell polarity | 0.256043561 | 0.464097286 | 0.439166324 | RICTOR/DLG2 | 2 |
| BP | GO:0046883 | regulation of hormone secretion | 0.256043561 | 0.464097286 | 0.439166324 | ISL1/CNR1 | 2 |
| BP | GO:0071356 | cellular response to tumor necrosis factor | 0.256043561 | 0.464097286 | 0.439166324 | TNFSF18/ST18 | 2 |
| BP | GO:0002287 | alpha-beta T cell activation involved in immune response | 0.257624613 | 0.464097286 | 0.439166324 | TNFSF18 | 1 |
| BP | GO:0002293 | alpha-beta T cell differentiation involved in immune response | 0.257624613 | 0.464097286 | 0.439166324 | TNFSF18 | 1 |
| BP | GO:0002548 | monocyte chemotaxis | 0.257624613 | 0.464097286 | 0.439166324 | TNFSF18 | 1 |
| BP | GO:0003208 | cardiac ventricle morphogenesis | 0.257624613 | 0.464097286 | 0.439166324 | ISL1 | 1 |
| BP | GO:0006487 | protein N-linked glycosylation | 0.257624613 | 0.464097286 | 0.439166324 | ALG6 | 1 |
| BP | GO:0009988 | cell-cell recognition | 0.257624613 | 0.464097286 | 0.439166324 | NEDD9 | 1 |
| BP | GO:0051057 | positive regulation of small GTPase mediated signal transduction | 0.257624613 | 0.464097286 | 0.439166324 | ROBO1 | 1 |
| BP | GO:0070527 | platelet aggregation | 0.257624613 | 0.464097286 | 0.439166324 | ADAMTS18 | 1 |
| BP | GO:2000379 | positive regulation of reactive oxygen species metabolic process | 0.257624613 | 0.464097286 | 0.439166324 | PLCG2 | 1 |
| BP | GO:0060537 | muscle tissue development | 0.258787398 | 0.46571038 | 0.440692764 | ISL1/EYA1/SGCG | 3 |
| BP | GO:0002274 | myeloid leukocyte activation | 0.260693369 | 0.466331733 | 0.441280738 | CNR1/PLCG2 | 2 |
| BP | GO:0016358 | dendrite development | 0.260693369 | 0.466331733 | 0.441280738 | DNM3/TULP1 | 2 |
| BP | GO:0009247 | glycolipid biosynthetic process | 0.260738868 | 0.466331733 | 0.441280738 | ST8SIA1 | 1 |
| BP | GO:0033143 | regulation of intracellular steroid hormone receptor signaling pathway | 0.260738868 | 0.466331733 | 0.441280738 | ISL1 | 1 |
| BP | GO:0070265 | necrotic cell death | 0.260738868 | 0.466331733 | 0.441280738 | FASLG | 1 |
| BP | GO:1900046 | regulation of hemostasis | 0.260738868 | 0.466331733 | 0.441280738 | ADAMTS18 | 1 |
| BP | GO:0030032 | lamellipodium assembly | 0.263840224 | 0.468511386 | 0.443343302 | VAV2 | 1 |
| BP | GO:0045665 | negative regulation of neuron differentiation | 0.263840224 | 0.468511386 | 0.443343302 | ISL1 | 1 |
| BP | GO:0046637 | regulation of alpha-beta T cell differentiation | 0.263840224 | 0.468511386 | 0.443343302 | TNFSF18 | 1 |
| BP | GO:0050795 | regulation of behavior | 0.263840224 | 0.468511386 | 0.443343302 | CNR1 | 1 |
| BP | GO:0051937 | catecholamine transport | 0.263840224 | 0.468511386 | 0.443343302 | CNR1 | 1 |
| BP | GO:0060415 | muscle tissue morphogenesis | 0.263840224 | 0.468511386 | 0.443343302 | ISL1 | 1 |
| BP | GO:0150076 | neuroinflammatory response | 0.263840224 | 0.468511386 | 0.443343302 | PLCG2 | 1 |
| BP | GO:0030595 | leukocyte chemotaxis | 0.265344498 | 0.469212767 | 0.444007005 | TNFSF18/NEDD9 | 2 |
| BP | GO:0045333 | cellular respiration | 0.265344498 | 0.469212767 | 0.444007005 | PDHA2/FXN | 2 |
| BP | GO:0048738 | cardiac muscle tissue development | 0.266895039 | 0.469212767 | 0.444007005 | ISL1/SGCG | 2 |
| BP | GO:0001707 | mesoderm formation | 0.266928733 | 0.469212767 | 0.444007005 | EYA1 | 1 |
| BP | GO:0003281 | ventricular septum development | 0.266928733 | 0.469212767 | 0.444007005 | ROBO1 | 1 |
| BP | GO:0032732 | positive regulation of interleukin-1 production | 0.266928733 | 0.469212767 | 0.444007005 | ISL1 | 1 |
| BP | GO:0035924 | cellular response to vascular endothelial growth factor stimulus | 0.266928733 | 0.469212767 | 0.444007005 | ROBO1 | 1 |
| BP | GO:0042058 | regulation of epidermal growth factor receptor signaling pathway | 0.266928733 | 0.469212767 | 0.444007005 | FASLG | 1 |
| BP | GO:0048662 | negative regulation of smooth muscle cell proliferation | 0.266928733 | 0.469212767 | 0.444007005 | GNA12 | 1 |
| BP | GO:2000756 | regulation of peptidyl-lysine acetylation | 0.266928733 | 0.469212767 | 0.444007005 | ISL1 | 1 |
| BP | GO:0022412 | cellular process involved in reproduction in multicellular organism | 0.267824087 | 0.470312057 | 0.445047242 | SPAG17/MEI4/ETV6 | 3 |
| BP | GO:0046395 | carboxylic acid catabolic process | 0.269996202 | 0.473186868 | 0.447767621 | BCKDHB/CNR1 | 2 |
| BP | GO:0050818 | regulation of coagulation | 0.270004447 | 0.473186868 | 0.447767621 | ADAMTS18 | 1 |
| BP | GO:0006869 | lipid transport | 0.271223261 | 0.474260663 | 0.448783732 | PLA2G2F/FASLG/ATP8A2 | 3 |
| BP | GO:0006979 | response to oxidative stress | 0.272357484 | 0.474260663 | 0.448783732 | PRDX6/FXN/ATRN | 3 |
| BP | GO:0002637 | regulation of immunoglobulin production | 0.27306742 | 0.474260663 | 0.448783732 | DNAJB9 | 1 |
| BP | GO:0006635 | fatty acid beta-oxidation | 0.27306742 | 0.474260663 | 0.448783732 | CNR1 | 1 |
| BP | GO:0009166 | nucleotide catabolic process | 0.27306742 | 0.474260663 | 0.448783732 | FHIT | 1 |
| BP | GO:0021536 | diencephalon development | 0.27306742 | 0.474260663 | 0.448783732 | ISL1 | 1 |
| BP | GO:0048332 | mesoderm morphogenesis | 0.27306742 | 0.474260663 | 0.448783732 | EYA1 | 1 |
| BP | GO:0051339 | regulation of lyase activity | 0.27306742 | 0.474260663 | 0.448783732 | FXN | 1 |
| BP | GO:0070192 | chromosome organization involved in meiotic cell cycle | 0.27306742 | 0.474260663 | 0.448783732 | MEI4 | 1 |
| BP | GO:0032102 | negative regulation of response to external stimulus | 0.274627603 | 0.475431918 | 0.449892068 | ROBO1/ISL1/ADAMTS18 | 3 |
| BP | GO:0008088 | axo-dendritic transport | 0.276117703 | 0.475431918 | 0.449892068 | DLG2 | 1 |
| BP | GO:0030968 | endoplasmic reticulum unfolded protein response | 0.276117703 | 0.475431918 | 0.449892068 | DNAJB9 | 1 |
| BP | GO:0032729 | positive regulation of interferon-gamma production | 0.276117703 | 0.475431918 | 0.449892068 | ISL1 | 1 |
| BP | GO:0090559 | regulation of membrane permeability | 0.276117703 | 0.475431918 | 0.449892068 | TJP2 | 1 |
| BP | GO:0006813 | potassium ion transport | 0.276198106 | 0.475431918 | 0.449892068 | SLC9C2/KCNQ3 | 2 |
| BP | GO:0016054 | organic acid catabolic process | 0.276198106 | 0.475431918 | 0.449892068 | BCKDHB/CNR1 | 2 |
| BP | GO:0016197 | endosomal transport | 0.276198106 | 0.475431918 | 0.449892068 | STX6/VPS53 | 2 |
| BP | GO:0031330 | negative regulation of cellular catabolic process | 0.276198106 | 0.475431918 | 0.449892068 | FHIT/CNR1 | 2 |
| BP | GO:0048863 | stem cell differentiation | 0.277748341 | 0.47762844 | 0.451970595 | ISL1/SHC4 | 2 |
| BP | GO:0050772 | positive regulation of axonogenesis | 0.279155347 | 0.478159897 | 0.452473502 | ROBO1 | 1 |
| BP | GO:0051966 | regulation of synaptic transmission, glutamatergic | 0.279155347 | 0.478159897 | 0.452473502 | CNR1 | 1 |
| BP | GO:0061045 | negative regulation of wound healing | 0.279155347 | 0.478159897 | 0.452473502 | ADAMTS18 | 1 |
| BP | GO:0072665 | protein localization to vacuole | 0.279155347 | 0.478159897 | 0.452473502 | MON1B | 1 |
| BP | GO:0060047 | heart contraction | 0.28084834 | 0.479569041 | 0.453806948 | ISL1/SGCG | 2 |
| BP | GO:0002292 | T cell differentiation involved in immune response | 0.282180406 | 0.479569041 | 0.453806948 | TNFSF18 | 1 |
| BP | GO:0002437 | inflammatory response to antigenic stimulus | 0.282180406 | 0.479569041 | 0.453806948 | CNR1 | 1 |
| BP | GO:0002720 | positive regulation of cytokine production involved in immune response | 0.282180406 | 0.479569041 | 0.453806948 | PLCG2 | 1 |
| BP | GO:0003407 | neural retina development | 0.282180406 | 0.479569041 | 0.453806948 | ATP8A2 | 1 |
| BP | GO:0007193 | adenylate cyclase-inhibiting G protein-coupled receptor signaling pathway | 0.282180406 | 0.479569041 | 0.453806948 | HTR1E | 1 |
| BP | GO:0048844 | artery morphogenesis | 0.282180406 | 0.479569041 | 0.453806948 | EYA1 | 1 |
| BP | GO:2000514 | regulation of CD4-positive, alpha-beta T cell activation | 0.282180406 | 0.479569041 | 0.453806948 | TNFSF18 | 1 |
| BP | GO:0050851 | antigen receptor-mediated signaling pathway | 0.283947542 | 0.481401244 | 0.455540726 | FYB1/PLCG2 | 2 |
| BP | GO:0001895 | retina homeostasis | 0.28519293 | 0.481401244 | 0.455540726 | TULP1 | 1 |
| BP | GO:0008344 | adult locomotory behavior | 0.28519293 | 0.481401244 | 0.455540726 | FXN | 1 |
| BP | GO:0042440 | pigment metabolic process | 0.28519293 | 0.481401244 | 0.455540726 | FXN | 1 |
| BP | GO:0048644 | muscle organ morphogenesis | 0.28519293 | 0.481401244 | 0.455540726 | ISL1 | 1 |
| BP | GO:1901184 | regulation of ERBB signaling pathway | 0.28519293 | 0.481401244 | 0.455540726 | FASLG | 1 |
| BP | GO:2001259 | positive regulation of cation channel activity | 0.28519293 | 0.481401244 | 0.455540726 | PLCG2 | 1 |
| BP | GO:0015833 | peptide transport | 0.287045747 | 0.482931287 | 0.456988577 | ISL1/CNR1 | 2 |
| BP | GO:0034612 | response to tumor necrosis factor | 0.287045747 | 0.482931287 | 0.456988577 | TNFSF18/ST18 | 2 |
| BP | GO:0045017 | glycerolipid biosynthetic process | 0.287045747 | 0.482931287 | 0.456988577 | GPAM/PLCG2 | 2 |
| BP | GO:0007422 | peripheral nervous system development | 0.28819297 | 0.482931287 | 0.456988577 | ISL1 | 1 |
| BP | GO:0044773 | mitotic DNA damage checkpoint signaling | 0.28819297 | 0.482931287 | 0.456988577 | FOXN3 | 1 |
| BP | GO:0072332 | intrinsic apoptotic signaling pathway by p53 class mediator | 0.28819297 | 0.482931287 | 0.456988577 | FHIT | 1 |
| BP | GO:1901657 | glycosyl compound metabolic process | 0.28819297 | 0.482931287 | 0.456988577 | ABHD10 | 1 |
| BP | GO:0021537 | telencephalon development | 0.288594414 | 0.482931287 | 0.456988577 | ROBO1/DCLK2 | 2 |
| BP | GO:0043122 | regulation of I-kappaB kinase/NF-kappaB signaling | 0.288594414 | 0.482931287 | 0.456988577 | FASLG/PLCG2 | 2 |
| BP | GO:0043161 | proteasome-mediated ubiquitin-dependent protein catabolic process | 0.290573378 | 0.48439018 | 0.458369099 | FHIT/GNA12/DNAJB9 | 3 |
| BP | GO:0010770 | positive regulation of cell morphogenesis involved in differentiation | 0.291180578 | 0.48439018 | 0.458369099 | NEDD9 | 1 |
| BP | GO:0021766 | hippocampus development | 0.291180578 | 0.48439018 | 0.458369099 | DCLK2 | 1 |
| BP | GO:0031016 | pancreas development | 0.291180578 | 0.48439018 | 0.458369099 | ISL1 | 1 |
| BP | GO:0060395 | SMAD protein signal transduction | 0.291180578 | 0.48439018 | 0.458369099 | BMP3 | 1 |
| BP | GO:1905897 | regulation of response to endoplasmic reticulum stress | 0.291180578 | 0.48439018 | 0.458369099 | DNAJB9 | 1 |
| BP | GO:0006898 | receptor-mediated endocytosis | 0.291690757 | 0.48439018 | 0.458369099 | DNM3/PLCG2 | 2 |
| BP | GO:0050708 | regulation of protein secretion | 0.291690757 | 0.48439018 | 0.458369099 | ISL1/CNR1 | 2 |
| BP | GO:0009314 | response to radiation | 0.292858069 | 0.485865481 | 0.459765148 | TULP1/EYA1/ATP8A2 | 3 |
| BP | GO:0014032 | neural crest cell development | 0.294155804 | 0.486628121 | 0.460486819 | ISL1 | 1 |
| BP | GO:0050848 | regulation of calcium-mediated signaling | 0.294155804 | 0.486628121 | 0.460486819 | PLCG2 | 1 |
| BP | GO:0055013 | cardiac muscle cell development | 0.294155804 | 0.486628121 | 0.460486819 | ISL1 | 1 |
| BP | GO:0008654 | phospholipid biosynthetic process | 0.294785617 | 0.487207348 | 0.46103493 | GPAM/PLCG2 | 2 |
| BP | GO:0007015 | actin filament organization | 0.295144162 | 0.487337565 | 0.461158153 | RICTOR/NEDD9/ARPC1B | 3 |
| BP | GO:0090068 | positive regulation of cell cycle process | 0.296332432 | 0.487692556 | 0.461494073 | SLF2/PLCG2 | 2 |
| BP | GO:0010921 | regulation of phosphatase activity | 0.2971187 | 0.487692556 | 0.461494073 | GNA12 | 1 |
| BP | GO:0015844 | monoamine transport | 0.2971187 | 0.487692556 | 0.461494073 | CNR1 | 1 |
| BP | GO:0021675 | nerve development | 0.2971187 | 0.487692556 | 0.461494073 | ISL1 | 1 |
| BP | GO:0097194 | execution phase of apoptosis | 0.2971187 | 0.487692556 | 0.461494073 | FASLG | 1 |
| BP | GO:0140056 | organelle localization by membrane tethering | 0.2971187 | 0.487692556 | 0.461494073 | STX6 | 1 |
| BP | GO:0002699 | positive regulation of immune effector process | 0.297878805 | 0.487692556 | 0.461494073 | DNAJB9/PLCG2 | 2 |
| BP | GO:0003015 | heart process | 0.297878805 | 0.487692556 | 0.461494073 | ISL1/SGCG | 2 |
| BP | GO:0006470 | protein dephosphorylation | 0.297878805 | 0.487692556 | 0.461494073 | GNA12/EYA1 | 2 |
| BP | GO:0031668 | cellular response to extracellular stimulus | 0.299424715 | 0.488981053 | 0.462713354 | RICTOR/MAX | 2 |
| BP | GO:0008625 | extrinsic apoptotic signaling pathway via death domain receptors | 0.300069315 | 0.488981053 | 0.462713354 | FASLG | 1 |
| BP | GO:0021954 | central nervous system neuron development | 0.300069315 | 0.488981053 | 0.462713354 | DCLK2 | 1 |
| BP | GO:0044774 | mitotic DNA integrity checkpoint signaling | 0.300069315 | 0.488981053 | 0.462713354 | FOXN3 | 1 |
| BP | GO:1901292 | nucleoside phosphate catabolic process | 0.300069315 | 0.488981053 | 0.462713354 | FHIT | 1 |
| BP | GO:0002088 | lens development in camera-type eye | 0.303007699 | 0.492847257 | 0.466371868 | TBC1D32 | 1 |
| BP | GO:0045921 | positive regulation of exocytosis | 0.303007699 | 0.492847257 | 0.466371868 | CNR1 | 1 |
| BP | GO:0051091 | positive regulation of DNA-binding transcription factor activity | 0.305603257 | 0.494834597 | 0.468252449 | TNFSF18/PLCG2 | 2 |
| BP | GO:0001523 | retinoid metabolic process | 0.305933904 | 0.494834597 | 0.468252449 | PLB1 | 1 |
| BP | GO:0014910 | regulation of smooth muscle cell migration | 0.305933904 | 0.494834597 | 0.468252449 | GNA12 | 1 |
| BP | GO:0030433 | ubiquitin-dependent ERAD pathway | 0.305933904 | 0.494834597 | 0.468252449 | DNAJB9 | 1 |
| BP | GO:0050672 | negative regulation of lymphocyte proliferation | 0.305933904 | 0.494834597 | 0.468252449 | PLA2G2F | 1 |
| BP | GO:0071277 | cellular response to calcium ion | 0.305933904 | 0.494834597 | 0.468252449 | PLCG2 | 1 |
| BP | GO:0002312 | B cell activation involved in immune response | 0.308847978 | 0.49632212 | 0.469660064 | PLCG2 | 1 |
| BP | GO:0032945 | negative regulation of mononuclear cell proliferation | 0.308847978 | 0.49632212 | 0.469660064 | PLA2G2F | 1 |
| BP | GO:0035023 | regulation of Rho protein signal transduction | 0.308847978 | 0.49632212 | 0.469660064 | ROBO1 | 1 |
| BP | GO:0043367 | CD4-positive, alpha-beta T cell differentiation | 0.308847978 | 0.49632212 | 0.469660064 | TNFSF18 | 1 |
| BP | GO:0055072 | iron ion homeostasis | 0.308847978 | 0.49632212 | 0.469660064 | FXN | 1 |
| BP | GO:1901983 | regulation of protein acetylation | 0.308847978 | 0.49632212 | 0.469660064 | ISL1 | 1 |
| BP | GO:2000781 | positive regulation of double-strand break repair | 0.308847978 | 0.49632212 | 0.469660064 | SLF2 | 1 |
| BP | GO:0001960 | negative regulation of cytokine-mediated signaling pathway | 0.311749972 | 0.499179985 | 0.472364407 | ROBO1 | 1 |
| BP | GO:0045104 | intermediate filament cytoskeleton organization | 0.311749972 | 0.499179985 | 0.472364407 | ATP8A2 | 1 |
| BP | GO:0048864 | stem cell development | 0.311749972 | 0.499179985 | 0.472364407 | ISL1 | 1 |
| BP | GO:0003018 | vascular process in circulatory system | 0.311772574 | 0.499179985 | 0.472364407 | SH3GL2/TJP2 | 2 |
| BP | GO:0006493 | protein O-linked glycosylation | 0.314639934 | 0.501008012 | 0.474094234 | XXYLT1 | 1 |
| BP | GO:0016101 | diterpenoid metabolic process | 0.314639934 | 0.501008012 | 0.474094234 | PLB1 | 1 |
| BP | GO:0045103 | intermediate filament-based process | 0.314639934 | 0.501008012 | 0.474094234 | ATP8A2 | 1 |
| BP | GO:0051591 | response to cAMP | 0.314639934 | 0.501008012 | 0.474094234 | BCKDHB | 1 |
| BP | GO:0055006 | cardiac cell development | 0.314639934 | 0.501008012 | 0.474094234 | ISL1 | 1 |
| BP | GO:0070098 | chemokine-mediated signaling pathway | 0.314639934 | 0.501008012 | 0.474094234 | ROBO1 | 1 |
| BP | GO:0007611 | learning or memory | 0.316392674 | 0.502832916 | 0.475821105 | NEDD9/CNR1 | 2 |
| BP | GO:1903131 | mononuclear cell differentiation | 0.316914964 | 0.502832916 | 0.475821105 | TNFSF18/DNAJB9/PLCG2 | 3 |
| BP | GO:0046928 | regulation of neurotransmitter secretion | 0.317517915 | 0.502832916 | 0.475821105 | CNR1 | 1 |
| BP | GO:0060021 | roof of mouth development | 0.317517915 | 0.502832916 | 0.475821105 | TBC1D32 | 1 |
| BP | GO:1901216 | positive regulation of neuron death | 0.317517915 | 0.502832916 | 0.475821105 | FASLG | 1 |
| BP | GO:1903351 | cellular response to dopamine | 0.317517915 | 0.502832916 | 0.475821105 | HTR1E | 1 |
| BP | GO:0032273 | positive regulation of protein polymerization | 0.320383962 | 0.505075893 | 0.477943591 | RICTOR | 1 |
| BP | GO:0060996 | dendritic spine development | 0.320383962 | 0.505075893 | 0.477943591 | DNM3 | 1 |
| BP | GO:0090277 | positive regulation of peptide hormone secretion | 0.320383962 | 0.505075893 | 0.477943591 | ISL1 | 1 |
| BP | GO:0097581 | lamellipodium organization | 0.320383962 | 0.505075893 | 0.477943591 | VAV2 | 1 |
| BP | GO:1903350 | response to dopamine | 0.320383962 | 0.505075893 | 0.477943591 | HTR1E | 1 |
| BP | GO:0022406 | membrane docking | 0.323238125 | 0.507280013 | 0.480029307 | STX6 | 1 |
| BP | GO:0070301 | cellular response to hydrogen peroxide | 0.323238125 | 0.507280013 | 0.480029307 | FXN | 1 |
| BP | GO:1904427 | positive regulation of calcium ion transmembrane transport | 0.323238125 | 0.507280013 | 0.480029307 | PLCG2 | 1 |
| BP | GO:1904705 | regulation of vascular associated smooth muscle cell proliferation | 0.323238125 | 0.507280013 | 0.480029307 | GNA12 | 1 |
| BP | GO:2000106 | regulation of leukocyte apoptotic process | 0.323238125 | 0.507280013 | 0.480029307 | GPAM | 1 |
| BP | GO:0110053 | regulation of actin filament organization | 0.324078042 | 0.507683408 | 0.480411032 | RICTOR/ARPC1B | 2 |
| BP | GO:1903532 | positive regulation of secretion by cell | 0.324078042 | 0.507683408 | 0.480411032 | ISL1/CNR1 | 2 |
| BP | GO:0002042 | cell migration involved in sprouting angiogenesis | 0.326080453 | 0.508078846 | 0.480785227 | ROBO1 | 1 |
| BP | GO:0002793 | positive regulation of peptide secretion | 0.326080453 | 0.508078846 | 0.480785227 | ISL1 | 1 |
| BP | GO:0009953 | dorsal/ventral pattern formation | 0.326080453 | 0.508078846 | 0.480785227 | TBC1D32 | 1 |
| BP | GO:0035304 | regulation of protein dephosphorylation | 0.326080453 | 0.508078846 | 0.480785227 | GNA12 | 1 |
| BP | GO:0060761 | negative regulation of response to cytokine stimulus | 0.326080453 | 0.508078846 | 0.480785227 | ROBO1 | 1 |
| BP | GO:1903035 | negative regulation of response to wounding | 0.326080453 | 0.508078846 | 0.480785227 | ADAMTS18 | 1 |
| BP | GO:0010876 | lipid localization | 0.328395482 | 0.510207437 | 0.482799472 | PLA2G2F/FASLG/ATP8A2 | 3 |
| BP | GO:0051258 | protein polymerization | 0.328679493 | 0.510207437 | 0.482799472 | RICTOR/ARPC1B | 2 |
| BP | GO:0014909 | smooth muscle cell migration | 0.328910994 | 0.510207437 | 0.482799472 | GNA12 | 1 |
| BP | GO:1903510 | mucopolysaccharide metabolic process | 0.328910994 | 0.510207437 | 0.482799472 | ITIH2 | 1 |
| BP | GO:1990874 | vascular associated smooth muscle cell proliferation | 0.328910994 | 0.510207437 | 0.482799472 | GNA12 | 1 |
| BP | GO:0045785 | positive regulation of cell adhesion | 0.329543797 | 0.510734248 | 0.483297983 | TNFSF18/NEDD9/GPAM | 3 |
| BP | GO:0002275 | myeloid cell activation involved in immune response | 0.331729796 | 0.510960063 | 0.483511667 | PLCG2 | 1 |
| BP | GO:0014033 | neural crest cell differentiation | 0.331729796 | 0.510960063 | 0.483511667 | ISL1 | 1 |
| BP | GO:0019217 | regulation of fatty acid metabolic process | 0.331729796 | 0.510960063 | 0.483511667 | CNR1 | 1 |
| BP | GO:0045807 | positive regulation of endocytosis | 0.331729796 | 0.510960063 | 0.483511667 | PLCG2 | 1 |
| BP | GO:0046330 | positive regulation of JNK cascade | 0.331729796 | 0.510960063 | 0.483511667 | GADD45G | 1 |
| BP | GO:0070664 | negative regulation of leukocyte proliferation | 0.331729796 | 0.510960063 | 0.483511667 | PLA2G2F | 1 |
| BP | GO:0043547 | positive regulation of GTPase activity | 0.331742727 | 0.510960063 | 0.483511667 | NEDD9/RGS6 | 2 |
| BP | GO:0001657 | ureteric bud development | 0.334536908 | 0.514354187 | 0.486723462 | EYA1 | 1 |
| BP | GO:0008593 | regulation of Notch signaling pathway | 0.334536908 | 0.514354187 | 0.486723462 | ROBO1 | 1 |
| BP | GO:0021510 | spinal cord development | 0.337332377 | 0.515015776 | 0.48734951 | ISL1 | 1 |
| BP | GO:0051952 | regulation of amine transport | 0.337332377 | 0.515015776 | 0.48734951 | CNR1 | 1 |
| BP | GO:0060349 | bone morphogenesis | 0.337332377 | 0.515015776 | 0.48734951 | FOXN3 | 1 |
| BP | GO:0072163 | mesonephric epithelium development | 0.337332377 | 0.515015776 | 0.48734951 | EYA1 | 1 |
| BP | GO:0072164 | mesonephric tubule development | 0.337332377 | 0.515015776 | 0.48734951 | EYA1 | 1 |
| BP | GO:0120162 | positive regulation of cold-induced thermogenesis | 0.337332377 | 0.515015776 | 0.48734951 | GADD45G | 1 |
| BP | GO:1990868 | response to chemokine | 0.337332377 | 0.515015776 | 0.48734951 | ROBO1 | 1 |
| BP | GO:1990869 | cellular response to chemokine | 0.337332377 | 0.515015776 | 0.48734951 | ROBO1 | 1 |
| BP | GO:0007249 | I-kappaB kinase/NF-kappaB signaling | 0.339384304 | 0.517000443 | 0.489227563 | FASLG/PLCG2 | 2 |
| BP | GO:0016049 | cell growth | 0.339877744 | 0.517000443 | 0.489227563 | RICTOR/SH3GL2/FXN | 3 |
| BP | GO:0010389 | regulation of G2/M transition of mitotic cell cycle | 0.34011625 | 0.517000443 | 0.489227563 | FOXN3 | 1 |
| BP | GO:0043279 | response to alkaloid | 0.34011625 | 0.517000443 | 0.489227563 | CNR1 | 1 |
| BP | GO:2001237 | negative regulation of extrinsic apoptotic signaling pathway | 0.34011625 | 0.517000443 | 0.489227563 | EYA1 | 1 |
| BP | GO:0001776 | leukocyte homeostasis | 0.342888576 | 0.518499913 | 0.490646482 | GPAM | 1 |
| BP | GO:0010769 | regulation of cell morphogenesis involved in differentiation | 0.342888576 | 0.518499913 | 0.490646482 | NEDD9 | 1 |
| BP | GO:0019080 | viral gene expression | 0.342888576 | 0.518499913 | 0.490646482 | MON1B | 1 |
| BP | GO:0030316 | osteoclast differentiation | 0.342888576 | 0.518499913 | 0.490646482 | NEDD9 | 1 |
| BP | GO:0034620 | cellular response to unfolded protein | 0.342888576 | 0.518499913 | 0.490646482 | DNAJB9 | 1 |
| BP | GO:0098869 | cellular oxidant detoxification | 0.342888576 | 0.518499913 | 0.490646482 | PRDX6 | 1 |
| BP | GO:0019221 | cytokine-mediated signaling pathway | 0.343321266 | 0.518622961 | 0.49076292 | TNFSF18/ROBO1/ST18 | 3 |
| BP | GO:0046879 | hormone secretion | 0.343957189 | 0.518622961 | 0.49076292 | ISL1/CNR1 | 2 |
| BP | GO:0006721 | terpenoid metabolic process | 0.345649402 | 0.518622961 | 0.49076292 | PLB1 | 1 |
| BP | GO:0009062 | fatty acid catabolic process | 0.345649402 | 0.518622961 | 0.49076292 | CNR1 | 1 |
| BP | GO:0015908 | fatty acid transport | 0.345649402 | 0.518622961 | 0.49076292 | PLA2G2F | 1 |
| BP | GO:0021549 | cerebellum development | 0.345649402 | 0.518622961 | 0.49076292 | ATRN | 1 |
| BP | GO:0032204 | regulation of telomere maintenance | 0.345649402 | 0.518622961 | 0.49076292 | SLF2 | 1 |
| BP | GO:0045132 | meiotic chromosome segregation | 0.345649402 | 0.518622961 | 0.49076292 | MEI4 | 1 |
| BP | GO:0061337 | cardiac conduction | 0.345649402 | 0.518622961 | 0.49076292 | ISL1 | 1 |
| BP | GO:0002366 | leukocyte activation involved in immune response | 0.347000454 | 0.520202058 | 0.49225719 | TNFSF18/PLCG2 | 2 |
| BP | GO:0001823 | mesonephros development | 0.348398774 | 0.520506573 | 0.492545346 | EYA1 | 1 |
| BP | GO:0031058 | positive regulation of histone modification | 0.348398774 | 0.520506573 | 0.492545346 | ISL1 | 1 |
| BP | GO:0035249 | synaptic transmission, glutamatergic | 0.348398774 | 0.520506573 | 0.492545346 | CNR1 | 1 |
| BP | GO:1901019 | regulation of calcium ion transmembrane transporter activity | 0.348398774 | 0.520506573 | 0.492545346 | PLCG2 | 1 |
| BP | GO:0006910 | phagocytosis, recognition | 0.35113674 | 0.522357131 | 0.494296494 | TULP1 | 1 |
| BP | GO:0019233 | sensory perception of pain | 0.35113674 | 0.522357131 | 0.494296494 | CNR1 | 1 |
| BP | GO:0045639 | positive regulation of myeloid cell differentiation | 0.35113674 | 0.522357131 | 0.494296494 | NEDD9 | 1 |
| BP | GO:0046620 | regulation of organ growth | 0.35113674 | 0.522357131 | 0.494296494 | FXN | 1 |
| BP | GO:0048477 | oogenesis | 0.35113674 | 0.522357131 | 0.494296494 | MEI4 | 1 |
| BP | GO:0042886 | amide transport | 0.351557015 | 0.522536109 | 0.494465857 | ISL1/CNR1 | 2 |
| BP | GO:0002263 | cell activation involved in immune response | 0.353073576 | 0.524342856 | 0.496175547 | TNFSF18/PLCG2 | 2 |
| BP | GO:0060291 | long-term synaptic potentiation | 0.353863346 | 0.525068099 | 0.496861831 | SCGN | 1 |
| BP | GO:0006664 | glycolipid metabolic process | 0.356578639 | 0.526406771 | 0.49812859 | ST8SIA1 | 1 |
| BP | GO:0032479 | regulation of type I interferon production | 0.356578639 | 0.526406771 | 0.49812859 | PLCG2 | 1 |
| BP | GO:0032606 | type I interferon production | 0.356578639 | 0.526406771 | 0.49812859 | PLCG2 | 1 |
| BP | GO:0051588 | regulation of neurotransmitter transport | 0.356578639 | 0.526406771 | 0.49812859 | CNR1 | 1 |
| BP | GO:0071868 | cellular response to monoamine stimulus | 0.356578639 | 0.526406771 | 0.49812859 | HTR1E | 1 |
| BP | GO:0071870 | cellular response to catecholamine stimulus | 0.356578639 | 0.526406771 | 0.49812859 | HTR1E | 1 |
| BP | GO:0009914 | hormone transport | 0.357616152 | 0.526826939 | 0.498526187 | ISL1/CNR1 | 2 |
| BP | GO:0051054 | positive regulation of DNA metabolic process | 0.359127919 | 0.526826939 | 0.498526187 | EYA1/SLF2 | 2 |
| BP | GO:0015837 | amine transport | 0.359282666 | 0.526826939 | 0.498526187 | CNR1 | 1 |
| BP | GO:0022600 | digestive system process | 0.359282666 | 0.526826939 | 0.498526187 | TJP2 | 1 |
| BP | GO:0035282 | segmentation | 0.359282666 | 0.526826939 | 0.498526187 | IRX1 | 1 |
| BP | GO:0042102 | positive regulation of T cell proliferation | 0.359282666 | 0.526826939 | 0.498526187 | GPAM | 1 |
| BP | GO:1903509 | liposaccharide metabolic process | 0.359282666 | 0.526826939 | 0.498526187 | ST8SIA1 | 1 |
| BP | GO:2001252 | positive regulation of chromosome organization | 0.359282666 | 0.526826939 | 0.498526187 | SLF2 | 1 |
| BP | GO:0002027 | regulation of heart rate | 0.361975471 | 0.52936056 | 0.500923704 | ISL1 | 1 |
| BP | GO:0010717 | regulation of epithelial to mesenchymal transition | 0.361975471 | 0.52936056 | 0.500923704 | ISL1 | 1 |
| BP | GO:0051047 | positive regulation of secretion | 0.363655721 | 0.52936056 | 0.500923704 | ISL1/CNR1 | 2 |
| BP | GO:0006090 | pyruvate metabolic process | 0.364657102 | 0.52936056 | 0.500923704 | PDHA2 | 1 |
| BP | GO:0008637 | apoptotic mitochondrial changes | 0.364657102 | 0.52936056 | 0.500923704 | FXN | 1 |
| BP | GO:0014812 | muscle cell migration | 0.364657102 | 0.52936056 | 0.500923704 | GNA12 | 1 |
| BP | GO:0034308 | primary alcohol metabolic process | 0.364657102 | 0.52936056 | 0.500923704 | PLB1 | 1 |
| BP | GO:0062014 | negative regulation of small molecule metabolic process | 0.364657102 | 0.52936056 | 0.500923704 | CNR1 | 1 |
| BP | GO:0071867 | response to monoamine | 0.364657102 | 0.52936056 | 0.500923704 | HTR1E | 1 |
| BP | GO:0071869 | response to catecholamine | 0.364657102 | 0.52936056 | 0.500923704 | HTR1E | 1 |
| BP | GO:0090263 | positive regulation of canonical Wnt signaling pathway | 0.364657102 | 0.52936056 | 0.500923704 | GPC5 | 1 |
| BP | GO:1902106 | negative regulation of leukocyte differentiation | 0.364657102 | 0.52936056 | 0.500923704 | TNFSF18 | 1 |
| BP | GO:0051056 | regulation of small GTPase mediated signal transduction | 0.365162435 | 0.529652758 | 0.501200205 | ROBO1/VAV2 | 2 |
| BP | GO:0042116 | macrophage activation | 0.367327605 | 0.531907471 | 0.503333796 | PLCG2 | 1 |
| BP | GO:0048024 | regulation of mRNA splicing, via spliceosome | 0.367327605 | 0.531907471 | 0.503333796 | PRDX6 | 1 |
| BP | GO:0010720 | positive regulation of cell development | 0.368171942 | 0.532687311 | 0.504071744 | ROBO1/NEDD9 | 2 |
| BP | GO:0019395 | fatty acid oxidation | 0.369987023 | 0.532948173 | 0.504318593 | CNR1 | 1 |
| BP | GO:0045833 | negative regulation of lipid metabolic process | 0.369987023 | 0.532948173 | 0.504318593 | CNR1 | 1 |
| BP | GO:1902749 | regulation of cell cycle G2/M phase transition | 0.369987023 | 0.532948173 | 0.504318593 | FOXN3 | 1 |
| BP | GO:1904035 | regulation of epithelial cell apoptotic process | 0.369987023 | 0.532948173 | 0.504318593 | FASLG | 1 |
| BP | GO:0001676 | long-chain fatty acid metabolic process | 0.372635405 | 0.532948173 | 0.504318593 | PLA2G2F | 1 |
| BP | GO:0016079 | synaptic vesicle exocytosis | 0.372635405 | 0.532948173 | 0.504318593 | CNR1 | 1 |
| BP | GO:0021761 | limbic system development | 0.372635405 | 0.532948173 | 0.504318593 | DCLK2 | 1 |
| BP | GO:0022037 | metencephalon development | 0.372635405 | 0.532948173 | 0.504318593 | ATRN | 1 |
| BP | GO:0030148 | sphingolipid biosynthetic process | 0.372635405 | 0.532948173 | 0.504318593 | ST8SIA1 | 1 |
| BP | GO:0032611 | interleukin-1 beta production | 0.372635405 | 0.532948173 | 0.504318593 | ISL1 | 1 |
| BP | GO:0032651 | regulation of interleukin-1 beta production | 0.372635405 | 0.532948173 | 0.504318593 | ISL1 | 1 |
| BP | GO:0042177 | negative regulation of protein catabolic process | 0.372635405 | 0.532948173 | 0.504318593 | FHIT | 1 |
| BP | GO:0071347 | cellular response to interleukin-1 | 0.372635405 | 0.532948173 | 0.504318593 | ST18 | 1 |
| BP | GO:0120034 | positive regulation of plasma membrane bounded cell projection assembly | 0.372635405 | 0.532948173 | 0.504318593 | DNM3 | 1 |
| BP | GO:0036503 | ERAD pathway | 0.375272793 | 0.534657543 | 0.505936137 | DNAJB9 | 1 |
| BP | GO:0043473 | pigmentation | 0.375272793 | 0.534657543 | 0.505936137 | ATRN | 1 |
| BP | GO:0045445 | myoblast differentiation | 0.375272793 | 0.534657543 | 0.505936137 | ISL1 | 1 |
| BP | GO:0048259 | regulation of receptor-mediated endocytosis | 0.375272793 | 0.534657543 | 0.505936137 | PLCG2 | 1 |
| BP | GO:0022604 | regulation of cell morphogenesis | 0.37567212 | 0.534657543 | 0.505936137 | NEDD9/GNA12 | 2 |
| BP | GO:2001020 | regulation of response to DNA damage stimulus | 0.37567212 | 0.534657543 | 0.505936137 | EYA1/SLF2 | 2 |
| BP | GO:0018105 | peptidyl-serine phosphorylation | 0.377167993 | 0.535910802 | 0.507122072 | DCLK2/RICTOR | 2 |
| BP | GO:0060485 | mesenchyme development | 0.377167993 | 0.535910802 | 0.507122072 | ROBO1/ISL1 | 2 |
| BP | GO:0009063 | cellular amino acid catabolic process | 0.377899234 | 0.536075297 | 0.50727773 | BCKDHB | 1 |
| BP | GO:0031123 | RNA 3'-end processing | 0.377899234 | 0.536075297 | 0.50727773 | PTCD1 | 1 |
| BP | GO:0050890 | cognition | 0.378662445 | 0.536720894 | 0.507888646 | NEDD9/CNR1 | 2 |
| BP | GO:0002526 | acute inflammatory response | 0.380514772 | 0.537812153 | 0.508921283 | CNR1 | 1 |
| BP | GO:0009408 | response to heat | 0.380514772 | 0.537812153 | 0.508921283 | ST8SIA1 | 1 |
| BP | GO:1903707 | negative regulation of hemopoiesis | 0.380514772 | 0.537812153 | 0.508921283 | TNFSF18 | 1 |
| BP | GO:0007281 | germ cell development | 0.381647028 | 0.537812153 | 0.508921283 | SPAG17/MEI4 | 2 |
| BP | GO:0032414 | positive regulation of ion transmembrane transporter activity | 0.383119452 | 0.537812153 | 0.508921283 | PLCG2 | 1 |
| BP | GO:0033559 | unsaturated fatty acid metabolic process | 0.383119452 | 0.537812153 | 0.508921283 | PLA2G2F | 1 |
| BP | GO:0034502 | protein localization to chromosome | 0.383119452 | 0.537812153 | 0.508921283 | SLF2 | 1 |
| BP | GO:0035710 | CD4-positive, alpha-beta T cell activation | 0.383119452 | 0.537812153 | 0.508921283 | TNFSF18 | 1 |
| BP | GO:0046634 | regulation of alpha-beta T cell activation | 0.383119452 | 0.537812153 | 0.508921283 | TNFSF18 | 1 |
| BP | GO:0048640 | negative regulation of developmental growth | 0.383119452 | 0.537812153 | 0.508921283 | FXN | 1 |
| BP | GO:1990748 | cellular detoxification | 0.383119452 | 0.537812153 | 0.508921283 | PRDX6 | 1 |
| BP | GO:1902105 | regulation of leukocyte differentiation | 0.383137131 | 0.537812153 | 0.508921283 | TNFSF18/NEDD9 | 2 |
| BP | GO:0030198 | extracellular matrix organization | 0.384625757 | 0.539381441 | 0.51040627 | ADAMTSL1/ADAMTS18 | 2 |
| BP | GO:0030518 | intracellular steroid hormone receptor signaling pathway | 0.385713318 | 0.539381441 | 0.51040627 | ISL1 | 1 |
| BP | GO:0034440 | lipid oxidation | 0.385713318 | 0.539381441 | 0.51040627 | CNR1 | 1 |
| BP | GO:0046916 | cellular transition metal ion homeostasis | 0.385713318 | 0.539381441 | 0.51040627 | FXN | 1 |
| BP | GO:0043062 | extracellular structure organization | 0.386112891 | 0.539381441 | 0.51040627 | ADAMTSL1/ADAMTS18 | 2 |
| BP | GO:0060326 | cell chemotaxis | 0.386112891 | 0.539381441 | 0.51040627 | TNFSF18/NEDD9 | 2 |
| BP | GO:0021987 | cerebral cortex development | 0.388296416 | 0.540927333 | 0.511869118 | ROBO1 | 1 |
| BP | GO:0043406 | positive regulation of MAP kinase activity | 0.388296416 | 0.540927333 | 0.511869118 | ROBO1 | 1 |
| BP | GO:0046632 | alpha-beta T cell differentiation | 0.388296416 | 0.540927333 | 0.511869118 | TNFSF18 | 1 |
| BP | GO:0009416 | response to light stimulus | 0.389082634 | 0.540927333 | 0.511869118 | TULP1/ATP8A2 | 2 |
| BP | GO:0045229 | external encapsulating structure organization | 0.389082634 | 0.540927333 | 0.511869118 | ADAMTSL1/ADAMTS18 | 2 |
| BP | GO:0098813 | nuclear chromosome segregation | 0.389082634 | 0.540927333 | 0.511869118 | MEI4/SLF2 | 2 |
| BP | GO:0002286 | T cell activation involved in immune response | 0.390868788 | 0.541250738 | 0.51217515 | TNFSF18 | 1 |
| BP | GO:0002718 | regulation of cytokine production involved in immune response | 0.390868788 | 0.541250738 | 0.51217515 | PLCG2 | 1 |
| BP | GO:0019751 | polyol metabolic process | 0.390868788 | 0.541250738 | 0.51217515 | PLCG2 | 1 |
| BP | GO:0051261 | protein depolymerization | 0.390868788 | 0.541250738 | 0.51217515 | SH3GL2 | 1 |
| BP | GO:0071346 | cellular response to interferon-gamma | 0.390868788 | 0.541250738 | 0.51217515 | FASLG | 1 |
| BP | GO:0006906 | vesicle fusion | 0.393430479 | 0.543071231 | 0.513897848 | STX6 | 1 |
| BP | GO:0015718 | monocarboxylic acid transport | 0.393430479 | 0.543071231 | 0.513897848 | PLA2G2F | 1 |
| BP | GO:0032609 | interferon-gamma production | 0.393430479 | 0.543071231 | 0.513897848 | ISL1 | 1 |
| BP | GO:0032649 | regulation of interferon-gamma production | 0.393430479 | 0.543071231 | 0.513897848 | ISL1 | 1 |
| BP | GO:1901214 | regulation of neuron death | 0.395003639 | 0.54443554 | 0.515188867 | FASLG/ISL1 | 2 |
| BP | GO:0002367 | cytokine production involved in immune response | 0.395981532 | 0.54443554 | 0.515188867 | PLCG2 | 1 |
| BP | GO:0007613 | memory | 0.395981532 | 0.54443554 | 0.515188867 | CNR1 | 1 |
| BP | GO:0035967 | cellular response to topologically incorrect protein | 0.395981532 | 0.54443554 | 0.515188867 | DNAJB9 | 1 |
| BP | GO:0051897 | positive regulation of protein kinase B signaling | 0.395981532 | 0.54443554 | 0.515188867 | RICTOR | 1 |
| BP | GO:0000077 | DNA damage checkpoint signaling | 0.398521992 | 0.544917826 | 0.515645245 | FOXN3 | 1 |
| BP | GO:0006720 | isoprenoid metabolic process | 0.398521992 | 0.544917826 | 0.515645245 | PLB1 | 1 |
| BP | GO:0032355 | response to estradiol | 0.398521992 | 0.544917826 | 0.515645245 | ARPC1B | 1 |
| BP | GO:0045446 | endothelial cell differentiation | 0.398521992 | 0.544917826 | 0.515645245 | TJP2 | 1 |
| BP | GO:0051101 | regulation of DNA binding | 0.398521992 | 0.544917826 | 0.515645245 | ISL1 | 1 |
| BP | GO:0090100 | positive regulation of transmembrane receptor protein serine/threonine kinase signaling pathway | 0.398521992 | 0.544917826 | 0.515645245 | BMP3 | 1 |
| BP | GO:0090174 | organelle membrane fusion | 0.398521992 | 0.544917826 | 0.515645245 | STX6 | 1 |
| BP | GO:0071496 | cellular response to external stimulus | 0.399427819 | 0.545728047 | 0.516411941 | RICTOR/MAX | 2 |
| BP | GO:0002698 | negative regulation of immune effector process | 0.401051902 | 0.54666073 | 0.517294521 | TNFSF18 | 1 |
| BP | GO:0002761 | regulation of myeloid leukocyte differentiation | 0.401051902 | 0.54666073 | 0.517294521 | NEDD9 | 1 |
| BP | GO:1901989 | positive regulation of cell cycle phase transition | 0.401051902 | 0.54666073 | 0.517294521 | PLCG2 | 1 |
| BP | GO:0002224 | toll-like receptor signaling pathway | 0.403571305 | 0.547951062 | 0.518515538 | PLCG2 | 1 |
| BP | GO:0030203 | glycosaminoglycan metabolic process | 0.403571305 | 0.547951062 | 0.518515538 | ITIH2 | 1 |
| BP | GO:0046887 | positive regulation of hormone secretion | 0.403571305 | 0.547951062 | 0.518515538 | ISL1 | 1 |
| BP | GO:0090630 | activation of GTPase activity | 0.403571305 | 0.547951062 | 0.518515538 | NEDD9 | 1 |
| BP | GO:0097237 | cellular response to toxic substance | 0.403571305 | 0.547951062 | 0.518515538 | PRDX6 | 1 |
| BP | GO:0006310 | DNA recombination | 0.405303984 | 0.548791143 | 0.51931049 | MEI4/SLF2 | 2 |
| BP | GO:0009895 | negative regulation of catabolic process | 0.405303984 | 0.548791143 | 0.51931049 | FHIT/CNR1 | 2 |
| BP | GO:0018209 | peptidyl-serine modification | 0.405303984 | 0.548791143 | 0.51931049 | DCLK2/RICTOR | 2 |
| BP | GO:0055007 | cardiac muscle cell differentiation | 0.406080243 | 0.548791143 | 0.51931049 | ISL1 | 1 |
| BP | GO:0072089 | stem cell proliferation | 0.406080243 | 0.548791143 | 0.51931049 | ETV6 | 1 |
| BP | GO:0072676 | lymphocyte migration | 0.406080243 | 0.548791143 | 0.51931049 | NEDD9 | 1 |
| BP | GO:0002440 | production of molecular mediator of immune response | 0.406768884 | 0.548870175 | 0.519385277 | DNAJB9/PLCG2 | 2 |
| BP | GO:0015980 | energy derivation by oxidation of organic compounds | 0.406768884 | 0.548870175 | 0.519385277 | PDHA2/FXN | 2 |
| BP | GO:0010811 | positive regulation of cell-substrate adhesion | 0.408578761 | 0.550885605 | 0.521292439 | NEDD9 | 1 |
| BP | GO:0060562 | epithelial tube morphogenesis | 0.409693633 | 0.551678382 | 0.522042628 | IRX1/EYA1 | 2 |
| BP | GO:0006690 | icosanoid metabolic process | 0.4110669 | 0.551678382 | 0.522042628 | PLA2G2F | 1 |
| BP | GO:0032411 | positive regulation of transporter activity | 0.4110669 | 0.551678382 | 0.522042628 | PLCG2 | 1 |
| BP | GO:0072329 | monocarboxylic acid catabolic process | 0.4110669 | 0.551678382 | 0.522042628 | CNR1 | 1 |
| BP | GO:0098659 | inorganic cation import across plasma membrane | 0.4110669 | 0.551678382 | 0.522042628 | SLC9C2 | 1 |
| BP | GO:0099587 | inorganic ion import across plasma membrane | 0.4110669 | 0.551678382 | 0.522042628 | SLC9C2 | 1 |
| BP | GO:0001704 | formation of primary germ layer | 0.413544703 | 0.553723961 | 0.523978321 | EYA1 | 1 |
| BP | GO:0001838 | embryonic epithelial tube formation | 0.413544703 | 0.553723961 | 0.523978321 | IRX1 | 1 |
| BP | GO:0006911 | phagocytosis, engulfment | 0.413544703 | 0.553723961 | 0.523978321 | PLCG2 | 1 |
| BP | GO:0048545 | response to steroid hormone | 0.415522602 | 0.555746377 | 0.525892094 | ISL1/BCKDHB | 2 |
| BP | GO:0045471 | response to ethanol | 0.416012213 | 0.555746377 | 0.525892094 | CNR1 | 1 |
| BP | GO:0046683 | response to organophosphorus | 0.416012213 | 0.555746377 | 0.525892094 | BCKDHB | 1 |
| BP | GO:0007041 | lysosomal transport | 0.418469472 | 0.556468565 | 0.526575487 | VPS53 | 1 |
| BP | GO:0031570 | DNA integrity checkpoint signaling | 0.418469472 | 0.556468565 | 0.526575487 | FOXN3 | 1 |
| BP | GO:0048593 | camera-type eye morphogenesis | 0.418469472 | 0.556468565 | 0.526575487 | ATP8A2 | 1 |
| BP | GO:0050714 | positive regulation of protein secretion | 0.418469472 | 0.556468565 | 0.526575487 | ISL1 | 1 |
| BP | GO:0051928 | positive regulation of calcium ion transport | 0.418469472 | 0.556468565 | 0.526575487 | PLCG2 | 1 |
| BP | GO:2000027 | regulation of animal organ morphogenesis | 0.418469472 | 0.556468565 | 0.526575487 | ROBO1 | 1 |
| BP | GO:0051235 | maintenance of location | 0.419876016 | 0.556747594 | 0.526839526 | FASLG/PLCG2 | 2 |
| BP | GO:0032612 | interleukin-1 production | 0.420916522 | 0.556747594 | 0.526839526 | ISL1 | 1 |
| BP | GO:0032652 | regulation of interleukin-1 production | 0.420916522 | 0.556747594 | 0.526839526 | ISL1 | 1 |
| BP | GO:0032874 | positive regulation of stress-activated MAPK cascade | 0.420916522 | 0.556747594 | 0.526839526 | GADD45G | 1 |
| BP | GO:0042542 | response to hydrogen peroxide | 0.420916522 | 0.556747594 | 0.526839526 | FXN | 1 |
| BP | GO:0050853 | B cell receptor signaling pathway | 0.420916522 | 0.556747594 | 0.526839526 | PLCG2 | 1 |
| BP | GO:0071621 | granulocyte chemotaxis | 0.420916522 | 0.556747594 | 0.526839526 | TNFSF18 | 1 |
| BP | GO:0007127 | meiosis I | 0.423353404 | 0.558698205 | 0.528685352 | MEI4 | 1 |
| BP | GO:0007368 | determination of left/right symmetry | 0.423353404 | 0.558698205 | 0.528685352 | TBC1D32 | 1 |
| BP | GO:0032543 | mitochondrial translation | 0.423353404 | 0.558698205 | 0.528685352 | PTCD1 | 1 |
| BP | GO:0070588 | calcium ion transmembrane transport | 0.424213412 | 0.559409359 | 0.529358303 | FASLG/PLCG2 | 2 |
| BP | GO:0070304 | positive regulation of stress-activated protein kinase signaling cascade | 0.425780161 | 0.561050712 | 0.530911484 | GADD45G | 1 |
| BP | GO:0035303 | regulation of dephosphorylation | 0.428196834 | 0.563382844 | 0.533118336 | GNA12 | 1 |
| BP | GO:0035601 | protein deacylation | 0.428196834 | 0.563382844 | 0.533118336 | ABHD10 | 1 |
| BP | GO:0007517 | muscle organ development | 0.428534508 | 0.563401594 | 0.533136079 | ISL1/SGCG | 2 |
| BP | GO:0045765 | regulation of angiogenesis | 0.429971204 | 0.564417784 | 0.53409768 | FASLG/ISL1 | 2 |
| BP | GO:0006022 | aminoglycan metabolic process | 0.430603465 | 0.564417784 | 0.53409768 | ITIH2 | 1 |
| BP | GO:0006661 | phosphatidylinositol biosynthetic process | 0.430603465 | 0.564417784 | 0.53409768 | PLCG2 | 1 |
| BP | GO:0008033 | tRNA processing | 0.430603465 | 0.564417784 | 0.53409768 | PTCD1 | 1 |
| BP | GO:0042113 | B cell activation | 0.431406049 | 0.565044615 | 0.534690837 | DNAJB9/PLCG2 | 2 |
| BP | GO:0035270 | endocrine system development | 0.433000093 | 0.565856086 | 0.535458717 | ISL1 | 1 |
| BP | GO:1900180 | regulation of protein localization to nucleus | 0.433000093 | 0.565856086 | 0.535458717 | DCLK2 | 1 |
| BP | GO:2000779 | regulation of double-strand break repair | 0.433000093 | 0.565856086 | 0.535458717 | SLF2 | 1 |
| BP | GO:0045861 | negative regulation of proteolysis | 0.434270147 | 0.565986818 | 0.535582426 | FHIT/ITIH2 | 2 |
| BP | GO:0006275 | regulation of DNA replication | 0.435386762 | 0.565986818 | 0.535582426 | SMARCAL1 | 1 |
| BP | GO:0007498 | mesoderm development | 0.435386762 | 0.565986818 | 0.535582426 | EYA1 | 1 |
| BP | GO:0010565 | regulation of cellular ketone metabolic process | 0.435386762 | 0.565986818 | 0.535582426 | CNR1 | 1 |
| BP | GO:0043401 | steroid hormone mediated signaling pathway | 0.435386762 | 0.565986818 | 0.535582426 | ISL1 | 1 |
| BP | GO:0099024 | plasma membrane invagination | 0.435386762 | 0.565986818 | 0.535582426 | PLCG2 | 1 |
| BP | GO:0007265 | Ras protein signal transduction | 0.435699382 | 0.565986818 | 0.535582426 | ROBO1/GNA12 | 2 |
| BP | GO:0016050 | vesicle organization | 0.435699382 | 0.565986818 | 0.535582426 | FASLG/STX6 | 2 |
| BP | GO:0061041 | regulation of wound healing | 0.437763511 | 0.566976978 | 0.536519395 | ADAMTS18 | 1 |
| BP | GO:0061982 | meiosis I cell cycle process | 0.437763511 | 0.566976978 | 0.536519395 | MEI4 | 1 |
| BP | GO:0098732 | macromolecule deacylation | 0.437763511 | 0.566976978 | 0.536519395 | ABHD10 | 1 |
| BP | GO:1904019 | epithelial cell apoptotic process | 0.437763511 | 0.566976978 | 0.536519395 | FASLG | 1 |
| BP | GO:1901342 | regulation of vasculature development | 0.438552173 | 0.567576438 | 0.537086653 | FASLG/ISL1 | 2 |
| BP | GO:0030879 | mammary gland development | 0.440130381 | 0.569196082 | 0.538619291 | ROBO1 | 1 |
| BP | GO:0016311 | dephosphorylation | 0.441397334 | 0.569285873 | 0.538704258 | GNA12/EYA1 | 2 |
| BP | GO:0003158 | endothelium development | 0.442487412 | 0.569285873 | 0.538704258 | TJP2 | 1 |
| BP | GO:0007586 | digestion | 0.442487412 | 0.569285873 | 0.538704258 | TJP2 | 1 |
| BP | GO:0008277 | regulation of G protein-coupled receptor signaling pathway | 0.442487412 | 0.569285873 | 0.538704258 | RGS6 | 1 |
| BP | GO:0050684 | regulation of mRNA processing | 0.442487412 | 0.569285873 | 0.538704258 | PRDX6 | 1 |
| BP | GO:0072175 | epithelial tube formation | 0.442487412 | 0.569285873 | 0.538704258 | IRX1 | 1 |
| BP | GO:1903008 | organelle disassembly | 0.442487412 | 0.569285873 | 0.538704258 | NEDD9 | 1 |
| BP | GO:0009306 | protein secretion | 0.444234793 | 0.569425319 | 0.538836214 | ISL1/CNR1 | 2 |
| BP | GO:0002705 | positive regulation of leukocyte mediated immunity | 0.444834646 | 0.569425319 | 0.538836214 | PLCG2 | 1 |
| BP | GO:0034341 | response to interferon-gamma | 0.444834646 | 0.569425319 | 0.538836214 | FASLG | 1 |
| BP | GO:0042157 | lipoprotein metabolic process | 0.444834646 | 0.569425319 | 0.538836214 | ABHD10 | 1 |
| BP | GO:0048813 | dendrite morphogenesis | 0.444834646 | 0.569425319 | 0.538836214 | DNM3 | 1 |
| BP | GO:1903313 | positive regulation of mRNA metabolic process | 0.444834646 | 0.569425319 | 0.538836214 | PRDX6 | 1 |
| BP | GO:0035592 | establishment of protein localization to extracellular region | 0.445650612 | 0.569425319 | 0.538836214 | ISL1/CNR1 | 2 |
| BP | GO:0042176 | regulation of protein catabolic process | 0.447064479 | 0.569425319 | 0.538836214 | FHIT/GNA12 | 2 |
| BP | GO:0006986 | response to unfolded protein | 0.447172122 | 0.569425319 | 0.538836214 | DNAJB9 | 1 |
| BP | GO:0007093 | mitotic cell cycle checkpoint signaling | 0.447172122 | 0.569425319 | 0.538836214 | FOXN3 | 1 |
| BP | GO:0009855 | determination of bilateral symmetry | 0.447172122 | 0.569425319 | 0.538836214 | TBC1D32 | 1 |
| BP | GO:0034754 | cellular hormone metabolic process | 0.447172122 | 0.569425319 | 0.538836214 | PLB1 | 1 |
| BP | GO:0046328 | regulation of JNK cascade | 0.447172122 | 0.569425319 | 0.538836214 | GADD45G | 1 |
| BP | GO:0070555 | response to interleukin-1 | 0.447172122 | 0.569425319 | 0.538836214 | ST18 | 1 |
| BP | GO:0045787 | positive regulation of cell cycle | 0.448476386 | 0.56988995 | 0.539275885 | SLF2/PLCG2 | 2 |
| BP | GO:0000086 | G2/M transition of mitotic cell cycle | 0.44949988 | 0.56988995 | 0.539275885 | FOXN3 | 1 |
| BP | GO:0009799 | specification of symmetry | 0.44949988 | 0.56988995 | 0.539275885 | TBC1D32 | 1 |
| BP | GO:0010977 | negative regulation of neuron projection development | 0.44949988 | 0.56988995 | 0.539275885 | DNM3 | 1 |
| BP | GO:0014074 | response to purine-containing compound | 0.44949988 | 0.56988995 | 0.539275885 | BCKDHB | 1 |
| BP | GO:0042552 | myelination | 0.44949988 | 0.56988995 | 0.539275885 | ATRN | 1 |
| BP | GO:0010212 | response to ionizing radiation | 0.451817961 | 0.570338325 | 0.539700173 | EYA1 | 1 |
| BP | GO:0010324 | membrane invagination | 0.451817961 | 0.570338325 | 0.539700173 | PLCG2 | 1 |
| BP | GO:0030534 | adult behavior | 0.451817961 | 0.570338325 | 0.539700173 | FXN | 1 |
| BP | GO:0033135 | regulation of peptidyl-serine phosphorylation | 0.451817961 | 0.570338325 | 0.539700173 | RICTOR | 1 |
| BP | GO:0046165 | alcohol biosynthetic process | 0.451817961 | 0.570338325 | 0.539700173 | PLCG2 | 1 |
| BP | GO:0055076 | transition metal ion homeostasis | 0.451817961 | 0.570338325 | 0.539700173 | FXN | 1 |
| BP | GO:0010631 | epithelial cell migration | 0.454104244 | 0.571182812 | 0.540499295 | ROBO1/PLCG2 | 2 |
| BP | GO:0007224 | smoothened signaling pathway | 0.454126404 | 0.571182812 | 0.540499295 | TBC1D32 | 1 |
| BP | GO:0007272 | ensheathment of neurons | 0.454126404 | 0.571182812 | 0.540499295 | ATRN | 1 |
| BP | GO:0008366 | axon ensheathment | 0.454126404 | 0.571182812 | 0.540499295 | ATRN | 1 |
| BP | GO:0030177 | positive regulation of Wnt signaling pathway | 0.454126404 | 0.571182812 | 0.540499295 | GPC5 | 1 |
| BP | GO:0006066 | alcohol metabolic process | 0.455506225 | 0.572504938 | 0.541750397 | PLB1/PLCG2 | 2 |
| BP | GO:0045089 | positive regulation of innate immune response | 0.456425248 | 0.572833416 | 0.54206123 | PLCG2 | 1 |
| BP | GO:1903305 | regulation of regulated secretory pathway | 0.456425248 | 0.572833416 | 0.54206123 | CNR1 | 1 |
| BP | GO:0071692 | protein localization to extracellular region | 0.456906197 | 0.573024186 | 0.542241752 | ISL1/CNR1 | 2 |
| BP | GO:0031589 | cell-substrate adhesion | 0.458304151 | 0.573228634 | 0.542435217 | NEDD9/ATRN | 2 |
| BP | GO:0090132 | epithelium migration | 0.458304151 | 0.573228634 | 0.542435217 | ROBO1/PLCG2 | 2 |
| BP | GO:0001508 | action potential | 0.458714532 | 0.573228634 | 0.542435217 | CNR1 | 1 |
| BP | GO:0031333 | negative regulation of protein-containing complex assembly | 0.458714532 | 0.573228634 | 0.542435217 | ISL1 | 1 |
| BP | GO:0050671 | positive regulation of lymphocyte proliferation | 0.458714532 | 0.573228634 | 0.542435217 | GPAM | 1 |
| BP | GO:0006119 | oxidative phosphorylation | 0.460994297 | 0.574429232 | 0.54357132 | FXN | 1 |
| BP | GO:0051592 | response to calcium ion | 0.460994297 | 0.574429232 | 0.54357132 | PLCG2 | 1 |
| BP | GO:0106106 | cold-induced thermogenesis | 0.460994297 | 0.574429232 | 0.54357132 | GADD45G | 1 |
| BP | GO:0120161 | regulation of cold-induced thermogenesis | 0.460994297 | 0.574429232 | 0.54357132 | GADD45G | 1 |
| BP | GO:0044272 | sulfur compound biosynthetic process | 0.463264582 | 0.576774743 | 0.545790832 | PDHA2 | 1 |
| BP | GO:0090130 | tissue migration | 0.465263401 | 0.576774743 | 0.545790832 | ROBO1/PLCG2 | 2 |
| BP | GO:2001233 | regulation of apoptotic signaling pathway | 0.465263401 | 0.576774743 | 0.545790832 | EYA1/FXN | 2 |
| BP | GO:0016525 | negative regulation of angiogenesis | 0.465525424 | 0.576774743 | 0.545790832 | FASLG | 1 |
| BP | GO:0032946 | positive regulation of mononuclear cell proliferation | 0.465525424 | 0.576774743 | 0.545790832 | GPAM | 1 |
| BP | GO:0046467 | membrane lipid biosynthetic process | 0.465525424 | 0.576774743 | 0.545790832 | ST8SIA1 | 1 |
| BP | GO:0051053 | negative regulation of DNA metabolic process | 0.465525424 | 0.576774743 | 0.545790832 | SMARCAL1 | 1 |
| BP | GO:2000377 | regulation of reactive oxygen species metabolic process | 0.465525424 | 0.576774743 | 0.545790832 | PLCG2 | 1 |
| BP | GO:0010821 | regulation of mitochondrion organization | 0.467776863 | 0.578330231 | 0.54726276 | FXN | 1 |
| BP | GO:0030010 | establishment of cell polarity | 0.467776863 | 0.578330231 | 0.54726276 | RICTOR | 1 |
| BP | GO:0034614 | cellular response to reactive oxygen species | 0.467776863 | 0.578330231 | 0.54726276 | FXN | 1 |
| BP | GO:0007269 | neurotransmitter secretion | 0.470018938 | 0.579457176 | 0.548329167 | CNR1 | 1 |
| BP | GO:0099643 | signal release from synapse | 0.470018938 | 0.579457176 | 0.548329167 | CNR1 | 1 |
| BP | GO:1903531 | negative regulation of secretion by cell | 0.470018938 | 0.579457176 | 0.548329167 | CNR1 | 1 |
| BP | GO:2000181 | negative regulation of blood vessel morphogenesis | 0.470018938 | 0.579457176 | 0.548329167 | FASLG | 1 |
| BP | GO:1901343 | negative regulation of vasculature development | 0.472251687 | 0.581798047 | 0.550544287 | FASLG | 1 |
| BP | GO:0016331 | morphogenesis of embryonic epithelium | 0.474475148 | 0.583299723 | 0.551965294 | IRX1 | 1 |
| BP | GO:0030902 | hindbrain development | 0.474475148 | 0.583299723 | 0.551965294 | ATRN | 1 |
| BP | GO:0048284 | organelle fusion | 0.474475148 | 0.583299723 | 0.551965294 | STX6 | 1 |
| BP | GO:0007059 | chromosome segregation | 0.476290625 | 0.583961226 | 0.552591262 | MEI4/SLF2 | 2 |
| BP | GO:0008360 | regulation of cell shape | 0.476689359 | 0.583961226 | 0.552591262 | GNA12 | 1 |
| BP | GO:0043524 | negative regulation of neuron apoptotic process | 0.476689359 | 0.583961226 | 0.552591262 | ISL1 | 1 |
| BP | GO:0045834 | positive regulation of lipid metabolic process | 0.476689359 | 0.583961226 | 0.552591262 | VAV2 | 1 |
| BP | GO:0098754 | detoxification | 0.476689359 | 0.583961226 | 0.552591262 | PRDX6 | 1 |
| BP | GO:0016052 | carbohydrate catabolic process | 0.478894359 | 0.585178112 | 0.553742778 | ABHD10 | 1 |
| BP | GO:0035148 | tube formation | 0.478894359 | 0.585178112 | 0.553742778 | IRX1 | 1 |
| BP | GO:2001236 | regulation of extrinsic apoptotic signaling pathway | 0.478894359 | 0.585178112 | 0.553742778 | EYA1 | 1 |
| BP | GO:1902903 | regulation of supramolecular fiber organization | 0.479026399 | 0.585178112 | 0.553742778 | RICTOR/ARPC1B | 2 |
| BP | GO:0044282 | small molecule catabolic process | 0.480391096 | 0.586433979 | 0.554931181 | BCKDHB/CNR1 | 2 |
| BP | GO:0050770 | regulation of axonogenesis | 0.481090185 | 0.586876122 | 0.555349572 | ROBO1 | 1 |
| BP | GO:0044839 | cell cycle G2/M phase transition | 0.483276875 | 0.588719102 | 0.557093548 | FOXN3 | 1 |
| BP | GO:0048754 | branching morphogenesis of an epithelial tube | 0.483276875 | 0.588719102 | 0.557093548 | EYA1 | 1 |
| BP | GO:0097530 | granulocyte migration | 0.485454466 | 0.590958546 | 0.559212692 | TNFSF18 | 1 |
| BP | GO:0001539 | cilium or flagellum-dependent cell motility | 0.487622996 | 0.591943735 | 0.560144956 | DNAH8 | 1 |
| BP | GO:0007605 | sensory perception of sound | 0.487622996 | 0.591943735 | 0.560144956 | EYA1 | 1 |
| BP | GO:0035051 | cardiocyte differentiation | 0.487622996 | 0.591943735 | 0.560144956 | ISL1 | 1 |
| BP | GO:0060285 | cilium-dependent cell motility | 0.487622996 | 0.591943735 | 0.560144956 | DNAH8 | 1 |
| BP | GO:0007338 | single fertilization | 0.489782502 | 0.592912522 | 0.561061701 | PLB1 | 1 |
| BP | GO:0051017 | actin filament bundle assembly | 0.489782502 | 0.592912522 | 0.561061701 | NEDD9 | 1 |
| BP | GO:1902600 | proton transmembrane transport | 0.489782502 | 0.592912522 | 0.561061701 | SLC9C2 | 1 |
| BP | GO:1990845 | adaptive thermogenesis | 0.489782502 | 0.592912522 | 0.561061701 | GADD45G | 1 |
| BP | GO:0002181 | cytoplasmic translation | 0.491933021 | 0.595102308 | 0.563133854 | RPL37A | 1 |
| BP | GO:0071900 | regulation of protein serine/threonine kinase activity | 0.493919624 | 0.596864034 | 0.564800941 | ROBO1/RICTOR | 2 |
| BP | GO:0046488 | phosphatidylinositol metabolic process | 0.494074591 | 0.596864034 | 0.564800941 | PLCG2 | 1 |
| BP | GO:0006665 | sphingolipid metabolic process | 0.496207247 | 0.598195864 | 0.566061226 | ST8SIA1 | 1 |
| BP | GO:0035966 | response to topologically incorrect protein | 0.496207247 | 0.598195864 | 0.566061226 | DNAJB9 | 1 |
| BP | GO:0140053 | mitochondrial gene expression | 0.496207247 | 0.598195864 | 0.566061226 | PTCD1 | 1 |
| BP | GO:0050900 | leukocyte migration | 0.497935672 | 0.599511497 | 0.567306184 | TNFSF18/NEDD9 | 2 |
| BP | GO:0021915 | neural tube development | 0.498331026 | 0.599511497 | 0.567306184 | TBC1D32 | 1 |
| BP | GO:0061572 | actin filament bundle organization | 0.498331026 | 0.599511497 | 0.567306184 | NEDD9 | 1 |
| BP | GO:0006261 | DNA-templated DNA replication | 0.502552101 | 0.603755697 | 0.571322389 | SMARCAL1 | 1 |
| BP | GO:0007009 | plasma membrane organization | 0.502552101 | 0.603755697 | 0.571322389 | FASLG | 1 |
| BP | GO:0001959 | regulation of cytokine-mediated signaling pathway | 0.504649469 | 0.605023658 | 0.572522236 | ROBO1 | 1 |
| BP | GO:0043271 | negative regulation of ion transport | 0.504649469 | 0.605023658 | 0.572522236 | CNR1 | 1 |
| BP | GO:0070665 | positive regulation of leukocyte proliferation | 0.504649469 | 0.605023658 | 0.572522236 | GPAM | 1 |
| BP | GO:0000724 | double-strand break repair via homologous recombination | 0.508818046 | 0.609182843 | 0.576457993 | SLF2 | 1 |
| BP | GO:0001837 | epithelial to mesenchymal transition | 0.508818046 | 0.609182843 | 0.576457993 | ISL1 | 1 |
| BP | GO:0007156 | homophilic cell adhesion via plasma membrane adhesion molecules | 0.510889327 | 0.610823067 | 0.578010105 | ROBO1 | 1 |
| BP | GO:0035637 | multicellular organismal signaling | 0.510889327 | 0.610823067 | 0.578010105 | ISL1 | 1 |
| BP | GO:1904064 | positive regulation of cation transmembrane transport | 0.512951984 | 0.612868557 | 0.579945712 | PLCG2 | 1 |
| BP | GO:0007254 | JNK cascade | 0.515006051 | 0.613639221 | 0.580674977 | GADD45G | 1 |
| BP | GO:1903034 | regulation of response to wounding | 0.515006051 | 0.613639221 | 0.580674977 | ADAMTS18 | 1 |
| BP | GO:1905475 | regulation of protein localization to membrane | 0.515006051 | 0.613639221 | 0.580674977 | GPC5 | 1 |
| BP | GO:1990138 | neuron projection extension | 0.515006051 | 0.613639221 | 0.580674977 | SH3GL2 | 1 |
| BP | GO:0000725 | recombinational repair | 0.517051565 | 0.615234854 | 0.582184894 | SLF2 | 1 |
| BP | GO:0072331 | signal transduction by p53 class mediator | 0.517051565 | 0.615234854 | 0.582184894 | FHIT | 1 |
| BP | GO:1903706 | regulation of hemopoiesis | 0.517715146 | 0.615603949 | 0.582534162 | TNFSF18/NEDD9 | 2 |
| BP | GO:0046631 | alpha-beta T cell activation | 0.519088561 | 0.616395551 | 0.583283239 | TNFSF18 | 1 |
| BP | GO:0055088 | lipid homeostasis | 0.519088561 | 0.616395551 | 0.583283239 | GPAM | 1 |
| BP | GO:0009267 | cellular response to starvation | 0.521117073 | 0.617121646 | 0.583970329 | MAX | 1 |
| BP | GO:0031056 | regulation of histone modification | 0.521117073 | 0.617121646 | 0.583970329 | ISL1 | 1 |
| BP | GO:0048660 | regulation of smooth muscle cell proliferation | 0.521117073 | 0.617121646 | 0.583970329 | GNA12 | 1 |
| BP | GO:0051048 | negative regulation of secretion | 0.521117073 | 0.617121646 | 0.583970329 | CNR1 | 1 |
| BP | GO:0010634 | positive regulation of epithelial cell migration | 0.523137136 | 0.618252979 | 0.585040888 | PLCG2 | 1 |
| BP | GO:1901136 | carbohydrate derivative catabolic process | 0.523137136 | 0.618252979 | 0.585040888 | ABHD10 | 1 |
| BP | GO:1902905 | positive regulation of supramolecular fiber organization | 0.523137136 | 0.618252979 | 0.585040888 | RICTOR | 1 |
| BP | GO:0001764 | neuron migration | 0.525148786 | 0.618950735 | 0.58570116 | UNC5C | 1 |
| BP | GO:0043534 | blood vessel endothelial cell migration | 0.525148786 | 0.618950735 | 0.58570116 | ROBO1 | 1 |
| BP | GO:0051099 | positive regulation of binding | 0.525148786 | 0.618950735 | 0.58570116 | ISL1 | 1 |
| BP | GO:0061025 | membrane fusion | 0.525148786 | 0.618950735 | 0.58570116 | STX6 | 1 |
| BP | GO:0045580 | regulation of T cell differentiation | 0.529146983 | 0.622204239 | 0.588779889 | TNFSF18 | 1 |
| BP | GO:0048659 | smooth muscle cell proliferation | 0.529146983 | 0.622204239 | 0.588779889 | GNA12 | 1 |
| BP | GO:0060759 | regulation of response to cytokine stimulus | 0.529146983 | 0.622204239 | 0.588779889 | ROBO1 | 1 |
| BP | GO:0001558 | regulation of cell growth | 0.529337934 | 0.622204239 | 0.588779889 | RICTOR/FXN | 2 |
| BP | GO:0006575 | cellular modified amino acid metabolic process | 0.531133598 | 0.622215688 | 0.588790723 | PLA2G2F | 1 |
| BP | GO:0016573 | histone acetylation | 0.531133598 | 0.622215688 | 0.588790723 | ISL1 | 1 |
| BP | GO:0035265 | organ growth | 0.531133598 | 0.622215688 | 0.588790723 | FXN | 1 |
| BP | GO:0043484 | regulation of RNA splicing | 0.531133598 | 0.622215688 | 0.588790723 | PRDX6 | 1 |
| BP | GO:0050954 | sensory perception of mechanical stimulus | 0.531133598 | 0.622215688 | 0.588790723 | EYA1 | 1 |
| BP | GO:0006941 | striated muscle contraction | 0.533111937 | 0.623694423 | 0.590190021 | ATP8A2 | 1 |
| BP | GO:0042770 | signal transduction in response to DNA damage | 0.533111937 | 0.623694423 | 0.590190021 | FOXN3 | 1 |
| BP | GO:0002822 | regulation of adaptive immune response based on somatic recombination of immune receptors built from immunoglobulin superfamily domains | 0.535082035 | 0.62557913 | 0.591973484 | TNFSF18 | 1 |
| BP | GO:0007219 | Notch signaling pathway | 0.537043923 | 0.626611195 | 0.592950107 | ROBO1 | 1 |
| BP | GO:0008361 | regulation of cell size | 0.537043923 | 0.626611195 | 0.592950107 | VAV2 | 1 |
| BP | GO:0071466 | cellular response to xenobiotic stimulus | 0.537043923 | 0.626611195 | 0.592950107 | VAV2 | 1 |
| BP | GO:0007416 | synapse assembly | 0.538997637 | 0.626791645 | 0.593120863 | DNM3 | 1 |
| BP | GO:0035725 | sodium ion transmembrane transport | 0.538997637 | 0.626791645 | 0.593120863 | SLC9C2 | 1 |
| BP | GO:0050728 | negative regulation of inflammatory response | 0.538997637 | 0.626791645 | 0.593120863 | ISL1 | 1 |
| BP | GO:1902107 | positive regulation of leukocyte differentiation | 0.538997637 | 0.626791645 | 0.593120863 | NEDD9 | 1 |
| BP | GO:1903708 | positive regulation of hemopoiesis | 0.538997637 | 0.626791645 | 0.593120863 | NEDD9 | 1 |
| BP | GO:0042391 | regulation of membrane potential | 0.540773701 | 0.62753692 | 0.593826103 | CNR1/KCNQ3 | 2 |
| BP | GO:0002040 | sprouting angiogenesis | 0.540943211 | 0.62753692 | 0.593826103 | ROBO1 | 1 |
| BP | GO:0009410 | response to xenobiotic stimulus | 0.542032697 | 0.62753692 | 0.593826103 | GNA12/VAV2 | 2 |
| BP | GO:0050808 | synapse organization | 0.542032697 | 0.62753692 | 0.593826103 | DNM3/NEDD9 | 2 |
| BP | GO:0000075 | cell cycle checkpoint signaling | 0.542880677 | 0.62753692 | 0.593826103 | FOXN3 | 1 |
| BP | GO:0009266 | response to temperature stimulus | 0.542880677 | 0.62753692 | 0.593826103 | ST8SIA1 | 1 |
| BP | GO:0018393 | internal peptidyl-lysine acetylation | 0.542880677 | 0.62753692 | 0.593826103 | ISL1 | 1 |
| BP | GO:0034767 | positive regulation of ion transmembrane transport | 0.542880677 | 0.62753692 | 0.593826103 | PLCG2 | 1 |
| BP | GO:0043405 | regulation of MAP kinase activity | 0.542880677 | 0.62753692 | 0.593826103 | ROBO1 | 1 |
| BP | GO:0002221 | pattern recognition receptor signaling pathway | 0.544810068 | 0.628515986 | 0.594752574 | PLCG2 | 1 |
| BP | GO:0045766 | positive regulation of angiogenesis | 0.544810068 | 0.628515986 | 0.594752574 | ISL1 | 1 |
| BP | GO:1904018 | positive regulation of vasculature development | 0.544810068 | 0.628515986 | 0.594752574 | ISL1 | 1 |
| BP | GO:0002833 | positive regulation of response to biotic stimulus | 0.546731419 | 0.628650913 | 0.594880252 | PLCG2 | 1 |
| BP | GO:0006006 | glucose metabolic process | 0.546731419 | 0.628650913 | 0.594880252 | PDHA2 | 1 |
| BP | GO:0006475 | internal protein amino acid acetylation | 0.546731419 | 0.628650913 | 0.594880252 | ISL1 | 1 |
| BP | GO:0061138 | morphogenesis of a branching epithelium | 0.546731419 | 0.628650913 | 0.594880252 | EYA1 | 1 |
| BP | GO:1903169 | regulation of calcium ion transmembrane transport | 0.546731419 | 0.628650913 | 0.594880252 | PLCG2 | 1 |
| BP | GO:0007286 | spermatid development | 0.548644763 | 0.629189715 | 0.59539011 | SPAG17 | 1 |
| BP | GO:0007565 | female pregnancy | 0.548644763 | 0.629189715 | 0.59539011 | CNR1 | 1 |
| BP | GO:0043123 | positive regulation of I-kappaB kinase/NF-kappaB signaling | 0.548644763 | 0.629189715 | 0.59539011 | FASLG | 1 |
| BP | GO:0051896 | regulation of protein kinase B signaling | 0.548644763 | 0.629189715 | 0.59539011 | RICTOR | 1 |
| BP | GO:0050777 | negative regulation of immune response | 0.550550131 | 0.630959426 | 0.597064754 | TNFSF18 | 1 |
| BP | GO:0006816 | calcium ion transport | 0.552020228 | 0.632228295 | 0.59826546 | FASLG/PLCG2 | 2 |
| BP | GO:0055001 | muscle cell development | 0.552447557 | 0.632302001 | 0.598335206 | ISL1 | 1 |
| BP | GO:0048732 | gland development | 0.553258078 | 0.632813901 | 0.598819608 | ROBO1/ISL1 | 2 |
| BP | GO:0009755 | hormone-mediated signaling pathway | 0.554337074 | 0.633216514 | 0.599200593 | ISL1 | 1 |
| BP | GO:0031099 | regeneration | 0.554337074 | 0.633216514 | 0.599200593 | ISL1 | 1 |
| BP | GO:0046578 | regulation of Ras protein signal transduction | 0.556218715 | 0.634949542 | 0.600840524 | ROBO1 | 1 |
| BP | GO:0009749 | response to glucose | 0.558092511 | 0.635422976 | 0.601288525 | GPAM | 1 |
| BP | GO:0031345 | negative regulation of cell projection organization | 0.558092511 | 0.635422976 | 0.601288525 | DNM3 | 1 |
| BP | GO:0140013 | meiotic nuclear division | 0.558092511 | 0.635422976 | 0.601288525 | MEI4 | 1 |
| BP | GO:1901991 | negative regulation of mitotic cell cycle phase transition | 0.558092511 | 0.635422976 | 0.601288525 | FOXN3 | 1 |
| BP | GO:0003341 | cilium movement | 0.559958494 | 0.63588507 | 0.601725796 | SPAG17 | 1 |
| BP | GO:0006399 | tRNA metabolic process | 0.559958494 | 0.63588507 | 0.601725796 | PTCD1 | 1 |
| BP | GO:0032872 | regulation of stress-activated MAPK cascade | 0.559958494 | 0.63588507 | 0.601725796 | GADD45G | 1 |
| BP | GO:0051495 | positive regulation of cytoskeleton organization | 0.559958494 | 0.63588507 | 0.601725796 | RICTOR | 1 |
| BP | GO:0007369 | gastrulation | 0.561816698 | 0.636795412 | 0.602587235 | EYA1 | 1 |
| BP | GO:0048515 | spermatid differentiation | 0.561816698 | 0.636795412 | 0.602587235 | SPAG17 | 1 |
| BP | GO:0050804 | modulation of chemical synaptic transmission | 0.561856802 | 0.636795412 | 0.602587235 | SCGN/CNR1 | 2 |
| BP | GO:0099177 | regulation of trans-synaptic signaling | 0.563075707 | 0.637188957 | 0.602959639 | SCGN/CNR1 | 2 |
| BP | GO:0007626 | locomotory behavior | 0.563667154 | 0.637188957 | 0.602959639 | FXN | 1 |
| BP | GO:0018394 | peptidyl-lysine acetylation | 0.563667154 | 0.637188957 | 0.602959639 | ISL1 | 1 |
| BP | GO:2001257 | regulation of cation channel activity | 0.563667154 | 0.637188957 | 0.602959639 | PLCG2 | 1 |
| BP | GO:1903829 | positive regulation of protein localization | 0.565506385 | 0.638029946 | 0.60375545 | ISL1/NEDD9 | 2 |
| BP | GO:0002819 | regulation of adaptive immune response | 0.565509894 | 0.638029946 | 0.60375545 | TNFSF18 | 1 |
| BP | GO:0070302 | regulation of stress-activated protein kinase signaling cascade | 0.565509894 | 0.638029946 | 0.60375545 | GADD45G | 1 |
| BP | GO:0009746 | response to hexose | 0.56734495 | 0.639272253 | 0.604931022 | GPAM | 1 |
| BP | GO:0120032 | regulation of plasma membrane bounded cell projection assembly | 0.56734495 | 0.639272253 | 0.604931022 | DNM3 | 1 |
| BP | GO:1901987 | regulation of cell cycle phase transition | 0.567927544 | 0.63951505 | 0.605160776 | FOXN3/PLCG2 | 2 |
| BP | GO:0051090 | regulation of DNA-binding transcription factor activity | 0.56913455 | 0.640460198 | 0.606055152 | TNFSF18/PLCG2 | 2 |
| BP | GO:0009566 | fertilization | 0.570992135 | 0.641307737 | 0.606857161 | PLB1 | 1 |
| BP | GO:0051216 | cartilage development | 0.570992135 | 0.641307737 | 0.606857161 | BMP3 | 1 |
| BP | GO:0060491 | regulation of cell projection assembly | 0.570992135 | 0.641307737 | 0.606857161 | DNM3 | 1 |
| BP | GO:0098739 | import across plasma membrane | 0.572804328 | 0.642928569 | 0.608390923 | SLC9C2 | 1 |
| BP | GO:0001763 | morphogenesis of a branching structure | 0.574608962 | 0.644124074 | 0.609522207 | EYA1 | 1 |
| BP | GO:1903828 | negative regulation of protein localization | 0.574608962 | 0.644124074 | 0.609522207 | DCLK2 | 1 |
| BP | GO:0000070 | mitotic sister chromatid segregation | 0.576406068 | 0.645308079 | 0.610642608 | SLF2 | 1 |
| BP | GO:0000302 | response to reactive oxygen species | 0.576406068 | 0.645308079 | 0.610642608 | FXN | 1 |
| BP | GO:0007204 | positive regulation of cytosolic calcium ion concentration | 0.578195678 | 0.646895871 | 0.612145105 | CNR1 | 1 |
| BP | GO:0034284 | response to monosaccharide | 0.581752533 | 0.650040354 | 0.615120669 | GPAM | 1 |
| BP | GO:0048167 | regulation of synaptic plasticity | 0.581752533 | 0.650040354 | 0.615120669 | SCGN | 1 |
| BP | GO:0019722 | calcium-mediated signaling | 0.583519839 | 0.65117973 | 0.616198839 | PLCG2 | 1 |
| BP | GO:0045216 | cell-cell junction organization | 0.583519839 | 0.65117973 | 0.616198839 | TJP2 | 1 |
| BP | GO:0006643 | membrane lipid metabolic process | 0.585279771 | 0.651474352 | 0.616477633 | ST8SIA1 | 1 |
| BP | GO:0030100 | regulation of endocytosis | 0.585279771 | 0.651474352 | 0.616477633 | PLCG2 | 1 |
| BP | GO:0044703 | multi-organism reproductive process | 0.585279771 | 0.651474352 | 0.616477633 | CNR1 | 1 |
| BP | GO:0071383 | cellular response to steroid hormone stimulus | 0.585279771 | 0.651474352 | 0.616477633 | ISL1 | 1 |
| BP | GO:0045619 | regulation of lymphocyte differentiation | 0.58703236 | 0.6530079 | 0.6179288 | TNFSF18 | 1 |
| BP | GO:0023061 | signal release | 0.588121558 | 0.653697031 | 0.618580912 | ISL1/CNR1 | 2 |
| BP | GO:0009612 | response to mechanical stimulus | 0.588777636 | 0.653697031 | 0.618580912 | ATP8A2 | 1 |
| BP | GO:0042594 | response to starvation | 0.588777636 | 0.653697031 | 0.618580912 | MAX | 1 |
| BP | GO:0006836 | neurotransmitter transport | 0.590515629 | 0.654791996 | 0.619617056 | CNR1 | 1 |
| BP | GO:1903046 | meiotic cell cycle process | 0.590515629 | 0.654791996 | 0.619617056 | MEI4 | 1 |
| BP | GO:0002064 | epithelial cell development | 0.592246369 | 0.655043286 | 0.619854847 | TJP2 | 1 |
| BP | GO:0017157 | regulation of exocytosis | 0.592246369 | 0.655043286 | 0.619854847 | CNR1 | 1 |
| BP | GO:0030258 | lipid modification | 0.592246369 | 0.655043286 | 0.619854847 | CNR1 | 1 |
| BP | GO:1902806 | regulation of cell cycle G1/S phase transition | 0.592246369 | 0.655043286 | 0.619854847 | PLCG2 | 1 |
| BP | GO:0008016 | regulation of heart contraction | 0.593969887 | 0.656116387 | 0.620870302 | ISL1 | 1 |
| BP | GO:0045637 | regulation of myeloid cell differentiation | 0.593969887 | 0.656116387 | 0.620870302 | NEDD9 | 1 |
| BP | GO:0002573 | myeloid leukocyte differentiation | 0.600792319 | 0.661166489 | 0.625649116 | NEDD9 | 1 |
| BP | GO:0042180 | cellular ketone metabolic process | 0.600792319 | 0.661166489 | 0.625649116 | CNR1 | 1 |
| BP | GO:0043491 | protein kinase B signaling | 0.600792319 | 0.661166489 | 0.625649116 | RICTOR | 1 |
| BP | GO:0044706 | multi-multicellular organism process | 0.600792319 | 0.661166489 | 0.625649116 | CNR1 | 1 |
| BP | GO:1901215 | negative regulation of neuron death | 0.600792319 | 0.661166489 | 0.625649116 | ISL1 | 1 |
| BP | GO:0000280 | nuclear division | 0.600818916 | 0.661166489 | 0.625649116 | MEI4/SLF2 | 2 |
| BP | GO:0001505 | regulation of neurotransmitter levels | 0.602480165 | 0.662158011 | 0.626587374 | CNR1 | 1 |
| BP | GO:0002377 | immunoglobulin production | 0.602480165 | 0.662158011 | 0.626587374 | DNAJB9 | 1 |
| BP | GO:0022411 | cellular component disassembly | 0.603096276 | 0.662417221 | 0.626832659 | NEDD9/SH3GL2 | 2 |
| BP | GO:0009152 | purine ribonucleotide biosynthetic process | 0.604160965 | 0.663168495 | 0.627543575 | PDHA2 | 1 |
| BP | GO:0001819 | positive regulation of cytokine production | 0.606494279 | 0.665310474 | 0.629570489 | ISL1/PLCG2 | 2 |
| BP | GO:0010810 | regulation of cell-substrate adhesion | 0.607501542 | 0.665577161 | 0.62982285 | NEDD9 | 1 |
| BP | GO:0071222 | cellular response to lipopolysaccharide | 0.607501542 | 0.665577161 | 0.62982285 | PLCG2 | 1 |
| BP | GO:0070374 | positive regulation of ERK1 and ERK2 cascade | 0.609161377 | 0.666976191 | 0.631146725 | ALKAL1 | 1 |
| BP | GO:0019318 | hexose metabolic process | 0.618975837 | 0.676871254 | 0.640510233 | PDHA2 | 1 |
| BP | GO:0048588 | developmental cell growth | 0.618975837 | 0.676871254 | 0.640510233 | SH3GL2 | 1 |
| BP | GO:0006473 | protein acetylation | 0.620587752 | 0.678208196 | 0.641775355 | ISL1 | 1 |
| BP | GO:0050769 | positive regulation of neurogenesis | 0.622192935 | 0.679536108 | 0.643031932 | ROBO1 | 1 |
| BP | GO:0008406 | gonad development | 0.62538321 | 0.681738143 | 0.645115676 | IRX5 | 1 |
| BP | GO:0060348 | bone development | 0.62538321 | 0.681738143 | 0.645115676 | FOXN3 | 1 |
| BP | GO:0060560 | developmental growth involved in morphogenesis | 0.62538321 | 0.681738143 | 0.645115676 | SH3GL2 | 1 |
| BP | GO:0009260 | ribonucleotide biosynthetic process | 0.626968359 | 0.682185435 | 0.64553894 | PDHA2 | 1 |
| BP | GO:0009743 | response to carbohydrate | 0.626968359 | 0.682185435 | 0.64553894 | GPAM | 1 |
| BP | GO:0071219 | cellular response to molecule of bacterial origin | 0.626968359 | 0.682185435 | 0.64553894 | PLCG2 | 1 |
| BP | GO:0072593 | reactive oxygen species metabolic process | 0.628546885 | 0.683476076 | 0.646760248 | PLCG2 | 1 |
| BP | GO:0034764 | positive regulation of transmembrane transport | 0.631684177 | 0.686459037 | 0.649582967 | PLCG2 | 1 |
| BP | GO:0045137 | development of primary sexual characteristics | 0.633242998 | 0.687724004 | 0.650779981 | IRX5 | 1 |
| BP | GO:0000819 | sister chromatid segregation | 0.634795305 | 0.688551321 | 0.651562855 | SLF2 | 1 |
| BP | GO:0051403 | stress-activated MAPK cascade | 0.634795305 | 0.688551321 | 0.651562855 | GADD45G | 1 |
| BP | GO:0045088 | regulation of innate immune response | 0.636341124 | 0.68979853 | 0.652743065 | PLCG2 | 1 |
| BP | GO:0002703 | regulation of leukocyte mediated immunity | 0.637880483 | 0.690122773 | 0.65304989 | PLCG2 | 1 |
| BP | GO:0042445 | hormone metabolic process | 0.637880483 | 0.690122773 | 0.65304989 | PLB1 | 1 |
| BP | GO:0046390 | ribose phosphate biosynthetic process | 0.637880483 | 0.690122773 | 0.65304989 | PDHA2 | 1 |
| BP | GO:0006164 | purine nucleotide biosynthetic process | 0.639413407 | 0.690122773 | 0.65304989 | PDHA2 | 1 |
| BP | GO:0032984 | protein-containing complex disassembly | 0.639413407 | 0.690122773 | 0.65304989 | SH3GL2 | 1 |
| BP | GO:0045055 | regulated exocytosis | 0.639413407 | 0.690122773 | 0.65304989 | CNR1 | 1 |
| BP | GO:0097529 | myeloid leukocyte migration | 0.639413407 | 0.690122773 | 0.65304989 | TNFSF18 | 1 |
| BP | GO:1901617 | organic hydroxy compound biosynthetic process | 0.643973843 | 0.694614511 | 0.657300335 | PLCG2 | 1 |
| BP | GO:0001649 | osteoblast differentiation | 0.645481297 | 0.694949579 | 0.657617403 | BMP3 | 1 |
| BP | GO:0031098 | stress-activated protein kinase signaling cascade | 0.645481297 | 0.694949579 | 0.657617403 | GADD45G | 1 |
| BP | GO:0045930 | negative regulation of mitotic cell cycle | 0.645481297 | 0.694949579 | 0.657617403 | FOXN3 | 1 |
| BP | GO:0033002 | muscle cell proliferation | 0.646982448 | 0.696135531 | 0.658739646 | GNA12 | 1 |
| BP | GO:0005996 | monosaccharide metabolic process | 0.648477323 | 0.696883094 | 0.659447052 | PDHA2 | 1 |
| BP | GO:0042593 | glucose homeostasis | 0.648477323 | 0.696883094 | 0.659447052 | CNR1 | 1 |
| BP | GO:0033500 | carbohydrate homeostasis | 0.649965947 | 0.697622107 | 0.660146365 | CNR1 | 1 |
| BP | GO:0045926 | negative regulation of growth | 0.649965947 | 0.697622107 | 0.660146365 | FXN | 1 |
| BP | GO:0009636 | response to toxic substance | 0.651448347 | 0.698352628 | 0.660837643 | PRDX6 | 1 |
| BP | GO:0016485 | protein processing | 0.651448347 | 0.698352628 | 0.660837643 | FXN | 1 |
| BP | GO:0050870 | positive regulation of T cell activation | 0.652924548 | 0.69893032 | 0.661384302 | GPAM | 1 |
| BP | GO:0072522 | purine-containing compound biosynthetic process | 0.652924548 | 0.69893032 | 0.661384302 | PDHA2 | 1 |
| BP | GO:0010951 | negative regulation of endopeptidase activity | 0.654394576 | 0.69893032 | 0.661384302 | ITIH2 | 1 |
| BP | GO:0033044 | regulation of chromosome organization | 0.654394576 | 0.69893032 | 0.661384302 | SLF2 | 1 |
| BP | GO:0048762 | mesenchymal cell differentiation | 0.654394576 | 0.69893032 | 0.661384302 | ISL1 | 1 |
| BP | GO:0098657 | import into cell | 0.654394576 | 0.69893032 | 0.661384302 | SLC9C2 | 1 |
| BP | GO:0090305 | nucleic acid phosphodiester bond hydrolysis | 0.655858457 | 0.699635904 | 0.662051982 | MEI4 | 1 |
| BP | GO:0097305 | response to alcohol | 0.655858457 | 0.699635904 | 0.662051982 | CNR1 | 1 |
| BP | GO:0006814 | sodium ion transport | 0.658767875 | 0.702309448 | 0.664581906 | SLC9C2 | 1 |
| BP | GO:0034976 | response to endoplasmic reticulum stress | 0.667351202 | 0.710590338 | 0.672417953 | DNAJB9 | 1 |
| BP | GO:0071216 | cellular response to biotic stimulus | 0.667351202 | 0.710590338 | 0.672417953 | PLCG2 | 1 |
| BP | GO:0010466 | negative regulation of peptidase activity | 0.670164663 | 0.712714801 | 0.674428291 | ITIH2 | 1 |
| BP | GO:1903522 | regulation of blood circulation | 0.670164663 | 0.712714801 | 0.674428291 | ISL1 | 1 |
| BP | GO:0030522 | intracellular receptor signaling pathway | 0.67434086 | 0.716718595 | 0.678217004 | ISL1 | 1 |
| BP | GO:0051924 | regulation of calcium ion transport | 0.677095942 | 0.719208006 | 0.680572686 | PLCG2 | 1 |
| BP | GO:0032868 | response to insulin | 0.67846484 | 0.720222883 | 0.681533044 | MAX | 1 |
| BP | GO:0031348 | negative regulation of defense response | 0.682537249 | 0.723223776 | 0.684372732 | ISL1 | 1 |
| BP | GO:0043543 | protein acylation | 0.682537249 | 0.723223776 | 0.684372732 | ISL1 | 1 |
| BP | GO:1901988 | negative regulation of cell cycle phase transition | 0.682537249 | 0.723223776 | 0.684372732 | FOXN3 | 1 |
| BP | GO:0061448 | connective tissue development | 0.685223853 | 0.72562915 | 0.686648891 | BMP3 | 1 |
| BP | GO:1903039 | positive regulation of leukocyte cell-cell adhesion | 0.686558724 | 0.726601031 | 0.687568563 | GPAM | 1 |
| BP | GO:0051962 | positive regulation of nervous system development | 0.691842545 | 0.73174846 | 0.692439476 | ROBO1 | 1 |
| BP | GO:0044843 | cell cycle G1/S phase transition | 0.693149699 | 0.732686151 | 0.693326794 | PLCG2 | 1 |
| BP | GO:0032412 | regulation of ion transmembrane transporter activity | 0.694451379 | 0.733172304 | 0.693786832 | PLCG2 | 1 |
| BP | GO:0043542 | endothelial cell migration | 0.694451379 | 0.733172304 | 0.693786832 | ROBO1 | 1 |
| BP | GO:0098742 | cell-cell adhesion via plasma-membrane adhesion molecules | 0.695747606 | 0.734095899 | 0.694660812 | ROBO1 | 1 |
| BP | GO:0051321 | meiotic cell cycle | 0.699603799 | 0.737271517 | 0.697665838 | MEI4 | 1 |
| BP | GO:0072659 | protein localization to plasma membrane | 0.699603799 | 0.737271517 | 0.697665838 | FYB1 | 1 |
| BP | GO:0006260 | DNA replication | 0.700878442 | 0.738168226 | 0.698514376 | SMARCAL1 | 1 |
| BP | GO:0007548 | sex differentiation | 0.703411728 | 0.739941564 | 0.700192452 | IRX5 | 1 |
| BP | GO:0034599 | cellular response to oxidative stress | 0.703411728 | 0.739941564 | 0.700192452 | FXN | 1 |
| BP | GO:0006520 | cellular amino acid metabolic process | 0.704670416 | 0.74037145 | 0.700599245 | BCKDHB | 1 |
| BP | GO:0043270 | positive regulation of ion transport | 0.704670416 | 0.74037145 | 0.700599245 | PLCG2 | 1 |
| BP | GO:0046942 | carboxylic acid transport | 0.705923829 | 0.740794765 | 0.70099982 | PLA2G2F | 1 |
| BP | GO:0090092 | regulation of transmembrane receptor protein serine/threonine kinase signaling pathway | 0.705923829 | 0.740794765 | 0.70099982 | BMP3 | 1 |
| BP | GO:0022898 | regulation of transmembrane transporter activity | 0.70717199 | 0.741657801 | 0.701816494 | PLCG2 | 1 |
| BP | GO:0010632 | regulation of epithelial cell migration | 0.70841492 | 0.742067824 | 0.702204491 | PLCG2 | 1 |
| BP | GO:0015850 | organic hydroxy compound transport | 0.70841492 | 0.742067824 | 0.702204491 | CNR1 | 1 |
| BP | GO:0051146 | striated muscle cell differentiation | 0.709652641 | 0.742917608 | 0.703008626 | ISL1 | 1 |
| BP | GO:0051222 | positive regulation of protein transport | 0.710885174 | 0.743760945 | 0.703806659 | ISL1 | 1 |
| BP | GO:0030217 | T cell differentiation | 0.713334763 | 0.744981509 | 0.704961655 | TNFSF18 | 1 |
| BP | GO:0044262 | cellular carbohydrate metabolic process | 0.713334763 | 0.744981509 | 0.704961655 | ABHD10 | 1 |
| BP | GO:0048872 | homeostasis of number of cells | 0.713334763 | 0.744981509 | 0.704961655 | GPAM | 1 |
| BP | GO:0048193 | Golgi vesicle transport | 0.715763858 | 0.746623138 | 0.706515097 | STX6 | 1 |
| BP | GO:0097193 | intrinsic apoptotic signaling pathway | 0.715763858 | 0.746623138 | 0.706515097 | FHIT | 1 |
| BP | GO:0043588 | skin development | 0.720561241 | 0.751177547 | 0.710824847 | ATP8A2 | 1 |
| BP | GO:0009165 | nucleotide biosynthetic process | 0.722929864 | 0.752745859 | 0.71230891 | PDHA2 | 1 |
| BP | GO:0051607 | defense response to virus | 0.722929864 | 0.752745859 | 0.71230891 | GPAM | 1 |
| BP | GO:0140546 | defense response to symbiont | 0.72410673 | 0.752941256 | 0.71249381 | GPAM | 1 |
| BP | GO:0034504 | protein localization to nucleus | 0.725278661 | 0.752941256 | 0.71249381 | DCLK2 | 1 |
| BP | GO:0048608 | reproductive structure development | 0.725278661 | 0.752941256 | 0.71249381 | IRX5 | 1 |
| BP | GO:0071375 | cellular response to peptide hormone stimulus | 0.725278661 | 0.752941256 | 0.71249381 | MAX | 1 |
| BP | GO:1901293 | nucleoside phosphate biosynthetic process | 0.725278661 | 0.752941256 | 0.71249381 | PDHA2 | 1 |
| BP | GO:1903311 | regulation of mRNA metabolic process | 0.728765042 | 0.756110008 | 0.71549234 | PRDX6 | 1 |
| BP | GO:0061458 | reproductive system development | 0.729917434 | 0.756404622 | 0.715771127 | IRX5 | 1 |
| BP | GO:1904951 | positive regulation of establishment of protein localization | 0.729917434 | 0.756404622 | 0.715771127 | ISL1 | 1 |
| BP | GO:0019932 | second-messenger-mediated signaling | 0.734478854 | 0.760679051 | 0.719815937 | PLCG2 | 1 |
| BP | GO:0070372 | regulation of ERK1 and ERK2 cascade | 0.735607272 | 0.76139505 | 0.720493473 | ALKAL1 | 1 |
| BP | GO:0010948 | negative regulation of cell cycle process | 0.738964195 | 0.764415456 | 0.723351625 | FOXN3 | 1 |
| BP | GO:0022409 | positive regulation of cell-cell adhesion | 0.743374711 | 0.768521511 | 0.727237106 | GPAM | 1 |
| BP | GO:0015849 | organic acid transport | 0.745552295 | 0.770315597 | 0.728934815 | PLA2G2F | 1 |
| BP | GO:0006605 | protein targeting | 0.746634235 | 0.770519453 | 0.72912772 | MON1B | 1 |
| BP | GO:0140014 | mitotic nuclear division | 0.746634235 | 0.770519453 | 0.72912772 | SLF2 | 1 |
| BP | GO:0032409 | regulation of transporter activity | 0.747711633 | 0.771174461 | 0.729747541 | PLCG2 | 1 |
| BP | GO:0000377 | RNA splicing, via transesterification reactions with bulged adenosine as nucleophile | 0.750916759 | 0.773564159 | 0.732008867 | PRDX6 | 1 |
| BP | GO:0000398 | mRNA splicing, via spliceosome | 0.750916759 | 0.773564159 | 0.732008867 | PRDX6 | 1 |
| BP | GO:0000375 | RNA splicing, via transesterification reactions | 0.755127798 | 0.777442449 | 0.735678817 | PRDX6 | 1 |
| BP | GO:0051604 | protein maturation | 0.758239875 | 0.780185388 | 0.738274408 | FXN | 1 |
| BP | GO:1990778 | protein localization to cell periphery | 0.75926853 | 0.780782633 | 0.738839569 | FYB1 | 1 |
| BP | GO:0001933 | negative regulation of protein phosphorylation | 0.760292864 | 0.780914015 | 0.738963894 | SH3GL2 | 1 |
| BP | GO:0090287 | regulation of cellular response to growth factor stimulus | 0.760292864 | 0.780914015 | 0.738963894 | ROBO1 | 1 |
| BP | GO:0062012 | regulation of small molecule metabolic process | 0.761312895 | 0.781500921 | 0.739519271 | CNR1 | 1 |
| BP | GO:0070371 | ERK1 and ERK2 cascade | 0.762328639 | 0.782082738 | 0.740069834 | ALKAL1 | 1 |
| BP | GO:0062197 | cellular response to chemical stress | 0.767343694 | 0.7867644 | 0.7445 | FXN | 1 |
| BP | GO:0006936 | muscle contraction | 0.773223996 | 0.792327177 | 0.749763949 | ATP8A2 | 1 |
| BP | GO:1901990 | regulation of mitotic cell cycle phase transition | 0.776108874 | 0.79481579 | 0.752118875 | FOXN3 | 1 |
| BP | GO:0002831 | regulation of response to biotic stimulus | 0.782700095 | 0.801094926 | 0.758060701 | PLCG2 | 1 |
| BP | GO:0010639 | negative regulation of organelle organization | 0.787290445 | 0.805319997 | 0.762058804 | FXN | 1 |
| BP | GO:0006887 | exocytosis | 0.789998559 | 0.807615898 | 0.764231371 | CNR1 | 1 |
| BP | GO:0015711 | organic anion transport | 0.792672627 | 0.808879699 | 0.765427281 | PLA2G2F | 1 |
| BP | GO:0051098 | regulation of binding | 0.792672627 | 0.808879699 | 0.765427281 | ISL1 | 1 |
| BP | GO:0006338 | chromatin remodeling | 0.79355649 | 0.808879699 | 0.765427281 | SMARCAL1 | 1 |
| BP | GO:0051346 | negative regulation of hydrolase activity | 0.79355649 | 0.808879699 | 0.765427281 | ITIH2 | 1 |
| BP | GO:1901653 | cellular response to peptide | 0.79355649 | 0.808879699 | 0.765427281 | MAX | 1 |
| BP | GO:0002460 | adaptive immune response based on somatic recombination of immune receptors built from immunoglobulin superfamily domains | 0.795313069 | 0.810196121 | 0.766672986 | TNFSF18 | 1 |
| BP | GO:0050767 | regulation of neurogenesis | 0.79705489 | 0.811495978 | 0.767903016 | ROBO1 | 1 |
| BP | GO:0042326 | negative regulation of phosphorylation | 0.800494744 | 0.814522105 | 0.770766582 | SH3GL2 | 1 |
| BP | GO:0007178 | transmembrane receptor protein serine/threonine kinase signaling pathway | 0.808025342 | 0.821704697 | 0.777563331 | BMP3 | 1 |
| BP | GO:0001701 | in utero embryonic development | 0.810472626 | 0.823712552 | 0.779463325 | GNA12 | 1 |
| BP | GO:1904062 | regulation of cation transmembrane transport | 0.811281521 | 0.824053884 | 0.77978632 | PLCG2 | 1 |
| BP | GO:0051251 | positive regulation of lymphocyte activation | 0.816063806 | 0.828428409 | 0.78392585 | GPAM | 1 |
| BP | GO:0045786 | negative regulation of cell cycle | 0.819957485 | 0.831896295 | 0.787207444 | FOXN3 | 1 |
| BP | GO:0030099 | myeloid cell differentiation | 0.821492069 | 0.832968094 | 0.788221666 | NEDD9 | 1 |
| BP | GO:0009615 | response to virus | 0.823013738 | 0.833540658 | 0.788763472 | GPAM | 1 |
| BP | GO:0042692 | muscle cell differentiation | 0.823013738 | 0.833540658 | 0.788763472 | ISL1 | 1 |
| BP | GO:0016032 | viral process | 0.831878466 | 0.841540237 | 0.79633332 | MON1B | 1 |
| BP | GO:0043434 | response to peptide hormone | 0.831878466 | 0.841540237 | 0.79633332 | MAX | 1 |
| BP | GO:0010959 | regulation of metal ion transport | 0.83331268 | 0.842501851 | 0.797243276 | PLCG2 | 1 |
| BP | GO:0034655 | nucleobase-containing compound catabolic process | 0.836845579 | 0.845582946 | 0.800158857 | FHIT | 1 |
| BP | GO:0001503 | ossification | 0.837543235 | 0.845797284 | 0.800361682 | BMP3 | 1 |
| BP | GO:0034329 | cell junction assembly | 0.838237946 | 0.846008402 | 0.800561458 | DNM3 | 1 |
| BP | GO:0034470 | ncRNA processing | 0.844359798 | 0.851693555 | 0.805941209 | PTCD1 | 1 |
| BP | GO:0045936 | negative regulation of phosphate metabolic process | 0.84502572 | 0.851871993 | 0.806110062 | SH3GL2 | 1 |
| BP | GO:0010563 | negative regulation of phosphorus metabolic process | 0.845688828 | 0.852047391 | 0.806276037 | SH3GL2 | 1 |
| BP | GO:0072594 | establishment of protein localization to organelle | 0.850893814 | 0.856795967 | 0.810769524 | MON1B | 1 |
| BP | GO:0002696 | positive regulation of leukocyte activation | 0.851532125 | 0.856943363 | 0.810909001 | GPAM | 1 |
| BP | GO:0003012 | muscle system process | 0.854683432 | 0.859618094 | 0.813440047 | ATP8A2 | 1 |
| BP | GO:0051960 | regulation of nervous system development | 0.855305722 | 0.859747587 | 0.813562584 | ROBO1 | 1 |
| BP | GO:0002443 | leukocyte mediated immunity | 0.85958869 | 0.863554497 | 0.81716499 | PLCG2 | 1 |
| BP | GO:0050867 | positive regulation of cell activation | 0.861979645 | 0.865457373 | 0.818965645 | GPAM | 1 |
| BP | GO:0046700 | heterocycle catabolic process | 0.862571076 | 0.865552312 | 0.819055484 | FHIT | 1 |
| BP | GO:0008380 | RNA splicing | 0.863160004 | 0.865644632 | 0.819142845 | PRDX6 | 1 |
| BP | GO:0044772 | mitotic cell cycle phase transition | 0.865490899 | 0.867062951 | 0.820484973 | FOXN3 | 1 |
| BP | GO:0016570 | histone modification | 0.866067471 | 0.867062951 | 0.820484973 | ISL1 | 1 |
| BP | GO:0044270 | cellular nitrogen compound catabolic process | 0.866067471 | 0.867062951 | 0.820484973 | FHIT | 1 |
| BP | GO:0051051 | negative regulation of transport | 0.868913929 | 0.869413018 | 0.822708796 | CNR1 | 1 |
| BP | GO:0019439 | aromatic compound catabolic process | 0.874428845 | 0.874428845 | 0.827455176 | FHIT | 1 |
| CC | GO:0090571 | RNA polymerase II transcription repressor complex | 0.00162482 | 0.315215114 | 0.307860665 | TBX15/MAX | 2 |
| CC | GO:0031526 | brush border membrane | 0.022147061 | 0.415069312 | 0.405385112 | PLB1/GNA12 | 2 |
| CC | GO:0150034 | distal axon | 0.025900949 | 0.415069312 | 0.405385112 | UNC5C/SCGN/TULP1/CNR1 | 4 |
| CC | GO:0044304 | main axon | 0.026685346 | 0.415069312 | 0.405385112 | KCNQ3/DLG2 | 2 |
| CC | GO:0005775 | vacuolar lumen | 0.034170336 | 0.415069312 | 0.405385112 | FASLG/PRDX6/GPC5 | 3 |
| CC | GO:0017053 | transcription repressor complex | 0.038568317 | 0.415069312 | 0.405385112 | TBX15/MAX | 2 |
| CC | GO:0035861 | site of double-strand break | 0.038568317 | 0.415069312 | 0.405385112 | SMARCAL1/SLF2 | 2 |
| CC | GO:0031932 | TORC2 complex | 0.039550777 | 0.415069312 | 0.405385112 | RICTOR | 1 |
| CC | GO:0044224 | juxtaparanode region of axon | 0.039550777 | 0.415069312 | 0.405385112 | DLG2 | 1 |
| CC | GO:0030062 | mitochondrial tricarboxylic acid cycle enzyme complex | 0.04341985 | 0.415069312 | 0.405385112 | BCKDHB | 1 |
| CC | GO:0036157 | outer dynein arm | 0.04341985 | 0.415069312 | 0.405385112 | DNAH8 | 1 |
| CC | GO:0038201 | TOR complex | 0.04341985 | 0.415069312 | 0.405385112 | RICTOR | 1 |
| CC | GO:0005759 | mitochondrial matrix | 0.045304059 | 0.415069312 | 0.405385112 | ABHD10/PDHA2/BCKDHB/PTCD1/FXN | 5 |
| CC | GO:1990229 | iron-sulfur cluster assembly complex | 0.047273532 | 0.415069312 | 0.405385112 | FXN | 1 |
| CC | GO:0005741 | mitochondrial outer membrane | 0.051073743 | 0.415069312 | 0.405385112 | PPP2R2B/CNR1/GPAM | 3 |
| CC | GO:0000800 | lateral element | 0.051111882 | 0.415069312 | 0.405385112 | MEI4 | 1 |
| CC | GO:0106068 | SUMO ligase complex | 0.051111882 | 0.415069312 | 0.405385112 | SLF2 | 1 |
| CC | GO:1990531 | phospholipid-translocating ATPase complex | 0.054934961 | 0.415069312 | 0.405385112 | ATP8A2 | 1 |
| CC | GO:0043202 | lysosomal lumen | 0.059378817 | 0.415069312 | 0.405385112 | FASLG/GPC5 | 2 |
| CC | GO:0005604 | basement membrane | 0.060449082 | 0.415069312 | 0.405385112 | TINAG/ATRN | 2 |
| CC | GO:0032993 | protein-DNA complex | 0.062433062 | 0.415069312 | 0.405385112 | ST18/EYA1/MAX | 3 |
| CC | GO:0033268 | node of Ranvier | 0.062535544 | 0.415069312 | 0.405385112 | KCNQ3 | 1 |
| CC | GO:0045239 | tricarboxylic acid cycle enzyme complex | 0.062535544 | 0.415069312 | 0.405385112 | BCKDHB | 1 |
| CC | GO:0032588 | trans-Golgi network membrane | 0.06260951 | 0.415069312 | 0.405385112 | STX6/VPS53 | 2 |
| CC | GO:0005903 | brush border | 0.063699534 | 0.415069312 | 0.405385112 | PLB1/GNA12 | 2 |
| CC | GO:0097060 | synaptic membrane | 0.066220488 | 0.415069312 | 0.405385112 | DNM3/CNR1/DLG2/SHC4 | 4 |
| CC | GO:0000159 | protein phosphatase type 2A complex | 0.066313167 | 0.415069312 | 0.405385112 | PPP2R2B | 1 |
| CC | GO:0016010 | dystrophin-associated glycoprotein complex | 0.066313167 | 0.415069312 | 0.405385112 | SGCG | 1 |
| CC | GO:0090734 | site of DNA damage | 0.068123436 | 0.415069312 | 0.405385112 | SMARCAL1/SLF2 | 2 |
| CC | GO:0031968 | organelle outer membrane | 0.069210333 | 0.415069312 | 0.405385112 | PPP2R2B/CNR1/GPAM | 3 |
| CC | GO:0002177 | manchette | 0.070075757 | 0.415069312 | 0.405385112 | SPAG17 | 1 |
| CC | GO:0043083 | synaptic cleft | 0.070075757 | 0.415069312 | 0.405385112 | DNM3 | 1 |
| CC | GO:0019867 | outer membrane | 0.070604574 | 0.415069312 | 0.405385112 | PPP2R2B/CNR1/GPAM | 3 |
| CC | GO:0032838 | plasma membrane bounded cell projection cytoplasm | 0.077034936 | 0.422662977 | 0.412801606 | SPAG17/DNAH8/DLG2 | 3 |
| CC | GO:0043194 | axon initial segment | 0.077556076 | 0.422662977 | 0.412801606 | KCNQ3 | 1 |
| CC | GO:0043679 | axon terminus | 0.078432305 | 0.422662977 | 0.412801606 | SCGN/TULP1 | 2 |
| CC | GO:0090665 | glycoprotein complex | 0.084976969 | 0.441464651 | 0.431164608 | SGCG | 1 |
| CC | GO:0005925 | focal adhesion | 0.090563197 | 0.441464651 | 0.431164608 | RPL37A/NEDD9/GNA12/ARPC1B | 4 |
| CC | GO:0005858 | axonemal dynein complex | 0.092338902 | 0.441464651 | 0.431164608 | DNAH8 | 1 |
| CC | GO:0045211 | postsynaptic membrane | 0.094582357 | 0.441464651 | 0.431164608 | DNM3/DLG2/SHC4 | 3 |
| CC | GO:0030055 | cell-substrate junction | 0.096625429 | 0.441464651 | 0.431164608 | RPL37A/NEDD9/GNA12/ARPC1B | 4 |
| CC | GO:0062023 | collagen-containing extracellular matrix | 0.097242054 | 0.441464651 | 0.431164608 | TINAG/ITIH2/GPC5/ATRN | 4 |
| CC | GO:0044306 | neuron projection terminus | 0.097850412 | 0.441464651 | 0.431164608 | SCGN/TULP1 | 2 |
| CC | GO:0099568 | cytoplasmic region | 0.107674015 | 0.474744519 | 0.463668005 | SPAG17/DNAH8/DLG2 | 3 |
| CC | GO:0030285 | integral component of synaptic vesicle membrane | 0.114075554 | 0.490479981 | 0.479036335 | STX6 | 1 |
| CC | GO:0071339 | MLL1 complex | 0.11764801 | 0.490479981 | 0.479036335 | MAX | 1 |
| CC | GO:0044665 | MLL1/2 complex | 0.12120624 | 0.490479981 | 0.479036335 | MAX | 1 |
| CC | GO:0043596 | nuclear replication fork | 0.124750299 | 0.490479981 | 0.479036335 | SMARCAL1 | 1 |
| CC | GO:0005834 | heterotrimeric G-protein complex | 0.128280243 | 0.490479981 | 0.479036335 | GNA12 | 1 |
| CC | GO:0005930 | axoneme | 0.130490117 | 0.490479981 | 0.479036335 | SPAG17/DNAH8 | 2 |
| CC | GO:0005788 | endoplasmic reticulum lumen | 0.131250422 | 0.490479981 | 0.479036335 | DNAJB9/ADAMTSL1/ITIH2 | 3 |
| CC | GO:0097014 | ciliary plasm | 0.131844274 | 0.490479981 | 0.479036335 | SPAG17/DNAH8 | 2 |
| CC | GO:0098862 | cluster of actin-based cell projections | 0.134562404 | 0.490479981 | 0.479036335 | PLB1/GNA12 | 2 |
| CC | GO:0043025 | neuronal cell body | 0.140394583 | 0.490479981 | 0.479036335 | UNC5C/GNA12/PDE1C/DLG2 | 4 |
| CC | GO:0098978 | glutamatergic synapse | 0.142270841 | 0.490479981 | 0.479036335 | DNM3/CNR1/SH3GL2 | 3 |
| CC | GO:0030426 | growth cone | 0.145558941 | 0.490479981 | 0.479036335 | UNC5C/CNR1 | 2 |
| CC | GO:0000795 | synaptonemal complex | 0.149166592 | 0.490479981 | 0.479036335 | MEI4 | 1 |
| CC | GO:0099086 | synaptonemal structure | 0.149166592 | 0.490479981 | 0.479036335 | MEI4 | 1 |
| CC | GO:1905360 | GTPase complex | 0.149166592 | 0.490479981 | 0.479036335 | GNA12 | 1 |
| CC | GO:0030427 | site of polarized growth | 0.15392638 | 0.496189289 | 0.484612436 | UNC5C/CNR1 | 2 |
| CC | GO:0098563 | intrinsic component of synaptic vesicle membrane | 0.156018282 | 0.496189289 | 0.484612436 | STX6 | 1 |
| CC | GO:0031253 | cell projection membrane | 0.161295686 | 0.500046394 | 0.488379549 | PLB1/GNA12/PLCG2 | 3 |
| CC | GO:0005912 | adherens junction | 0.1623862 | 0.500046394 | 0.488379549 | TJP2/DLG2 | 2 |
| CC | GO:0001772 | immunological synapse | 0.166193777 | 0.503774888 | 0.492021051 | NEDD9 | 1 |
| CC | GO:0043195 | terminal bouton | 0.172910038 | 0.516069961 | 0.504029262 | SCGN | 1 |
| CC | GO:0031201 | SNARE complex | 0.176248107 | 0.518062618 | 0.505975427 | STX6 | 1 |
| CC | GO:0055037 | recycling endosome | 0.189684631 | 0.549236095 | 0.53642158 | STX6/VPS53 | 2 |
| CC | GO:0030286 | dynein complex | 0.195998951 | 0.551178 | 0.538318177 | DNAH8 | 1 |
| CC | GO:0030027 | lamellipodium | 0.196973956 | 0.551178 | 0.538318177 | UNC5C/NEDD9 | 2 |
| CC | GO:0008287 | protein serine/threonine phosphatase complex | 0.202478069 | 0.551178 | 0.538318177 | PPP2R2B | 1 |
| CC | GO:0031301 | integral component of organelle membrane | 0.207649107 | 0.551178 | 0.538318177 | STX6/XXYLT1/CNR1 | 3 |
| CC | GO:0030136 | clathrin-coated vesicle | 0.208708404 | 0.551178 | 0.538318177 | STX6/SH3GL2 | 2 |
| CC | GO:1903293 | phosphatase complex | 0.208905622 | 0.551178 | 0.538318177 | PPP2R2B | 1 |
| CC | GO:0022625 | cytosolic large ribosomal subunit | 0.212100188 | 0.551178 | 0.538318177 | RPL37A | 1 |
| CC | GO:0035097 | histone methyltransferase complex | 0.221607649 | 0.551178 | 0.538318177 | MAX | 1 |
| CC | GO:0046658 | anchored component of plasma membrane | 0.221607649 | 0.551178 | 0.538318177 | GPC5 | 1 |
| CC | GO:0099023 | vesicle tethering complex | 0.221607649 | 0.551178 | 0.538318177 | VPS53 | 1 |
| CC | GO:1904115 | axon cytoplasm | 0.221607649 | 0.551178 | 0.538318177 | DLG2 | 1 |
| CC | GO:0030658 | transport vesicle membrane | 0.224959228 | 0.552431522 | 0.539542452 | STX6/SCGN | 2 |
| CC | GO:0016328 | lateral plasma membrane | 0.227882925 | 0.552616094 | 0.539722718 | GNA12 | 1 |
| CC | GO:0005657 | replication fork | 0.231001803 | 0.553263577 | 0.540355094 | SMARCAL1 | 1 |
| CC | GO:0031300 | intrinsic component of organelle membrane | 0.23935928 | 0.555282205 | 0.542326624 | STX6/XXYLT1/CNR1 | 3 |
| CC | GO:0031594 | neuromuscular junction | 0.240283982 | 0.555282205 | 0.542326624 | DLG2 | 1 |
| CC | GO:0031252 | cell leading edge | 0.24043147 | 0.555282205 | 0.542326624 | UNC5C/NEDD9/PLCG2 | 3 |
| CC | GO:0001917 | photoreceptor inner segment | 0.249455504 | 0.555512591 | 0.542551635 | TULP1 | 1 |
| CC | GO:0030669 | clathrin-coated endocytic vesicle membrane | 0.252488313 | 0.555512591 | 0.542551635 | SH3GL2 | 1 |
| CC | GO:0044291 | cell-cell contact zone | 0.252488313 | 0.555512591 | 0.542551635 | TJP2 | 1 |
| CC | GO:0098982 | GABA-ergic synapse | 0.252488313 | 0.555512591 | 0.542551635 | CNR1 | 1 |
| CC | GO:0099056 | integral component of presynaptic membrane | 0.25550902 | 0.555512591 | 0.542551635 | CNR1 | 1 |
| CC | GO:0031514 | motile cilium | 0.257712027 | 0.555512591 | 0.542551635 | SPAG17/DNAH8 | 2 |
| CC | GO:0000228 | nuclear chromosome | 0.265178566 | 0.564272654 | 0.551107312 | SMARCAL1/MEI4 | 2 |
| CC | GO:0000794 | condensed nuclear chromosome | 0.273381768 | 0.564272654 | 0.551107312 | MEI4 | 1 |
| CC | GO:0008076 | voltage-gated potassium channel complex | 0.273381768 | 0.564272654 | 0.551107312 | KCNQ3 | 1 |
| CC | GO:0033116 | endoplasmic reticulum-Golgi intermediate compartment membrane | 0.276319083 | 0.564272654 | 0.551107312 | ROBO1 | 1 |
| CC | GO:0098889 | intrinsic component of presynaptic membrane | 0.276319083 | 0.564272654 | 0.551107312 | CNR1 | 1 |
| CC | GO:0005901 | caveola | 0.28215858 | 0.568576143 | 0.555310395 | FASLG | 1 |
| CC | GO:0005802 | trans-Golgi network | 0.284591276 | 0.568576143 | 0.555310395 | STX6/VPS53 | 2 |
| CC | GO:0005874 | microtubule | 0.289389847 | 0.568576143 | 0.555310395 | SPAG17/DNM3/DNAH8 | 3 |
| CC | GO:0000793 | condensed chromosome | 0.300987731 | 0.568576143 | 0.555310395 | MEI4/SLF2 | 2 |
| CC | GO:0034705 | potassium channel complex | 0.30223275 | 0.568576143 | 0.555310395 | KCNQ3 | 1 |
| CC | GO:0098839 | postsynaptic density membrane | 0.30223275 | 0.568576143 | 0.555310395 | DLG2 | 1 |
| CC | GO:0045334 | clathrin-coated endocytic vesicle | 0.305054862 | 0.568576143 | 0.555310395 | SH3GL2 | 1 |
| CC | GO:0120111 | neuron projection cytoplasm | 0.305054862 | 0.568576143 | 0.555310395 | DLG2 | 1 |
| CC | GO:0035578 | azurophil granule lumen | 0.307865702 | 0.568576143 | 0.555310395 | PRDX6 | 1 |
| CC | GO:0032592 | integral component of mitochondrial membrane | 0.310665315 | 0.568576143 | 0.555310395 | CNR1 | 1 |
| CC | GO:0034708 | methyltransferase complex | 0.310665315 | 0.568576143 | 0.555310395 | MAX | 1 |
| CC | GO:0098573 | intrinsic component of mitochondrial membrane | 0.316231036 | 0.573353467 | 0.559976256 | CNR1 | 1 |
| CC | GO:0001750 | photoreceptor outer segment | 0.324496512 | 0.577544251 | 0.564069263 | TULP1 | 1 |
| CC | GO:0032587 | ruffle membrane | 0.324496512 | 0.577544251 | 0.564069263 | PLCG2 | 1 |
| CC | GO:0031234 | extrinsic component of cytoplasmic side of plasma membrane | 0.329951935 | 0.58077027 | 0.567220014 | GNA12 | 1 |
| CC | GO:0016605 | PML body | 0.3460585 | 0.58077027 | 0.567220014 | MAX | 1 |
| CC | GO:0022626 | cytosolic ribosome | 0.3460585 | 0.58077027 | 0.567220014 | RPL37A | 1 |
| CC | GO:0005796 | Golgi lumen | 0.348705501 | 0.58077027 | 0.567220014 | GPC5 | 1 |
| CC | GO:0030175 | filopodium | 0.3539678 | 0.58077027 | 0.567220014 | UNC5C | 1 |
| CC | GO:0030135 | coated vesicle | 0.355575679 | 0.58077027 | 0.567220014 | STX6/SH3GL2 | 2 |
| CC | GO:0019898 | extrinsic component of membrane | 0.36430521 | 0.58077027 | 0.567220014 | GNA12/RGS6 | 2 |
| CC | GO:0044853 | plasma membrane raft | 0.366940563 | 0.58077027 | 0.567220014 | FASLG | 1 |
| CC | GO:0030672 | synaptic vesicle membrane | 0.369504076 | 0.58077027 | 0.567220014 | STX6 | 1 |
| CC | GO:0099501 | exocytic vesicle membrane | 0.369504076 | 0.58077027 | 0.567220014 | STX6 | 1 |
| CC | GO:0014069 | postsynaptic density | 0.371546924 | 0.58077027 | 0.567220014 | DNM3/DLG2 | 2 |
| CC | GO:0015934 | large ribosomal subunit | 0.372057337 | 0.58077027 | 0.567220014 | RPL37A | 1 |
| CC | GO:0099634 | postsynaptic specialization membrane | 0.372057337 | 0.58077027 | 0.567220014 | DLG2 | 1 |
| CC | GO:0060205 | cytoplasmic vesicle lumen | 0.377317538 | 0.58077027 | 0.567220014 | FASLG/PRDX6 | 2 |
| CC | GO:0045121 | membrane raft | 0.378756921 | 0.58077027 | 0.567220014 | FASLG/CNR1 | 2 |
| CC | GO:0031983 | vesicle lumen | 0.380194971 | 0.58077027 | 0.567220014 | FASLG/PRDX6 | 2 |
| CC | GO:0032279 | asymmetric synapse | 0.380194971 | 0.58077027 | 0.567220014 | DNM3/DLG2 | 2 |
| CC | GO:0098857 | membrane microdomain | 0.380194971 | 0.58077027 | 0.567220014 | FASLG/CNR1 | 2 |
| CC | GO:0097733 | photoreceptor cell cilium | 0.389646505 | 0.588571295 | 0.57483903 | TULP1 | 1 |
| CC | GO:1990204 | oxidoreductase complex | 0.397034946 | 0.588571295 | 0.57483903 | BCKDHB | 1 |
| CC | GO:0099572 | postsynaptic specialization | 0.401597401 | 0.588571295 | 0.57483903 | DNM3/DLG2 | 2 |
| CC | GO:0005923 | bicellular tight junction | 0.401911435 | 0.588571295 | 0.57483903 | TJP2 | 1 |
| CC | GO:0030139 | endocytic vesicle | 0.403012482 | 0.588571295 | 0.57483903 | STX6/SH3GL2 | 2 |
| CC | GO:0030665 | clathrin-coated vesicle membrane | 0.413932923 | 0.588571295 | 0.57483903 | SH3GL2 | 1 |
| CC | GO:0070160 | tight junction | 0.413932923 | 0.588571295 | 0.57483903 | TJP2 | 1 |
| CC | GO:0098984 | neuron to neuron synapse | 0.414277104 | 0.588571295 | 0.57483903 | DNM3/DLG2 | 2 |
| CC | GO:0001669 | acrosomal vesicle | 0.416308429 | 0.588571295 | 0.57483903 | SPAG17 | 1 |
| CC | GO:0005793 | endoplasmic reticulum-Golgi intermediate compartment | 0.416308429 | 0.588571295 | 0.57483903 | ROBO1 | 1 |
| CC | GO:0097731 | 9+0 non-motile cilium | 0.418674427 | 0.588571295 | 0.57483903 | TULP1 | 1 |
| CC | GO:0042383 | sarcolemma | 0.423378045 | 0.589934438 | 0.576170368 | SGCG | 1 |
| CC | GO:0045335 | phagocytic vesicle | 0.430363093 | 0.589934438 | 0.576170368 | STX6 | 1 |
| CC | GO:0001650 | fibrillar center | 0.444083464 | 0.589934438 | 0.576170368 | FHIT | 1 |
| CC | GO:0072562 | blood microparticle | 0.444083464 | 0.589934438 | 0.576170368 | ITIH2 | 1 |
| CC | GO:0042734 | presynaptic membrane | 0.450820752 | 0.589934438 | 0.576170368 | CNR1 | 1 |
| CC | GO:0036126 | sperm flagellum | 0.455267417 | 0.589934438 | 0.576170368 | DNAH8 | 1 |
| CC | GO:0043296 | apical junction complex | 0.457477397 | 0.589934438 | 0.576170368 | TJP2 | 1 |
| CC | GO:0098791 | Golgi apparatus subcompartment | 0.463624069 | 0.589934438 | 0.576170368 | STX6/VPS53 | 2 |
| CC | GO:0043204 | perikaryon | 0.464054353 | 0.589934438 | 0.576170368 | DLG2 | 1 |
| CC | GO:0005766 | primary lysosome | 0.466229126 | 0.589934438 | 0.576170368 | PRDX6 | 1 |
| CC | GO:0042582 | azurophil granule | 0.466229126 | 0.589934438 | 0.576170368 | PRDX6 | 1 |
| CC | GO:0099699 | integral component of synaptic membrane | 0.466229126 | 0.589934438 | 0.576170368 | CNR1 | 1 |
| CC | GO:0030176 | integral component of endoplasmic reticulum membrane | 0.48541402 | 0.589934438 | 0.576170368 | XXYLT1 | 1 |
| CC | GO:0036064 | ciliary basal body | 0.48541402 | 0.589934438 | 0.576170368 | NEDD9 | 1 |
| CC | GO:1990351 | transporter complex | 0.487393654 | 0.589934438 | 0.576170368 | KCNQ3/ATP8A2 | 2 |
| CC | GO:0097729 | 9+2 motile cilium | 0.487503179 | 0.589934438 | 0.576170368 | DNAH8 | 1 |
| CC | GO:0005875 | microtubule associated complex | 0.489583962 | 0.589934438 | 0.576170368 | DNAH8 | 1 |
| CC | GO:0099240 | intrinsic component of synaptic membrane | 0.489583962 | 0.589934438 | 0.576170368 | CNR1 | 1 |
| CC | GO:0000781 | chromosome, telomeric region | 0.493720532 | 0.589934438 | 0.576170368 | SLF2 | 1 |
| CC | GO:0097730 | non-motile cilium | 0.493720532 | 0.589934438 | 0.576170368 | TULP1 | 1 |
| CC | GO:0031225 | anchored component of membrane | 0.495776385 | 0.589934438 | 0.576170368 | GPC5 | 1 |
| CC | GO:0005769 | early endosome | 0.499036227 | 0.589934438 | 0.576170368 | STX6/SH3GL2 | 2 |
| CC | GO:0000922 | spindle pole | 0.501894608 | 0.589934438 | 0.576170368 | NEDD9 | 1 |
| CC | GO:0019897 | extrinsic component of plasma membrane | 0.501894608 | 0.589934438 | 0.576170368 | GNA12 | 1 |
| CC | GO:0031227 | intrinsic component of endoplasmic reticulum membrane | 0.501894608 | 0.589934438 | 0.576170368 | XXYLT1 | 1 |
| CC | GO:0009898 | cytoplasmic side of plasma membrane | 0.503917679 | 0.589934438 | 0.576170368 | GNA12 | 1 |
| CC | GO:0043197 | dendritic spine | 0.50793951 | 0.589934438 | 0.576170368 | DNM3 | 1 |
| CC | GO:0044309 | neuron spine | 0.509938334 | 0.589934438 | 0.576170368 | DNM3 | 1 |
| CC | GO:0030133 | transport vesicle | 0.510512485 | 0.589934438 | 0.576170368 | STX6/SCGN | 2 |
| CC | GO:0031256 | leading edge membrane | 0.51192914 | 0.589934438 | 0.576170368 | PLCG2 | 1 |
| CC | GO:0001726 | ruffle | 0.513911959 | 0.589934438 | 0.576170368 | PLCG2 | 1 |
| CC | GO:0072686 | mitotic spindle | 0.523707356 | 0.597642512 | 0.583698601 | NEDD9 | 1 |
| CC | GO:0044391 | ribosomal subunit | 0.533307779 | 0.605039235 | 0.590922747 | RPL37A | 1 |
| CC | GO:0030666 | endocytic vesicle membrane | 0.544576313 | 0.614231423 | 0.599900467 | SH3GL2 | 1 |
| CC | GO:0030662 | coated vesicle membrane | 0.551938962 | 0.618937333 | 0.604496581 | SH3GL2 | 1 |
| CC | GO:0098562 | cytoplasmic side of membrane | 0.56276308 | 0.627448492 | 0.612809162 | GNA12 | 1 |
| CC | GO:0008021 | synaptic vesicle | 0.569835284 | 0.631703114 | 0.616964518 | STX6 | 1 |
| CC | GO:0034703 | cation channel complex | 0.593709874 | 0.652546109 | 0.637321214 | KCNQ3 | 1 |
| CC | GO:0098858 | actin-based cell projection | 0.595364234 | 0.652546109 | 0.637321214 | UNC5C | 1 |
| CC | GO:0070382 | exocytic vesicle | 0.600287504 | 0.654245932 | 0.638981377 | STX6 | 1 |
| CC | GO:0016323 | basolateral plasma membrane | 0.605151613 | 0.655862642 | 0.640560366 | DLG2 | 1 |
| CC | GO:0005840 | ribosome | 0.60995726 | 0.657398381 | 0.642060274 | RPL37A | 1 |
| CC | GO:0090575 | RNA polymerase II transcription regulator complex | 0.63611805 | 0.681806087 | 0.665898511 | MAX | 1 |
| CC | GO:0009925 | basal plasma membrane | 0.643477134 | 0.685904198 | 0.669901007 | DLG2 | 1 |
| CC | GO:0045178 | basal part of cell | 0.66877328 | 0.708972767 | 0.692431352 | DLG2 | 1 |
| CC | GO:0005770 | late endosome | 0.689765588 | 0.727252848 | 0.710284931 | MON1B | 1 |
| CC | GO:0098798 | mitochondrial protein-containing complex | 0.698531346 | 0.729860818 | 0.712832052 | BCKDHB | 1 |
| CC | GO:0034702 | ion channel complex | 0.699763465 | 0.729860818 | 0.712832052 | KCNQ3 | 1 |
| CC | GO:0005938 | cell cortex | 0.718814324 | 0.745721812 | 0.728322985 | NEDD9 | 1 |
| CC | GO:0034774 | secretory granule lumen | 0.73010964 | 0.753411011 | 0.735832784 | PRDX6 | 1 |
| CC | GO:0016324 | apical plasma membrane | 0.776559005 | 0.797102894 | 0.778505268 | PLB1 | 1 |
| CC | GO:1902495 | transmembrane transporter complex | 0.786440166 | 0.802996801 | 0.784261661 | KCNQ3 | 1 |
| CC | GO:0098687 | chromosomal region | 0.794202766 | 0.806677155 | 0.787856147 | SLF2 | 1 |
| CC | GO:0005819 | spindle | 0.824019778 | 0.832603317 | 0.813177413 | NEDD9 | 1 |
| CC | GO:0045177 | apical part of cell | 0.830431624 | 0.834734378 | 0.815258753 | PLB1 | 1 |
| CC | GO:0009897 | external side of plasma membrane | 0.848314893 | 0.848314893 | 0.828522413 | FASLG | 1 |
| MF | GO:1990782 | protein tyrosine kinase binding | 0.000158701 | 0.035707824 | 0.033410829 | NEDD9/ALKAL1/TJP2/SHC4/PLCG2 | 5 |
| MF | GO:0004623 | phospholipase A2 activity | 0.000488636 | 0.054971566 | 0.051435383 | PLA2G2F/PRDX6/PLB1 | 3 |
| MF | GO:0004620 | phospholipase activity | 0.00119507 | 0.089630271 | 0.083864581 | PLA2G2F/PRDX6/PLB1/PLCG2 | 4 |
| MF | GO:0016298 | lipase activity | 0.002359547 | 0.132724533 | 0.124186698 | PLA2G2F/PRDX6/PLB1/PLCG2 | 4 |
| MF | GO:0050145 | nucleoside monophosphate kinase activity | 0.003277989 | 0.147509484 | 0.13802057 | TJP2/DLG2 | 2 |
| MF | GO:0052689 | carboxylic ester hydrolase activity | 0.00413661 | 0.15512287 | 0.145144206 | PLA2G2F/PRDX6/PLB1/ABHD10 | 4 |
| MF | GO:0005154 | epidermal growth factor receptor binding | 0.008787358 | 0.282450787 | 0.264281438 | FAM83B/VAV2 | 2 |
| MF | GO:0016776 | phosphotransferase activity, phosphate group as acceptor | 0.012129722 | 0.283503444 | 0.26526638 | TJP2/DLG2 | 2 |
| MF | GO:0001227 | DNA-binding transcription repressor activity, RNA polymerase II-specific | 0.012564798 | 0.283503444 | 0.26526638 | TBX15/IRX1/ZFP37/ETV6/MAX | 5 |
| MF | GO:0001217 | DNA-binding transcription repressor activity | 0.013192999 | 0.283503444 | 0.26526638 | TBX15/IRX1/ZFP37/ETV6/MAX | 5 |
| MF | GO:0019205 | nucleobase-containing compound kinase activity | 0.014619535 | 0.283503444 | 0.26526638 | TJP2/DLG2 | 2 |
| MF | GO:0001784 | phosphotyrosine residue binding | 0.016618818 | 0.283503444 | 0.26526638 | VAV2/PLCG2 | 2 |
| MF | GO:0016903 | oxidoreductase activity, acting on the aldehyde or oxo group of donors | 0.017309754 | 0.283503444 | 0.26526638 | PDHA2/BCKDHB | 2 |
| MF | GO:0032813 | tumor necrosis factor receptor superfamily binding | 0.018727704 | 0.283503444 | 0.26526638 | FASLG/TNFSF18 | 2 |
| MF | GO:0005125 | cytokine activity | 0.01890023 | 0.283503444 | 0.26526638 | FASLG/TNFSF18/BMP3/ALKAL1 | 4 |
| MF | GO:0008374 | O-acyltransferase activity | 0.022477794 | 0.316093977 | 0.295760447 | PRDX6/GPAM | 2 |
| MF | GO:0045309 | protein phosphorylated amino acid binding | 0.025681678 | 0.324986325 | 0.304080772 | VAV2/PLCG2 | 2 |
| MF | GO:0070567 | cytidylyltransferase activity | 0.042053041 | 0.324986325 | 0.304080772 | CMAS | 1 |
| MF | GO:0030971 | receptor tyrosine kinase binding | 0.043160255 | 0.324986325 | 0.304080772 | ALKAL1/SHC4 | 2 |
| MF | GO:0008199 | ferric iron binding | 0.046161054 | 0.324986325 | 0.304080772 | FXN | 1 |
| MF | GO:0035252 | UDP-xylosyltransferase activity | 0.046161054 | 0.324986325 | 0.304080772 | XXYLT1 | 1 |
| MF | GO:0035256 | G protein-coupled glutamate receptor binding | 0.046161054 | 0.324986325 | 0.304080772 | DNM3 | 1 |
| MF | GO:0036312 | phosphatidylinositol 3-kinase regulatory subunit binding | 0.046161054 | 0.324986325 | 0.304080772 | FAM83B | 1 |
| MF | GO:0042285 | xylosyltransferase activity | 0.046161054 | 0.324986325 | 0.304080772 | XXYLT1 | 1 |
| MF | GO:0102545 | phosphatidyl phospholipase B activity | 0.046161054 | 0.324986325 | 0.304080772 | PLB1 | 1 |
| MF | GO:0016530 | metallochaperone activity | 0.050251672 | 0.324986325 | 0.304080772 | FXN | 1 |
| MF | GO:0051378 | serotonin binding | 0.050251672 | 0.324986325 | 0.304080772 | HTR1E | 1 |
| MF | GO:0043176 | amine binding | 0.054324968 | 0.324986325 | 0.304080772 | HTR1E | 1 |
| MF | GO:0050998 | nitric-oxide synthase binding | 0.054324968 | 0.324986325 | 0.304080772 | DNM3 | 1 |
| MF | GO:0099186 | structural constituent of postsynapse | 0.054324968 | 0.324986325 | 0.304080772 | DNM3 | 1 |
| MF | GO:0140666 | annealing activity | 0.054324968 | 0.324986325 | 0.304080772 | SMARCAL1 | 1 |
| MF | GO:0008081 | phosphoric diester hydrolase activity | 0.054802074 | 0.324986325 | 0.304080772 | PDE1C/PLCG2 | 2 |
| MF | GO:0008474 | palmitoyl-(protein) hydrolase activity | 0.058381015 | 0.324986325 | 0.304080772 | ABHD10 | 1 |
| MF | GO:0015386 | potassium:proton antiporter activity | 0.058381015 | 0.324986325 | 0.304080772 | SLC9C2 | 1 |
| MF | GO:0098599 | palmitoyl hydrolase activity | 0.058381015 | 0.324986325 | 0.304080772 | ABHD10 | 1 |
| MF | GO:0140333 | glycerophospholipid flippase activity | 0.058381015 | 0.324986325 | 0.304080772 | ATP8A2 | 1 |
| MF | GO:0051219 | phosphoprotein binding | 0.059280873 | 0.324986325 | 0.304080772 | VAV2/PLCG2 | 2 |
| MF | GO:0015385 | sodium:proton antiporter activity | 0.062419885 | 0.324986325 | 0.304080772 | SLC9C2 | 1 |
| MF | GO:0047555 | 3',5'-cyclic-GMP phosphodiesterase activity | 0.062419885 | 0.324986325 | 0.304080772 | PDE1C | 1 |
| MF | GO:0050780 | dopamine receptor binding | 0.062419885 | 0.324986325 | 0.304080772 | GNA12 | 1 |
| MF | GO:0070700 | BMP receptor binding | 0.062419885 | 0.324986325 | 0.304080772 | BMP3 | 1 |
| MF | GO:0019888 | protein phosphatase regulator activity | 0.063881769 | 0.324986325 | 0.304080772 | PPP2R2B/GNA12 | 2 |
| MF | GO:0016722 | oxidoreductase activity, acting on metal ions | 0.066441649 | 0.324986325 | 0.304080772 | FXN | 1 |
| MF | GO:0030275 | LRR domain binding | 0.066441649 | 0.324986325 | 0.304080772 | ROBO1 | 1 |
| MF | GO:0047498 | calcium-dependent phospholipase A2 activity | 0.066441649 | 0.324986325 | 0.304080772 | PLA2G2F | 1 |
| MF | GO:0140328 | floppase activity | 0.066441649 | 0.324986325 | 0.304080772 | ATP8A2 | 1 |
| MF | GO:0004115 | 3',5'-cyclic-AMP phosphodiesterase activity | 0.070446379 | 0.330217401 | 0.308975346 | PDE1C | 1 |
| MF | GO:0140327 | flippase activity | 0.070446379 | 0.330217401 | 0.308975346 | ATP8A2 | 1 |
| MF | GO:0008569 | minus-end-directed microtubule motor activity | 0.074434146 | 0.330256032 | 0.309011492 | DNAH8 | 1 |
| MF | GO:0046527 | glucosyltransferase activity | 0.074434146 | 0.330256032 | 0.309011492 | ALG6 | 1 |
| MF | GO:0022821 | solute:potassium antiporter activity | 0.078405022 | 0.330256032 | 0.309011492 | SLC9C2 | 1 |
| MF | GO:0098918 | structural constituent of synapse | 0.078405022 | 0.330256032 | 0.309011492 | DNM3 | 1 |
| MF | GO:0051139 | metal cation:proton antiporter activity | 0.082359076 | 0.330256032 | 0.309011492 | SLC9C2 | 1 |
| MF | GO:0019208 | phosphatase regulator activity | 0.085956876 | 0.330256032 | 0.309011492 | PPP2R2B/GNA12 | 2 |
| MF | GO:0005123 | death receptor binding | 0.08629638 | 0.330256032 | 0.309011492 | FASLG | 1 |
| MF | GO:0008373 | sialyltransferase activity | 0.08629638 | 0.330256032 | 0.309011492 | ST8SIA1 | 1 |
| MF | GO:0030295 | protein kinase activator activity | 0.087243383 | 0.330256032 | 0.309011492 | RICTOR/ALKAL1 | 2 |
| MF | GO:0004602 | glutathione peroxidase activity | 0.090217002 | 0.330256032 | 0.309011492 | PRDX6 | 1 |
| MF | GO:0031683 | G-protein beta/gamma-subunit complex binding | 0.090217002 | 0.330256032 | 0.309011492 | GNA12 | 1 |
| MF | GO:0019209 | kinase activator activity | 0.095082347 | 0.330256032 | 0.309011492 | RICTOR/ALKAL1 | 2 |
| MF | GO:0004114 | 3',5'-cyclic-nucleotide phosphodiesterase activity | 0.098008484 | 0.330256032 | 0.309011492 | PDE1C | 1 |
| MF | GO:0004622 | lysophospholipase activity | 0.098008484 | 0.330256032 | 0.309011492 | PLB1 | 1 |
| MF | GO:0005540 | hyaluronic acid binding | 0.098008484 | 0.330256032 | 0.309011492 | ITIH2 | 1 |
| MF | GO:0051537 | 2 iron, 2 sulfur cluster binding | 0.098008484 | 0.330256032 | 0.309011492 | FXN | 1 |
| MF | GO:0051959 | dynein light intermediate chain binding | 0.098008484 | 0.330256032 | 0.309011492 | DNAH8 | 1 |
| MF | GO:0016779 | nucleotidyltransferase activity | 0.101764114 | 0.330256032 | 0.309011492 | FHIT/CMAS | 2 |
| MF | GO:0004112 | cyclic-nucleotide phosphodiesterase activity | 0.105734077 | 0.330256032 | 0.309011492 | PDE1C | 1 |
| MF | GO:0004435 | phosphatidylinositol phospholipase C activity | 0.105734077 | 0.330256032 | 0.309011492 | PLCG2 | 1 |
| MF | GO:0004806 | triglyceride lipase activity | 0.105734077 | 0.330256032 | 0.309011492 | PLB1 | 1 |
| MF | GO:0070696 | transmembrane receptor protein serine/threonine kinase binding | 0.105734077 | 0.330256032 | 0.309011492 | BMP3 | 1 |
| MF | GO:0016757 | glycosyltransferase activity | 0.106978061 | 0.330256032 | 0.309011492 | ALG6/XXYLT1/ST8SIA1 | 3 |
| MF | GO:0008198 | ferrous iron binding | 0.109572337 | 0.330256032 | 0.309011492 | FXN | 1 |
| MF | GO:0140326 | ATPase-coupled intramembrane lipid transporter activity | 0.109572337 | 0.330256032 | 0.309011492 | ATP8A2 | 1 |
| MF | GO:0004629 | phospholipase C activity | 0.117200125 | 0.330256032 | 0.309011492 | PLCG2 | 1 |
| MF | GO:0051787 | misfolded protein binding | 0.117200125 | 0.330256032 | 0.309011492 | DNAJB9 | 1 |
| MF | GO:0005451 | monovalent cation:proton antiporter activity | 0.120989789 | 0.330256032 | 0.309011492 | SLC9C2 | 1 |
| MF | GO:0015299 | solute:proton antiporter activity | 0.120989789 | 0.330256032 | 0.309011492 | SLC9C2 | 1 |
| MF | GO:0030296 | protein tyrosine kinase activator activity | 0.120989789 | 0.330256032 | 0.309011492 | ALKAL1 | 1 |
| MF | GO:0043548 | phosphatidylinositol 3-kinase binding | 0.120989789 | 0.330256032 | 0.309011492 | FAM83B | 1 |
| MF | GO:0051721 | protein phosphatase 2A binding | 0.120989789 | 0.330256032 | 0.309011492 | GNA12 | 1 |
| MF | GO:0070851 | growth factor receptor binding | 0.121120604 | 0.330256032 | 0.309011492 | FAM83B/VAV2 | 2 |
| MF | GO:0005164 | tumor necrosis factor receptor binding | 0.12476339 | 0.330256032 | 0.309011492 | FASLG | 1 |
| MF | GO:0033612 | receptor serine/threonine kinase binding | 0.12476339 | 0.330256032 | 0.309011492 | BMP3 | 1 |
| MF | GO:0043425 | bHLH transcription factor binding | 0.12476339 | 0.330256032 | 0.309011492 | ISL1 | 1 |
| MF | GO:0070566 | adenylyltransferase activity | 0.12476339 | 0.330256032 | 0.309011492 | FHIT | 1 |
| MF | GO:0140828 | metal cation:monoatomic cation antiporter activity | 0.128520995 | 0.334910746 | 0.31336678 | SLC9C2 | 1 |
| MF | GO:0005251 | delayed rectifier potassium channel activity | 0.132262671 | 0.334910746 | 0.31336678 | KCNQ3 | 1 |
| MF | GO:0140658 | ATP-dependent chromatin remodeler activity | 0.132262671 | 0.334910746 | 0.31336678 | SMARCAL1 | 1 |
| MF | GO:0004993 | G protein-coupled serotonin receptor activity | 0.135988485 | 0.334910746 | 0.31336678 | HTR1E | 1 |
| MF | GO:0099589 | serotonin receptor activity | 0.135988485 | 0.334910746 | 0.31336678 | HTR1E | 1 |
| MF | GO:0022843 | voltage-gated cation channel activity | 0.136911929 | 0.334910746 | 0.31336678 | CNR1/KCNQ3 | 2 |
| MF | GO:0031625 | ubiquitin protein ligase binding | 0.136941283 | 0.334910746 | 0.31336678 | PRDX6/FHIT/SLF2 | 3 |
| MF | GO:0045505 | dynein intermediate chain binding | 0.139698503 | 0.337980249 | 0.31623883 | DNAH8 | 1 |
| MF | GO:0005484 | SNAP receptor activity | 0.147071415 | 0.34860362 | 0.326178825 | STX6 | 1 |
| MF | GO:0015079 | potassium ion transmembrane transporter activity | 0.147188195 | 0.34860362 | 0.326178825 | SLC9C2/KCNQ3 | 2 |
| MF | GO:0016620 | oxidoreductase activity, acting on the aldehyde or oxo group of donors, NAD or NADP as acceptor | 0.154381934 | 0.358062142 | 0.335028905 | PDHA2 | 1 |
| MF | GO:0030331 | nuclear estrogen receptor binding | 0.154381934 | 0.358062142 | 0.335028905 | ISL1 | 1 |
| MF | GO:0044389 | ubiquitin-like protein ligase binding | 0.155955955 | 0.358062142 | 0.335028905 | PRDX6/FHIT/SLF2 | 3 |
| MF | GO:0048018 | receptor ligand activity | 0.159502484 | 0.360967136 | 0.337747028 | FASLG/TNFSF18/BMP3/ALKAL1 | 4 |
| MF | GO:0015298 | solute:cation antiporter activity | 0.165231862 | 0.360967136 | 0.337747028 | SLC9C2 | 1 |
| MF | GO:0035254 | glutamate receptor binding | 0.165231862 | 0.360967136 | 0.337747028 | DNM3 | 1 |
| MF | GO:0140303 | intramembrane lipid transporter activity | 0.165231862 | 0.360967136 | 0.337747028 | ATP8A2 | 1 |
| MF | GO:0030546 | signaling receptor activator activity | 0.165242733 | 0.360967136 | 0.337747028 | FASLG/TNFSF18/BMP3/ALKAL1 | 4 |
| MF | GO:0003924 | GTPase activity | 0.169420188 | 0.365919339 | 0.342380668 | DNM3/GNA12/RGS6 | 3 |
| MF | GO:0005245 | voltage-gated calcium channel activity | 0.172388667 | 0.365919339 | 0.342380668 | CNR1 | 1 |
| MF | GO:0016790 | thiolester hydrolase activity | 0.172388667 | 0.365919339 | 0.342380668 | ABHD10 | 1 |
| MF | GO:0030544 | Hsp70 protein binding | 0.179484883 | 0.377421484 | 0.353142909 | DNAJB9 | 1 |
| MF | GO:0016763 | pentosyltransferase activity | 0.186521019 | 0.385120539 | 0.360346703 | XXYLT1 | 1 |
| MF | GO:0008237 | metallopeptidase activity | 0.186569506 | 0.385120539 | 0.360346703 | AMZ1/ADAMTS18 | 2 |
| MF | GO:0070888 | E-box binding | 0.190016713 | 0.388670548 | 0.363668349 | MAX | 1 |
| MF | GO:0008227 | G protein-coupled amine receptor activity | 0.196963666 | 0.399250674 | 0.373567882 | HTR1E | 1 |
| MF | GO:0042578 | phosphoric ester hydrolase activity | 0.206099319 | 0.409733901 | 0.383376749 | PDE1C/EYA1/PLCG2 | 3 |
| MF | GO:0043539 | protein serine/threonine kinase activator activity | 0.207273936 | 0.409733901 | 0.383376749 | RICTOR | 1 |
| MF | GO:0004601 | peroxidase activity | 0.210681562 | 0.409733901 | 0.383376749 | PRDX6 | 1 |
| MF | GO:0005516 | calmodulin binding | 0.211491871 | 0.409733901 | 0.383376749 | PDE1C/KCNQ3 | 2 |
| MF | GO:0005244 | voltage-gated ion channel activity | 0.213061628 | 0.409733901 | 0.383376749 | CNR1/KCNQ3 | 2 |
| MF | GO:0022832 | voltage-gated channel activity | 0.213061628 | 0.409733901 | 0.383376749 | CNR1/KCNQ3 | 2 |
| MF | GO:0015631 | tubulin binding | 0.217225564 | 0.411151548 | 0.384703202 | DNM3/UNC5C/DCLK2 | 3 |
| MF | GO:0016684 | oxidoreductase activity, acting on peroxide as acceptor | 0.217453485 | 0.411151548 | 0.384703202 | PRDX6 | 1 |
| MF | GO:1990841 | promoter-specific chromatin binding | 0.237426949 | 0.44038165 | 0.412053006 | ISL1 | 1 |
| MF | GO:0019887 | protein kinase regulator activity | 0.239898492 | 0.44038165 | 0.412053006 | RICTOR/ALKAL1 | 2 |
| MF | GO:0005548 | phospholipid transporter activity | 0.240706565 | 0.44038165 | 0.412053006 | ATP8A2 | 1 |
| MF | GO:0051015 | actin filament binding | 0.241483958 | 0.44038165 | 0.412053006 | TULP1/ARPC1B | 2 |
| MF | GO:0030145 | manganese ion binding | 0.243972254 | 0.44038165 | 0.412053006 | XXYLT1 | 1 |
| MF | GO:0016747 | acyltransferase activity, transferring groups other than amino-acyl groups | 0.244656472 | 0.44038165 | 0.412053006 | PRDX6/GPAM | 2 |
| MF | GO:0097110 | scaffold protein binding | 0.247224075 | 0.441223093 | 0.412840321 | PLCG2 | 1 |
| MF | GO:0003777 | microtubule motor activity | 0.250462085 | 0.441223093 | 0.412840321 | DNAH8 | 1 |
| MF | GO:0000287 | magnesium ion binding | 0.251006915 | 0.441223093 | 0.412840321 | XXYLT1/ATP8A2 | 2 |
| MF | GO:0051536 | iron-sulfur cluster binding | 0.256896906 | 0.444629261 | 0.416027379 | FXN | 1 |
| MF | GO:0051540 | metal cluster binding | 0.256896906 | 0.444629261 | 0.416027379 | FXN | 1 |
| MF | GO:0016811 | hydrolase activity, acting on carbon-nitrogen (but not peptide) bonds, in linear amides | 0.260093832 | 0.446726047 | 0.417989284 | FHIT | 1 |
| MF | GO:0019905 | syntaxin binding | 0.263277177 | 0.448767916 | 0.419899804 | STX6 | 1 |
| MF | GO:0000049 | tRNA binding | 0.269603355 | 0.4526922 | 0.423571649 | PTCD1 | 1 |
| MF | GO:0043022 | ribosome binding | 0.269603355 | 0.4526922 | 0.423571649 | RICTOR | 1 |
| MF | GO:0017018 | myosin phosphatase activity | 0.278992186 | 0.464986976 | 0.435075533 | EYA1 | 1 |
| MF | GO:0019207 | kinase regulator activity | 0.284391646 | 0.468862518 | 0.438701771 | RICTOR/ALKAL1 | 2 |
| MF | GO:0046873 | metal ion transmembrane transporter activity | 0.287308058 | 0.468862518 | 0.438701771 | SLC9C2/CNR1/KCNQ3 | 3 |
| MF | GO:0016746 | acyltransferase activity | 0.287569011 | 0.468862518 | 0.438701771 | PRDX6/GPAM | 2 |
| MF | GO:0140104 | molecular carrier activity | 0.30043856 | 0.486321409 | 0.455037576 | FXN | 1 |
| MF | GO:0005546 | phosphatidylinositol-4,5-bisphosphate binding | 0.306449556 | 0.489015248 | 0.457558127 | TULP1 | 1 |
| MF | GO:0016209 | antioxidant activity | 0.306449556 | 0.489015248 | 0.457558127 | PRDX6 | 1 |
| MF | GO:0008017 | microtubule binding | 0.323965727 | 0.508542813 | 0.475829533 | DNM3/DCLK2 | 2 |
| MF | GO:0001228 | DNA-binding transcription activator activity, RNA polymerase II-specific | 0.324903279 | 0.508542813 | 0.475829533 | ISL1/ETV6/IRX6 | 3 |
| MF | GO:0005126 | cytokine receptor binding | 0.327112881 | 0.508542813 | 0.475829533 | FASLG/TNFSF18 | 2 |
| MF | GO:0001216 | DNA-binding transcription activator activity | 0.329614014 | 0.508542813 | 0.475829533 | ISL1/ETV6/IRX6 | 3 |
| MF | GO:0015297 | antiporter activity | 0.329987781 | 0.508542813 | 0.475829533 | SLC9C2 | 1 |
| MF | GO:0004553 | hydrolase activity, hydrolyzing O-glycosyl compounds | 0.332874032 | 0.50950107 | 0.476726147 | ABHD10 | 1 |
| MF | GO:0004725 | protein tyrosine phosphatase activity | 0.344296787 | 0.515974166 | 0.482782845 | EYA1 | 1 |
| MF | GO:0004867 | serine-type endopeptidase inhibitor activity | 0.344296787 | 0.515974166 | 0.482782845 | ITIH2 | 1 |
| MF | GO:0005249 | voltage-gated potassium channel activity | 0.347122169 | 0.515974166 | 0.482782845 | KCNQ3 | 1 |
| MF | GO:0001664 | G protein-coupled receptor binding | 0.352145238 | 0.515974166 | 0.482782845 | DNM3/GNA12 | 2 |
| MF | GO:0004722 | protein serine/threonine phosphatase activity | 0.352736921 | 0.515974166 | 0.482782845 | EYA1 | 1 |
| MF | GO:0005496 | steroid binding | 0.352736921 | 0.515974166 | 0.482782845 | IRX5 | 1 |
| MF | GO:0060090 | molecular adaptor activity | 0.353155651 | 0.515974166 | 0.482782845 | STX6/RICTOR/TJP2 | 3 |
| MF | GO:0051087 | chaperone binding | 0.363823781 | 0.528131295 | 0.494157937 | DNAJB9 | 1 |
| MF | GO:0005200 | structural constituent of cytoskeleton | 0.369296686 | 0.532639451 | 0.498376095 | ARPC1B | 1 |
| MF | GO:1902936 | phosphatidylinositol bisphosphate binding | 0.3747231 | 0.534520423 | 0.500136069 | TULP1 | 1 |
| MF | GO:0000149 | SNARE binding | 0.377418995 | 0.534520423 | 0.500136069 | STX6 | 1 |
| MF | GO:0003774 | cytoskeletal motor activity | 0.380103412 | 0.534520423 | 0.500136069 | DNAH8 | 1 |
| MF | GO:0030594 | neurotransmitter receptor activity | 0.380103412 | 0.534520423 | 0.500136069 | HTR1E | 1 |
| MF | GO:0004222 | metalloendopeptidase activity | 0.382776401 | 0.534935964 | 0.500524879 | ADAMTS18 | 1 |
| MF | GO:0005262 | calcium channel activity | 0.401171331 | 0.557182404 | 0.521340261 | CNR1 | 1 |
| MF | GO:0004197 | cysteine-type endopeptidase activity | 0.403754603 | 0.557329974 | 0.521478338 | TINAG | 1 |
| MF | GO:0008094 | ATP-dependent activity, acting on DNA | 0.406326872 | 0.557460648 | 0.521600606 | SMARCAL1 | 1 |
| MF | GO:0005267 | potassium channel activity | 0.408888184 | 0.557574796 | 0.521707411 | KCNQ3 | 1 |
| MF | GO:0003697 | single-stranded DNA binding | 0.41397812 | 0.557754951 | 0.521875978 | SMARCAL1 | 1 |
| MF | GO:0031072 | heat shock protein binding | 0.41397812 | 0.557754951 | 0.521875978 | DNAJB9 | 1 |
| MF | GO:0045296 | cadherin binding | 0.419172588 | 0.561391859 | 0.525278933 | PRDX6/TJP2 | 2 |
| MF | GO:0016810 | hydrolase activity, acting on carbon-nitrogen (but not peptide) bonds | 0.42402852 | 0.564535011 | 0.528219894 | FHIT | 1 |
| MF | GO:0022836 | gated channel activity | 0.431003584 | 0.570370502 | 0.533680002 | CNR1/KCNQ3 | 2 |
| MF | GO:0016798 | hydrolase activity, acting on glycosyl bonds | 0.433908698 | 0.570370502 | 0.533680002 | ABHD10 | 1 |
| MF | GO:0005261 | cation channel activity | 0.438334794 | 0.570370502 | 0.533680002 | CNR1/KCNQ3 | 2 |
| MF | GO:0030674 | protein-macromolecule adaptor activity | 0.43979506 | 0.570370502 | 0.533680002 | STX6/TJP2 | 2 |
| MF | GO:0015085 | calcium ion transmembrane transporter activity | 0.441208838 | 0.570370502 | 0.533680002 | CNR1 | 1 |
| MF | GO:0015078 | proton transmembrane transporter activity | 0.443621502 | 0.570370502 | 0.533680002 | SLC9C2 | 1 |
| MF | GO:0016922 | nuclear receptor binding | 0.45079795 | 0.576304198 | 0.539231998 | ISL1 | 1 |
| MF | GO:0008194 | UDP-glycosyltransferase activity | 0.45788299 | 0.582054649 | 0.544612537 | XXYLT1 | 1 |
| MF | GO:0019842 | vitamin binding | 0.474065703 | 0.595892644 | 0.557560368 | IRX5 | 1 |
| MF | GO:0019903 | protein phosphatase binding | 0.474065703 | 0.595892644 | 0.557560368 | GNA12 | 1 |
| MF | GO:0005506 | iron ion binding | 0.483097843 | 0.602177804 | 0.56344122 | FXN | 1 |
| MF | GO:0005525 | GTP binding | 0.485411646 | 0.602177804 | 0.56344122 | DNM3/GNA12 | 2 |
| MF | GO:0004386 | helicase activity | 0.487556308 | 0.602177804 | 0.56344122 | SMARCAL1 | 1 |
| MF | GO:0015081 | sodium ion transmembrane transporter activity | 0.489771281 | 0.602177804 | 0.56344122 | SLC9C2 | 1 |
| MF | GO:0043021 | ribonucleoprotein complex binding | 0.496359637 | 0.606961513 | 0.567917205 | RICTOR | 1 |
| MF | GO:0005319 | lipid transporter activity | 0.500705138 | 0.608303208 | 0.569172592 | ATP8A2 | 1 |
| MF | GO:0008083 | growth factor activity | 0.502863985 | 0.608303208 | 0.569172592 | BMP3 | 1 |
| MF | GO:0019001 | guanyl nucleotide binding | 0.515441282 | 0.616884513 | 0.577201884 | DNM3/GNA12 | 2 |
| MF | GO:0032561 | guanyl ribonucleotide binding | 0.515441282 | 0.616884513 | 0.577201884 | DNM3/GNA12 | 2 |
| MF | GO:0140030 | modification-dependent protein binding | 0.523951891 | 0.623752251 | 0.583627837 | PLCG2 | 1 |
| MF | GO:0003735 | structural constituent of ribosome | 0.532137541 | 0.628810525 | 0.588360725 | RPL37A | 1 |
| MF | GO:0004721 | phosphoprotein phosphatase activity | 0.534162143 | 0.628810525 | 0.588360725 | EYA1 | 1 |
| MF | GO:0004866 | endopeptidase inhibitor activity | 0.540184191 | 0.628810525 | 0.588360725 | ITIH2 | 1 |
| MF | GO:1901981 | phosphatidylinositol phosphate binding | 0.540184191 | 0.628810525 | 0.588360725 | TULP1 | 1 |
| MF | GO:0008022 | protein C-terminus binding | 0.542174408 | 0.628810525 | 0.588360725 | FOXN3 | 1 |
| MF | GO:0004175 | endopeptidase activity | 0.548162962 | 0.631435614 | 0.590816949 | TINAG/ADAMTS18 | 2 |
| MF | GO:0008234 | cysteine-type peptidase activity | 0.550050579 | 0.631435614 | 0.590816949 | TINAG | 1 |
| MF | GO:0030414 | peptidase inhibitor activity | 0.553938345 | 0.632670698 | 0.591972583 | ITIH2 | 1 |
| MF | GO:0003779 | actin binding | 0.5645704 | 0.637119944 | 0.59613562 | TULP1/ARPC1B | 2 |
| MF | GO:0061135 | endopeptidase regulator activity | 0.567286134 | 0.637119944 | 0.59613562 | ITIH2 | 1 |
| MF | GO:0016758 | hexosyltransferase activity | 0.569160484 | 0.637119944 | 0.59613562 | ALG6 | 1 |
| MF | GO:0019902 | phosphatase binding | 0.569160484 | 0.637119944 | 0.59613562 | GNA12 | 1 |
| MF | GO:0005216 | ion channel activity | 0.573230329 | 0.638499129 | 0.597426086 | CNR1/KCNQ3 | 2 |
| MF | GO:0005543 | phospholipid binding | 0.606635616 | 0.671631491 | 0.628427126 | PLA2G2F/TULP1 | 2 |
| MF | GO:0140297 | DNA-binding transcription factor binding | 0.608945885 | 0.671631491 | 0.628427126 | ISL1/MAX | 2 |
| MF | GO:0005085 | guanyl-nucleotide exchange factor activity | 0.620163044 | 0.676733832 | 0.633201246 | VAV2 | 1 |
| MF | GO:0030695 | GTPase regulator activity | 0.622595125 | 0.676733832 | 0.633201246 | VAV2/RGS6 | 2 |
| MF | GO:0060589 | nucleoside-triphosphatase regulator activity | 0.622595125 | 0.676733832 | 0.633201246 | VAV2/RGS6 | 2 |
| MF | GO:0015267 | channel activity | 0.62928339 | 0.678376252 | 0.634738013 | CNR1/KCNQ3 | 2 |
| MF | GO:0022803 | passive transmembrane transporter activity | 0.630389278 | 0.678376252 | 0.634738013 | CNR1/KCNQ3 | 2 |
| MF | GO:0061134 | peptidase regulator activity | 0.633151168 | 0.678376252 | 0.634738013 | ITIH2 | 1 |
| MF | GO:0005539 | glycosaminoglycan binding | 0.641045037 | 0.683578831 | 0.639605924 | ITIH2 | 1 |
| MF | GO:0140097 | catalytic activity, acting on DNA | 0.647239178 | 0.686928373 | 0.642739998 | SMARCAL1 | 1 |
| MF | GO:0015291 | secondary active transmembrane transporter activity | 0.654833624 | 0.69172566 | 0.647228687 | SLC9C2 | 1 |
| MF | GO:0005096 | GTPase activator activity | 0.695802061 | 0.722224886 | 0.675765975 | RGS6 | 1 |
| MF | GO:0030246 | carbohydrate binding | 0.695802061 | 0.722224886 | 0.675765975 | ATRN | 1 |
| MF | GO:0016791 | phosphatase activity | 0.697125608 | 0.722224886 | 0.675765975 | EYA1 | 1 |
| MF | GO:0035091 | phosphatidylinositol binding | 0.698443468 | 0.722224886 | 0.675765975 | TULP1 | 1 |
| MF | GO:0022853 | active ion transmembrane transporter activity | 0.699755667 | 0.722224886 | 0.675765975 | SLC9C2 | 1 |
| MF | GO:0046982 | protein heterodimerization activity | 0.77199385 | 0.793144367 | 0.742123384 | MAX | 1 |
| MF | GO:0016887 | ATP hydrolysis activity | 0.777903779 | 0.794933925 | 0.743797824 | ATP8A2 | 1 |
| MF | GO:0061629 | RNA polymerase II-specific DNA-binding transcription factor binding | 0.780801766 | 0.794933925 | 0.743797824 | ISL1 | 1 |
| MF | GO:0106310 | protein serine kinase activity | 0.792934263 | 0.803649591 | 0.751952834 | DCLK2 | 1 |
| MF | GO:0004857 | enzyme inhibitor activity | 0.820046091 | 0.827400765 | 0.774176154 | ITIH2 | 1 |
| MF | GO:0022804 | active transmembrane transporter activity | 0.836620577 | 0.84035549 | 0.786297534 | SLC9C2 | 1 |
| MF | GO:0004674 | protein serine/threonine kinase activity | 0.845696297 | 0.845696297 | 0.791294781 | DCLK2 | 1 |

**
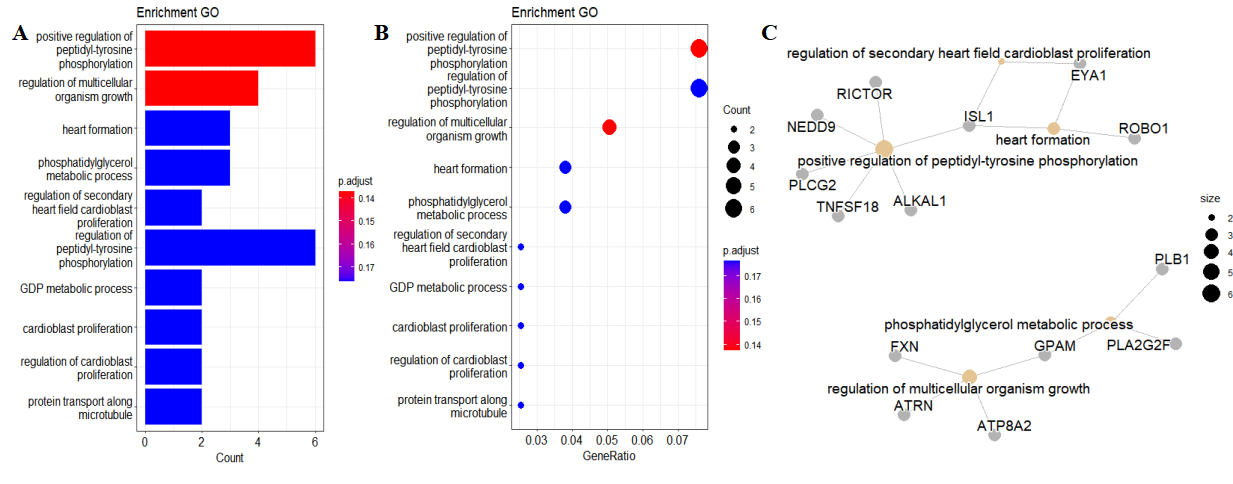
Supplementary Figure.1** Functional enrichment analysis based on cross-trait meta-analysis results. **(A)** Barplot of the top 10 changes of GO analysis. **(B)** Dotplot of the top 10 changes of GO analysis. **(C)** Netplot of the top 5 changes of GO analysis.

**
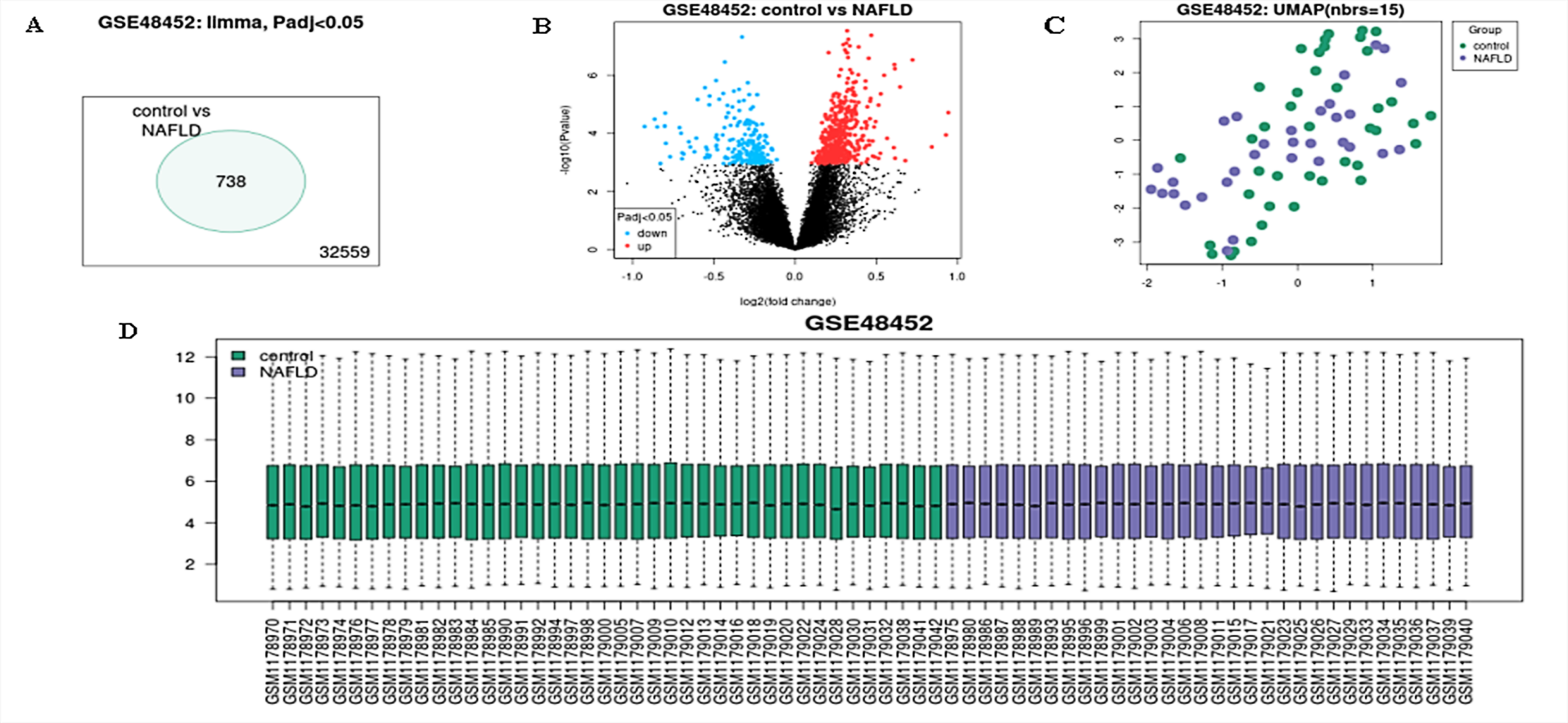
**

**Supplementary Figure.2** Identification of differentially expressed genes in the discovery set GSE48452. **(A)** Venn diagram of important genes in NAFLD and healthy people. **(B)** The volcano plot show DEGs in 73 samples from GSE48452. **(C)** UMAP diagram of the relationship between samples of NAFLD patients and healthy individuals. **(D)** Boxplot of gene expression profiles of 32 NAFLD patients and 41 healthy people.

**
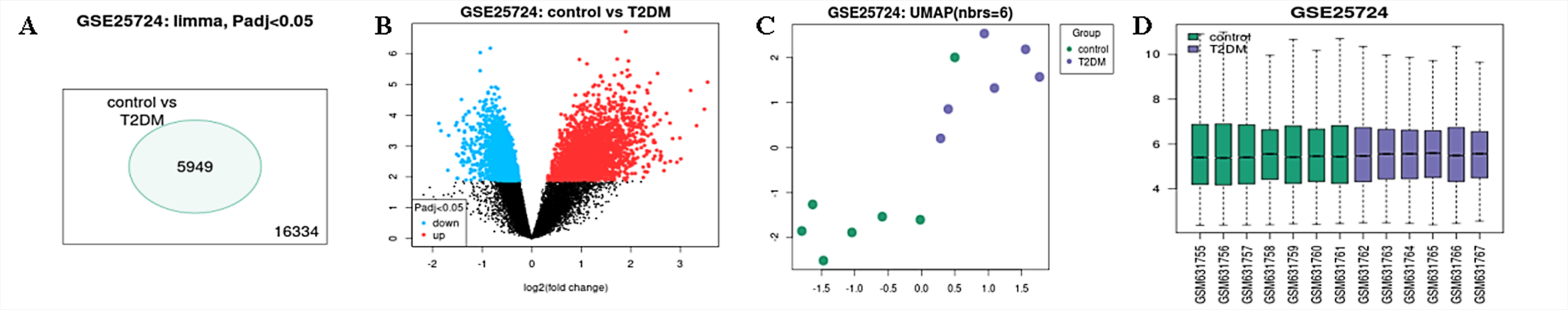
Supplementary Figure.3** Identification of differentially expressed genes in the discovery set GSE25724. **(A)** Venn diagram of important genes in T2D and healthy people. **(B)** The volcano plot show DEGs in 13 samples from GSE25724. **(C)** UMAP diagram of the relationship between samples of T2D patients and healthy individuals. **(D)** Boxplot of gene expression profiles of 6 T2D patients and 7 healthy people.

**
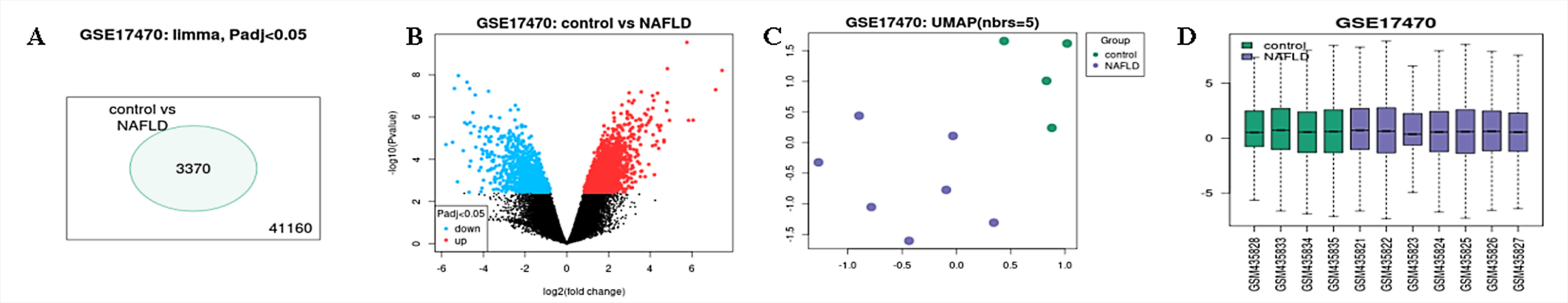
Supplementary Figure.4** Identification of differentially expressed genes in the validation set GSE17470.**(A)** Venn diagram of important genes in NAFLD and healthy people. **(B)** The volcano plot show DEGs in 11 samples from GSE17470. **(C)** UMAP diagram of the relationship between samples of NAFLD patients and healthy individuals. **(D)** Boxplot of gene expression profiles of 7 NAFLD patients and 4 healthy people.


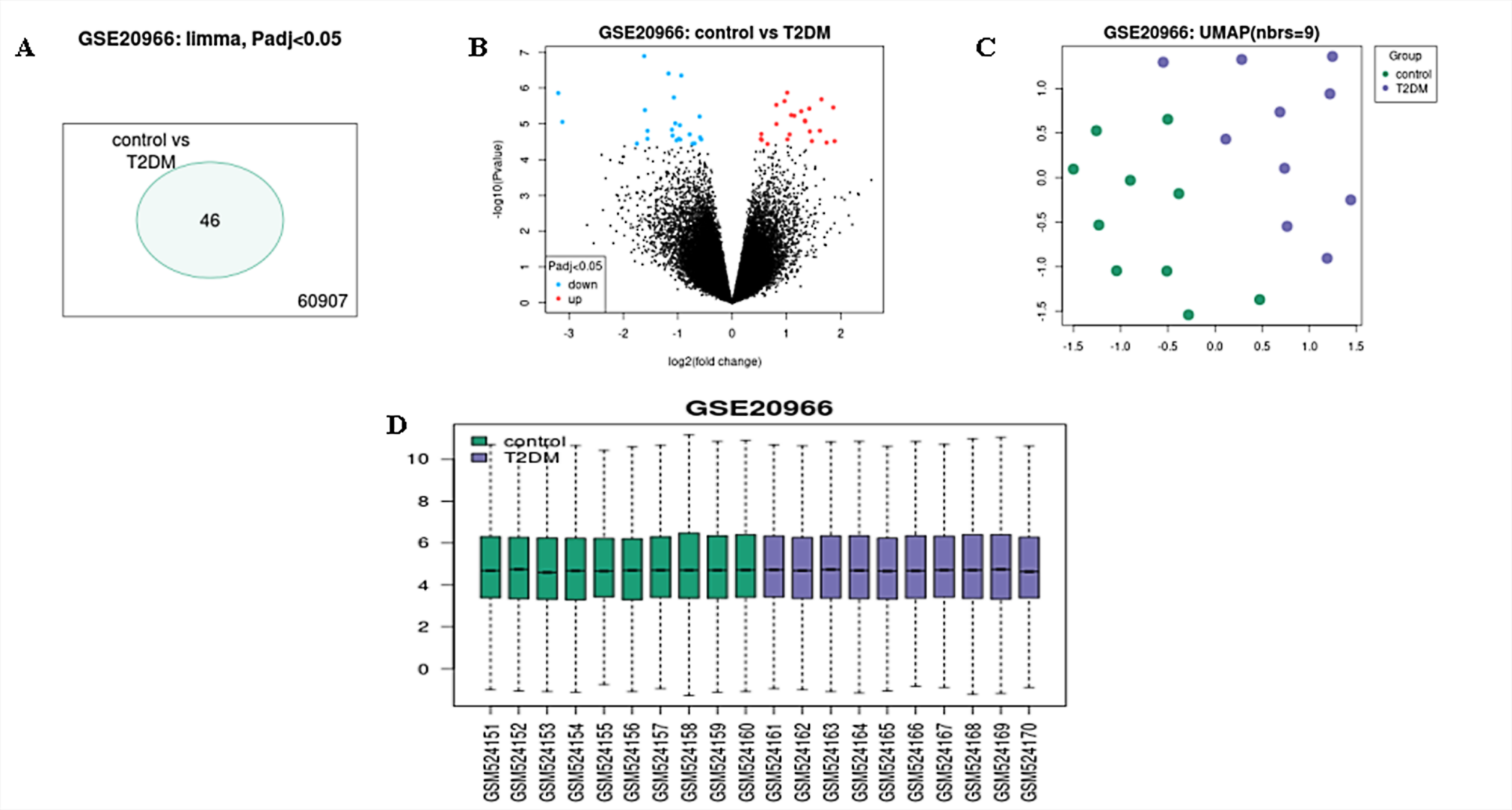


**Supplementary Figure.5** Identification of differentially expressed genes in the validation set GSE20966.**(A)** Venn diagram of important genes in T2D and healthy people. **(B)** The volcano plot show DEGs in 20 samples from GSE20966. **(C)** UMAP diagram of the relationship between samples of T2D patients and healthy individuals. **(D)** Boxplot of gene expression profiles of 10 T2D patients and 10 healthy people.

**
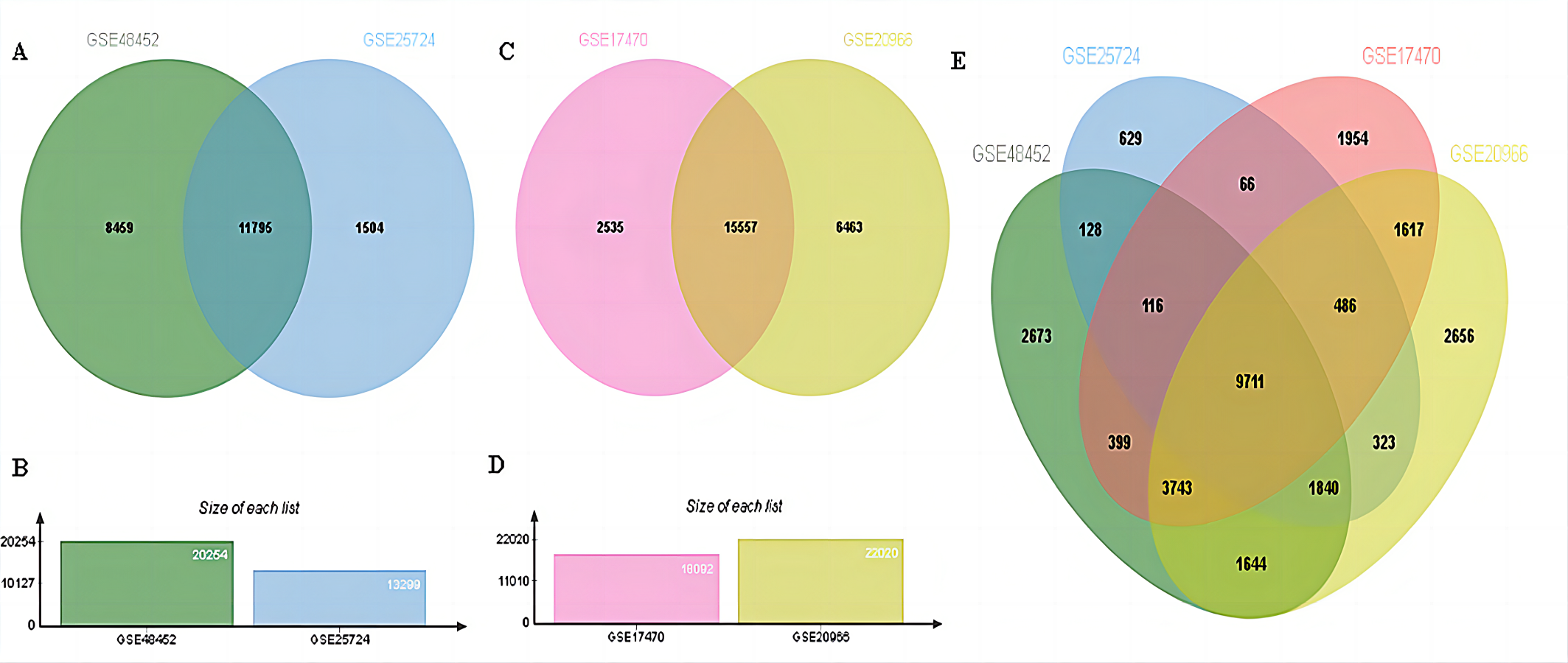
Supplementary Figure.6** Venn diagram of differential gene expression analysis process. **(A)** Venn diagram of shared genes of NAFLD discovery set GSE48452 and T2D discovery set GSE25724. **(B)** Size of NAFLD discovery set GSE48452 and T2D discovery set GSE25724. **(C)** Venn diagram of shared genes for NAFLD validation set GSE17470 and T2D validation set GSE20966. **(D)** Size of NAFLD validation set GSE17470 and T2D validation set GSE20966. **(E)** Venn diagram of shared genes for all discovery and validation sets.

**Supplementary Table 4** Shared genes for all discovery and validation sets in differential gene expression analysis (5545 in total)

| Group | Gene |
| --- | --- |
| Shared genes across all discovery and validation sets (adj.P<0.05) | SYBU, FUT6, SERBP1, HAX1, MFN1, TCEA1, SSR1, ERO1B, CDC40, PRDX4, APEX1, PSMG1, IAPP, UQCRC2, SRP72, HADHA, UPF3A, PDIA6, PLCB3, PRKCZ, IARS, BCL2L2, PPP1R7, ACSL1, APBA2, USP1, TM9SF2, HSPE1, MICB, C7orf43, SRSF8, NELFCD, ARCN1, EDEM3, DAP, FNDC3A, TOP3A, DNAJB9, BET1, TGM2, SECTM1, IPO5, POLQ, PIKFYVE, XPOT, WDR12, LMNA, WDR61, DHX29, SEC62, MAPRE1, GAL3ST4, PPM1A, TMX4, KDELR2, PSMD11, COL16A1, RPL15, UNC50, SRSF6, NMT1, TMC6, MKRN2, L1CAM, LSM12, CLK1, AHCY, DDOST, SRSF10, KSR1, MBD1, CACNB1, ARMCX1, TTC38, ZFAND1, PEX2, UAP1, NSMAF, BRD4, TMX1, TMEM251, PRDM10, DCTD, CAMKK2, MRPL3, LTN1, SH3GLB1, DCTN6, ZNHIT6, PCSK1, GTF3C3, ATP5B, TMED10, G3BP2, TCF7L2, SLC25A5, RNF14, 6-Mar, SEC63, ILF2, ERC1, OXSM, MUC5B, GORASP2, PITPNA, SEL1L, OAZ1, FTSJ1, SUCO, ZNF473, AKAP13, EMC2, UBE2N, HADH, TMEM70, PIK3R4, GRK6, SRPRB, RBP4, ANAPC5, EXOC1, SLC33A1, PRPSAP2, CTBP2, MTO1, USP16, PGRMC2, NR2F2, KATNB1, SDC2, UBE2D2, MAP3K11, CEND1, HOXA3, CCL5, CX3CL1, CYP3A4, SYN3, EDEM1, DLD, SLC28A3, ZNF771, NECAP1, TRIM2, ISOC1, KLHL28, NT5M, GTSE1, HSP90AB1, ME2, EEF1A1, SEPHS1, COPS2, RNF13, MON2, PJA2, IDH3A, SEC11A, RAD54L, RND2, DERL1, SNX24, PIGT, MYH14, ARPP19, BUB3, PI4KB, CD38, RAPGEF3, HTR2A, TPD52, UBR7, CPNE3, POU3F1, HAT1, C5orf22, CRKL, WDR47, VPS53, AKAP11, MLH1, TMEM50A, BEX4, RAB1A, ARFGEF2, ZNHIT3, TNFRSF14, GOLGA5, TFR2, SH3PXD2A, UBE3A, FANCC, PFN2, NUCB2, TRADD, PAICS, SLC30A5, PTS, PTGES3, COPZ1, CAPN7, MTMR3, SUCLA2, MAST4, SCGN, ATRX, RECQL5, HMGN2, ISCA1, ZC3H15, KIAA0101, CALCOCO2, PRKAR1A, SLC35B1, ARVCF, CDC16, PTPN3, KIFC1, EID1, KCNJ9, SEMA7A, MFAP3, MCM3AP, PSMD6, CYP17A1, UNC119B, SRSF7, TOMM20, SERP1, LMBRD1, TMED2, KIAA1033, RPL6, CLIP2, PGM3, GPATCH2, CASZ1, GNS, SERPINI1, RCHY1, RIN3, RRBP1, CUL3, HPRT1, VDAC3, TIMELESS, SF3B1, SEC23B, SNX2, ENPP2, NUDT7, TXNDC15, PPP3CA, TRIM62, GRIN1, SHOC2, 15-Sep, ATP6V1A, CNBP, KLHDC2, ESD, SIM2, MANF, SSTR3, PCM1, NUP37, UFM1, SLC45A2, LRRFIP1, GALC, CAT, COL9A1, RBBP4, FAM208B, RPA1, NME7, NAP1L2, DHX15, ACP1, RWDD2A, CREB3L1, SH2B2, PPP1CC, TEX261, GNAQ, APC, OR2C1, PRPS1, MRC2, SDHD, XBP1, PTPRF, DTX4, MAP2K4, SMC3, PCNP, ANP32E, SUCLG2, ZZZ3, EIF4E, TTC19, MGAT2, BCL2L13, CMKLR1, PRELID3B, FHL1, PEX11B, INTS12, TTC37, H2AFX, PPP6C, UCHL1, PSMA1, MTMR2, PPIP5K2, HSPA12A, PDHX, CCDC15, DMXL1, PRKRA, PSMD8, VDAC1, GSPT1, ZNF277, PRKAG1, KRT33B, PAWR, TOM1L1, RFC3, LYPLA1, WFDC2, SLC25A38, IRF7, ARGLU1, CHAF1A, ITGB4, CHUK, DHRS7B, TRMT5, RASA1, SLC16A1, TIGAR, PARVA, SOX4, CRY1, FAM179B, KHSRP, TMX2, ADSS, SLC25A11, CRYBB3, ENPP4, RPL22, MYO7A, TUBB, COPB1, MIS12, ZMIZ2, CAND1, TMEM14B, CSE1L, ZDHHC4, AP2M1, NDFIP1, NENF, DDX46, SERPINB13, COX7A2, BCLAF1, GATA1, RAB11A, RPS6KC1, PTAFR, SLC16A8, NOL7, FGFR1, EIF3J, ANKRD49, SERINC3, UBL3, CD164, FBL, HNRNPDL, SELT, RNF113A, ZBTB7A, SRPRA, MPP6, DCK, SSR3, CAPN9, SPC24, PFKM, NCBP2, CAPRIN1, PSMB5, METAP2, RBM8A, ZNF335, KPNA2, TAF2, CCT4, TNFAIP2, SEC14L4, PRDM2, TFB2M, AIPL1, ALMS1, FLJ11710, LGR4, POU2F2, GJC2, PTCD3, EAPP, AGA, OSTM1, CMAHP, AZIN1, MCFD2, PSMD5, EMC7, TMEM104, CFAP20, ASPH, PPP2CB, KPNA1, FBXW2, CYP2D6, EXD3, LST1, CST4, WNK1, PQBP1, ERH, TMEM267, THRA, RBM7, PLEKHA4, CRH, PRKAB1, HMBOX1, RFC5, PCBP4, RAB14, SUN1, EHD1, TNNI2, CUL5, ZNF148, RAB2A, PDGFB, SLBP, GRIN2B, CTR9, KCNA10, LAMTOR5, TPRKB, ARPC1A, MPC2, PSMB7, RAP1GDS1, MRPL17, WISP2, CLTCL1, NPTN, SLC30A1, RHOA, SERINC1, DHX35, SRP54, HDDC2, SOX2, ATF1, DCAF7, PSMC3, STARD13, RBM23, RREB1, FUBP1, NOL11, ALG5, NOD1, CST8, EIF2B1, DYNC1LI1, LOC101927181, PEX3, IFNB1, CCDC40, CD248, COL2A1, ENOPH1, VLDLR, KCTD3, TRAPPC3, GLI1, ATXN7, CCL13, MED6, AP5M1, WDYHV1, TMEM41B, SNHG4///MATR3, XRCC5, EIF5, SEPT11, BTK, GPR63, DNASE2, MAGEB2, RDX, PCDH1, SDHB, TSN, TSNAXIP1, TEX2, CMAS, CHMP5, LZTFL1, GFER, ERGIC2, ZAP70, SPRED2, SAR1A, REXO2, AGPAT5, RIOK2, TTLL5, CENPC, BEX1, RSL1D1, KCTD17, PSMD7, PNMA2, UBE2G1, SEC23IP, MAPRE3, POLR2B, FZR1, ACAP2, ZFP36L1, CCNF, PRKACA, HTR4, SNX4, MUC4, AGMAT, RRAGA, COG5, GMFB, ALDH9A1, AK2, FGFR2, CNIH1, ARFGAP3, EPS8L1, TMEM208, YTHDF3, PML, BST2, PAK5, MAGEA10, PLEKHA6, IMP4, IMPA1, SEC24A, ARPIN, RUNX3, MPC1, INCENP, CBX8, MEAF6, CCSER2, TIMM17A, CREG1, SMARCA4, BMP10, PVRIG, NRGN, EED, SLC22A2, LARP1, MLX, MTX2, NFATC4, HSPA2, JUND, TRMT11, SLC26A2, TMEM135, ACAD8, ABCE1, PCBP2, TGFBR2, OAT, NR4A3, VAMP7, RRAGD, LPXN, GIN1, MBD4, PANK3, MMP24, RAB28, CYC1, ANGPT1, COPS5, GMIP, CDK4, RPL30, CD99, POLE3, CYP4F12, NR3C1, SLC30A9, TDP2, PREPL, SDF4, APP, BRD8, PSD3, TRA2B, RER1, MAP1LC3B, FGF5, NEK7, MLIP, ACADM, MLLT10, PEBP1, ZDHHC17, RFX5, BHLHE40, H6PD, ENTPD2, CCDC9, SREK1, GNAS, ZC3H7B, LPCAT4, DICER1, EPX, RPL35A, CTF1, SH3TC2, PLP2, GAPDH, CD7, FBXW11, RPS7, PNMA3, TLK1, JOSD1, TMEM165, SLC24A2, POP7, ARAP3, ZIC1, SPN, EPRS, SKIV2L, RBM25, POLR2K, EBLN2, USP46, ZNF391, AMZ2, NPY6R, FEZ2, PPP1R12A, SERPINF2, ACAT1, DHRS7, SLC35E1, CRYGC, RTN1, GAS7, ELOVL5, PPP3CC, NKX2-1, SCG5, ESR1, CLCN3, LIPT1, LSM14A, PRKCH, TNS4, GLO1, DDX1, POLR1B, HSPA14, HNRNPH3, NRBF2, NDUFB8, CLN5, ADIPOR2, TCF25, SNX13, CD46, HMGCR, TNFSF14, CHRNA2, CORO7, ENTPD3, LMBR1L, SPON1, PPT1, ATP10A, TMEM63A, SLC8A1, ARMT1, NOC3L, MAPRE2, CDC73, MMP11, IRF4, TMED3, MRS2, KCNH6, BDKRB2, STAM2, ABAT, HSF2BP, CIITA, RWDD1, MOG, GJD2, HRASLS2, PPP2CA, PRIM1, CDC5L, CEP63, EIF4H, UFC1, DNAAF2, ADAM28, DCAF15, THAP12, JAG1, IPO7, PDGFA, SCARB1, AGK, PAFAH1B1, RNF167, STMN2, MRPL19, ATP6V1B2, ALG9, ADARB1, ORMDL2, OSBPL8, GRAP, ATXN2L, MAN1A1, MEX3D, PAX9, HMG20A, CYTH3, LPP, LAPTM4B, IGF2BP2, SLC4A1, PTGER1, PRDX3, SNX3, CARD14, RGS12, GSTA4, FLJ42627, EPHB1, ZBTB1, DDX3X, FASTKD2, EDA, ACTR1A, RPL7, PCBP1, CCNG1, RNGTT, BRF1, CEACAM1, ZSWIM1, WIPI2, FOXA2, MED24, C1QTNF1, CUL1, TMEM140, ALOX15B, ZNF706, MRPS22, ERN2, EIF4B, B3GALT5, LECT1, TARS, HDAC7, PAPSS1, DUOX1, FGF16, HNF4A, MAP7, RNF130, CALU, C21orf2, PEG10, ALDH4A1, DERL2, RANBP2, HAUS2, PSMD14, MFF, NR6A1, AGRN, QTRT2, GPRC5B, COPB2, OXA1L, AKAP9, TMEM33, SLC34A1, DNAI1, APOC1, NDOR1, CEP76, SPTBN1, URI1, TCF7, LARP4, MYO7B, VRTN, HIPK1, GRSF1, DPM1, UBE2A, GPR153, HAUS5, GRB10, KDM4B, EIF1AX, CLNS1A, TGFB1, CD4, FRS3, CLINT1, ARID1A, JMJD6, ARPC5, PLCB2, ZNF587, BTBD3, KIFAP3, PIK3C3, TNPO1, TNNI1, UBA5, GGA1, EIF1B, CDC37L1, NARS2, CTTN, COPS6, ACYP1, ST8SIA5, PPIE, BAG2, POGK, NUP205, PGK1, PIP4K2C, RPL14, RNF139, ZNF646, ATN1, ENSA, TARDBP, TXNL1, CALHM2, GTF2E2, RAP1GAP, ETFA, CEP350, SEC23A, DDO, SUGP1, CFAP74, SH3BP2, ELF1, SNRPB, ADAM15, FAM8A1, COPS7A, ID4, SOX9, GYG1, MDH2, LILRA3, ADAM8, HMGN4, IDH3B, KRT38, PSCA, RTCB, KCNA1, TXNL4A, NPVF, FHL3, MTUS2, CHGB, KIR2DL4, TP53AIP1, PDE9A, COL6A1, OR11A1, SS18L1, NUP93, CDHR5, RAD17, GZMM, ACTL8, LENEP, TMED5, LAMP2, CCNB1IP1, DPP8, DAD1, CRX, AP3B1, CSPG4, MGEA5, CIAPIN1, PPP1R12B, UFL1, FUS, UCHL5, TCF3, PAM, 8-Mar, STAG2, SEPT10, SIRPA, ZNF343, TMEM30B, MAPK8IP3, DDX21, SLC39A6, RPS3, KRT2, UBE2L3, MAP4K3, IQSEC2, SEC24D, PSMD12, YME1L1, KCNJ10, COA1, HFE, FAM13B, TBK1, BMP7, TM2D1, UBE2Q1, DHX34, CANX, ICMT, HNRNPD, COL9A2, TERF1, SLC12A7, LANCL1, DYNC1I1, TSKU, ZFR, TOR1AIP1, RARG, DOHH, TAS2R16, USP5, NDUFA5, LETMD1, CDC42EP3, SBF1, PSMD10, TMEM9B, FNTA, CREB3L2, ADH5, TOB1, NPEPL1, PKN2, DIMT1, KCNB2, FUT3, STAM, STAG1, CROCC, FAM96B, BTF3, WSB2, EVPL, BARX2, ENTPD5, FCAR, YIPF6, RGS6, MUC2, SASH3, LCK, PRDM4, PI4K2A, CCR3, CD1A, WIPF1, PSMD1, TCEAL1, MFNG, MED16, ETAA1, C22orf31, PPFIA2, CITED2, UNC93B1, MRPS30, DRICH1, KLRD1, SGPL1, MYBPH, IFRD1, ST3GAL4, C15orf39, INPP5D, CSNK1A1, SQLE, MYO9B, TRANK1, DLK1, PACSIN3, SUPT16H, SEC24B, MKL1, EEF2, RUSC2, PPP1R14D, GPX5, ARIH2, FOSL2, SUMO1, LY9, GFPT1, SOGA1, MAMLD1, TMPRSS5, ANAPC10, CXorf57, ERBB2, DNM1L, RALGDS, PHOX2A, PVALB, IDUA, TCTN3, MRPL39, KPNA3, NEU3, RNF122, BLCAP, KIF5C, ASAP3, RANBP9, PPP2R1B, PSMB3, TMEM209, CSTF3, TGIF2, SLC35E3, CLP1, SS18, ENDOD1, IL3RA, SLC6A20, SLC9A7, SLC25A3, MMP26, SLC7A1, RNF114, API5, MAN1A2, SEC13, ADNP, PDE4D, CACYBP, EXO1, PSMC2, SCNN1A, GPR21, TFDP1, GTF2H3, MORF4L1, ADO, ITGB3, ZCWPW1, GREM1, RALGAPA1, UBE2J1, CHI3L1, AGTR2, PRMT5, ATP5G3, PSMG2, IL36A, TAOK1, PDHB, YBX1, GPATCH8, KCNH4, ZBP1, DUSP11, SULT1C2, TRIM15, CLEC4M, SPI1, PRX, CAPZA2, SLCO1A2, ARL1, FNBP4, NUDT9, ANK3, INF2, UBR5, GAK, KIF1BP, GCA, GLYR1, AIMP1, PSMB1, AKT3, CDS2, KIF2A, TBC1D9, PSAP, RCOR1, LRRC20, FBXO11, ADCY2, ADAM19, SPAM1, OGT, COPS3, TFRC, C1orf27, SGMS1, CCT7, LRRC61, DSCR4, BCL2, CDA, BLM, MRFAP1L1, HLA-DMB, DDX18, NDEL1, HSD17B11, CABP1, CTSO, GAS2L1, TBXA2R, NGB, DST, SMAD6, RUFY3, IDE, TP53I11, IRF5, PARP2, TMEM248, RBPMS, MED17, GCC2, MIOS, WRB, TCOF1, HP1BP3, KLHDC4, CSTF1, BMPR1A, CHRNB2, QRICH1, MED13, ATP6V0A2, ATMIN, MUSK, PSMB4, BTG3, ARFGAP1, GRM3, EIF5A, GPR52, GPA33, ETV1, UBR2, PLXNA1, SLC35G2, MTDH, GMEB2, AIFM1, CRNN, CACFD1, IL1RAPL1, NR1D1///THRA, ECE1, CCL7, COIL, SLIT3, SAR1B, RIOK3, SERPINA7, FASLG, SYDE1, MAST2, LBP, MRPL18, SEMA5A, SPOP, SPIB, SPG20, PNMT, MRPL49, MAGI1, YES1, BTN2A1, TAPT1, SCAF11, SMIM7, NEFM, HIGD1A, EFR3B, ARL2BP, DCTN3, EXOC2, MTR, HDLBP, DOK4, KCNQ2, BCAS2, BIRC7, ALOX12, BMP1, GTF2B, ATP6AP2, ENPP3, APOBEC3C, ABHD11, EFS, RPS15A, SEC61A1, CYP3A5, LSM4, EFCC1, AK1, EEF1D, ALPPL2, ZNF207, TYR, PGAP1, GNAI1, RHBG, SH2D3A, FZD6, CYB5R1, MLH3, MRPL9, THY1, PAK2, SLC13A4, EIF2S2, OSBP, VPS26A, H2AFV, ATP5C1, STRAP, CEACAM21, NFATC1, VPS4B, CRIP1, IL13RA2, AP1AR, CRLF3, APOL2, TASP1, TBC1D12, TRAM1, B3GNT4, ATR, MIIP, GTF3C2, APIP, ORC2, POFUT2, PRKACB, AMD1, DPF2, ACTN2, NTRK3, SPHK2, GIMAP5, ZDHHC6, ATF5, HLA-DOB, SLC4A4, LIPA, HLTF, TBX1, TJAP1, FNDC11, SIRPB1, TOMM70, DBH, CST3, COPE, CHTOP, DNPEP, SMARCA2, NTSR2, NAA35, RALGAPB, SMAD4, CSF1, NCOA2, CXCR3, CCR8, RUNX2, AGFG1, HR, NCAM1, ADGRA2, ADRA2B, HBE1, RHOBTB3, ALS2CL, ITFG1, PKM, MASP1, RCN2, ZNF696, ZNF287, PIGP, UBA2, TRIM44, ATP8B1, ANXA13, ARF4, EIF2AK3, ADAMTS2, EVC, MEGF6, TMEM126B, TNFRSF1B, MT1G, MC1R, PPIB, TBL2, CACNB4, SC5D, DHX16, PLXNA3, SLC25A36, RCBTB2, ALDH1A1, LRRC40, HOXC8, SSX3, SYMPK, SF3A2, ZBED5, PRUNE2, ATP2A2, ABCB9, MAFB, WAC, PREB, PLS1, NCK1, ADGRE1, SEC22B, CRHR2, CLASRP, HBS1L, ACAA2, SACM1L, COPS8, NECTIN2, SLC23A2, ICA1, ERICH1, ECHDC3, BNC2, CACNB3, IDH3G, FYB, PPP2R5C, ADORA1, DIAPH2, KLK13, EEF1E1, POP4, HSPA13, CNOT7, SPG21, MMADHC, UQCRFS1, SMARCA5, EPAS1, GLOD4, NPM1, GOLGA2P5, ZNF444, ITGAE, FZD2, HBP1, UGP2, MKS1, CCL16, CYP4F2, IKBKE, GABRB2, PYGM, ZMYND11, WDR37, NBN, PCMT1, SNF8, TSPAN7, PRRC2A, SLC38A3, INSIG1, PLEKHF2, MAPK11, TNFSF8, SYT12, MRPL13, ACRV1, MAZ, LDLR, PPIL2, LMAN1L, EMC10, CKS1B, MTFR1, SLC25A17, KDM5B, SON, AFF2, DYRK2, LTBP2, GSDMD, ITGB1BP1, TNK1, TROVE2, NOX1, MMP19, LRP1, PLEKHB2, CD6, COX11, SPCS2, RPF1, RHOD, CASD1, AOC3, GCM1, USP9X, BCL2L1, NPPA, GRK5, CES2, HUWE1, NOTCH3, SKAP1, PDCD11, WFDC1, FOXD2, NPFFR1, ENOX2, PEX5, EFNA2, FUCA1, APOOL, GDI2, MAU2, SLC35A3, CD2, TNR, KCNMB3, EML3, CNGB3, SEPT6, PWP1, KIAA0196, GLRX3, DBF4, POLR2C, DBT, RBM41, C9orf3, OR10C1, PTPA, TEFM, PEX7, MRPS27, RHOT2, RAB3IL1, XPNPEP1, KRT20, G3BP1, ERP44, PRR11, ATP11A, PPCS, XRCC6, SLC7A10, CLGN, SHOX2, MFN2, PDLIM7, SLC35D1, PTDSS2, SCAMP1, TIAL1, KCNAB3, LRRN2, KPNB1, THBS1, NDE1, AGRP, GOLIM4, LIPC, TRPC3, KLF11, NET1, CYTH4, FHL5, DNASE1L3, SULT1A2, HERPUD1, NUP160, NRP1, GGA2, VDAC2, SCG2, ACLY, ESPL1, RNF6, PSMA3, PNOC, GPR20, FAM186A, KCTD9, PDSS1, MAT2B, FBXO28, CYB5B, PAPOLA, TTC33, MICALL2, EDF1, RAC2, ACSL6, FAM49B, SPO11, ART4, CYCS, TMF1, PNRC2, SNX1, KIF1B, LY6D, SNX29, CABP2, DNAJA2, TPI1, PSG6, SFTPB, EFNB3, SMG6, OPA1, IL1RAPL2, SNW1, KAT6B, CMTM6, PCDH11Y///PCDH11X, RPL3L, C14orf1, AMPD1, MRM1, HOXC11, TRIM37, ITGA9, CRYZ, ODC1, LAT2, RDH11, CLIC5, CPNE7, CAAP1, PGF, KANSL2, SH2D1A, EIF2B2, RANBP17, RAD21, FAM98A, ZWINT, OSBPL9, ARID5B, MED14, PTDSS1, ZC3H13, PRODH2, HNRNPC, APLNR, STAB2, SLIT2, SPDEF, PPP6R1, RABAC1, IL19, FBXO9, TTC17, EBAG9, MR1, ZNF235, MTOR, SEMA3F, RANBP6, HIRA, HNRNPF, RABGAP1, TOX3, TCEB2, PCSK6, SNRNP70, COG2, PIP4K2A, SIN3B, ALDH1L1, WHSC1L1, LRRC8B, REEP5, DOCK5, GNAI3, ALX4, B4GALT2, PNMA1, ZNF576, PNN, YIF1A, SEPT4, GMPR2, GOT2, ITGB7, CSF2RA, CDK19, DENND1C, MOBP, GPR4, OR5I1, KRT33A, PPP2R3C, INPP1, PTPRN2, FUBP3, NFRKB, PRKAR2A, TNIP1, MRPL15, SRSF3, POLD4, HHAT, DLX4, LAMTOR3, PATZ1, SFTPC, CYP4A11, FBXO21, CAPZA1, UBAP2L, PRMT2, PTENP1///PTEN, STATH, MLPH, PBXIP1, UBE3B, PTPN11, CASS4, GTF2H1, CLCF1, PCNX4, CDC25A, RPGRIP1, TGFB3, EPB41L1, ACAN, CPED1, FRY, SAE1, ASGR2, SMO, ZNF330, TMEM59, TNNT1, SERPINB10, GJA8, MRPL52, DAZAP2, SRP19, TH, RHOH, CER1, CPNE6, MRPS11, OR10H3, ITPK1, SLTM, PDE8A, DUSP12, TRAIP, FOXO4, XPNPEP2, CLDN11, KLHL22, MTRR, EXTL1, RAB11FIP2, NDUFA6, ATG3, RBM3, C1QL1, NUP153, ASXL2, DRD2, TTC9, ABI1, ANXA7, VDR, ARMCX2, EIF4G2, SDK2, EBF2, SNX7, PSG2, SLC39A14, ARIH1, LARP4B, DYNC1LI2, CREBZF, GFAP, GDPD3, CNOT9, CRNKL1, IL25, PMEPA1, GALR1, AXL, RAB5A, PIK3IP1, PAX2, SCN5A, MISP, GPR157, EIF1, HPCA, SBNO1, MVB12B, GLYAT, MYO5C, KDM2A, ATP5L, TRIM31, CLCN1, APBB2, IMMT, ABHD10, GIPC1, FAM175B, GNE, MEF2A, U2AF2, RASL12, ALDH1A2, ASNA1, DLG1, PARL, HSD17B10, NUP107, ELF5, SUZ12, FZD5, FBXO3, NPAS1, MDH1, KLC1, SH3GL3, BCL7A, LRP8, CDKN2AIP, PTK7, DTWD1, B4GALT5, CARMIL1, MLF2, TNK2, NADK, CD3D, FKBP10, SMC4, BEST2, ATRN, FN1, ATP5A1, ETNK1, CARD10, TSPYL5, DNAJC12, TEX30, NUP50, ACVRL1, GGNBP2, ARMCX5, CNGB1, EPHA4, NCKIPSD, ARHGAP33, PLA2G3, SNX27, ZFC3H1, ARF3, AQR, THUMPD2, CHST5, ATP6V1H, CACNA1E, FAR2, FAM20B, DMP1, BTN3A1, ADCY10, SGPP1, SMNDC1, SNAPC4, ABCC10, HIF3A, TMPRSS4, PPWD1, ARHGEF4, CHMP1A, RNF219, WWTR1, ELP4, BTN1A1, NHLH1, SLC1A7, GREB1, NDUFB6, DEGS1, TMCO3, CDK14, SDHC, SSX2IP, ARL3, EPB41L3, SYNCRIP, GOT1, FCER1A, COX5B, ARID5A, NUPL2, MMP14, CDC14B, SCCPDH, TACR1, HMX1, TEAD4, NAXD, DNAJC16, PPIL6, CDC123, NIPA2, SH2D4A, NCR2, PDE3A, DIP2A, ATG4B, DHCR7, GTF3C4, FOXP3, RNF34, MRPS35, C5AR2, TMEM59L, MAGED2, SOCS5, ZCCHC14, ALPL, CD84, MARK4, GADD45B, AOC1, PUS7, PLA2G2F, VEZF1, CALD1, ABCG5, OR3A3, UTY, MKKS, CBFB, GNL1, RC3H2, TRIM26, TRPM6, FGF18, MUC7, SOX10, GUCY1B3, MCOLN1, CREB1, CAMK2A, RPL3, CD300A, ETF1, AKAP8L, COMMD8, KIF17, GDNF, APOL1, AP4S1, MKRN1, C1GALT1C1, DSCC1, TTLL4, ACBD3, STX3, PPP2R5A, ZFR2, IBTK, BBS4, GTPBP4, GALNT3, IL7R, CAPN6, CADM4, ZNF451, ACKR2, PI15, MFSD7, XRCC3, MFGE8, NPY2R, EPB41, RARB, SKP2, CTSC, GPN3, POLR2A, CUL4B, NUDT3, HNMT, NAB2, NCOR2, EIF3D, WDR62, MYCN, UNC45A, RPS6KA6, SIGLEC8, P2RX6, PDCD1, KCNJ13, WDR44, STAT2, PRPH, ZFP36L2, PBX1, EPHB4, COX5A, MYB, ABCF2, AKR1B1, GSAP, KCNS1, PAXBP1, INA, POT1, ZNF710, FPGS, SCML1, PCF11, TPX2, HYAL4, XPO1, ACSF2, IMPACT, NUP98, CAV3, ERCC3, TMEM131, MORF4L2, SEPT9, RIN1, SLC22A7, GABARAPL2, IDS, DHX30, EIF3E, F12, RAB21, STX18, DPYSL4, STK38, C2, RABL2A///RABL2B, LARS2, NACA, DSCR3, UTP18, SLC6A8, NAMPT, CCNJL, SMG5, ASNSD1, NRCAM, IL18BP, MPO, IFNGR1, KDM4A, DEFA4, DAP3, VAMP5, PACS1, ENTPD1, EYA2, REV3L, MYOD1, MAST1, PGLYRP1, SLC47A1, CSGALNACT2, STXBP1, IFT22, FOXO1, PLXNC1, VAMP3, KPTN, PDE6D, HLA-DOA, DBN1, TMEM230, CA12, FARS2, INPP5F, SGCD, ZNF202, RPH3A, ZNF302, SPATA5L1, GINS4, GABRR2, CROCCP3, IKZF1, FCGBP, CHD1, FCRL2, TMPRSS3, GABRA5, MKL2, MRPL2, EPB41L5, KCNE2, ILVBL, EIF2S1, ITGAM, HAND2, LAIR1, TFDP3, KCNAB1, IBA57, NEK1, DYNLT3, BOK, SORL1, PLA2G6, CDH15, RPL8, SNRPD1, HSPB7, C2CD5, FN3K, RNASEL, SHQ1, ATP5S, NKX2-5, DGCR2, TRIM25, NUDT21, AMBN, CDK7, KDM5C, RNF41, ZC3HAV1, PLXNB1, RSRC2, CDKL2, UBE2D1, FSCN1, TBC1D5, GRIA1, FUT2, PLEKHA5, ATP2B2, TSC22D2, RPS6KB2, ACE, RAPSN, ST8SIA2, RASSF7, FXYD7, PRMT1, CASP4, RAD54L2, HBEGF, RIMS3, INTS3, PBX2, PPFIBP1, APMAP, ATP6V1E1, ANAPC2, ITIH2, SCG3, HOXD9, TBCE, TTC22, WRNIP1, GSN, ROBO4, TROAP, GOLGA7, PIK3CA, NEK3, KCNK2, PTPRD, TMEM47, CUL4A, HSPD1, RBM12B, GABBR2, JTB, SMAD2, VAC14, XPC, PRPF18, ZBTB40, TNPO3, SSR4, NCOA1, SLC22A6, HOXB1, NCAPH2, MINPP1, ZNF428, RPL11, PAAF1, SIRT2, ATP9A, PEX16, FAIM, KIZ, TRIP4, FASTKD3, NKX3-2, TAL1, TWF2, ARID4A, MYBPC3, INTS8, AKAP7, IKZF3, HAPLN2, PCDH9, EIF3M, AR, SENP5, PQLC2, TUBA8, SRCAP, SNTB1, MSL1, UBE3C, SNAP25, TXNDC9, NPR3, PECR, COX6C, DEF8, MEPE, MBTD1, PTPRC, ARHGAP1, ATP6V0B, SUB1, GCFC2, PLPPR2, GTPBP1, GHITM, ACTR10, TUSC3, DR1, BRS3, CNTN2, LTA, C8orf33, RAP2A, SFPQ, PRSS22, USB1, OTOF, GM2A, DHODH, PRPF8, ARHGEF38, PHACTR2, PTGIS, PICK1, FES, CAPN5, RPS6KA3, CDX2, PIK3C2A, ETNPPL, FAAP24, EPS8L2, RSBN1, EIF3I, ENG, EXTL3, ATRAID, CA7, SCGB2A1, ART1, ZSWIM8, RAI1, TBX5, CR1, CGRRF1, CXCR5, STXBP6, ADNP2, GHRH, CADM3, ABCD1, AVPR1B, MAP4K1, DENND5A, JAK2, GLG1, INHA, PROZ, LGI2, DCUN1D4, GOLPH3, IHH, PCCB, ELK3, CREB3, NOC4L, KLF9, CUEDC1, KCNMA1, PON2, HSF2, CASP2, SMG9, ARFIP1, USP25, POMT2, NAT2, MYCT1, AOC2, TRMT61B, KAZALD1, ITGA7, PER2, BLVRA, BIRC5, DHRS12, MAGED1, OR1G1, STUM, TAT, FRMPD4, TMBIM6, PTCRA, WNT11, TRAPPC13, EFR3A, ADCY3, RAD23B, NCKAP1L, SLC35A2, ORC5, PLEC, GCKR, SYPL1, CHD1L, FOXL1, TMED1, TACR2, MOSPD3, TOR1B, WWP1, COMMD4, GPLD1, ZKSCAN3, PNPLA3, SPTAN1, LAMP1, AGFG2, SEMA6A, RPS14, ABCA6, SLC10A1, ARMC6, UFSP2, PIGA, CD8A, FAT1, BMP15, TERT, HNRNPA3, LRBA, GRAMD3, CDK5RAP2, ROBO1, GSTA1, ACVR1, SMARCA1, SLC25A46, MNAT1, GUCA2B, FGFR1OP, N4BP3, BMP8A, ARMC8, SULT1B1, LOX, KCNN1, TCERG1, ERBIN, DEFA6, TRIM38, TMEM161A, PLEKHG6, CD86, DDX51, ADGRL1, PRKAA2, TIMP3, BMPR1B, MCM9, NKX3-1, MAPK8IP2, SYTL2, KCNAB2, OSBPL11, CUTC, CETN3, IL13RA1, PDX1, ZNF160, NLRP3, GNA15, TXNIP, MYOF, WIPI1, PRKCSH, PPP4R1, MAP2K6, CCL21, NECTIN1, NPEPPS, ACOT7, WASF3, OPN1SW, MARK2, DLG5, TSC22D4, ME1, SF1, CDK10, HRG, PDE1B, MLC1, CTDP1, USP24, RPL41, GFRA2, RBX1, CALML5, PMPCA, MTHFR, WDR76, RAB22A, BTD, PLD1, RDH16, CD36, PZP, MARK1, DBF4B, SP110, SMC6, ETFDH, GAP43, HMOX2, NTNG1, ITPR3, CHPF, RIMS1, PPARD, NPTXR, SLC39A7, DCAF4, APOL3, TSKS, GOLGA4, CFB, HES1, EXT1, GPD2, EGFL7, DTNB, RARS, NUSAP1, PNISR, DRG1, ADCY6, NGDN, ATP6V0E1, CTSB, ESR2, DAO, UBXN4, IFITM1, GABRG3, ZNF609, SLC6A2, IGSF9B, MRPS15, CCNT1, SCN4A, DPEP3, CA5B, VPS54, ONECUT2, PPP5C, TMEM87A, TMEM50B, EFCAB14, SESN1, JAK3, CHIT1, EHMT1, GLP2R, SLPI, EPB41L4A, MAPK12, IFT52, RAC1, MAGEC2, CDC23, SCFD1, QPCT, RNPS1, SMPD1, PON1, RBM48, CCPG1, CTBS, OPLAH, SLC39A8, IL18RAP, ITM2A, SOD3, WHRN, THSD4, CLTC, MATK, RGS14, ECEL1, TSPYL4, GPR1, NAA50, TCN2, SLC28A1, CD40, DYRK1A, PAPD7, TSR3, SLC27A5, KCNMB4, VPREB3, SLC19A3, DSC1, HSPB6, INPP4A, TNFRSF10B, MED28, RRP15, CSDC2, CD200, SGCG, TLE6, FBXO42, GSTM3, SPSB3, PROP1, CCT6B, CHRNG, PTBP2, EML1, PDPN, RWDD3, CCKAR, COL11A2, AKAP10, IL27RA, STAU1, SENP6, MBTPS1, ACVR1B, EGR4, GPR162, MRPL35, PRR36, ARL8B, PTP4A3, MAP1B, IL4, MCL1, WDFY3, ADRB1, COX7A2L, MPHOSPH6, DNAH9, RENBP, PLA2G5, SHMT1, CTNND2, UNC5B, KCNV1, N4BP2L1, FEM1C, MAP4K5, NAA60, WNT1, NHLRC2, MRPL28, ALAS1, DCLRE1C, NECTIN3, CD160, TMEM223, ACTR6, PRRX2, CDKN1B, ATP6V1D, CDK8, NARS, AQP5, NAE1, TRIP10, CHRM2, SPAG9, PPID, NMRK1, CYP2E1, UBE2E1, XAB2, POU3F4, SLC39A9, SPTBN5, CYLD, SH3BGRL, GJB4, MAPK8, RABGGTA, KCNA2, PRRX1, KIF1C, DAXX, OPRPN, UPK1A, USP4, SPATS2L, IARS2, RNF4, GPRC5D, TFPI, ESRRA, ACTL6A, NREP, RGS4, EIF3CL///EIF3C, WSB1, SH3GL2, SLC11A2, ATG5, ITGA8, SPATA2L, PARP12, SOCS6, SRRT, STRN3, PREP, PRKCB, CLPTM1, GRM5, SGSM2, MAPK4, LYST, ALPP, RRAS2, IFT122, DAB1, CACNA1I, STX16, NFKBIB, PSG9, NR2C1, TG, TDG, SRPK3, DDR2, DNAH17, EPHX3, PPM1B, FMNL1, PRPF4, SYT13, FOXB1, CFH, KALRN, TFIP11, ACAD10, KIF5A, LYL1, BRSK2, CRTC1, TBX2, PEMT, HSD17B12, FAM13A, RFXAP, COL5A3, AKAP1, IMPG2, LAPTM4A, DCAKD, TRIM27, KCNN3, ASIC4, HIPK2, C16orf72, CRK, TMEM30A, MAPK1, SLN, RAB30, HLX, OBSL1, CABYR, ARRB1, TM4SF5, PDCD2, MPPE1, CDK18, ROCK2, FCER2, LRRC15, MAPKAP1, RAD52, SPTLC1, IGHM, SETX, NCAPD2, NDST4, THBS2, HGF, HS3ST1, ROR2, MFAP4, BTNL3, IQCA1, OSM, CPN2, GPR137, PGLYRP4, PKD2, POLM, ANK2, RFC1, DDX41, DOCK9, LUZP4, DHDDS, ACHE, THOC5, PRRG3, EHBP1, MTMR1, RNF7, GRTP1, LPAR1, CLTA, RNF11, GNL3L, KIAA0930, MAPK1IP1L, ATP6V0D1, PAX8, C22orf24, TTPA, C10orf12, NR0B1, PAPPA2, SNX17, ATXN10, SOCS1, AMBRA1, C6orf47, PDGFRA, FBXL18, TMEM14A, RAPGEFL1, GRIP1, HBA2///HBA1, MEF2D, RAPGEF2, TNKS, EPS15L1, PLIN1, OR10H2, MAPK3, CXorf21, NDUFAF4, NCDN, ADGRB1, SLC35A1, DHRS2, MFAP5, C11orf16, TP53TG5, CLIP1, LRRC41, CNOT2, HNRNPK, PDE10A, WDR78, OMD, DNMT1, NR1I3, KCNH1, C3orf14, UTS2, GCH1, ZC3H3, NRN1, ABCD3, PLXNB3, ADCY9, ARHGAP45, RPS6KA4, FANCA, PRMT8, SURF1, GLRA2, GATA2, LSM2, SORBS1, ZP2, KRCC1, TINAGL1, CDKN2C, ENO3, FURIN, RPS17, RAB3A, FUZ, UBA3, CSRP3, GUCY2F, CAV2, LRFN4, UIMC1, AVP, SV2C, RTN3, EPN1, PCGF2, CYHR1, MAGI2, CAPZB, DUSP9, TRIL, TM9SF4, HIVEP2, MYOG, EPS15, RBMS3, HUNK, DLG4, ARFGEF1, INSM1, ID3, NEUROG1, TMEM5, TEF, ID1, AKAP5, SLC38A7, HTT, YY1, F7, REST, GIGYF2, SERPINC1, BCAN, SLC20A1, CCDC57, SLC35A5, NOS1, TEK, CPS1, TNS1, TNPO2, SAP30, TLX3, TNFRSF8, PARK7, PRKG1, FRS2, DYSF, SEMG1, CNR1, NDUFA10, CBX1, PTGER3, ELAC2, NR2E1, HOXB3, DLAT, PHTF1, NRG2, CHPT1, PTPRT, MINA, ADAM3A, COPS4, ATP4B, PTGES, CD33, ZNF226, TRAPPC9, BLK, STAP1, AAK1, RNF115, MYOZ1, NTRK2, NRP2, IMPAD1, CDC7, SORBS3, FOXL2, FOLH1B///FOLH1, GATAD1, PLPP1, TP73, ITM2B, USP2, ADAM10, PCGF3, PEPD, P2RY2, HDAC11, NOD2, CHORDC1, SCAF4, TRIM3, INSR, HIST1H3I, ALDH18A1, IGFBP5, AMN, SIGLEC1, APPL2, MYO15A, ITSN2, RPS10, RARA, PLCB1, MTMR14, ETV4, SZRD1, SNAP91, MLN, KEL, ARF5, RCVRN, PSMC6, XPNPEP3, MAP3K9, CASP10, NSD1, FNBP1L, SRSF1, RB1CC1, BRE, ALX3, PHF7, HLA-DPA1, GGCT, PSMF1, ICAM5, ASGR1, PDS5B, MAPK9, CHD9, KIAA0907, CACNA1G, HEYL, USPL1, NUDT15, TRAK2, MYO1C, LIPE, SAMD4A, SPTSSA, KCNJ3, NDUFAF3, PCDHB1, GRIK5, RAP2C, IGFBP1, NOP10, RAF1, SH2D2A, LIMK1, ADGRF5, VAX2, KCNC4, CD19, SH2D3C, SSBP3, MRPL34, IL17B, ADK, NRG1, GPR31, RTCA, CHRNA6, SLC1A1, MBP, MTERF3, PLD2, CYB5R4, TCTA, PRAME, SAP18, ADRB3, PTPN1, LEMD3, PUM2, TRAK1, OSGEP, PASK, ASAH1, MYO1B, PCSK2, AVPR2, ENTPD4, PPP1R8, PSENEN, TTC27, EGFR, NGFR, CCNB2, ADAM30, BMP2, MYH11, NPR1, SQRDL, TPM2, LILRB2, LSP1, KIDINS220, FBXO7, CLCC1, METTL7A, FOXJ3, ID2, SPSB1, MSH6, CLDN17, NDUFA7, AUH, CLPX, AGTPBP1, PYGL, ADD2, MUC3A, NUP85, LOC202181, SPOCK2, GRAP2, CHCHD3, ARHGAP32, FMR1, CYB561, ADH1B, TAF7, PIK3CD, ERF, CHN1, FCMR, GABRA4, LRRC3, EIF4E2, UBE4A, DDC, ZBTB17, MAOA, AIM2, APTX, RXRG, MGAT3, AKR7A2, CST5, PARP4, FAM214B, ATP1A2, CHP1, PTGDS, MYDGF, EPHA1, PHF3, VPS50, HDAC6, ERCC4, RAB6B, AKAP4, SRD5A2, OPRK1, DUSP21, PTGS1, TSPAN12, CLEC1B, ODF1, CXCL13, VWA1, THEG, ADRA2C, ALOX5, RNF103, ZFP64, C1QB, CES3, ZNF79, NTRK1, KCNC2, ZMYND10, HOXB7, MED8, C1orf109, TNFSF9, HTR1A, LILRA1, ELK1, S1PR2, DRD4, CERK, PDLIM4, TGM4, PTPN12, 7-Mar, OLAH, ZSCAN18, LMAN1, NPHS1, TSPAN3, CHN2, RPIA, GALNT12, HRH3, EIF4A3, NES, HSP90AA1, TMEM268, RARRES1, CD2BP2, AHSP, ARHGAP22, FAM174B, SWAP70, CYBB, GTF2E1, SNRK, ADAM11, MAD2L1BP, APH1A, OPRD1, KCNK1, PAFAH2, GLTSCR1L, CRYM, ZNF669, GUCA1A, CLSPN, CCDC91, IL5RA, DAPP1, ADRA1A, SUMO3, SFI1, OLFM1, PTP4A1, FOXN3, STAG3, FAM3A, CHMP1B, ONECUT1, PARK2, ELAVL3, RHCG, FBN1, PHF24, KLF12, SKAP2, TNS3, MDFI, BRD2, USP22, METAP1, KLK2, AMOT, UFD1L, ASCC2, DRD1, PALB2, BRAP, TSSK2, SLC17A7, CXXC1, SLC1A2, SCPEP1, CDK6, B3GNT3, FOXC2, SOSTDC1, POU3F2, NDUFAB1, DCLRE1B, WAS, GML, DDN, RBM17, COMT, VPS13C, CLTB, BAIAP2, YRDC, ARMC1, LRFN3, HOPX, QKI, LRP6, ZNF132, CREBL2, OCA2, NXPH4, VSX1, DCHS1, NUP210, CEP164, BTBD1, NSFL1C, PHF8, LSM6, ATAT1, PKP1, CEP192, PCTP, MTMR4, RAB27A, ITPR2, SPTBN4, LYN, PDZD7, NAT8B, DSCAM, AIDA, HEXA, SKIL, RPS6KB1, ARHGAP26, RBCK1, SRBD1, GK, COL1A1, INSL5, ASH2L, MFSD11, MEF2C, GRIN2C, PPAT, SLC25A12, TRIM33, CLIC4, DSE, CDK13, SLC4A7, GOSR1, SEMA4C, GPSM2, TBX6, A4GALT, ETV7, BCL2L14, ASIC1, FERMT2, PRF1, TAF1, SIX5, SRRM2, CAMSAP2, SNCB, SCLY, MTUS1, C6orf15, PSMA2, FTCD, ZNF143, LTB, ABHD4, GSDMB, TUBB4A, PURA, PDLIM5, TACR3, JADE3, SNRNP25, CHRNA10, CTPS1, GLCE, IL2RG, RNPEPL1, MNT, E2F4, PRPSAP1, PDGFRL, RTF1, MKI67, RNMT, CALCR, PDPK1, BUD31, MYOZ2, CCDC85B, MPHOSPH9, KLHL24, RIT2, APBA1, MLLT1, TELO2, TRPM8, SETD3, FKBP6, CA5A, SNX15, RSAD1, LETM1, TMPO, ATP6V1G1, APOA1, HAND2-AS1, WDR1, TBCC, CETN2, GJB3, PDZD3, TRIM10, BRINP2, GALNT1, ZC3H14, PF4, HADHB, ZFAND6, TBC1D29, TAOK2, AKTIP, CBX2, PIGG, MRPS33, HSPA9, CSK, SIPA1L1, DENND1B, GRHPR, MVK, 1-Dec, ZNF318, SSR2, ANKRD12, MYOZ3, CCND2, CSNK1G2, EMC1, HGH1, HTR3B, BIN2, TCAP, RANBP1, ELAVL1, RBMS1, GZMH, SEMA4G, TAF4, PCDHA5, C1orf105, POLR1D, NCF4, MIA, SKI, GOLGA2, VASP, NEDD4L, CSNK1G1, MFSD13A, ARHGEF15, BAG5, ATAD2B, CRHBP, SLC2A1, DNAJC4, CAMK1D, SEPHS2, PRNP, CHRNA1, IMP3, TM9SF3, PAIP1, COLEC12, CACNA2D1, TDRD1, C12orf10, ABCC8, TMLHE, MBNL2, SLC28A2, MAGEA12, MET, SMC1A, FPR3, ATG12, DCHS2, MED23, MAPK6, MOGAT2, ACPP, PTGIR, PSME3, DARS, ACACB, AGTR1, DNAJB12, TRIM24, NKAIN1, GLRX5, GPM6A, NMNAT2, TMC7, LSR, PAPOLG, FCN1, LYVE1, TRAF3IP2, ZBED4, AATK, E2F6, PDK2, FAM135A, AKT2, C1QA, CST7, TNFRSF10C, GPRASP1, HBG2///HBG1, GIF, UBTD1, CHMP2B, PLXNA2, RORC, NONO, CRYL1, PRCP, IL12RB1, EFNA3, CYP26A1, KCNJ5, EIF2AK2, CEP170B, CDK16, NDUFB4, MAP4K2, CCHCR1, LDLRAD4, CNTNAP1, B3GNTL1, HDGFRP3, TRIAP1, ZC3H7A, FMO2, PLOD1, DUSP14, LDB3, TFCP2, MTHFD2, ARSA, FLI1, MZB1, RNF111, OAZ3, PCIF1, KRT37, GJA4, BZW2, FPR1, FGF21, IKBKG, CASQ1, NRF1, UBE2D3, FGD1, CRYBB1, SNAPC3, YWHAH, IFNAR2, RIF1, RPAIN, RNF8, SLC48A1, ADCYAP1, DPY19L4, SIAH1, CDKL5, FA2H, AQP6, MASP2, MUTYH, MPZ, C5orf30, ATP2B3, ATG9A, FOXC1, TANK, FAM64A, SLC12A4, PIGV, RINT1, TMEM206, USP49, FBXO24, ADAMTS7, SUCLG1, GPR161, HSBP1, PCDH12, ICE1, REL, MED1, SMARCD1, KIF21B, ZNF146, REEP2, CELF3, ZNF224, RAB11B, RABEP2, TYMS, CASP5, GNB2, PIGO, ZNF205, LMO4, BNC1, ZFHX4, CDSN, GPATCH4, HCRT, ALOXE3, PTPRJ, ALOX12P2, KLHDC10, SMC2, NDUFS1, TEX10, SARAF, TTR, MAL, SLC1A6, CABP5, INSIG2, CAMK4, TEX13B, FAM89B, EFNB1, ESPN, CA6, HPSE2, TAS2R4, TNNT2, EDN3, ERO1A, CYTL1, PDE3B, TBCD, TIMP2, LIMCH1, LIPF, IL1RL1, PRSS23, CTIF, RHOT1, LHPP, G6PC, IGHA2///IGHA1///IGH, PLXDC2, PICALM, RNF24, FRMD4B, HOXC13, MITF, YWHAZ, FZD3, SAGE1, CERS2, STAC, PKIG, OPRM1, PCK2, JUN, KLF6, HSPA4, COL4A3, ANKLE2, ALPK1, NEUROD1, CATSPERG, OLFML2B, JPH2, ADAM23, CELSR1, ANGPT4, LEF1, IDO1, RBPJ, PHKG1, TBC1D2, MORN1, KIAA0368, SLAMF7, GPD1L, DVL2, KRT4, NDUFB5, SAP130, KARS, THUMPD1, VRK3, DXO, E2F5, BAG1, ANKS1B, GC, CTSZ, TCP10L, IFT27, MYO9A, AZU1, NEK2, B4GALT1, CYP3A43, TIMM10, KLC2, MYT1, NCOR1, GNAT1, ACADS, NXPH3, ALG12, CD9, SOS1, OSGIN1, DRP2, APC2, MCM7, CTC1, AFDN, WNT7B, MERTK, C6orf25, GLP1R, AFF1, NOL4, SMPX, TYRO3, SIRT3, ZSCAN12, IGHMBP2, BAAT, JARID2, GDF7, BRD7, STARD8, EPB41L4B, GALNT6, CD5L, ATXN7L1, NPRL2, ALLC, RPS19, ERAP1, PAK4, SIT1, SNX10, CD28, TRAPPC12, CST2, NBEA, TRAT1, SLC17A6, CYP2J2, STAMBPL1, MPHOSPH8, GABRR1, CTDSP1, HPCAL4, FSHB, ZKSCAN8, XRCC1, CACNA1F, KCNE5, GIPR, FAF1, DIRAS3, MCHR1, WASL, DLX2, PADI2, CCL22, LMO2, SFRP1, PSG1, TMEM106B, SCD, ALDH3A1, UTP11, SRC, IRX4, NSA2, PSEN1, NOL8, MACF1, ITGAX, PKNOX2, HPD, SMAD3, ASCL3, MAN2A1, PLCB4, ANGPTL2, GGA3, RHOQ, SAG, COBLL1, RSPH6A, PRPF39, R3HCC1, NODAL, ZCCHC10, DSTYK, PPP1R13B, UTP6, TRIM52, REV1, CFAP70, NCKAP1, HSD17B4, WDR48, GRK2, FKBP8, OR1D2, FGF1, APOBEC2, NKX2-8, CENPM, ALCAM, RSG1, INPP5E, NCR1, RPL18, HTR6, WT1, GABRQ, ST18, BTG2, PFKL, UBA6, SLC18A2, TOB2, HLA-DQB1, AFF3, PLXND1, PTMA, CCT5, TMEM168, ZNF106, USP39, ZNF280A, IL6ST, INSL3, IFNW1, FXR1, ASIC2, NOS3, SELPLG, PKLR, RNASE3, APOBR, ATP6V1C1, DKK2, UCP3, SLC25A44, CCR4, C10orf88, COG4, ADH6, RPL23A, SIRT1, CHGA, TCIRG1, NOP16, NIT2, PPFIA1, KLF15, PCOLCE, SLC15A2, BCAP29, TULP1, MYCL, SLC29A2, LLGL1, MPP5, ACTR2, C11orf24, PSMC4, BEST1, FOLR1, TRMT61A, SHC3, TCL1A, GOLGA1, C6orf10, PPP1R17, SHB, RNASE6, FABP2, PPM1D, WDTC1, EIF5B, FAM60A, SST, GPR15, CCDC28B, RBBP7, PSIP1, CFLAR, FETUB, ITPKC, BCOR, ZBTB10, RNASE1, NDUFA2, ACTB, ZIC4, ZNF556, LDLRAP1, SULT2B1, RGS7, BTRC, ACTR3, HIC2, ZCCHC8, FAM3C, CEP83, OR3A2, DYNC1I2, CHD4, MSMO1, CKMT2, BUB1, PFKFB2, SEMA4A, WNT8B, FSD1, TMEM186, IL2, SLC29A3, TADA3, WDR73, DLGAP4, CROCCP2, NGF, SOAT1, AHCYL1, PCID2, KCNJ6, NDUFV2, MPP2, LYPLA2, WNT10B, KIAA0391, SLC30A6, COQ8B, SAYSD1, HIST1H4G, CCDC81, IDI1, SUPT6H, POLI, LOXL2, MDN1, NKTR, CD14, FAM110D, SARDH, C1orf21, OR2F1, ASTE1, MC5R, SLC6A11, VCP, CEACAM3, INPP5K, FTH1, IQCC, LMAN2, CENPI, FRYL, WNT2, ACOX1, OPRL1, STK11, PLXNB2, SOX5, FAM215A, UBE2C, PID1, ADCY1, INTS7, BCL11B, HOXD1, OGFR, SNTB2, DOK1, CLEC11A, UMPS, CD22, MAPK14, FAM162A, SLC2A2, DYRK1B, OLIG2, SLC25A42, GLRB, CEP57, NAT1, GPR22, TCEAL4, SUGP2, NDNF, ITPKA, PDXK, ARHGAP29, KRIT1, MYLPF, SREBF2, COA4, PLAC4, IL37, ATOH1, RTP4, SAP30BP, MYOM1, STS, CHST8, TUBGCP2, UNC93A, UBQLN2, FGR, CACNA1D, ATP5G1, FGF23, ZBED2, TEC, ABLIM3, EYA1, FUT5, F2RL2, PLCE1, USP3, TPPP, TM4SF20, NAPG, CFHR2, KLHL20, ISLR, PLP1, ZNF629, SEMG2, MAP3K10, PIK3R5, DHX58, C7orf49, NDP, PIM1, VIPR1, RELB, PRG3, ABHD5, ADGRG2, ABHD8, TEX13A, OR7A5, IFT46, TFG, PC, TGFBR3, FEM1B, GEM, ITGB1, SUPT5H, TARBP1, CCNH, PRKCA, MBOAT7, SOX17, PFKFB1, CACNG2, EBI3, ACO2, PSMA7, MTMR9, STMN4, LDHC, TMCO1, XRCC2, SH3YL1, PAX1, NOSIP, STAR, PCDHA1///PCDHA2///PCDHA3///PCDHA4///PCDHA5///PCDHA6///PCDHA7///PCDHA8///PCDHA10///PCDHA11///PCDHA12///PCDHA13///PCDHAC1///PCDHAC2///PCDHA9, PDE5A, RNASEH2B, BFSP1, FXN, POU2F3, SLC25A4, IQGAP1, DMPK, CRCT1, RPN1, CCDC90B, IL21R, PDE6G, MCTP2, CFHR5, LEPROT///LEPR, RAB29, GSE1, ZNF614, RSU1, SDC1, LOC81691, PSORS1C1, LONRF1, HS3ST3B1, KRT85, BNIP2, TET3, RNF17, ZNF81, DNMT3A, HOXA11, SERINC2, FDFT1, TGFB1I1, KLHL11, TMEM258, DTX3, LAGE3, KCNE4, GZMA, ARFRP1, EPB42, RPP14, APOH, NDUFAF5, IL2RB, HECW1, SPCS3, CD180, BMP2K, FLRT1, ALOX12B, LGALS14, CTCF, ITGB3BP, ARHGAP4, TRIM14, PUS3, SCN2A, POLR3G, KCTD13, KRT16, CCT8, NOVA2, HSPA5, RAB9A, FH, PDIA3, SPAST, DMWD, SNU13, METTL3, TACC1, EPOR, CD58, TLK2, RLBP1, NGLY1, LCMT2, LCMT1, ATP6V0A1, KRT84, RXFP3, GBAS, TGDS, ARHGAP44, BDH1, DDX50, RAB23, ATF2, RBM10, IRF8, PPP2R2B, STRN, SIX2, ALDH5A1, SP100, SERPINB1, GPR12, CCT2, ZMYND8, STAT5B, LAG3, RETN, PREX2, P2RY10, PMAIP1, TRMT13, LAX1, ADGRL3, SDCBP, P2RY13, ZNF839, SVIL, MRPS14, JAK1, FOXG1, SRI, BNIP3, RASL10A, SERPINB4///SERPINB3, C7, SORBS2, PRUNE1, MTM1, RBM5, SLC4A5, PYY, GLI3, CNTD2, GNG13, HNF4G, OR2H1, XPO7, COLEC10, SP3, ROBO3, S1PR4, CCDC7, PTPN22, CYSLTR2, DNAI2, PTK2, B4GALT3, POP5, CMPK1, TMEM38B, SHMT2, POLR2D, PHLDB1, CHRM4, APBB1IP, TTC30A, TEX15, CPN1, HAPLN1, CRYZL1, MBNL1, DBP, ICOSLG, ANKRD27, POU6F1, METTL5, ARSD, HOXA1, WDR26, DCBLD2, TCEAL9, MMP25, PRKDC, ABO, PSMA5, ZIC3, CHRNA3, NCL, LMOD1, HEATR1, DDX58, SLCO4C1, SERPINB3, SEMA3G, TSEN2, TLE3, C9orf116, YWHAB, ARNT, BSG, COLQ, LFNG, EGR1, RPS6KA2, HTR5A, MFAP3L, ARHGEF26, AJAP1, RCBTB1, ARHGEF9, TLN2, FER1L4, CLCA4, MRPS2, LIMS2, PYGO1, ARHGEF11, USP8, NDRG3, PPM1H, FBXO31, CAMP, FLII, DPYSL2, SEMA3D, EIF3F, SLC18A3, CDK17, LIMK2, CDC42EP2, SACS, ITGAV, KLK7, TGFBRAP1, NRIP2, DSTN, SLA, VPS72, NEK9, CRABP2, RMDN1, DMD, MAP3K2, APOL6, AEBP1, LGI1, RIN2, APOL5, PRSS12, TNNI3, MC4R, COL14A1, GRIA2, ATP2B4, TMPRSS15, MARCKS, EPHB3, TIPIN, SLC9A8, USP10, FLVCR2, GTF2A2, LPAR3, WNT5A, CNOT1, VPS45, DCAF6, ARHGEF17, VRK1, ACSBG1, ARHGEF7, CWH43, TSC22D1, PTPN18, TRIM29, LY6E, ST8SIA4, TRIM9, ATP2B1, XDH, CTSL, MPZL1, NOTCH1, SLURP1, CLN3, CLOCK, RASSF1, PROX1, SIRT5, HRH1, PCNX1, SPRY2, TRA2A, EXOSC8, CDC42, POLR3C, AMOTL2, TCF15, C8orf44, SPTLC2, HINFP, KCTD12, NFU1, CLCNKB, ASUN, CDKN2D, NEU1, DNAJC8, YBX3, MBD2, RASGRF1, PPP1R3D, MOSPD1, FAM83E, KAT5, HIST1H2AK, PRRC2C, B4GALT4, HNRNPU, PRL, CYP27A1, CLASP2, THPO, PTGS2, ING2, MRE11A, NPAS3, SEC61G, WDR7, DMXL2, EDDM3A, PDE7B, CRADD, MYO15B, MEIS1, CFAP45, CTNS, CRISP3, TSSC1, WLS, GPR45, HCCS, PDZD8, RRP12, LAPTM5, CLUH, CUBN, MAP1A, SGK1, ZNF589, SLC30A10, PFDN4, FSTL4, ZNF276, ATP2C1, VWA7, FBXL14, AQP9, GFOD1, PTGER4, PPP4R3B, SEMA3C, CHD2, HNF1A, ZNF304, DTNA, CCNE2, TAB2, SLC30A3, YIF1B, SMCP, EBP, PEAK1, ZNF415, SNRPB2, PCLO, RANBP3, GGCX, MS4A1, CACNA1C, MLF1, HEATR6, USP14, PDIA2, TBX4, FANCE, SYNJ1, CACNG4, RPL38, PRELP, SIGLEC9, DIDO1, CRYGD, BCL2L10, GPR182, TNP1, LRP2, GRK1, MSR1, TSG101, MDC1, TRMO, ZBTB7C, DNAH2, HPX, BORCS6, PHLDA3, NOS2, CBFA2T2, TXLNA, PLAUR, FASN, BATF, CLCN4, ANKRD26, RNF170, BHMT, NFYA, GRPEL1, MAP2K5, TFAP2A, PELP1, SLC2A4, CTNNA3, TTC13, C2orf54, CCDC71, KLHL41, PQLC3, HK2, MUT, KDM3B, FAM188A, KCTD15, MAPK7, FOXO3, ZBTB32, ACTN4, TM2D3, KCTD20, CHIC2, CACNG3, SLC34A2, C3AR1, PPP1CB, ZFYVE16, PDE4DIP, ZBTB44, TNIK, FAM120C, HPS1, S100A4, KCNMB1, ANGPTL4, CELSR2, TMEM19, HNRNPR, PELI2, NEU2, GNPTAB, MYRF, LOXL1, RGS19, SEPT7, HAL, MDM1, CLEC1A, NUP155, CSTF2T, HMMR, IVD, DOCK10, TUBAL3, CYFIP1, HAGH, GMPS, INTS6, RHO, WNT6, MAP1LC3C, UGGT2, VPS13B, DZIP3, TAAR2, PHF20, TMEM123, TCEA2, MLXIPL, ZDHHC13, 5-Mar, ZCCHC4, FAM189A2, ULK2, TCEAL2, SIKE1, NPAT, CCNT2, SBNO2, ATP1B2, CACNB2, CDKN1C, RMND1, MORC1, A4GNT, BTG1, ABHD3, FMO4, PCMTD2, GJA3, PPP2R2A, NAPA, KDM6A, LMF1, CDHR1, SYNJ2, MAP3K13, KLHL7, DDX3Y, NMT2, PTPRS, SLK, GABRA6, VEGFA, NLGN3, SP1, SLC4A1AP, ZNF281, DSG2, YLPM1, FST, PIP4K2B, ELOVL2, QPCTL, SNAPC5, AHSG, FEZ1, IGFALS, ORC6, MSL2, ZNF701, CSNK1G3, GART, NSL1, CDC42BPA, DDX6, NFKBIA, IFT57, UCHL3, UPB1, NIPSNAP1, UBAC1, EHMT2, KCTD2, FAT2, ZSCAN32, TLR8, MPRIP, PHLPP1, MAPKAPK5, CCDC170, ANKRA2, CLU, DPY19L1, VCAN, SAV1, POU2F1, COLGALT1, SGCA, TSHZ2, SYNDIG1, TESPA1, HRH2, ACSL3, CCDC85C, FRAS1, KRBOX4, PEX13, ABCG1, PSMD9, PPP2R1A, TAX1BP1, MELTF, PLA2G2A, HS1BP3, FOXN2, TMEM159, GABRA3, PRND, HYAL1, ADAM5, E2F3, LEPROTL1, GCNT2, EXT2, MAGEL2, ITGB8, NPC2, GABRD, SIPA1L3, CD5, RASSF4, SPINK4, ZNF529, FLOT2, EMG1, YKT6, TRPC1, MRPL23, ZNF257, PLK2, PLEKHA1, NRXN2, DCT, ADGRF1, SLC50A1, ZMPSTE24, SIGLEC5, AOAH, STMN3, SPTB, MEX3C, ARHGAP5, CD82, AP1S1, LYZL6, HOXB8, RPRD2, SLC38A1, CNIH4, IGLL1, STARD7, AGAP2, TPM1, GSK3A, MST1R, ABCF1, INPP5B, GRIP2, PLK1, IQCE, DESI1, ATP5J, BMX, CDH11, SLC9A1, APOD, IGF1, BIRC2, STX2, ZNF507, KDM4D, TK1, KCNF1, TRIP12, BMP4, MPL, SLC6A12, HCFC2, SIRT4, VTCN1, SLC24A1, CTDSPL, HIP1, NFS1, GABRG2, NEUROD4, HNRNPAB, DNMT3L, ERN1, SLC18A1, LYRM1, GRIN2A, KNOP1, PINLYP, FAM189B, FECH, C19orf53, FAM65A, PRELID3A, CLCA2, PPP1CA, ARL6IP1, CSF3R, FAM205BP, AGAP1, CD207, FGF4, HEY2, DNAJA3, MARCKSL1, PPP1R16B, BIK, C9, APAF1, GP9, PDE4A, ENO1, NEMF, CAMKV, KCND3, PLA2G16, HIF1AN, CYP1A2, ARHGEF2, CLDN8, MMP28, ZNF24, PRKCQ, TRPV6, PIK3R2, KCNK5, BCKDHB, ZNF83, NPAS2, IL1A, GPR135, C21orf59, SH2B3, NT5C2, TOP2A, CCDC33, EIF3H, FAM234B, SHH, PHC3, KCMF1, TRPM2, HDAC2, PRR14, PDK1, ANK1, UBE2V2, CHAT, CDKN2A, ARTN, CLDN6, POLE, ARMC4, RIC3, OSER1, NPY5R, PEX12, ARMC9, SMARCC2, CXCR6, RFC2, PITX3, PDZRN3, COL13A1, ZNHIT2, PLA2G12A, TRMT1L, ERG, MYO1F, CRTAM, HOXA4, KLRB1, PAFAH1B2, SLC2A11, OVOL2, OXR1, CEBPD, ISCU, RFC4, SASH1, CDC42EP1, P4HTM, TNMD, TNF, KRT75, STK26, EOGT, KDM4C, LRIF1, MFHAS1, MAML3, RXRA, GCNT4, CAST, SMARCC1, POGZ, TGFBR1, KIAA1324, IGF2R, RBP3, EML2, FLT1, TMEM143, CAPN2, TSC22D3, SLC35D2, KLHL25, PJA1, SLC17A4, FBXL15, HLA-DRA, TFAP2B, ADIPOR1, INHBC, PAX5, RNF19A, TPD52L2, KIAA0355, SOS2, GTDC1, RTN4, PDK3, SSB, C8G, PDE12, ABCB8, ECM1, FAM65B, ABCA7, LRRK1, FAM76A, CD244, HTR2C, GABBR1, UPK3B, TLR6, PFDN2, SF3A1, KCNV2, VAMP4, NPFF, KRT83, PP14571, ZXDB, CCNC, CST6, GFRA3, PACS2, RANGAP1, DHX8, MMD, FABP3, SIGLEC15, DUSP13, PIGF, ACVR2B, ZFYVE9, CACNA1B, TMEM109, ZW10, SIRT6, ADAMTS8, HSD3B2, LSS, ILF3, S100B, STEAP1, TACSTD2, ADRA1D, F2, ATP8A1, SLC6A9, ELAC1, QSER1, MEIS2, CFL1, NRIP1, LY6G6C, TOMM22, TPP1, MYO1D, LARGE1, MECP2, FBLN2, WNT4, SLC12A5, TSEN34, RASAL2, SP4, RAB11FIP3, POLH, NACAD, NUP214, TRAF3IP3, MRPL58, GAL3ST1, SLC6A7, KCNC1, BCL6, CLDND1, GDE1, ATP2A1, DENND4C, KCNK7, CCNE1, NIPAL2, NUMA1, LRRC23, LRRC31, NF2, NEO1, FMO5, ALG6, MBIP, SMURF1, ABCB11, TREM2, DPP6, ZCCHC11, NTF3, SH3BP1, TSPAN15, ZNF197, PRDM1, SRD5A1, MCCC1, SEMA3A, MTIF2, BAZ2B, SMAD1, INO80D, SIX3, TULP3, AP2A2, CD226, KDM8, CAMSAP1, MTRF1L, TRIM68, TBKBP1, CREM, GAS1, BSPRY, MTSS1, ADAMTS12, TINF2, BICD1, CCDC121, CHRM5, GTF3A, CCDC106, PROC, LUZP2, ATP6V0C, STX4, CSTA, ABCD4, PPIF, FLOT1, MIPEP, ADD1, OSBP2, MBD5, TLE1, APOA4, HTR7, SLC17A9, RIDA, NSDHL, AVL9, CNTN6, KNG1, CILP, H2AFY, CD44, HAO2, LAMA2, STK3, HIST3H3, NIN, EZH1, ADAMTSL4, PCDH7, NDN, RPRM, IL23A, MAP4K4, PGGHG, ST6GAL1, RBMXL2, MECOM, CCDC70, TTC39A, IFI44L, MOCS1, ZNF32, MCM4, PCYOX1, GNA14, EDDM3B, ABI2, LILRB5, CNKSR1, EPM2A, KLHL29, DNAJC28, SLC17A5, FDPS, SMR3B, WRAP73, SDS, MIEF1, ZCCHC24, BZW1, GRB7, CBR3, ATP12A, NR1I2, MAX, MYF5, CDX4, MIA2, LXN, CUX2, PTPRO, FMOD, HAND1, CDC42SE1, EFNA1, NEUROG3, CETN1, TGM5, RARS2, MAP3K12, PPM1G, FAF2, LSM3, TRAM2, TBXAS1, MSRB2, DLGAP1, ARR3, KLHL1, RBM26, WBSCR22, TRMT44, BPNT1, PRB3, RCN3, HAMP, PSD, AVPR1A, ANKRD17, MTA1, GKN1, LUC7L3, GALK1, NAP1L1, GBE1, KIAA0040, GDF11, ELAVL2, PHF20L1, REPS1, EXTL2, SHBG, HAAO, HHEX, ZBTB43, SLC11A1, ING3, MOB4, GON4L, LOC441204, UXT, SMCHD1, UBR4, DUS4L, SOCS7, ST3GAL2, CDS1, B3GAT3, CDRT1, METTL21B, VEZT, TMPRSS11E, OTUD7B, ACOT13, TNP2, ULBP1, LPO, DPF3, XK, NLGN1, IPCEF1, TBC1D30, KDM3A, GOLGA3, SLC5A5, DCN, TAF1B, GPC1, RAB8A, EXOSC2, TRAPPC8, MVD, KIF18B, STXBP2, CD163, RASL11B, ATF7, DLX5, NSUN5, IFNG, SMOX, USP33, MADCAM1, EN1, KDM1A, EMCN, TTF2, CYP19A1, ITIH4, CRYGB, GNG12, EPM2AIP1, LRP5, SNCA, IGF1R, GP5, MYL10, IKBKB, SEC14L3, PTRF, DNAJC7, PHKA2, GLI2, MRPS16, POLD3, GRWD1, KIN, SPP2, SFSWAP, NPL, GUCY1A2, CD1B, TNS2, CDHR2, PLSCR1, TMEM39A, PEX19, SPPL2B, TRIOBP, KCNC3, RBBP8, PLK4, SEC14L1, PRDX6, UBE2I, ARHGEF12, GALR2, TMEM246, UEVLD, TLDC1, FAM134B, DNPH1, CRISP1, AADAC, GCDH, SSFA2, HOXB13, RUFY2, SF3B3, OAS1, RAP1GAP2, OAS2, LHX2, BPIFA1, FOXE1, ALX1, F13B, GPATCH2L, MDK, CSRNP2, EFHC2, UBXN1, ABL2, SLC25A13, RPL9, PMS2P1, BIN1, CA1, GJB5, EPCAM, IRS2, GPN1, FSCN3, POLR2F, ZBTB11, CLSTN3, TRAF2, CXCL8, HEXB, R3HDM4, TBC1D13, CBLN1, SERTAD3, CHRNA9, PRLR, STAP2, SLC22A8, OR3A1, LAD1, SPACA1, HTR1D, MATN4, RFX2, RAB3GAP2, AMHR2, MEP1B, PMEL, TMEM40, LGALS13, MRC1, CALCOCO1, FAXDC2, VGLL1, FAM168B, AHCYL2, ZNF385D, TAF7L, ALKBH1, C1R, GLTSCR1, CORO2A, PLPP3, NFIB, LMF2, SLC6A4, CDH4, MAP7D3, ESRRB, KIAA0141, PDCD5, BCAT2, ZNF665, NOLC1, DNAJA4, PRPS2, GNRHR, GBP2, CXADR, CDH6, CA5BP1, AGER, SF3B5, VILL, DNASE1L2, CYP2U1, CDX1, S100A12, SMTN, ACTR3B, FABP6, RGS3, GPR132, UNC5C, TREML2, KCNB1, TNIP3, FAM120A, KRT7, ICAM1, OR1F1, PIK3CG, VPS28, HSDL2, MAP2K2, SIRPG, LLPH, DNAJB5, DDA1, NRBP1, CEP55, LDOC1, FLT3, NDUFS4, RCAN2, CXorf36, MSH2, ATP9B, M6PR, SZT2, IL6R, GNG5, TSSC4, FAM49A, CHST3, ZNF107, IPO13, BACH1, TRIM45, GTF2A1, ZBTB39, SORT1, PIEZO2, WISP1, ISL1, C16orf62, ARHGAP12, MFSD1, PLIN2, CEACAM7, ADGRL2, ROM1, DFNA5, MPPED1, RMND5A, DRG2, NXT2, ADGRG1, FYN, CABIN1, TAS2R8, CD209, RMND5B, C12orf4, ENOSF1, ELK4, SCN9A, CYP4F11, SHTN1, BAZ1A, HSD17B6, CHRND, ANGPTL3, ITIH1, NFKBIL1, BEGAIN, IMPDH2, PUS7L, GNAO1, SCAPER, RNF216, RPS23, LRRC42, LRPPRC, IGFBP3, CERS6, CARHSP1, EHD3, RFX4, LRRC8E, YTHDC1, PDE1C, PSG4, PLA2G15, LHFPL2, CCDC53, RPAP1, MYH1, UBXN8, TANC2, GULP1, KMT2B, DIEXF, P2RX2, MTHFD2L, MYO1A, LRP3, IRF3, SLC22A18, FZD1, CAPRIN2, ADH1A, DSC3, SNRNP40, CNNM1, SCAF8, KAT2B, MX2, CAP2, NFYB, RERE, GNB3, EDRF1, CASP3, DAGLA, HOXB2, THOC7, NUCKS1, HMGXB4, BCAP31, RIT1, SCN10A, RASSF9, ZNF407, GPR3, PBRM1, PPY, OPCML, ZFPL1, KIAA0513, MCM2, RNF126P1, GZMK, HELZ, FEZF2, CDC42EP4, TGM1, PRKCG, KLK14, PIWIL1, NIPBL, ANGPTL7, MTTP, PTCH2, TIMM44, IL17A, PCSK1N, UMOD, LY86, PNPLA4, ZAK, GCLM, RXRB, HIST3H2A, LRRC75B, SLC1A4, S100A8, PGR, TXN, UPF2, EFNB2, C14orf2, GHSR, RAP2B, BABAM1, CAMK2B, PPP1R1A, ITGAL, ST6GALNAC2, RFX1, SLC25A23, ITGBL1, TRAPPC10, ANKRD55, ULK4, HDC, KRTAP5-8, ZFAND5, GNA13, IFNA10, DUSP7, DYNC2LI1, ZNF45, CDKL1, SLC8A2, APOF, SCN2B, SRF, AAAS, DCLK1, SERPING1, ESRRG, FGD6, DNTT, GABRA1, COQ2, SCIN, CMA1, PRICKLE3, GPR32, TLE2, KL, CGGBP1, PIK3R1, HLA-DPB1, JAM2, ZNF668, CCDC28A, DAPK2, KLF2, AVPI1, RAVER2, UPF3B, GPKOW, OVGP1, MAS1, TEAD3, LOC441601, FGD2, ZNF692, HTATIP2, GSTO1, NEIL3, MAGEA4, CSN2, NFKBIE, TBX19, POLR1E, ITGA5, DNAJA1, FXR2, TRAPPC11, ANKRD2, ANXA6, GFRA4, PSTPIP1, RGS17, EFCAB2, RB1, OSBPL3, TSFM, MTSS1L, TAS2R3, IST1, TONSL, LAMP5, LIN28A, S100G, RAB7A, CD101, PHEX, PADI4, EPHA3, TCF12, METTL9, SMAD7, ECT2, TIAM1, CHST10, PON3, C1orf115, TAAR5, PKI55, CELF2, CHST1, XRCC4, STAT3, PDGFC, BTG4, TSPOAP1, ADGRG3, GPR137B, NPC1, VHL, DEF6, CEP68, CLC, CBR4, PHLDA2, LAMB2, KIAA0087, MSX2, STK17A, CALCA, ALG13, ZER1, WDR59, IKZF4, PTCH1, NRDE2, ZMAT4, OR2W1, NR4A1, VWA5A, DAPK1, SLC25A24, SYK, ETV5, RBFOX2, CDO1, PRKG2, TBC1D19, CDC14A, PRKACG, ARSF, DDX28, TERF2, PDE6B, SDHAF3, SLC22A4, NTN3, ZNF365, TFEB, AGPAT4, PTPN2 |

**Supplementary Table 5** Results of differential gene expression analysis of genes shared by GWAS and DEGs

| Gene.symbol | ID | adj.P.Val | P.Value | t | B | logFC |
| --- | --- | --- | --- | --- | --- | --- |
| DNAJB9 | 202842_s_at | 0.0114 | 2.76E-05 | 6.130639 | 2.788706 | 1.8703983 |
| VPS53 | 219794_at | 0.01322 | 0.000145 | -5.183862 | 1.240722 | -0.8617662 |
| SCGN | 205697_at | 0.01368 | 0.000174 | 5.082976 | 1.06767 | 2.1976062 |
| CMAS | 218111_s_at | 0.01598 | 0.000534 | 4.481797 | 0.007639 | 1.4435364 |
| RGS6 | 210270_at | 0.02023 | 0.00133 | -4.008533 | -0.855813 | -0.6676233 |
| FASLG | 211333_s_at | 0.02229 | 0.00178 | -3.858207 | -1.133864 | -0.4972055 |
| ABHD10 | 218633_x_at | 0.0273 | 0.00331 | 3.542316 | -1.72144 | 0.8123664 |
| ATRN | 211852_s_at | 0.02763 | 0.00342 | 3.525479 | -1.752819 | 0.9160507 |
| PLA2G2F | 221416_at | 0.02875 | 0.00376 | -3.477494 | -1.842254 | -0.5509669 |
| ITIH2 | 204987_at | 0.03101 | 0.00461 | -3.374641 | -2.033894 | -0.590874 |
| ROBO1 | 213194_at | 0.03337 | 0.00554 | 3.281862 | -2.206563 | 1.6319826 |
| SGCG | 207302_at | 0.03642 | 0.00661 | -3.192778 | -2.372028 | -0.4223329 |
| SH3GL2 | 205751_at | 0.03742 | 0.00707 | 3.1589 | -2.434839 | 1.6941395 |
| CNR1 | 207940_x_at | 0.04002 | 0.00835 | -3.074463 | -2.591039 | -0.3313695 |
| FOXN3 | 205021_s_at | 0.04374 | 0.0102 | -2.973994 | -2.776097 | -0.7551398 |

**Supplementary Table 6** DisGeNET enrichment results of core genes shared by NAFLD and T2D

| GO | Description | Count | % | Hits | Log10(P) | Log10(q) |
| --- | --- | --- | --- | --- | --- | --- |
| C0007138 | Carcinoma, Transitional Cell | 4 | 27 | FASLG|CNR1|SH3GL2|CMAS | -3.7 | 0 |
| C0013264 | Muscular Dystrophy, Duchenne | 3 | 20 | CNR1|SGCG|RGS6 | -3.1 | 0 |
| C0220597 | Adult Hodgkin Lymphoma | 3 | 20 | FOXN3|CNR1|RGS6 | -3 | 0 |
| C0220644 | Childhood Hodgkin Lymphoma | 3 | 20 | FOXN3|CNR1|RGS6 | -3 | 0 |
| C2145472 | Urothelial Carcinoma | 3 | 20 | CNR1|SH3GL2|CMAS | -2.9 | 0 |
| C0595989 | Carcinoma of larynx | 3 | 20 | FOXN3|SH3GL2|CMAS | -2.9 | 0 |
| C0021368 | Inflammation | 3 | 20 | FASLG|CNR1|ITIH2 | -2.8 | 0 |
| C0002726 | Amyloidosis | 3 | 20 | DNAJB9|SGCG|SH3GL2 | -2.3 | 0 |

**Supplementary Table 7** GO enrichment results of core genes shared by NAFLD and T2D

| ONTOLOGY | ID | Description | pvalue | p.adjust | qvalue | geneID | Count[[2]](#footnote-1) |
| --- | --- | --- | --- | --- | --- | --- | --- |
| Biological process | GO:0008038 | neuron recognition | 0.000649129 | 0.136240759 | 0.094263332 | ROBO1/CNR1 | 2 |
| Biological process | GO:0006919 | activation of cysteine-type endopeptidase activity involved in apoptotic process | 0.0016616 | 0.136240759 | 0.094263332 | FASLG/ROBO1 | 2 |
| Biological process | GO:0052548 | regulation of endopeptidase activity | 0.004282288 | 0.136240759 | 0.094263332 | FASLG/ITIH2/ROBO1 | 3 |
| Biological process | GO:0043280 | positive regulation of cysteine-type endopeptidase activity involved in apoptotic process | 0.004305368 | 0.136240759 | 0.094263332 | FASLG/ROBO1 | 2 |
| Biological process | GO:0052547 | regulation of peptidase activity | 0.005206559 | 0.136240759 | 0.094263332 | FASLG/ITIH2/ROBO1 | 3 |
| Biological process | GO:2001056 | positive regulation of cysteine-type endopeptidase activity | 0.00559405 | 0.136240759 | 0.094263332 | FASLG/ROBO1 | 2 |
| Biological process | GO:0050806 | positive regulation of synaptic transmission | 0.007817631 | 0.136240759 | 0.094263332 | SCGN/CNR1 | 2 |
| Biological process | GO:0002084 | protein depalmitoylation | 0.007908849 | 0.136240759 | 0.094263332 | ABHD10 | 1 |
| Biological process | GO:0003129 | heart induction | 0.007908849 | 0.136240759 | 0.094263332 | ROBO1 | 1 |
| Biological process | GO:0038171 | cannabinoid signaling pathway | 0.007908849 | 0.136240759 | 0.094263332 | CNR1 | 1 |
| Biological process | GO:0070099 | regulation of chemokine-mediated signaling pathway | 0.007908849 | 0.136240759 | 0.094263332 | ROBO1 | 1 |
| Biological process | GO:0010950 | positive regulation of endopeptidase activity | 0.008175996 | 0.136240759 | 0.094263332 | FASLG/ROBO1 | 2 |
| Biological process | GO:0001660 | fever generation | 0.008696514 | 0.136240759 | 0.094263332 | CNR1 | 1 |
| Biological process | GO:0006054 | N-acetylneuraminate metabolic process | 0.008696514 | 0.136240759 | 0.094263332 | CMAS | 1 |
| Biological process | GO:0021889 | olfactory bulb interneuron differentiation | 0.008696514 | 0.136240759 | 0.094263332 | ROBO1 | 1 |
| Biological process | GO:0030320 | cellular monovalent inorganic anion homeostasis | 0.008696514 | 0.136240759 | 0.094263332 | FASLG | 1 |
| Biological process | GO:0030644 | cellular chloride ion homeostasis | 0.008696514 | 0.136240759 | 0.094263332 | FASLG | 1 |
| Biological process | GO:0036152 | phosphatidylethanolamine acyl-chain remodeling | 0.008696514 | 0.136240759 | 0.094263332 | PLA2G2F | 1 |
| Biological process | GO:0043084 | penile erection | 0.008696514 | 0.136240759 | 0.094263332 | CNR1 | 1 |
| Biological process | GO:2000826 | regulation of heart morphogenesis | 0.008696514 | 0.136240759 | 0.094263332 | ROBO1 | 1 |
| Biological process | GO:0031650 | regulation of heat generation | 0.009483596 | 0.136240759 | 0.094263332 | CNR1 | 1 |
| Biological process | GO:0051001 | negative regulation of nitric-oxide synthase activity | 0.009483596 | 0.136240759 | 0.094263332 | CNR1 | 1 |
| Biological process | GO:0099509 | regulation of presynaptic cytosolic calcium ion concentration | 0.009483596 | 0.136240759 | 0.094263332 | SCGN | 1 |
| Biological process | GO:0099504 | synaptic vesicle cycle | 0.00987886 | 0.136240759 | 0.094263332 | SH3GL2/CNR1 | 2 |
| Biological process | GO:0010952 | positive regulation of peptidase activity | 0.010077071 | 0.136240759 | 0.094263332 | FASLG/ROBO1 | 2 |
| Biological process | GO:0002863 | positive regulation of inflammatory response to antigenic stimulus | 0.010270095 | 0.136240759 | 0.094263332 | CNR1 | 1 |
| Biological process | GO:0033604 | negative regulation of catecholamine secretion | 0.010270095 | 0.136240759 | 0.094263332 | CNR1 | 1 |
| Biological process | GO:0099550 | trans-synaptic signaling, modulating synaptic transmission | 0.010270095 | 0.136240759 | 0.094263332 | CNR1 | 1 |
| Biological process | GO:0002695 | negative regulation of leukocyte activation | 0.010478833 | 0.136240759 | 0.094263332 | PLA2G2F/CNR1 | 2 |
| Biological process | GO:0033004 | negative regulation of mast cell activation | 0.011056011 | 0.136240759 | 0.094263332 | CNR1 | 1 |
| Biological process | GO:0036151 | phosphatidylcholine acyl-chain remodeling | 0.011056011 | 0.136240759 | 0.094263332 | PLA2G2F | 1 |
| Biological process | GO:0048388 | endosomal lumen acidification | 0.011056011 | 0.136240759 | 0.094263332 | FASLG | 1 |
| Biological process | GO:0098734 | macromolecule depalmitoylation | 0.011056011 | 0.136240759 | 0.094263332 | ABHD10 | 1 |
| Biological process | GO:0099171 | presynaptic modulation of chemical synaptic transmission | 0.011056011 | 0.136240759 | 0.094263332 | CNR1 | 1 |
| Biological process | GO:1903894 | regulation of IRE1-mediated unfolded protein response | 0.011056011 | 0.136240759 | 0.094263332 | DNAJB9 | 1 |
| Biological process | GO:2001140 | positive regulation of phospholipid transport | 0.011056011 | 0.136240759 | 0.094263332 | FASLG | 1 |
| Biological process | GO:0043281 | regulation of cysteine-type endopeptidase activity involved in apoptotic process | 0.011198929 | 0.136240759 | 0.094263332 | FASLG/ROBO1 | 2 |
| Biological process | GO:0002864 | regulation of acute inflammatory response to antigenic stimulus | 0.011841344 | 0.136240759 | 0.094263332 | CNR1 | 1 |
| Biological process | GO:0033599 | regulation of mammary gland epithelial cell proliferation | 0.011841344 | 0.136240759 | 0.094263332 | ROBO1 | 1 |
| Biological process | GO:0046322 | negative regulation of fatty acid oxidation | 0.011841344 | 0.136240759 | 0.094263332 | CNR1 | 1 |
| Biological process | GO:1900102 | negative regulation of endoplasmic reticulum unfolded protein response | 0.011841344 | 0.136240759 | 0.094263332 | DNAJB9 | 1 |
| Biological process | GO:2001138 | regulation of phospholipid transport | 0.011841344 | 0.136240759 | 0.094263332 | FASLG | 1 |
| Biological process | GO:0099003 | vesicle-mediated transport in synapse | 0.011940485 | 0.136240759 | 0.094263332 | SH3GL2/CNR1 | 2 |
| Biological process | GO:0042159 | lipoprotein catabolic process | 0.012626095 | 0.136240759 | 0.094263332 | ABHD10 | 1 |
| Biological process | GO:0050866 | negative regulation of cell activation | 0.012925081 | 0.136240759 | 0.094263332 | PLA2G2F/CNR1 | 2 |
| Biological process | GO:0003184 | pulmonary valve morphogenesis | 0.013410265 | 0.136240759 | 0.094263332 | ROBO1 | 1 |
| Biological process | GO:0031649 | heat generation | 0.013410265 | 0.136240759 | 0.094263332 | CNR1 | 1 |
| Biological process | GO:1903729 | regulation of plasma membrane organization | 0.013410265 | 0.136240759 | 0.094263332 | FASLG | 1 |
| Biological process | GO:0008037 | cell recognition | 0.013944297 | 0.136240759 | 0.094263332 | ROBO1/CNR1 | 2 |
| Biological process | GO:0001956 | positive regulation of neurotransmitter secretion | 0.014193853 | 0.136240759 | 0.094263332 | CNR1 | 1 |
| Biological process | GO:0003128 | heart field specification | 0.014193853 | 0.136240759 | 0.094263332 | ROBO1 | 1 |
| Biological process | GO:0032769 | negative regulation of monooxygenase activity | 0.014193853 | 0.136240759 | 0.094263332 | CNR1 | 1 |
| Biological process | GO:0043116 | negative regulation of vascular permeability | 0.014193853 | 0.136240759 | 0.094263332 | SH3GL2 | 1 |
| Biological process | GO:0097094 | craniofacial suture morphogenesis | 0.014193853 | 0.136240759 | 0.094263332 | FOXN3 | 1 |
| Biological process | GO:2000116 | regulation of cysteine-type endopeptidase activity | 0.014525313 | 0.136240759 | 0.094263332 | FASLG/ROBO1 | 2 |
| Biological process | GO:0006837 | serotonin transport | 0.01497686 | 0.136240759 | 0.094263332 | CNR1 | 1 |
| Biological process | GO:0030002 | cellular anion homeostasis | 0.015759287 | 0.136240759 | 0.094263332 | FASLG | 1 |
| Biological process | GO:0031998 | regulation of fatty acid beta-oxidation | 0.015759287 | 0.136240759 | 0.094263332 | CNR1 | 1 |
| Biological process | GO:0036498 | IRE1-mediated unfolded protein response | 0.015759287 | 0.136240759 | 0.094263332 | DNAJB9 | 1 |
| Biological process | GO:0055064 | chloride ion homeostasis | 0.015759287 | 0.136240759 | 0.094263332 | FASLG | 1 |
| Biological process | GO:0055083 | monovalent inorganic anion homeostasis | 0.015759287 | 0.136240759 | 0.094263332 | FASLG | 1 |
| Biological process | GO:0003177 | pulmonary valve development | 0.016541134 | 0.136240759 | 0.094263332 | ROBO1 | 1 |
| Biological process | GO:0007413 | axonal fasciculation | 0.016541134 | 0.136240759 | 0.094263332 | CNR1 | 1 |
| Biological process | GO:0016137 | glycoside metabolic process | 0.016541134 | 0.136240759 | 0.094263332 | ABHD10 | 1 |
| Biological process | GO:0052695 | cellular glucuronidation | 0.016541134 | 0.136240759 | 0.094263332 | ABHD10 | 1 |
| Biological process | GO:0106030 | neuron projection fasciculation | 0.016541134 | 0.136240759 | 0.094263332 | CNR1 | 1 |
| Biological process | GO:0007620 | copulation | 0.017322401 | 0.136240759 | 0.094263332 | CNR1 | 1 |
| Biological process | GO:0001759 | organ induction | 0.018883197 | 0.136240759 | 0.094263332 | ROBO1 | 1 |
| Biological process | GO:0006925 | inflammatory cell apoptotic process | 0.018883197 | 0.136240759 | 0.094263332 | FASLG | 1 |
| Biological process | GO:0010623 | programmed cell death involved in cell development | 0.018883197 | 0.136240759 | 0.094263332 | FASLG | 1 |
| Biological process | GO:0051590 | positive regulation of neurotransmitter transport | 0.018883197 | 0.136240759 | 0.094263332 | CNR1 | 1 |
| Biological process | GO:2000353 | positive regulation of endothelial cell apoptotic process | 0.018883197 | 0.136240759 | 0.094263332 | FASLG | 1 |
| Biological process | GO:0003148 | outflow tract septum morphogenesis | 0.019662727 | 0.136240759 | 0.094263332 | ROBO1 | 1 |
| Biological process | GO:0006658 | phosphatidylserine metabolic process | 0.019662727 | 0.136240759 | 0.094263332 | PLA2G2F | 1 |
| Biological process | GO:0017121 | plasma membrane phospholipid scrambling | 0.019662727 | 0.136240759 | 0.094263332 | FASLG | 1 |
| Biological process | GO:0002438 | acute inflammatory response to antigenic stimulus | 0.020441679 | 0.136240759 | 0.094263332 | CNR1 | 1 |
| Biological process | GO:0006063 | uronic acid metabolic process | 0.020441679 | 0.136240759 | 0.094263332 | ABHD10 | 1 |
| Biological process | GO:0006929 | substrate-dependent cell migration | 0.020441679 | 0.136240759 | 0.094263332 | ROBO1 | 1 |
| Biological process | GO:0019585 | glucuronate metabolic process | 0.020441679 | 0.136240759 | 0.094263332 | ABHD10 | 1 |
| Biological process | GO:0033598 | mammary gland epithelial cell proliferation | 0.020441679 | 0.136240759 | 0.094263332 | ROBO1 | 1 |
| Biological process | GO:0050995 | negative regulation of lipid catabolic process | 0.020441679 | 0.136240759 | 0.094263332 | CNR1 | 1 |
| Biological process | GO:0006874 | cellular calcium ion homeostasis | 0.020760373 | 0.136240759 | 0.094263332 | SCGN/FASLG | 2 |
| Biological process | GO:0051953 | negative regulation of amine transport | 0.021220054 | 0.136240759 | 0.094263332 | CNR1 | 1 |
| Biological process | GO:0060259 | regulation of feeding behavior | 0.021220054 | 0.136240759 | 0.094263332 | CNR1 | 1 |
| Biological process | GO:1900746 | regulation of vascular endothelial growth factor signaling pathway | 0.021220054 | 0.136240759 | 0.094263332 | ROBO1 | 1 |
| Biological process | GO:0002675 | positive regulation of acute inflammatory response | 0.021997851 | 0.136240759 | 0.094263332 | CNR1 | 1 |
| Biological process | GO:0003272 | endocardial cushion formation | 0.021997851 | 0.136240759 | 0.094263332 | ROBO1 | 1 |
| Biological process | GO:0014072 | response to isoquinoline alkaloid | 0.021997851 | 0.136240759 | 0.094263332 | CNR1 | 1 |
| Biological process | GO:0043278 | response to morphine | 0.021997851 | 0.136240759 | 0.094263332 | CNR1 | 1 |
| Biological process | GO:0003156 | regulation of animal organ formation | 0.022775071 | 0.136240759 | 0.094263332 | ROBO1 | 1 |
| Biological process | GO:1902547 | regulation of cellular response to vascular endothelial growth factor stimulus | 0.022775071 | 0.136240759 | 0.094263332 | ROBO1 | 1 |
| Biological process | GO:1900101 | regulation of endoplasmic reticulum unfolded protein response | 0.023551715 | 0.136240759 | 0.094263332 | DNAJB9 | 1 |
| Biological process | GO:0055074 | calcium ion homeostasis | 0.024304611 | 0.136240759 | 0.094263332 | SCGN/FASLG | 2 |
| Biological process | GO:0003180 | aortic valve morphogenesis | 0.025103275 | 0.136240759 | 0.094263332 | ROBO1 | 1 |
| Biological process | GO:0050482 | arachidonic acid secretion | 0.025103275 | 0.136240759 | 0.094263332 | PLA2G2F | 1 |
| Biological process | GO:1903963 | arachidonate transport | 0.025103275 | 0.136240759 | 0.094263332 | PLA2G2F | 1 |
| Biological process | GO:0021772 | olfactory bulb development | 0.025878191 | 0.136240759 | 0.094263332 | ROBO1 | 1 |
| Biological process | GO:0072503 | cellular divalent inorganic cation homeostasis | 0.02639165 | 0.136240759 | 0.094263332 | SCGN/FASLG | 2 |
| Biological process | GO:0007095 | mitotic G2 DNA damage checkpoint signaling | 0.026652533 | 0.136240759 | 0.094263332 | FOXN3 | 1 |
| Biological process | GO:0035025 | positive regulation of Rho protein signal transduction | 0.026652533 | 0.136240759 | 0.094263332 | ROBO1 | 1 |
| Biological process | GO:0110110 | positive regulation of animal organ morphogenesis | 0.026652533 | 0.136240759 | 0.094263332 | ROBO1 | 1 |
| Biological process | GO:0021988 | olfactory lobe development | 0.0274263 | 0.136240759 | 0.094263332 | ROBO1 | 1 |
| Biological process | GO:0030212 | hyaluronan metabolic process | 0.0274263 | 0.136240759 | 0.094263332 | ITIH2 | 1 |
| Biological process | GO:0031128 | developmental induction | 0.0274263 | 0.136240759 | 0.094263332 | ROBO1 | 1 |
| Biological process | GO:0035633 | maintenance of blood-brain barrier | 0.0274263 | 0.136240759 | 0.094263332 | SH3GL2 | 1 |
| Biological process | GO:0051354 | negative regulation of oxidoreductase activity | 0.0274263 | 0.136240759 | 0.094263332 | CNR1 | 1 |
| Biological process | GO:0060914 | heart formation | 0.0274263 | 0.136240759 | 0.094263332 | ROBO1 | 1 |
| Biological process | GO:1901658 | glycosyl compound catabolic process | 0.0274263 | 0.136240759 | 0.094263332 | ABHD10 | 1 |
| Biological process | GO:0060249 | anatomical structure homeostasis | 0.027926176 | 0.136240759 | 0.094263332 | SCGN/SH3GL2 | 2 |
| Biological process | GO:0032228 | regulation of synaptic transmission, GABAergic | 0.028199493 | 0.136240759 | 0.094263332 | CNR1 | 1 |
| Biological process | GO:0045777 | positive regulation of blood pressure | 0.028199493 | 0.136240759 | 0.094263332 | CNR1 | 1 |
| Biological process | GO:0046320 | regulation of fatty acid oxidation | 0.028199493 | 0.136240759 | 0.094263332 | CNR1 | 1 |
| Biological process | GO:0046471 | phosphatidylglycerol metabolic process | 0.028199493 | 0.136240759 | 0.094263332 | PLA2G2F | 1 |
| Biological process | GO:0097484 | dendrite extension | 0.028199493 | 0.136240759 | 0.094263332 | SH3GL2 | 1 |
| Biological process | GO:1903514 | release of sequestered calcium ion into cytosol by endoplasmic reticulum | 0.028199493 | 0.136240759 | 0.094263332 | FASLG | 1 |
| Biological process | GO:0016042 | lipid catabolic process | 0.028393546 | 0.136240759 | 0.094263332 | PLA2G2F/CNR1 | 2 |
| Biological process | GO:0032496 | response to lipopolysaccharide | 0.028864123 | 0.136240759 | 0.094263332 | FASLG/CNR1 | 2 |
| Biological process | GO:0003176 | aortic valve development | 0.028972112 | 0.136240759 | 0.094263332 | ROBO1 | 1 |
| Biological process | GO:0010092 | specification of animal organ identity | 0.028972112 | 0.136240759 | 0.094263332 | ROBO1 | 1 |
| Biological process | GO:0014046 | dopamine secretion | 0.028972112 | 0.136240759 | 0.094263332 | CNR1 | 1 |
| Biological process | GO:0014059 | regulation of dopamine secretion | 0.028972112 | 0.136240759 | 0.094263332 | CNR1 | 1 |
| Biological process | GO:0003203 | endocardial cushion morphogenesis | 0.029744158 | 0.136487306 | 0.094433915 | ROBO1 | 1 |
| Biological process | GO:0007617 | mating behavior | 0.029744158 | 0.136487306 | 0.094433915 | CNR1 | 1 |
| Biological process | GO:0045742 | positive regulation of epidermal growth factor receptor signaling pathway | 0.029744158 | 0.136487306 | 0.094433915 | FASLG | 1 |
| Biological process | GO:0045922 | negative regulation of fatty acid metabolic process | 0.030515631 | 0.138308272 | 0.095693819 | CNR1 | 1 |
| Biological process | GO:0031346 | positive regulation of cell projection organization | 0.030939982 | 0.138308272 | 0.095693819 | ROBO1/CNR1 | 2 |
| Biological process | GO:1901186 | positive regulation of ERBB signaling pathway | 0.031286532 | 0.138308272 | 0.095693819 | FASLG | 1 |
| Biological process | GO:0072507 | divalent inorganic cation homeostasis | 0.031590572 | 0.138308272 | 0.095693819 | SCGN/FASLG | 2 |
| Biological process | GO:0006040 | amino sugar metabolic process | 0.03205686 | 0.138308272 | 0.095693819 | CMAS | 1 |
| Biological process | GO:0070050 | neuron cellular homeostasis | 0.03205686 | 0.138308272 | 0.095693819 | SCGN | 1 |
| Biological process | GO:1905314 | semi-lunar valve development | 0.03205686 | 0.138308272 | 0.095693819 | ROBO1 | 1 |
| Biological process | GO:0002237 | response to molecule of bacterial origin | 0.032246681 | 0.138308272 | 0.095693819 | FASLG/CNR1 | 2 |
| Biological process | GO:0050999 | regulation of nitric-oxide synthase activity | 0.032826616 | 0.138308272 | 0.095693819 | CNR1 | 1 |
| Biological process | GO:0060412 | ventricular septum morphogenesis | 0.033595801 | 0.138308272 | 0.095693819 | ROBO1 | 1 |
| Biological process | GO:1904037 | positive regulation of epithelial cell apoptotic process | 0.033595801 | 0.138308272 | 0.095693819 | FASLG | 1 |
| Biological process | GO:0045862 | positive regulation of proteolysis | 0.034247758 | 0.138308272 | 0.095693819 | FASLG/ROBO1 | 2 |
| Biological process | GO:0044275 | cellular carbohydrate catabolic process | 0.034364416 | 0.138308272 | 0.095693819 | ABHD10 | 1 |
| Biological process | GO:1903573 | negative regulation of response to endoplasmic reticulum stress | 0.034364416 | 0.138308272 | 0.095693819 | DNAJB9 | 1 |
| Biological process | GO:0019098 | reproductive behavior | 0.035132459 | 0.138308272 | 0.095693819 | CNR1 | 1 |
| Biological process | GO:0033003 | regulation of mast cell activation | 0.035132459 | 0.138308272 | 0.095693819 | CNR1 | 1 |
| Biological process | GO:0021795 | cerebral cortex cell migration | 0.035899933 | 0.138308272 | 0.095693819 | ROBO1 | 1 |
| Biological process | GO:0070266 | necroptotic process | 0.035899933 | 0.138308272 | 0.095693819 | FASLG | 1 |
| Biological process | GO:1900271 | regulation of long-term synaptic potentiation | 0.035899933 | 0.138308272 | 0.095693819 | SCGN | 1 |
| Biological process | GO:1990090 | cellular response to nerve growth factor stimulus | 0.035899933 | 0.138308272 | 0.095693819 | SH3GL2 | 1 |
| Biological process | GO:0002861 | regulation of inflammatory response to antigenic stimulus | 0.036666836 | 0.138308272 | 0.095693819 | CNR1 | 1 |
| Biological process | GO:0050919 | negative chemotaxis | 0.036666836 | 0.138308272 | 0.095693819 | ROBO1 | 1 |
| Biological process | GO:0002673 | regulation of acute inflammatory response | 0.03743317 | 0.138308272 | 0.095693819 | CNR1 | 1 |
| Biological process | GO:0003197 | endocardial cushion development | 0.03743317 | 0.138308272 | 0.095693819 | ROBO1 | 1 |
| Biological process | GO:0006953 | acute-phase response | 0.03743317 | 0.138308272 | 0.095693819 | CNR1 | 1 |
| Biological process | GO:0032309 | icosanoid secretion | 0.03743317 | 0.138308272 | 0.095693819 | PLA2G2F | 1 |
| Biological process | GO:0042220 | response to cocaine | 0.03743317 | 0.138308272 | 0.095693819 | CNR1 | 1 |
| Biological process | GO:0045747 | positive regulation of Notch signaling pathway | 0.03743317 | 0.138308272 | 0.095693819 | ROBO1 | 1 |
| Biological process | GO:0045776 | negative regulation of blood pressure | 0.03743317 | 0.138308272 | 0.095693819 | CNR1 | 1 |
| Biological process | GO:1990089 | response to nerve growth factor | 0.03743317 | 0.138308272 | 0.095693819 | SH3GL2 | 1 |
| Biological process | GO:0035094 | response to nicotine | 0.038198935 | 0.138440728 | 0.095785463 | CNR1 | 1 |
| Biological process | GO:0043114 | regulation of vascular permeability | 0.038198935 | 0.138440728 | 0.095785463 | SH3GL2 | 1 |
| Biological process | GO:0055081 | anion homeostasis | 0.038198935 | 0.138440728 | 0.095785463 | FASLG | 1 |
| Biological process | GO:0038084 | vascular endothelial growth factor signaling pathway | 0.038964132 | 0.139061374 | 0.096214881 | ROBO1 | 1 |
| Biological process | GO:0044818 | mitotic G2/M transition checkpoint | 0.038964132 | 0.139061374 | 0.096214881 | FOXN3 | 1 |
| Biological process | GO:0006631 | fatty acid metabolic process | 0.039103374 | 0.139061374 | 0.096214881 | PLA2G2F/CNR1 | 2 |
| Biological process | GO:0015872 | dopamine transport | 0.03972876 | 0.13954114 | 0.096546825 | CNR1 | 1 |
| Biological process | GO:1903307 | positive regulation of regulated secretory pathway | 0.03972876 | 0.13954114 | 0.096546825 | CNR1 | 1 |
| Biological process | GO:0051452 | intracellular pH reduction | 0.040492821 | 0.140490336 | 0.097203562 | FASLG | 1 |
| Biological process | GO:0051480 | regulation of cytosolic calcium ion concentration | 0.040492821 | 0.140490336 | 0.097203562 | SCGN | 1 |
| Biological process | GO:0002639 | positive regulation of immunoglobulin production | 0.041256314 | 0.14056792 | 0.097257242 | DNAJB9 | 1 |
| Biological process | GO:0045332 | phospholipid translocation | 0.041256314 | 0.14056792 | 0.097257242 | FASLG | 1 |
| Biological process | GO:0097300 | programmed necrotic cell death | 0.041256314 | 0.14056792 | 0.097257242 | FASLG | 1 |
| Biological process | GO:0006875 | cellular metal ion homeostasis | 0.042726176 | 0.142355146 | 0.098493802 | SCGN/FASLG | 2 |
| Biological process | GO:0003179 | heart valve morphogenesis | 0.042781599 | 0.142355146 | 0.098493802 | ROBO1 | 1 |
| Biological process | GO:0043525 | positive regulation of neuron apoptotic process | 0.042781599 | 0.142355146 | 0.098493802 | FASLG | 1 |
| Biological process | GO:0072132 | mesenchyme morphogenesis | 0.042781599 | 0.142355146 | 0.098493802 | ROBO1 | 1 |
| Biological process | GO:0071715 | icosanoid transport | 0.043543392 | 0.142716831 | 0.098744047 | PLA2G2F | 1 |
| Biological process | GO:0032768 | regulation of monooxygenase activity | 0.04430462 | 0.142716831 | 0.098744047 | CNR1 | 1 |
| Biological process | GO:0050433 | regulation of catecholamine secretion | 0.04430462 | 0.142716831 | 0.098744047 | CNR1 | 1 |
| Biological process | GO:0051932 | synaptic transmission, GABAergic | 0.04430462 | 0.142716831 | 0.098744047 | CNR1 | 1 |
| Biological process | GO:0070231 | T cell apoptotic process | 0.04430462 | 0.142716831 | 0.098744047 | FASLG | 1 |
| Biological process | GO:0019369 | arachidonic acid metabolic process | 0.045065281 | 0.142716831 | 0.098744047 | PLA2G2F | 1 |
| Biological process | GO:0034204 | lipid translocation | 0.045065281 | 0.142716831 | 0.098744047 | FASLG | 1 |
| Biological process | GO:0050432 | catecholamine secretion | 0.045065281 | 0.142716831 | 0.098744047 | CNR1 | 1 |
| Biological process | GO:0006869 | lipid transport | 0.045147679 | 0.142716831 | 0.098744047 | FASLG/PLA2G2F | 2 |
| Biological process | GO:0022029 | telencephalon cell migration | 0.04658491 | 0.143306232 | 0.099151847 | ROBO1 | 1 |
| Biological process | GO:2000300 | regulation of synaptic vesicle exocytosis | 0.04658491 | 0.143306232 | 0.099151847 | CNR1 | 1 |
| Biological process | GO:2000351 | regulation of endothelial cell apoptotic process | 0.04658491 | 0.143306232 | 0.099151847 | FASLG | 1 |
| Biological process | GO:0060135 | maternal process involved in female pregnancy | 0.047343877 | 0.143306232 | 0.099151847 | CNR1 | 1 |
| Biological process | GO:0098900 | regulation of action potential | 0.047343877 | 0.143306232 | 0.099151847 | CNR1 | 1 |
| Biological process | GO:0010975 | regulation of neuron projection development | 0.047620343 | 0.143306232 | 0.099151847 | ROBO1/CNR1 | 2 |
| Biological process | GO:0050804 | modulation of chemical synaptic transmission | 0.048005221 | 0.143306232 | 0.099151847 | SCGN/CNR1 | 2 |
| Biological process | GO:0035904 | aorta development | 0.048102281 | 0.143306232 | 0.099151847 | ROBO1 | 1 |
| Biological process | GO:0002683 | negative regulation of immune system process | 0.048198102 | 0.143306232 | 0.099151847 | PLA2G2F/CNR1 | 2 |
| Biological process | GO:0099177 | regulation of trans-synaptic signaling | 0.048198102 | 0.143306232 | 0.099151847 | SCGN/CNR1 | 2 |
| Biological process | GO:0021885 | forebrain cell migration | 0.048860121 | 0.143306232 | 0.099151847 | ROBO1 | 1 |
| Biological process | GO:0046579 | positive regulation of Ras protein signal transduction | 0.048860121 | 0.143306232 | 0.099151847 | ROBO1 | 1 |
| Biological process | GO:0050994 | regulation of lipid catabolic process | 0.048860121 | 0.143306232 | 0.099151847 | CNR1 | 1 |
| Biological process | GO:0097035 | regulation of membrane lipid distribution | 0.048860121 | 0.143306232 | 0.099151847 | FASLG | 1 |
| Biological process | GO:0010972 | negative regulation of G2/M transition of mitotic cell cycle | 0.049617398 | 0.144720838 | 0.100130596 | FOXN3 | 1 |
| Biological process | GO:0003170 | heart valve development | 0.050374112 | 0.144720838 | 0.100130596 | ROBO1 | 1 |
| Biological process | GO:0040014 | regulation of multicellular organism growth | 0.050374112 | 0.144720838 | 0.100130596 | ATRN | 1 |
| Biological process | GO:0048488 | synaptic vesicle endocytosis | 0.051130264 | 0.144720838 | 0.100130596 | SH3GL2 | 1 |
| Biological process | GO:0072577 | endothelial cell apoptotic process | 0.051130264 | 0.144720838 | 0.100130596 | FASLG | 1 |
| Biological process | GO:0140238 | presynaptic endocytosis | 0.051130264 | 0.144720838 | 0.100130596 | SH3GL2 | 1 |
| Biological process | GO:1902750 | negative regulation of cell cycle G2/M phase transition | 0.051130264 | 0.144720838 | 0.100130596 | FOXN3 | 1 |
| Biological process | GO:0007187 | G protein-coupled receptor signaling pathway, coupled to cyclic nucleotide second messenger | 0.051885854 | 0.144720838 | 0.100130596 | CNR1 | 1 |
| Biological process | GO:0015909 | long-chain fatty acid transport | 0.051885854 | 0.144720838 | 0.100130596 | PLA2G2F | 1 |
| Biological process | GO:0045576 | mast cell activation | 0.051885854 | 0.144720838 | 0.100130596 | CNR1 | 1 |
| Biological process | GO:0048645 | animal organ formation | 0.053395349 | 0.146772724 | 0.10155027 | ROBO1 | 1 |
| Biological process | GO:0050922 | negative regulation of chemotaxis | 0.053395349 | 0.146772724 | 0.10155027 | ROBO1 | 1 |
| Biological process | GO:0061180 | mammary gland epithelium development | 0.053395349 | 0.146772724 | 0.10155027 | ROBO1 | 1 |
| Biological process | GO:0042130 | negative regulation of T cell proliferation | 0.054149256 | 0.148129454 | 0.102488976 | PLA2G2F | 1 |
| Biological process | GO:0051057 | positive regulation of small GTPase mediated signal transduction | 0.054902601 | 0.148509728 | 0.102752082 | ROBO1 | 1 |
| Biological process | GO:0010876 | lipid localization | 0.054927846 | 0.148509728 | 0.102752082 | FASLG/PLA2G2F | 2 |
| Biological process | GO:0060411 | cardiac septum morphogenesis | 0.055655387 | 0.148509728 | 0.102752082 | ROBO1 | 1 |
| Biological process | GO:0070265 | necrotic cell death | 0.055655387 | 0.148509728 | 0.102752082 | FASLG | 1 |
| Biological process | GO:0061564 | axon development | 0.05635379 | 0.148509728 | 0.102752082 | ROBO1/CNR1 | 2 |
| Biological process | GO:0050795 | regulation of behavior | 0.056407613 | 0.148509728 | 0.102752082 | CNR1 | 1 |
| Biological process | GO:0051937 | catecholamine transport | 0.056407613 | 0.148509728 | 0.102752082 | CNR1 | 1 |
| Biological process | GO:1904888 | cranial skeletal system development | 0.056407613 | 0.148509728 | 0.102752082 | FOXN3 | 1 |
| Biological process | GO:0003281 | ventricular septum development | 0.05715928 | 0.148509728 | 0.102752082 | ROBO1 | 1 |
| Biological process | GO:0035924 | cellular response to vascular endothelial growth factor stimulus | 0.05715928 | 0.148509728 | 0.102752082 | ROBO1 | 1 |
| Biological process | GO:0042058 | regulation of epidermal growth factor receptor signaling pathway | 0.05715928 | 0.148509728 | 0.102752082 | FASLG | 1 |
| Biological process | GO:0030003 | cellular cation homeostasis | 0.05820682 | 0.150351682 | 0.104026508 | SCGN/FASLG | 2 |
| Biological process | GO:0002637 | regulation of immunoglobulin production | 0.058660937 | 0.150351682 | 0.104026508 | DNAJB9 | 1 |
| Biological process | GO:0006635 | fatty acid beta-oxidation | 0.058660937 | 0.150351682 | 0.104026508 | CNR1 | 1 |
| Biological process | GO:0030968 | endoplasmic reticulum unfolded protein response | 0.059410929 | 0.150914368 | 0.104415824 | DNAJB9 | 1 |
| Biological process | GO:0046470 | phosphatidylcholine metabolic process | 0.059410929 | 0.150914368 | 0.104415824 | PLA2G2F | 1 |
| Biological process | GO:0050772 | positive regulation of axonogenesis | 0.060160362 | 0.151465691 | 0.104797277 | ROBO1 | 1 |
| Biological process | GO:0051966 | regulation of synaptic transmission, glutamatergic | 0.060160362 | 0.151465691 | 0.104797277 | CNR1 | 1 |
| Biological process | GO:0002437 | inflammatory response to antigenic stimulus | 0.060909238 | 0.151875109 | 0.105080549 | CNR1 | 1 |
| Biological process | GO:0036465 | synaptic vesicle recycling | 0.060909238 | 0.151875109 | 0.105080549 | SH3GL2 | 1 |
| Biological process | GO:0003151 | outflow tract morphogenesis | 0.061657558 | 0.151875109 | 0.105080549 | ROBO1 | 1 |
| Biological process | GO:0070227 | lymphocyte apoptotic process | 0.061657558 | 0.151875109 | 0.105080549 | FASLG | 1 |
| Biological process | GO:1901184 | regulation of ERBB signaling pathway | 0.061657558 | 0.151875109 | 0.105080549 | FASLG | 1 |
| Biological process | GO:0044773 | mitotic DNA damage checkpoint signaling | 0.06240532 | 0.152397542 | 0.105442014 | FOXN3 | 1 |
| Biological process | GO:1901657 | glycosyl compound metabolic process | 0.06240532 | 0.152397542 | 0.105442014 | ABHD10 | 1 |
| Biological process | GO:1905897 | regulation of response to endoplasmic reticulum stress | 0.063152527 | 0.153563195 | 0.106248516 | DNAJB9 | 1 |
| Biological process | GO:0015844 | monoamine transport | 0.064645273 | 0.155679383 | 0.10771268 | CNR1 | 1 |
| Biological process | GO:0097194 | execution phase of apoptosis | 0.064645273 | 0.155679383 | 0.10771268 | FASLG | 1 |
| Biological process | GO:0008625 | extrinsic apoptotic signaling pathway via death domain receptors | 0.065390813 | 0.155679383 | 0.10771268 | FASLG | 1 |
| Biological process | GO:0044774 | mitotic DNA integrity checkpoint signaling | 0.065390813 | 0.155679383 | 0.10771268 | FOXN3 | 1 |
| Biological process | GO:0051453 | regulation of intracellular pH | 0.065390813 | 0.155679383 | 0.10771268 | FASLG | 1 |
| Biological process | GO:0032456 | endocytic recycling | 0.066135798 | 0.156146346 | 0.108035766 | VPS53 | 1 |
| Biological process | GO:0045921 | positive regulation of exocytosis | 0.066135798 | 0.156146346 | 0.108035766 | CNR1 | 1 |
| Biological process | GO:0030433 | ubiquitin-dependent ERAD pathway | 0.06688023 | 0.156415109 | 0.10822172 | DNAJB9 | 1 |
| Biological process | GO:0050672 | negative regulation of lymphocyte proliferation | 0.06688023 | 0.156415109 | 0.10822172 | PLA2G2F | 1 |
| Biological process | GO:0032370 | positive regulation of lipid transport | 0.067624107 | 0.156415109 | 0.10822172 | FASLG | 1 |
| Biological process | GO:0032945 | negative regulation of mononuclear cell proliferation | 0.067624107 | 0.156415109 | 0.10822172 | PLA2G2F | 1 |
| Biological process | GO:0035023 | regulation of Rho protein signal transduction | 0.067624107 | 0.156415109 | 0.10822172 | ROBO1 | 1 |
| Biological process | GO:0001960 | negative regulation of cytokine-mediated signaling pathway | 0.068367431 | 0.157494203 | 0.108968332 | ROBO1 | 1 |
| Biological process | GO:0030641 | regulation of cellular pH | 0.069110202 | 0.157926525 | 0.10926745 | FASLG | 1 |
| Biological process | GO:0070098 | chemokine-mediated signaling pathway | 0.069110202 | 0.157926525 | 0.10926745 | ROBO1 | 1 |
| Biological process | GO:0046928 | regulation of neurotransmitter secretion | 0.06985242 | 0.158350704 | 0.109560935 | CNR1 | 1 |
| Biological process | GO:1901216 | positive regulation of neuron death | 0.06985242 | 0.158350704 | 0.109560935 | FASLG | 1 |
| Biological process | GO:0042147 | retrograde transport, endosome to Golgi | 0.071335199 | 0.16107035 | 0.111442625 | VPS53 | 1 |
| Biological process | GO:0002042 | cell migration involved in sprouting angiogenesis | 0.072075761 | 0.161461055 | 0.111712949 | ROBO1 | 1 |
| Biological process | GO:0060761 | negative regulation of response to cytokine stimulus | 0.072075761 | 0.161461055 | 0.111712949 | ROBO1 | 1 |
| Biological process | GO:1903510 | mucopolysaccharide metabolic process | 0.072815772 | 0.1620801 | 0.112141259 | ITIH2 | 1 |
| Biological process | GO:0007032 | endosome organization | 0.073555232 | 0.1620801 | 0.112141259 | FASLG | 1 |
| Biological process | GO:0019217 | regulation of fatty acid metabolic process | 0.073555232 | 0.1620801 | 0.112141259 | CNR1 | 1 |
| Biological process | GO:0070664 | negative regulation of leukocyte proliferation | 0.073555232 | 0.1620801 | 0.112141259 | PLA2G2F | 1 |
| Biological process | GO:0008593 | regulation of Notch signaling pathway | 0.074294141 | 0.1620801 | 0.112141259 | ROBO1 | 1 |
| Biological process | GO:0051952 | regulation of amine transport | 0.075032501 | 0.1620801 | 0.112141259 | CNR1 | 1 |
| Biological process | GO:0060349 | bone morphogenesis | 0.075032501 | 0.1620801 | 0.112141259 | FOXN3 | 1 |
| Biological process | GO:1990868 | response to chemokine | 0.075032501 | 0.1620801 | 0.112141259 | ROBO1 | 1 |
| Biological process | GO:1990869 | cellular response to chemokine | 0.075032501 | 0.1620801 | 0.112141259 | ROBO1 | 1 |
| Biological process | GO:0006885 | regulation of pH | 0.07577031 | 0.1620801 | 0.112141259 | FASLG | 1 |
| Biological process | GO:0010389 | regulation of G2/M transition of mitotic cell cycle | 0.07577031 | 0.1620801 | 0.112141259 | FOXN3 | 1 |
| Biological process | GO:0043279 | response to alkaloid | 0.07577031 | 0.1620801 | 0.112141259 | CNR1 | 1 |
| Biological process | GO:0034620 | cellular response to unfolded protein | 0.076507571 | 0.162785173 | 0.11262909 | DNAJB9 | 1 |
| Biological process | GO:0009062 | fatty acid catabolic process | 0.077244282 | 0.162785173 | 0.11262909 | CNR1 | 1 |
| Biological process | GO:0015908 | fatty acid transport | 0.077244282 | 0.162785173 | 0.11262909 | PLA2G2F | 1 |
| Biological process | GO:0021549 | cerebellum development | 0.077244282 | 0.162785173 | 0.11262909 | ATRN | 1 |
| Biological process | GO:0035249 | synaptic transmission, glutamatergic | 0.077980445 | 0.16373016 | 0.113282915 | CNR1 | 1 |
| Biological process | GO:0019233 | sensory perception of pain | 0.07871606 | 0.164667052 | 0.11393114 | CNR1 | 1 |
| Biological process | GO:0015914 | phospholipid transport | 0.079451127 | 0.164991574 | 0.114155672 | FASLG | 1 |
| Biological process | GO:0060291 | long-term synaptic potentiation | 0.079451127 | 0.164991574 | 0.114155672 | SCGN | 1 |
| Biological process | GO:0051588 | regulation of neurotransmitter transport | 0.080185647 | 0.165911392 | 0.114792084 | CNR1 | 1 |
| Biological process | GO:0015837 | amine transport | 0.080919619 | 0.165954323 | 0.114821787 | CNR1 | 1 |
| Biological process | GO:0003279 | cardiac septum development | 0.081653045 | 0.165954323 | 0.114821787 | ROBO1 | 1 |
| Biological process | GO:0060840 | artery development | 0.081653045 | 0.165954323 | 0.114821787 | ROBO1 | 1 |
| Biological process | GO:0007631 | feeding behavior | 0.082385925 | 0.165954323 | 0.114821787 | CNR1 | 1 |
| Biological process | GO:0051341 | regulation of oxidoreductase activity | 0.082385925 | 0.165954323 | 0.114821787 | CNR1 | 1 |
| Biological process | GO:0062014 | negative regulation of small molecule metabolic process | 0.082385925 | 0.165954323 | 0.114821787 | CNR1 | 1 |
| Biological process | GO:0007173 | epidermal growth factor receptor signaling pathway | 0.083850046 | 0.165954323 | 0.114821787 | FASLG | 1 |
| Biological process | GO:0019395 | fatty acid oxidation | 0.083850046 | 0.165954323 | 0.114821787 | CNR1 | 1 |
| Biological process | GO:0045833 | negative regulation of lipid metabolic process | 0.083850046 | 0.165954323 | 0.114821787 | CNR1 | 1 |
| Biological process | GO:1902749 | regulation of cell cycle G2/M phase transition | 0.083850046 | 0.165954323 | 0.114821787 | FOXN3 | 1 |
| Biological process | GO:1904035 | regulation of epithelial cell apoptotic process | 0.083850046 | 0.165954323 | 0.114821787 | FASLG | 1 |
| Biological process | GO:0001676 | long-chain fatty acid metabolic process | 0.084581289 | 0.165954323 | 0.114821787 | PLA2G2F | 1 |
| Biological process | GO:0016079 | synaptic vesicle exocytosis | 0.084581289 | 0.165954323 | 0.114821787 | CNR1 | 1 |
| Biological process | GO:0022037 | metencephalon development | 0.084581289 | 0.165954323 | 0.114821787 | ATRN | 1 |
| Biological process | GO:0034446 | substrate adhesion-dependent cell spreading | 0.084581289 | 0.165954323 | 0.114821787 | ATRN | 1 |
| Biological process | GO:0036503 | ERAD pathway | 0.085311988 | 0.16624151 | 0.115020488 | DNAJB9 | 1 |
| Biological process | GO:0043473 | pigmentation | 0.085311988 | 0.16624151 | 0.115020488 | ATRN | 1 |
| Biological process | GO:1905954 | positive regulation of lipid localization | 0.086042141 | 0.167092077 | 0.115608985 | FASLG | 1 |
| Biological process | GO:0002526 | acute inflammatory response | 0.086771751 | 0.16736653 | 0.115798876 | CNR1 | 1 |
| Biological process | GO:0030004 | cellular monovalent inorganic cation homeostasis | 0.086771751 | 0.16736653 | 0.115798876 | FASLG | 1 |
| Biological process | GO:0033559 | unsaturated fatty acid metabolic process | 0.087500817 | 0.168202585 | 0.116377332 | PLA2G2F | 1 |
| Biological process | GO:0034440 | lipid oxidation | 0.08822934 | 0.168722384 | 0.116736974 | CNR1 | 1 |
| Biological process | GO:0021987 | cerebral cortex development | 0.08895732 | 0.168722384 | 0.116736974 | ROBO1 | 1 |
| Biological process | GO:0043406 | positive regulation of MAP kinase activity | 0.08895732 | 0.168722384 | 0.116736974 | ROBO1 | 1 |
| Biological process | GO:0071887 | leukocyte apoptotic process | 0.08895732 | 0.168722384 | 0.116736974 | FASLG | 1 |
| Biological process | GO:0051261 | protein depolymerization | 0.089684758 | 0.168975586 | 0.116912162 | SH3GL2 | 1 |
| Biological process | GO:0071346 | cellular response to interferon-gamma | 0.089684758 | 0.168975586 | 0.116912162 | FASLG | 1 |
| Biological process | GO:0015718 | monocarboxylic acid transport | 0.090411653 | 0.169782939 | 0.11747076 | PLA2G2F | 1 |
| Biological process | GO:0007613 | memory | 0.091138006 | 0.170024674 | 0.117638014 | CNR1 | 1 |
| Biological process | GO:0035967 | cellular response to topologically incorrect protein | 0.091138006 | 0.170024674 | 0.117638014 | DNAJB9 | 1 |
| Biological process | GO:0000077 | DNA damage checkpoint signaling | 0.091863819 | 0.170818669 | 0.118187369 | FOXN3 | 1 |
| Biological process | GO:0030203 | glycosaminoglycan metabolic process | 0.09331382 | 0.172949719 | 0.119661817 | ITIH2 | 1 |
| Biological process | GO:0051209 | release of sequestered calcium ion into cytosol | 0.094038011 | 0.173028137 | 0.119716073 | FASLG | 1 |
| Biological process | GO:0051283 | negative regulation of sequestering of calcium ion | 0.094761661 | 0.173028137 | 0.119716073 | FASLG | 1 |
| Biological process | GO:0003206 | cardiac chamber morphogenesis | 0.095484772 | 0.173028137 | 0.119716073 | ROBO1 | 1 |
| Biological process | GO:0003231 | cardiac ventricle development | 0.095484772 | 0.173028137 | 0.119716073 | ROBO1 | 1 |
| Biological process | GO:0006690 | icosanoid metabolic process | 0.095484772 | 0.173028137 | 0.119716073 | PLA2G2F | 1 |
| Biological process | GO:0038127 | ERBB signaling pathway | 0.095484772 | 0.173028137 | 0.119716073 | FASLG | 1 |
| Biological process | GO:0072329 | monocarboxylic acid catabolic process | 0.095484772 | 0.173028137 | 0.119716073 | CNR1 | 1 |
| Biological process | GO:0051282 | regulation of sequestering of calcium ion | 0.096207343 | 0.173635455 | 0.120136269 | FASLG | 1 |
| Biological process | GO:0045471 | response to ethanol | 0.096929376 | 0.173635455 | 0.120136269 | CNR1 | 1 |
| Biological process | GO:0050868 | negative regulation of T cell activation | 0.096929376 | 0.173635455 | 0.120136269 | PLA2G2F | 1 |
| Biological process | GO:0007041 | lysosomal transport | 0.097650871 | 0.173635455 | 0.120136269 | VPS53 | 1 |
| Biological process | GO:0031570 | DNA integrity checkpoint signaling | 0.097650871 | 0.173635455 | 0.120136269 | FOXN3 | 1 |
| Biological process | GO:2000027 | regulation of animal organ morphogenesis | 0.097650871 | 0.173635455 | 0.120136269 | ROBO1 | 1 |
| Biological process | GO:0051208 | sequestering of calcium ion | 0.099092246 | 0.175649495 | 0.121529759 | FASLG | 1 |
| Biological process | GO:0002702 | positive regulation of production of molecular mediator of immune response | 0.100531472 | 0.177097238 | 0.122531434 | DNAJB9 | 1 |
| Biological process | GO:0035601 | protein deacylation | 0.100531472 | 0.177097238 | 0.122531434 | ABHD10 | 1 |
| Biological process | GO:0006022 | aminoglycan metabolic process | 0.10125028 | 0.177812992 | 0.123026656 | ITIH2 | 1 |
| Biological process | GO:0010565 | regulation of cellular ketone metabolic process | 0.102686288 | 0.179779994 | 0.124387601 | CNR1 | 1 |
| Biological process | GO:0098732 | macromolecule deacylation | 0.103403489 | 0.179928395 | 0.124490278 | ABHD10 | 1 |
| Biological process | GO:1904019 | epithelial cell apoptotic process | 0.103403489 | 0.179928395 | 0.124490278 | FASLG | 1 |
| Biological process | GO:0030879 | mammary gland development | 0.104120155 | 0.180623073 | 0.124970917 | ROBO1 | 1 |
| Biological process | GO:0008277 | regulation of G protein-coupled receptor signaling pathway | 0.104836286 | 0.180900666 | 0.12516298 | RGS6 | 1 |
| Biological process | GO:0007266 | Rho protein signal transduction | 0.105551882 | 0.180900666 | 0.12516298 | ROBO1 | 1 |
| Biological process | GO:0034341 | response to interferon-gamma | 0.105551882 | 0.180900666 | 0.12516298 | FASLG | 1 |
| Biological process | GO:0042157 | lipoprotein metabolic process | 0.105551882 | 0.180900666 | 0.12516298 | ABHD10 | 1 |
| Biological process | GO:0006986 | response to unfolded protein | 0.106266945 | 0.181035604 | 0.125256343 | DNAJB9 | 1 |
| Biological process | GO:0007093 | mitotic cell cycle checkpoint signaling | 0.106266945 | 0.181035604 | 0.125256343 | FOXN3 | 1 |
| Biological process | GO:0000086 | G2/M transition of mitotic cell cycle | 0.106981474 | 0.181168032 | 0.125347968 | FOXN3 | 1 |
| Biological process | GO:0042552 | myelination | 0.106981474 | 0.181168032 | 0.125347968 | ATRN | 1 |
| Biological process | GO:0007272 | ensheathment of neurons | 0.108408933 | 0.182499062 | 0.126268892 | ATRN | 1 |
| Biological process | GO:0008366 | axon ensheathment | 0.108408933 | 0.182499062 | 0.126268892 | ATRN | 1 |
| Biological process | GO:1903305 | regulation of regulated secretory pathway | 0.109121864 | 0.183157347 | 0.126724351 | CNR1 | 1 |
| Biological process | GO:0001508 | action potential | 0.109834263 | 0.183810869 | 0.127176515 | CNR1 | 1 |
| Biological process | GO:0035264 | multicellular organism growth | 0.110546129 | 0.183920315 | 0.127252239 | ATRN | 1 |
| Biological process | GO:1903038 | negative regulation of leukocyte cell-cell adhesion | 0.110546129 | 0.183920315 | 0.127252239 | PLA2G2F | 1 |
| Biological process | GO:0015748 | organophosphate ester transport | 0.111257465 | 0.184336958 | 0.127540509 | FASLG | 1 |
| Biological process | GO:0016525 | negative regulation of angiogenesis | 0.111968269 | 0.184336958 | 0.127540509 | FASLG | 1 |
| Biological process | GO:0050796 | regulation of insulin secretion | 0.111968269 | 0.184336958 | 0.127540509 | CNR1 | 1 |
| Biological process | GO:0098876 | vesicle-mediated transport to the plasma membrane | 0.112678543 | 0.184336958 | 0.127540509 | VPS53 | 1 |
| Biological process | GO:0007269 | neurotransmitter secretion | 0.113388287 | 0.184336958 | 0.127540509 | CNR1 | 1 |
| Biological process | GO:0099643 | signal release from synapse | 0.113388287 | 0.184336958 | 0.127540509 | CNR1 | 1 |
| Biological process | GO:1903531 | negative regulation of secretion by cell | 0.113388287 | 0.184336958 | 0.127540509 | CNR1 | 1 |
| Biological process | GO:2000181 | negative regulation of blood vessel morphogenesis | 0.113388287 | 0.184336958 | 0.127540509 | FASLG | 1 |
| Biological process | GO:0030183 | B cell differentiation | 0.1140975 | 0.184436016 | 0.127609047 | DNAJB9 | 1 |
| Biological process | GO:1901343 | negative regulation of vasculature development | 0.1140975 | 0.184436016 | 0.127609047 | FASLG | 1 |
| Biological process | GO:0030902 | hindbrain development | 0.114806185 | 0.185055861 | 0.12803791 | ATRN | 1 |
| Biological process | GO:0007584 | response to nutrient | 0.11551434 | 0.185148336 | 0.128101892 | CNR1 | 1 |
| Biological process | GO:0050729 | positive regulation of inflammatory response | 0.11551434 | 0.185148336 | 0.128101892 | CNR1 | 1 |
| Biological process | GO:0016052 | carbohydrate catabolic process | 0.116221966 | 0.185238932 | 0.128164574 | ABHD10 | 1 |
| Biological process | GO:0032368 | regulation of lipid transport | 0.116221966 | 0.185238932 | 0.128164574 | FASLG | 1 |
| Biological process | GO:0050770 | regulation of axonogenesis | 0.116929064 | 0.185414614 | 0.128286126 | ROBO1 | 1 |
| Biological process | GO:0007189 | adenylate cyclase-activating G protein-coupled receptor signaling pathway | 0.117635634 | 0.185414614 | 0.128286126 | CNR1 | 1 |
| Biological process | GO:0010976 | positive regulation of neuron projection development | 0.117635634 | 0.185414614 | 0.128286126 | CNR1 | 1 |
| Biological process | GO:0044839 | cell cycle G2/M phase transition | 0.117635634 | 0.185414614 | 0.128286126 | FOXN3 | 1 |
| Biological process | GO:0048592 | eye morphogenesis | 0.119047191 | 0.187121137 | 0.129466849 | FASLG | 1 |
| Biological process | GO:0120254 | olefinic compound metabolic process | 0.119752178 | 0.187710715 | 0.129874771 | PLA2G2F | 1 |
| Biological process | GO:0007034 | vacuolar transport | 0.121160575 | 0.189396613 | 0.131041223 | VPS53 | 1 |
| Biological process | GO:0035966 | response to topologically incorrect protein | 0.121863984 | 0.189974265 | 0.131440894 | DNAJB9 | 1 |
| Biological process | GO:0055067 | monovalent inorganic cation homeostasis | 0.122566868 | 0.190547944 | 0.131837816 | FASLG | 1 |
| Biological process | GO:0007009 | plasma membrane organization | 0.12397106 | 0.191209105 | 0.132295265 | FASLG | 1 |
| Biological process | GO:0051250 | negative regulation of lymphocyte activation | 0.12397106 | 0.191209105 | 0.132295265 | PLA2G2F | 1 |
| Biological process | GO:0001959 | regulation of cytokine-mediated signaling pathway | 0.124672369 | 0.191209105 | 0.132295265 | ROBO1 | 1 |
| Biological process | GO:0003205 | cardiac chamber development | 0.124672369 | 0.191209105 | 0.132295265 | ROBO1 | 1 |
| Biological process | GO:0043271 | negative regulation of ion transport | 0.124672369 | 0.191209105 | 0.132295265 | CNR1 | 1 |
| Biological process | GO:0007568 | aging | 0.126073416 | 0.192838102 | 0.133422348 | CNR1 | 1 |
| Biological process | GO:0007156 | homophilic cell adhesion via plasma membrane adhesion molecules | 0.126773155 | 0.193388539 | 0.133803189 | ROBO1 | 1 |
| Biological process | GO:0016482 | cytosolic transport | 0.12747237 | 0.193935237 | 0.134181442 | VPS53 | 1 |
| Biological process | GO:1990138 | neuron projection extension | 0.128171063 | 0.194478226 | 0.13455713 | SH3GL2 | 1 |
| Biological process | GO:0021543 | pallium development | 0.129566882 | 0.196073287 | 0.135660734 | ROBO1 | 1 |
| Biological process | GO:0051048 | negative regulation of secretion | 0.13026401 | 0.196605362 | 0.13602887 | CNR1 | 1 |
| Biological process | GO:0050680 | negative regulation of epithelial cell proliferation | 0.130960616 | 0.196613695 | 0.136034635 | ROBO1 | 1 |
| Biological process | GO:1901136 | carbohydrate derivative catabolic process | 0.130960616 | 0.196613695 | 0.136034635 | ABHD10 | 1 |
| Biological process | GO:0043534 | blood vessel endothelial cell migration | 0.131656701 | 0.197138587 | 0.136397801 | ROBO1 | 1 |
| Biological process | GO:0001659 | temperature homeostasis | 0.132352266 | 0.197659946 | 0.136758523 | CNR1 | 1 |
| Biological process | GO:0060759 | regulation of response to cytokine stimulus | 0.133047311 | 0.197660366 | 0.136758814 | ROBO1 | 1 |
| Biological process | GO:0090276 | regulation of peptide hormone secretion | 0.133047311 | 0.197660366 | 0.136758814 | CNR1 | 1 |
| Biological process | GO:0006575 | cellular modified amino acid metabolic process | 0.133741836 | 0.198161249 | 0.137105369 | PLA2G2F | 1 |
| Biological process | GO:0042770 | signal transduction in response to DNA damage | 0.134435842 | 0.198161249 | 0.137105369 | FOXN3 | 1 |
| Biological process | GO:0002791 | regulation of peptide secretion | 0.135129329 | 0.198161249 | 0.137105369 | CNR1 | 1 |
| Biological process | GO:0042129 | regulation of T cell proliferation | 0.135129329 | 0.198161249 | 0.137105369 | PLA2G2F | 1 |
| Biological process | GO:0097553 | calcium ion transmembrane import into cytosol | 0.135129329 | 0.198161249 | 0.137105369 | FASLG | 1 |
| Biological process | GO:0007219 | Notch signaling pathway | 0.135822297 | 0.198161249 | 0.137105369 | ROBO1 | 1 |
| Biological process | GO:0030073 | insulin secretion | 0.135822297 | 0.198161249 | 0.137105369 | CNR1 | 1 |
| Biological process | GO:0090087 | regulation of peptide transport | 0.136514748 | 0.198643078 | 0.13743874 | CNR1 | 1 |
| Biological process | GO:0002040 | sprouting angiogenesis | 0.13720668 | 0.198643078 | 0.13743874 | ROBO1 | 1 |
| Biological process | GO:1905952 | regulation of lipid localization | 0.13720668 | 0.198643078 | 0.13743874 | FASLG | 1 |
| Biological process | GO:0000075 | cell cycle checkpoint signaling | 0.137898094 | 0.198643078 | 0.13743874 | FOXN3 | 1 |
| Biological process | GO:0043405 | regulation of MAP kinase activity | 0.137898094 | 0.198643078 | 0.13743874 | ROBO1 | 1 |
| Biological process | GO:0002700 | regulation of production of molecular mediator of immune response | 0.138588992 | 0.199134183 | 0.13777853 | DNAJB9 | 1 |
| Biological process | GO:0007565 | female pregnancy | 0.139969237 | 0.200106773 | 0.138451454 | CNR1 | 1 |
| Biological process | GO:0043123 | positive regulation of I-kappaB kinase/NF-kappaB signaling | 0.139969237 | 0.200106773 | 0.138451454 | FASLG | 1 |
| Biological process | GO:0008217 | regulation of blood pressure | 0.141347417 | 0.201570628 | 0.139464277 | CNR1 | 1 |
| Biological process | GO:0046578 | regulation of Ras protein signal transduction | 0.142723537 | 0.203024231 | 0.140470007 | ROBO1 | 1 |
| Biological process | GO:1901991 | negative regulation of mitotic cell cycle phase transition | 0.143410824 | 0.203493164 | 0.140794456 | FOXN3 | 1 |
| Biological process | GO:0022408 | negative regulation of cell-cell adhesion | 0.148890638 | 0.210743217 | 0.14581068 | PLA2G2F | 1 |
| Biological process | GO:0007204 | positive regulation of cytosolic calcium ion concentration | 0.15093712 | 0.21310973 | 0.147448042 | CNR1 | 1 |
| Biological process | GO:2000241 | regulation of reproductive process | 0.151618259 | 0.213541558 | 0.147746819 | CNR1 | 1 |
| Biological process | GO:0048167 | regulation of synaptic plasticity | 0.152298889 | 0.213970537 | 0.148043624 | SCGN | 1 |
| Biological process | GO:0044703 | multi-organism reproductive process | 0.153658619 | 0.215349148 | 0.148997468 | CNR1 | 1 |
| Biological process | GO:0006836 | neurotransmitter transport | 0.155694397 | 0.217013788 | 0.150149212 | CNR1 | 1 |
| Biological process | GO:0042098 | T cell proliferation | 0.155694397 | 0.217013788 | 0.150149212 | PLA2G2F | 1 |
| Biological process | GO:0017157 | regulation of exocytosis | 0.156371974 | 0.217013788 | 0.150149212 | CNR1 | 1 |
| Biological process | GO:0030258 | lipid modification | 0.156371974 | 0.217013788 | 0.150149212 | CNR1 | 1 |
| Biological process | GO:0042180 | cellular ketone metabolic process | 0.15975225 | 0.219958655 | 0.15218673 | CNR1 | 1 |
| Biological process | GO:0043523 | regulation of neuron apoptotic process | 0.15975225 | 0.219958655 | 0.15218673 | FASLG | 1 |
| Biological process | GO:0044706 | multi-multicellular organism process | 0.15975225 | 0.219958655 | 0.15218673 | CNR1 | 1 |
| Biological process | GO:0001505 | regulation of neurotransmitter levels | 0.160426787 | 0.219958655 | 0.15218673 | CNR1 | 1 |
| Biological process | GO:0002377 | immunoglobulin production | 0.160426787 | 0.219958655 | 0.15218673 | DNAJB9 | 1 |
| Biological process | GO:0044242 | cellular lipid catabolic process | 0.162447366 | 0.222193632 | 0.153733084 | CNR1 | 1 |
| Biological process | GO:0097191 | extrinsic apoptotic signaling pathway | 0.163791896 | 0.223495416 | 0.154633772 | FASLG | 1 |
| Biological process | GO:0030072 | peptide hormone secretion | 0.164463405 | 0.223874826 | 0.154896282 | CNR1 | 1 |
| Biological process | GO:0051651 | maintenance of location in cell | 0.165134411 | 0.224251742 | 0.155157065 | FASLG | 1 |
| Biological process | GO:0048705 | skeletal system morphogenesis | 0.165804914 | 0.224626181 | 0.155416135 | FOXN3 | 1 |
| Biological process | GO:0048588 | developmental cell growth | 0.167144412 | 0.225202428 | 0.155814833 | SH3GL2 | 1 |
| Biological process | GO:0071902 | positive regulation of protein serine/threonine kinase activity | 0.167144412 | 0.225202428 | 0.155814833 | ROBO1 | 1 |
| Biological process | GO:0002790 | peptide secretion | 0.167813408 | 0.225202428 | 0.155814833 | CNR1 | 1 |
| Biological process | GO:0050920 | regulation of chemotaxis | 0.167813408 | 0.225202428 | 0.155814833 | ROBO1 | 1 |
| Biological process | GO:0050769 | positive regulation of neurogenesis | 0.168481903 | 0.225567536 | 0.156067447 | ROBO1 | 1 |
| Biological process | GO:0060348 | bone development | 0.169817389 | 0.226290619 | 0.156567739 | FOXN3 | 1 |
| Biological process | GO:0060560 | developmental growth involved in morphogenesis | 0.169817389 | 0.226290619 | 0.156567739 | SH3GL2 | 1 |
| Biological process | GO:0046883 | regulation of hormone secretion | 0.170484381 | 0.226648627 | 0.156815441 | CNR1 | 1 |
| Biological process | GO:0007411 | axon guidance | 0.171816864 | 0.227708726 | 0.15754891 | ROBO1 | 1 |
| Biological process | GO:0002274 | myeloid leukocyte activation | 0.172482357 | 0.227708726 | 0.15754891 | CNR1 | 1 |
| Biological process | GO:0097485 | neuron projection guidance | 0.172482357 | 0.227708726 | 0.15754891 | ROBO1 | 1 |
| Biological process | GO:0050670 | regulation of lymphocyte proliferation | 0.173811844 | 0.22893273 | 0.158395784 | PLA2G2F | 1 |
| Biological process | GO:0048738 | cardiac muscle tissue development | 0.175139338 | 0.229767723 | 0.158973505 | SGCG | 1 |
| Biological process | GO:0032984 | protein-containing complex disassembly | 0.175802339 | 0.229767723 | 0.158973505 | SH3GL2 | 1 |
| Biological process | GO:0045055 | regulated exocytosis | 0.175802339 | 0.229767723 | 0.158973505 | CNR1 | 1 |
| Biological process | GO:0032944 | regulation of mononuclear cell proliferation | 0.176464841 | 0.229767723 | 0.158973505 | PLA2G2F | 1 |
| Biological process | GO:0046395 | carboxylic acid catabolic process | 0.176464841 | 0.229767723 | 0.158973505 | CNR1 | 1 |
| Biological process | GO:0045930 | negative regulation of mitotic cell cycle | 0.178449369 | 0.230900851 | 0.159757503 | FOXN3 | 1 |
| Biological process | GO:0016054 | organic acid catabolic process | 0.179109886 | 0.230900851 | 0.159757503 | CNR1 | 1 |
| Biological process | GO:0016197 | endosomal transport | 0.179109886 | 0.230900851 | 0.159757503 | VPS53 | 1 |
| Biological process | GO:0031330 | negative regulation of cellular catabolic process | 0.179109886 | 0.230900851 | 0.159757503 | CNR1 | 1 |
| Biological process | GO:0007188 | adenylate cyclase-modulating G protein-coupled receptor signaling pathway | 0.179769907 | 0.230900851 | 0.159757503 | CNR1 | 1 |
| Biological process | GO:0042593 | glucose homeostasis | 0.179769907 | 0.230900851 | 0.159757503 | CNR1 | 1 |
| Biological process | GO:0033500 | carbohydrate homeostasis | 0.180429432 | 0.231226007 | 0.159982474 | CNR1 | 1 |
| Biological process | GO:0060047 | heart contraction | 0.181088463 | 0.231549068 | 0.160205997 | SGCG | 1 |
| Biological process | GO:0010951 | negative regulation of endopeptidase activity | 0.182405041 | 0.232616541 | 0.160944568 | ITIH2 | 1 |
| Biological process | GO:0097305 | response to alcohol | 0.183062589 | 0.232616541 | 0.160944568 | CNR1 | 1 |
| Biological process | GO:0015833 | peptide transport | 0.183719643 | 0.232616541 | 0.160944568 | CNR1 | 1 |
| Biological process | GO:0021537 | telencephalon development | 0.184376204 | 0.232616541 | 0.160944568 | ROBO1 | 1 |
| Biological process | GO:0043122 | regulation of I-kappaB kinase/NF-kappaB signaling | 0.184376204 | 0.232616541 | 0.160944568 | FASLG | 1 |
| Biological process | GO:0051402 | neuron apoptotic process | 0.184376204 | 0.232616541 | 0.160944568 | FASLG | 1 |
| Biological process | GO:0014706 | striated muscle tissue development | 0.185687847 | 0.233237053 | 0.161373893 | SGCG | 1 |
| Biological process | GO:0050708 | regulation of protein secretion | 0.185687847 | 0.233237053 | 0.161373893 | CNR1 | 1 |
| Biological process | GO:0003007 | heart morphogenesis | 0.186342931 | 0.233544334 | 0.161586497 | ROBO1 | 1 |
| Biological process | GO:0002699 | positive regulation of immune effector process | 0.18830523 | 0.234454433 | 0.162216184 | DNAJB9 | 1 |
| Biological process | GO:0003015 | heart process | 0.18830523 | 0.234454433 | 0.162216184 | SGCG | 1 |
| Biological process | GO:0034976 | response to endoplasmic reticulum stress | 0.18830523 | 0.234454433 | 0.162216184 | DNAJB9 | 1 |
| Biological process | GO:0010466 | negative regulation of peptidase activity | 0.189610975 | 0.235564727 | 0.162984382 | ITIH2 | 1 |
| Biological process | GO:0070663 | regulation of leukocyte proliferation | 0.193516457 | 0.23989295 | 0.165979028 | PLA2G2F | 1 |
| Biological process | GO:0003018 | vascular process in circulatory system | 0.194165661 | 0.24017448 | 0.166173815 | SH3GL2 | 1 |
| Biological process | GO:0090596 | sensory organ morphogenesis | 0.195462604 | 0.240732082 | 0.166559612 | FASLG | 1 |
| Biological process | GO:1901988 | negative regulation of cell cycle phase transition | 0.195462604 | 0.240732082 | 0.166559612 | FOXN3 | 1 |
| Biological process | GO:0007611 | learning or memory | 0.196110345 | 0.241008178 | 0.16675064 | CNR1 | 1 |
| Biological process | GO:0001894 | tissue homeostasis | 0.197404367 | 0.242075614 | 0.167489186 | SH3GL2 | 1 |
| Biological process | GO:1903532 | positive regulation of secretion by cell | 0.199341755 | 0.243925717 | 0.16876925 | CNR1 | 1 |
| Biological process | GO:0051962 | positive regulation of nervous system development | 0.19998658 | 0.244189623 | 0.168951843 | ROBO1 | 1 |
| Biological process | GO:0043542 | endothelial cell migration | 0.201274777 | 0.245236292 | 0.169676021 | ROBO1 | 1 |
| Biological process | GO:0098742 | cell-cell adhesion via plasma-membrane adhesion molecules | 0.20191815 | 0.245494502 | 0.169854674 | ROBO1 | 1 |
| Biological process | GO:0043547 | positive regulation of GTPase activity | 0.202561038 | 0.245751025 | 0.170032159 | RGS6 | 1 |
| Biological process | GO:0007249 | I-kappaB kinase/NF-kappaB signaling | 0.205768237 | 0.249110908 | 0.172356821 | FASLG | 1 |
| Biological process | GO:0046942 | carboxylic acid transport | 0.207047742 | 0.25012774 | 0.173060355 | PLA2G2F | 1 |
| Biological process | GO:0046879 | hormone secretion | 0.207686773 | 0.250368165 | 0.173226702 | CNR1 | 1 |
| Biological process | GO:0015850 | organic hydroxy compound transport | 0.208325324 | 0.250606996 | 0.173391946 | CNR1 | 1 |
| Biological process | GO:0042886 | amide transport | 0.210874725 | 0.252605723 | 0.174774841 | CNR1 | 1 |
| Biological process | GO:0044262 | cellular carbohydrate metabolic process | 0.210874725 | 0.252605723 | 0.174774841 | ABHD10 | 1 |
| Biological process | GO:0009914 | hormone transport | 0.213416462 | 0.255113376 | 0.176509856 | CNR1 | 1 |
| Biological process | GO:0051047 | positive regulation of secretion | 0.215950556 | 0.257276959 | 0.178006813 | CNR1 | 1 |
| Biological process | GO:0046651 | lymphocyte proliferation | 0.216582888 | 0.257276959 | 0.178006813 | PLA2G2F | 1 |
| Biological process | GO:0051056 | regulation of small GTPase mediated signal transduction | 0.216582888 | 0.257276959 | 0.178006813 | ROBO1 | 1 |
| Biological process | GO:0010720 | positive regulation of cell development | 0.217846124 | 0.257701548 | 0.178300581 | ROBO1 | 1 |
| Biological process | GO:0031349 | positive regulation of defense response | 0.217846124 | 0.257701548 | 0.178300581 | CNR1 | 1 |
| Biological process | GO:0007162 | negative regulation of cell adhesion | 0.218477029 | 0.25791168 | 0.178445969 | PLA2G2F | 1 |
| Biological process | GO:0006650 | glycerophospholipid metabolic process | 0.220366896 | 0.259604065 | 0.179616909 | PLA2G2F | 1 |
| Biological process | GO:0032943 | mononuclear cell proliferation | 0.220995902 | 0.259807166 | 0.179757433 | PLA2G2F | 1 |
| Biological process | GO:0060485 | mesenchyme development | 0.221624436 | 0.260008874 | 0.179896992 | ROBO1 | 1 |
| Biological process | GO:0050890 | cognition | 0.222252496 | 0.260209197 | 0.180035593 | CNR1 | 1 |
| Biological process | GO:0010948 | negative regulation of cell cycle process | 0.224760008 | 0.262604609 | 0.18169295 | FOXN3 | 1 |
| Biological process | GO:0015849 | organic acid transport | 0.228507133 | 0.266435571 | 0.184343546 | PLA2G2F | 1 |
| Biological process | GO:1901214 | regulation of neuron death | 0.229130008 | 0.26661549 | 0.184468029 | FASLG | 1 |
| Biological process | GO:0009895 | negative regulation of catabolic process | 0.233477008 | 0.270567042 | 0.18720206 | CNR1 | 1 |
| Biological process | GO:0048638 | regulation of developmental growth | 0.233477008 | 0.270567042 | 0.18720206 | ATRN | 1 |
| Biological process | GO:0002440 | production of molecular mediator of immune response | 0.234096137 | 0.270733134 | 0.187316978 | DNAJB9 | 1 |
| Biological process | GO:0001933 | negative regulation of protein phosphorylation | 0.237184787 | 0.273194623 | 0.189020052 | SH3GL2 | 1 |
| Biological process | GO:0090287 | regulation of cellular response to growth factor stimulus | 0.237184787 | 0.273194623 | 0.189020052 | ROBO1 | 1 |
| Biological process | GO:0062012 | regulation of small molecule metabolic process | 0.237801121 | 0.273351187 | 0.189128377 | CNR1 | 1 |
| Biological process | GO:0051235 | maintenance of location | 0.239647333 | 0.274918009 | 0.190212442 | FASLG | 1 |
| Biological process | GO:0070588 | calcium ion transmembrane transport | 0.241489371 | 0.276473747 | 0.191288838 | FASLG | 1 |
| Biological process | GO:0019216 | regulation of lipid metabolic process | 0.242715082 | 0.276906403 | 0.191588187 | CNR1 | 1 |
| Biological process | GO:0007517 | muscle organ development | 0.243327243 | 0.276906403 | 0.191588187 | SGCG | 1 |
| Biological process | GO:0070661 | leukocyte proliferation | 0.243327243 | 0.276906403 | 0.191588187 | PLA2G2F | 1 |
| Biological process | GO:0045765 | regulation of angiogenesis | 0.243938943 | 0.277048421 | 0.191686448 | FASLG | 1 |
| Biological process | GO:0042113 | B cell activation | 0.244550182 | 0.277189349 | 0.191783954 | DNAJB9 | 1 |
| Biological process | GO:0045861 | negative regulation of proteolysis | 0.245771275 | 0.27760567 | 0.192072002 | ITIH2 | 1 |
| Biological process | GO:0007265 | Ras protein signal transduction | 0.24638113 | 0.27760567 | 0.192072002 | ROBO1 | 1 |
| Biological process | GO:0016050 | vesicle organization | 0.24638113 | 0.27760567 | 0.192072002 | FASLG | 1 |
| Biological process | GO:1901990 | regulation of mitotic cell cycle phase transition | 0.246990526 | 0.277742311 | 0.192166542 | FOXN3 | 1 |
| Biological process | GO:1901342 | regulation of vasculature development | 0.247599461 | 0.277877896 | 0.192260352 | FASLG | 1 |
| Biological process | GO:0009306 | protein secretion | 0.25003061 | 0.27963254 | 0.193474368 | CNR1 | 1 |
| Biological process | GO:0003002 | regionalization | 0.25063725 | 0.27963254 | 0.193474368 | ROBO1 | 1 |
| Biological process | GO:0035592 | establishment of protein localization to extracellular region | 0.25063725 | 0.27963254 | 0.193474368 | CNR1 | 1 |
| Biological process | GO:0010631 | epithelial cell migration | 0.254267486 | 0.28183268 | 0.194996619 | ROBO1 | 1 |
| Biological process | GO:0030336 | negative regulation of cell migration | 0.255473912 | 0.28183268 | 0.194996619 | ROBO1 | 1 |
| Biological process | GO:0070997 | neuron death | 0.255473912 | 0.28183268 | 0.194996619 | FASLG | 1 |
| Biological process | GO:0071692 | protein localization to extracellular region | 0.255473912 | 0.28183268 | 0.194996619 | CNR1 | 1 |
| Biological process | GO:0006887 | exocytosis | 0.256076442 | 0.28183268 | 0.194996619 | CNR1 | 1 |
| Biological process | GO:0031589 | cell-substrate adhesion | 0.256076442 | 0.28183268 | 0.194996619 | ATRN | 1 |
| Biological process | GO:0090132 | epithelium migration | 0.256076442 | 0.28183268 | 0.194996619 | ROBO1 | 1 |
| Biological process | GO:0015711 | organic anion transport | 0.257881302 | 0.28327116 | 0.195991886 | PLA2G2F | 1 |
| Biological process | GO:0051346 | negative regulation of hydrolase activity | 0.258482013 | 0.283284168 | 0.196000886 | ITIH2 | 1 |
| Biological process | GO:0090130 | tissue migration | 0.259082271 | 0.283284168 | 0.196000886 | ROBO1 | 1 |
| Biological process | GO:0043087 | regulation of GTPase activity | 0.260281425 | 0.283284168 | 0.196000886 | RGS6 | 1 |
| Biological process | GO:0050863 | regulation of T cell activation | 0.260281425 | 0.283284168 | 0.196000886 | PLA2G2F | 1 |
| Biological process | GO:0050767 | regulation of neurogenesis | 0.260880323 | 0.283284168 | 0.196000886 | ROBO1 | 1 |
| Biological process | GO:1903037 | regulation of leukocyte cell-cell adhesion | 0.260880323 | 0.283284168 | 0.196000886 | PLA2G2F | 1 |
| Biological process | GO:0002697 | regulation of immune effector process | 0.262076761 | 0.28404129 | 0.196524729 | DNAJB9 | 1 |
| Biological process | GO:0042326 | negative regulation of phosphorylation | 0.263271391 | 0.284793577 | 0.197045228 | SH3GL2 | 1 |
| Biological process | GO:0030900 | forebrain development | 0.264464216 | 0.285000264 | 0.197188232 | ROBO1 | 1 |
| Biological process | GO:2000146 | negative regulation of cell motility | 0.264464216 | 0.285000264 | 0.197188232 | ROBO1 | 1 |
| Biological process | GO:0044282 | small molecule catabolic process | 0.265655239 | 0.285742591 | 0.197701839 | CNR1 | 1 |
| Biological process | GO:0001654 | eye development | 0.267438398 | 0.287117827 | 0.198653348 | FASLG | 1 |
| Biological process | GO:0006644 | phospholipid metabolic process | 0.268031885 | 0.287213075 | 0.198719249 | PLA2G2F | 1 |
| Biological process | GO:0150063 | visual system development | 0.269809657 | 0.288574614 | 0.199661281 | FASLG | 1 |
| Biological process | GO:0071900 | regulation of protein serine/threonine kinase activity | 0.271583398 | 0.289926742 | 0.200596802 | ROBO1 | 1 |
| Biological process | GO:0048880 | sensory system development | 0.273353117 | 0.291269519 | 0.201525854 | FASLG | 1 |
| Biological process | GO:0046486 | glycerolipid metabolic process | 0.273942131 | 0.291351537 | 0.201582601 | PLA2G2F | 1 |
| Biological process | GO:0045786 | negative regulation of cell cycle | 0.277466867 | 0.294549715 | 0.203795381 | FOXN3 | 1 |
| Biological process | GO:0007159 | leukocyte cell-cell adhesion | 0.282724038 | 0.298460069 | 0.206500908 | PLA2G2F | 1 |
| Biological process | GO:0050678 | regulation of epithelial cell proliferation | 0.282724038 | 0.298460069 | 0.206500908 | ROBO1 | 1 |
| Biological process | GO:0050727 | regulation of inflammatory response | 0.282724038 | 0.298460069 | 0.206500908 | CNR1 | 1 |
| Biological process | GO:0030098 | lymphocyte differentiation | 0.28562924 | 0.300412269 | 0.207851611 | DNAJB9 | 1 |
| Biological process | GO:0040013 | negative regulation of locomotion | 0.28562924 | 0.300412269 | 0.207851611 | ROBO1 | 1 |
| Biological process | GO:0045860 | positive regulation of protein kinase activity | 0.286208961 | 0.300466603 | 0.207889204 | ROBO1 | 1 |
| Biological process | GO:0060537 | muscle tissue development | 0.287367085 | 0.301126835 | 0.208346011 | SGCG | 1 |
| Biological process | GO:0042391 | regulation of membrane potential | 0.292556977 | 0.306001691 | 0.211718865 | CNR1 | 1 |
| Biological process | GO:0006979 | response to oxidative stress | 0.294279087 | 0.307238166 | 0.212574367 | ATRN | 1 |
| Biological process | GO:0032102 | negative regulation of response to external stimulus | 0.295424986 | 0.307869628 | 0.213011268 | ROBO1 | 1 |
| Biological process | GO:0007409 | axonogenesis | 0.296569148 | 0.308026383 | 0.213119725 | ROBO1 | 1 |
| Biological process | GO:0006816 | calcium ion transport | 0.297711575 | 0.308026383 | 0.213119725 | FASLG | 1 |
| Biological process | GO:0045936 | negative regulation of phosphate metabolic process | 0.297711575 | 0.308026383 | 0.213119725 | SH3GL2 | 1 |
| Biological process | GO:0010563 | negative regulation of phosphorus metabolic process | 0.298282139 | 0.308026383 | 0.213119725 | SH3GL2 | 1 |
| Biological process | GO:0048732 | gland development | 0.298282139 | 0.308026383 | 0.213119725 | ROBO1 | 1 |
| Biological process | GO:0043161 | proteasome-mediated ubiquitin-dependent protein catabolic process | 0.303397783 | 0.312741556 | 0.216382096 | DNAJB9 | 1 |
| Biological process | GO:1901987 | regulation of cell cycle phase transition | 0.305095248 | 0.313922597 | 0.217199244 | FOXN3 | 1 |
| Biological process | GO:0051960 | regulation of nervous system development | 0.306788852 | 0.31509541 | 0.218010699 | ROBO1 | 1 |
| Biological process | GO:0032103 | positive regulation of response to external stimulus | 0.311286314 | 0.319138581 | 0.22080812 | CNR1 | 1 |
| Biological process | GO:0023061 | signal release | 0.314641522 | 0.321962396 | 0.222761883 | CNR1 | 1 |
| Biological process | GO:0007389 | pattern specification process | 0.31575653 | 0.321962396 | 0.222761883 | ROBO1 | 1 |
| Biological process | GO:0044772 | mitotic cell cycle phase transition | 0.316313399 | 0.321962396 | 0.222761883 | FOXN3 | 1 |
| Biological process | GO:1903131 | mononuclear cell differentiation | 0.316313399 | 0.321962396 | 0.222761883 | DNAJB9 | 1 |
| Biological process | GO:0031667 | response to nutrient levels | 0.316869845 | 0.321962396 | 0.222761883 | CNR1 | 1 |
| Biological process | GO:0051051 | negative regulation of transport | 0.31964574 | 0.324203968 | 0.2243128 | CNR1 | 1 |
| Biological process | GO:0050673 | epithelial cell proliferation | 0.320753147 | 0.324748293 | 0.224689412 | ROBO1 | 1 |
| Biological process | GO:0022411 | cellular component disassembly | 0.321858872 | 0.325288984 | 0.22506351 | SH3GL2 | 1 |
| Biological process | GO:0022407 | regulation of cell-cell adhesion | 0.325715693 | 0.328603244 | 0.227356606 | PLA2G2F | 1 |
| Biological process | GO:0001667 | ameboidal-type cell migration | 0.326813876 | 0.329005235 | 0.227634739 | ROBO1 | 1 |
| Biological process | GO:0016049 | cell growth | 0.327362341 | 0.329005235 | 0.227634739 | SH3GL2 | 1 |
| Biological process | GO:0019221 | cytokine-mediated signaling pathway | 0.329005235 | 0.329005235 | 0.227634739 | ROBO1 | 1 |
| Biological process | GO:0033674 | positive regulation of kinase activity | 0.329005235 | 0.329005235 | 0.227634739 | ROBO1 | 1 |
| Biological process | GO:0043410 | positive regulation of MAPK cascade | 0.329005235 | 0.329005235 | 0.227634739 | ROBO1 | 1 |
| Cellular component | GO:0016010 | dystrophin-associated glycoprotein complex | 0.012761951 | 0.190743074 | 0.165557239 | SGCG | 1 |
| Cellular component | GO:0090665 | glycoprotein complex | 0.016486436 | 0.190743074 | 0.165557239 | SGCG | 1 |
| Cellular component | GO:0150034 | distal axon | 0.018164003 | 0.190743074 | 0.165557239 | SCGN/CNR1 | 2 |
| Cellular component | GO:0005788 | endoplasmic reticulum lumen | 0.022552467 | 0.190743074 | 0.165557239 | DNAJB9/ITIH2 | 2 |
| Cellular component | GO:0098978 | glutamatergic synapse | 0.024197252 | 0.190743074 | 0.165557239 | SH3GL2/CNR1 | 2 |
| Cellular component | GO:0045121 | membrane raft | 0.024476131 | 0.190743074 | 0.165557239 | FASLG/CNR1 | 2 |
| Cellular component | GO:0098857 | membrane microdomain | 0.024616076 | 0.190743074 | 0.165557239 | FASLG/CNR1 | 2 |
| Cellular component | GO:0043195 | terminal bouton | 0.034912955 | 0.190743074 | 0.165557239 | SCGN | 1 |
| Cellular component | GO:0062023 | collagen-containing extracellular matrix | 0.041255616 | 0.190743074 | 0.165557239 | ATRN/ITIH2 | 2 |
| Cellular component | GO:0099023 | vesicle tethering complex | 0.045813672 | 0.190743074 | 0.165557239 | VPS53 | 1 |
| Cellular component | GO:0030669 | clathrin-coated endocytic vesicle membrane | 0.053016856 | 0.190743074 | 0.165557239 | SH3GL2 | 1 |
| Cellular component | GO:0098982 | GABA-ergic synapse | 0.053016856 | 0.190743074 | 0.165557239 | CNR1 | 1 |
| Cellular component | GO:0099056 | integral component of presynaptic membrane | 0.053734376 | 0.190743074 | 0.165557239 | CNR1 | 1 |
| Cellular component | GO:0033116 | endoplasmic reticulum-Golgi intermediate compartment membrane | 0.058742828 | 0.190743074 | 0.165557239 | ROBO1 | 1 |
| Cellular component | GO:0098889 | intrinsic component of presynaptic membrane | 0.058742828 | 0.190743074 | 0.165557239 | CNR1 | 1 |
| Cellular component | GO:0005901 | caveola | 0.060169263 | 0.190743074 | 0.165557239 | FASLG | 1 |
| Cellular component | GO:0045334 | clathrin-coated endocytic vesicle | 0.065854853 | 0.190743074 | 0.165557239 | SH3GL2 | 1 |
| Cellular component | GO:0032592 | integral component of mitochondrial membrane | 0.067271225 | 0.190743074 | 0.165557239 | CNR1 | 1 |
| Cellular component | GO:0098573 | intrinsic component of mitochondrial membrane | 0.068685593 | 0.190743074 | 0.165557239 | CNR1 | 1 |
| Cellular component | GO:0043202 | lysosomal lumen | 0.071508327 | 0.190743074 | 0.165557239 | FASLG | 1 |
| Cellular component | GO:0005604 | basement membrane | 0.072212761 | 0.190743074 | 0.165557239 | ATRN | 1 |
| Cellular component | GO:0032588 | trans-Golgi network membrane | 0.073620134 | 0.190743074 | 0.165557239 | VPS53 | 1 |
| Cellular component | GO:0044853 | plasma membrane raft | 0.082022615 | 0.198113223 | 0.171954228 | FASLG | 1 |
| Cellular component | GO:0043679 | axon terminus | 0.083416094 | 0.198113223 | 0.171954228 | SCGN | 1 |
| Cellular component | GO:0044306 | neuron projection terminus | 0.09449306 | 0.199355273 | 0.173032278 | SCGN | 1 |
| Cellular component | GO:0030665 | clathrin-coated vesicle membrane | 0.095181205 | 0.199355273 | 0.173032278 | SH3GL2 | 1 |
| Cellular component | GO:0005793 | endoplasmic reticulum-Golgi intermediate compartment | 0.095868861 | 0.199355273 | 0.173032278 | ROBO1 | 1 |
| Cellular component | GO:0042383 | sarcolemma | 0.097928906 | 0.199355273 | 0.173032278 | SGCG | 1 |
| Cellular component | GO:0072562 | blood microparticle | 0.104082786 | 0.201638178 | 0.175013747 | ITIH2 | 1 |
| Cellular component | GO:0042734 | presynaptic membrane | 0.106125357 | 0.201638178 | 0.175013747 | CNR1 | 1 |
| Cellular component | GO:0099699 | integral component of synaptic membrane | 0.110874465 | 0.203540282 | 0.176664695 | CNR1 | 1 |
| Cellular component | GO:0099240 | intrinsic component of synaptic membrane | 0.118289794 | 0.203540282 | 0.176664695 | CNR1 | 1 |
| Cellular component | GO:0030426 | growth cone | 0.118961045 | 0.203540282 | 0.176664695 | CNR1 | 1 |
| Cellular component | GO:0030427 | site of polarized growth | 0.122978543 | 0.203540282 | 0.176664695 | CNR1 | 1 |
| Cellular component | GO:0005775 | vacuolar lumen | 0.124980875 | 0.203540282 | 0.176664695 | FASLG | 1 |
| Cellular component | GO:0030666 | endocytic vesicle membrane | 0.136905542 | 0.209302182 | 0.181665791 | SH3GL2 | 1 |
| Cellular component | GO:0030662 | coated vesicle membrane | 0.139534788 | 0.209302182 | 0.181665791 | SH3GL2 | 1 |
| Cellular component | GO:0055037 | recycling endosome | 0.139534788 | 0.209302182 | 0.181665791 | VPS53 | 1 |
| Cellular component | GO:0005741 | mitochondrial outer membrane | 0.145423284 | 0.21094033 | 0.183087636 | CNR1 | 1 |
| Cellular component | GO:0030136 | clathrin-coated vesicle | 0.148028302 | 0.21094033 | 0.183087636 | SH3GL2 | 1 |
| Cellular component | GO:0030658 | transport vesicle membrane | 0.155153936 | 0.215701814 | 0.187220411 | SCGN | 1 |
| Cellular component | GO:0031968 | organelle outer membrane | 0.163503419 | 0.218430758 | 0.189589024 | CNR1 | 1 |
| Cellular component | GO:0019867 | outer membrane | 0.164781098 | 0.218430758 | 0.189589024 | CNR1 | 1 |
| Cellular component | GO:0005802 | trans-Golgi network | 0.180599164 | 0.233958007 | 0.203066045 | VPS53 | 1 |
| Cellular component | GO:0030135 | coated vesicle | 0.210189032 | 0.261757541 | 0.227194911 | SH3GL2 | 1 |
| Cellular component | GO:0019898 | extrinsic component of membrane | 0.21381682 | 0.261757541 | 0.227194911 | RGS6 | 1 |
| Cellular component | GO:0060205 | cytoplasmic vesicle lumen | 0.219229351 | 0.261757541 | 0.227194911 | FASLG | 1 |
| Cellular component | GO:0031983 | vesicle lumen | 0.220427403 | 0.261757541 | 0.227194911 | FASLG | 1 |
| Cellular component | GO:0030139 | endocytic vesicle | 0.229950236 | 0.267493131 | 0.23217317 | SH3GL2 | 1 |
| Cellular component | GO:0097060 | synaptic membrane | 0.250404201 | 0.282039785 | 0.244799075 | CNR1 | 1 |
| Cellular component | GO:0098791 | Golgi apparatus subcompartment | 0.255581212 | 0.282039785 | 0.244799075 | VPS53 | 1 |
| Cellular component | GO:0031301 | integral component of organelle membrane | 0.257299453 | 0.282039785 | 0.244799075 | CNR1 | 1 |
| Cellular component | GO:0005769 | early endosome | 0.270912726 | 0.285992202 | 0.248229612 | SH3GL2 | 1 |
| Cellular component | GO:0031300 | intrinsic component of organelle membrane | 0.274279465 | 0.285992202 | 0.248229612 | CNR1 | 1 |
| Cellular component | GO:0030133 | transport vesicle | 0.275957388 | 0.285992202 | 0.248229612 | SCGN | 1 |
| Cellular component | GO:0009897 | external side of plasma membrane | 0.297443464 | 0.302754955 | 0.262779 | FASLG | 1 |
| Cellular component | GO:0005759 | mitochondrial matrix | 0.308764921 | 0.308764921 | 0.267995407 | ABHD10 | 1 |
| Molecular function | GO:0052689 | carboxylic ester hydrolase activity | 0.006611352 | 0.110048636 | 0.081769884 | ABHD10/PLA2G2F | 2 |
| Molecular function | GO:0070567 | cytidylyltransferase activity | 0.008110256 | 0.110048636 | 0.081769884 | CMAS | 1 |
| Molecular function | GO:0008474 | palmitoyl-(protein) hydrolase activity | 0.011337135 | 0.110048636 | 0.081769884 | ABHD10 | 1 |
| Molecular function | GO:0098599 | palmitoyl hydrolase activity | 0.011337135 | 0.110048636 | 0.081769884 | ABHD10 | 1 |
| Molecular function | GO:0030275 | LRR domain binding | 0.012946898 | 0.110048636 | 0.081769884 | ROBO1 | 1 |
| Molecular function | GO:0047498 | calcium-dependent phospholipase A2 activity | 0.012946898 | 0.110048636 | 0.081769884 | PLA2G2F | 1 |
| Molecular function | GO:0005123 | death receptor binding | 0.016960609 | 0.123429681 | 0.091712456 | FASLG | 1 |
| Molecular function | GO:0005540 | hyaluronic acid binding | 0.019361519 | 0.123429681 | 0.091712456 | ITIH2 | 1 |
| Molecular function | GO:0051787 | misfolded protein binding | 0.023350876 | 0.127206103 | 0.094518467 | DNAJB9 | 1 |
| Molecular function | GO:0005164 | tumor necrosis factor receptor binding | 0.024942373 | 0.127206103 | 0.094518467 | FASLG | 1 |
| Molecular function | GO:0004623 | phospholipase A2 activity | 0.028910533 | 0.133134903 | 0.098923767 | PLA2G2F | 1 |
| Molecular function | GO:0005245 | voltage-gated calcium channel activity | 0.035228253 | 0.133134903 | 0.098923767 | CNR1 | 1 |
| Molecular function | GO:0016790 | thiolester hydrolase activity | 0.035228253 | 0.133134903 | 0.098923767 | ABHD10 | 1 |
| Molecular function | GO:0030544 | Hsp70 protein binding | 0.036801677 | 0.133134903 | 0.098923767 | DNAJB9 | 1 |
| Molecular function | GO:0032813 | tumor necrosis factor receptor superfamily binding | 0.039157324 | 0.133134903 | 0.098923767 | FASLG | 1 |
| Molecular function | GO:0004553 | hydrolase activity, hydrolyzing O-glycosyl compounds | 0.073853027 | 0.213064146 | 0.158313917 | ABHD10 | 1 |
| Molecular function | GO:0004867 | serine-type endopeptidase inhibitor activity | 0.076879814 | 0.213064146 | 0.158313917 | ITIH2 | 1 |
| Molecular function | GO:0051087 | chaperone binding | 0.082154485 | 0.213064146 | 0.158313917 | DNAJB9 | 1 |
| Molecular function | GO:0004620 | phospholipase activity | 0.084406436 | 0.213064146 | 0.158313917 | PLA2G2F | 1 |
| Molecular function | GO:0005262 | calcium channel activity | 0.092619545 | 0.213064146 | 0.158313917 | CNR1 | 1 |
| Molecular function | GO:0031072 | heat shock protein binding | 0.09633 | 0.213064146 | 0.158313917 | DNAJB9 | 1 |
| Molecular function | GO:0016779 | nucleotidyltransferase activity | 0.097810213 | 0.213064146 | 0.158313917 | CMAS | 1 |
| Molecular function | GO:0016298 | lipase activity | 0.100763852 | 0.213064146 | 0.158313917 | PLA2G2F | 1 |
| Molecular function | GO:0016798 | hydrolase activity, acting on glycosyl bonds | 0.102237284 | 0.213064146 | 0.158313917 | ABHD10 | 1 |
| Molecular function | GO:0015085 | calcium ion transmembrane transporter activity | 0.104443209 | 0.213064146 | 0.158313917 | CNR1 | 1 |
| Molecular function | GO:0022843 | voltage-gated cation channel activity | 0.11612295 | 0.227779632 | 0.169248024 | CNR1 | 1 |
| Molecular function | GO:0004866 | endopeptidase inhibitor activity | 0.136930908 | 0.241791543 | 0.179659351 | ITIH2 | 1 |
| Molecular function | GO:0008022 | protein C-terminus binding | 0.137640202 | 0.241791543 | 0.179659351 | FOXN3 | 1 |
| Molecular function | GO:0030414 | peptidase inhibitor activity | 0.141884555 | 0.241791543 | 0.179659351 | ITIH2 | 1 |
| Molecular function | GO:0061135 | endopeptidase regulator activity | 0.146811655 | 0.241791543 | 0.179659351 | ITIH2 | 1 |
| Molecular function | GO:0005244 | voltage-gated ion channel activity | 0.151712341 | 0.241791543 | 0.179659351 | CNR1 | 1 |
| Molecular function | GO:0022832 | voltage-gated channel activity | 0.151712341 | 0.241791543 | 0.179659351 | CNR1 | 1 |
| Molecular function | GO:0061134 | peptidase regulator activity | 0.173101241 | 0.25719089 | 0.19110159 | ITIH2 | 1 |
| Molecular function | GO:0005125 | cytokine activity | 0.176503552 | 0.25719089 | 0.19110159 | FASLG | 1 |
| Molecular function | GO:0005539 | glycosaminoglycan binding | 0.176503552 | 0.25719089 | 0.19110159 | ITIH2 | 1 |
| Molecular function | GO:0005126 | cytokine receptor binding | 0.200617218 | 0.271021177 | 0.201377965 | FASLG | 1 |
| Molecular function | GO:0005096 | GTPase activator activity | 0.201937348 | 0.271021177 | 0.201377965 | RGS6 | 1 |
| Molecular function | GO:0030246 | carbohydrate binding | 0.201937348 | 0.271021177 | 0.201377965 | ATRN | 1 |
| Molecular function | GO:0003924 | GTPase activity | 0.238085331 | 0.307872078 | 0.228759438 | RGS6 | 1 |
| Molecular function | GO:0022836 | gated channel activity | 0.244377258 | 0.307872078 | 0.228759438 | CNR1 | 1 |
| Molecular function | GO:0005261 | cation channel activity | 0.247505004 | 0.307872078 | 0.228759438 | CNR1 | 1 |
| Molecular function | GO:0004857 | enzyme inhibitor activity | 0.277523966 | 0.336993388 | 0.250397564 | ITIH2 | 1 |
| Molecular function | GO:0046873 | metal ion transmembrane transporter activity | 0.301779059 | 0.337017875 | 0.250415759 | CNR1 | 1 |
| Molecular function | GO:0005216 | ion channel activity | 0.307578524 | 0.337017875 | 0.250415759 | CNR1 | 1 |
| Molecular function | GO:0005543 | phospholipid binding | 0.323578744 | 0.337017875 | 0.250415759 | PLA2G2F | 1 |
| Molecular function | GO:0030695 | GTPase regulator activity | 0.331448813 | 0.337017875 | 0.250415759 | RGS6 | 1 |
| Molecular function | GO:0060589 | nucleoside-triphosphatase regulator activity | 0.331448813 | 0.337017875 | 0.250415759 | RGS6 | 1 |
| Molecular function | GO:0048018 | receptor ligand activity | 0.333124099 | 0.337017875 | 0.250415759 | FASLG | 1 |
| Molecular function | GO:0015267 | channel activity | 0.334795467 | 0.337017875 | 0.250415759 | CNR1 | 1 |
| Molecular function | GO:0022803 | passive transmembrane transporter activity | 0.33535172 | 0.337017875 | 0.250415759 | CNR1 | 1 |
| Molecular function | GO:0030546 | signaling receptor activator activity | 0.337017875 | 0.337017875 | 0.250415759 | FASLG | 1 |

**
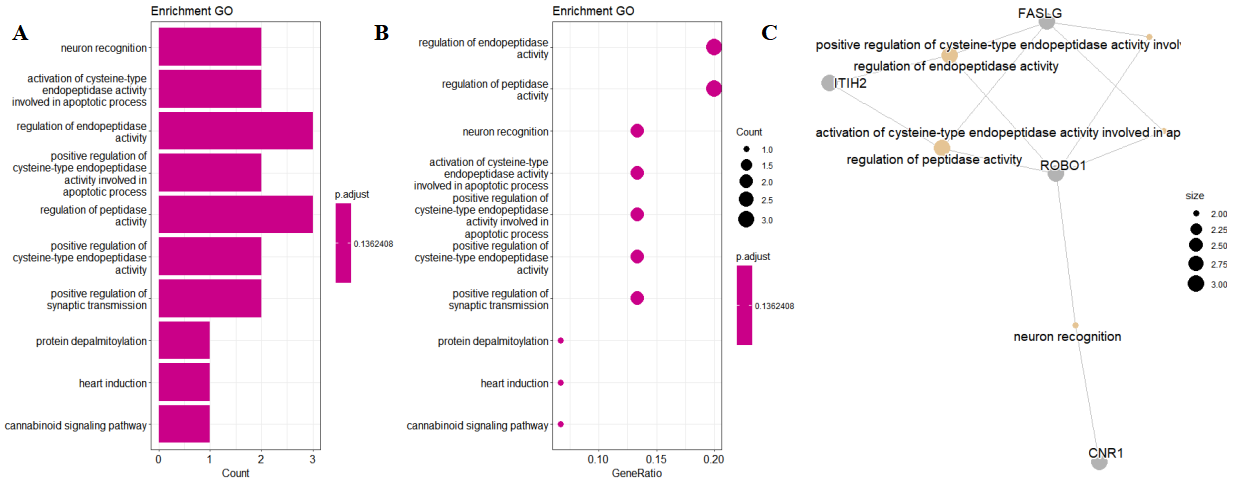
Supplementary Figure.7** Functional enrichment analysis of overlapping genes based on DEGs and GWAS. **(A)** Barplot of the top 10 changes of GO analysis. **(B)** Dotplot of the top 10 changes of GO analysis. **(C)** Netplot of the top 5 changes of GO analysis.

**
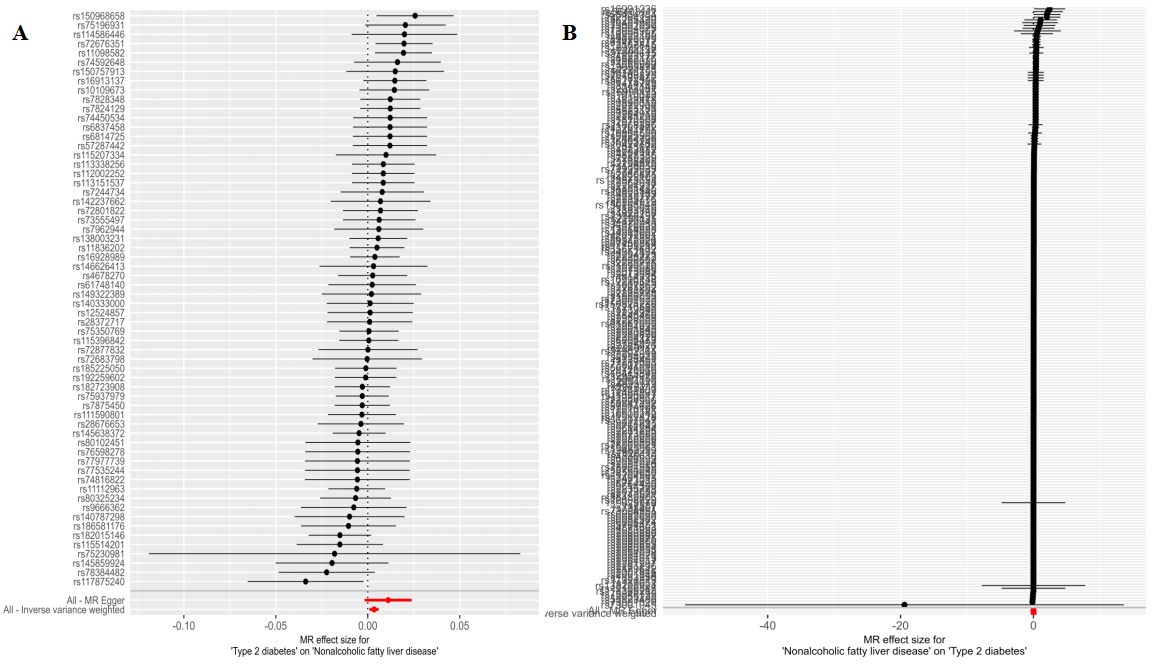
**

**Supplementary Figure.8** Forest plot of MR analysis. **(A)** Forest plot when T2D is exposure and NAFLD is outcome. **(B)** Forest plot when NAFLD is exposure and T2D is outcome.

**
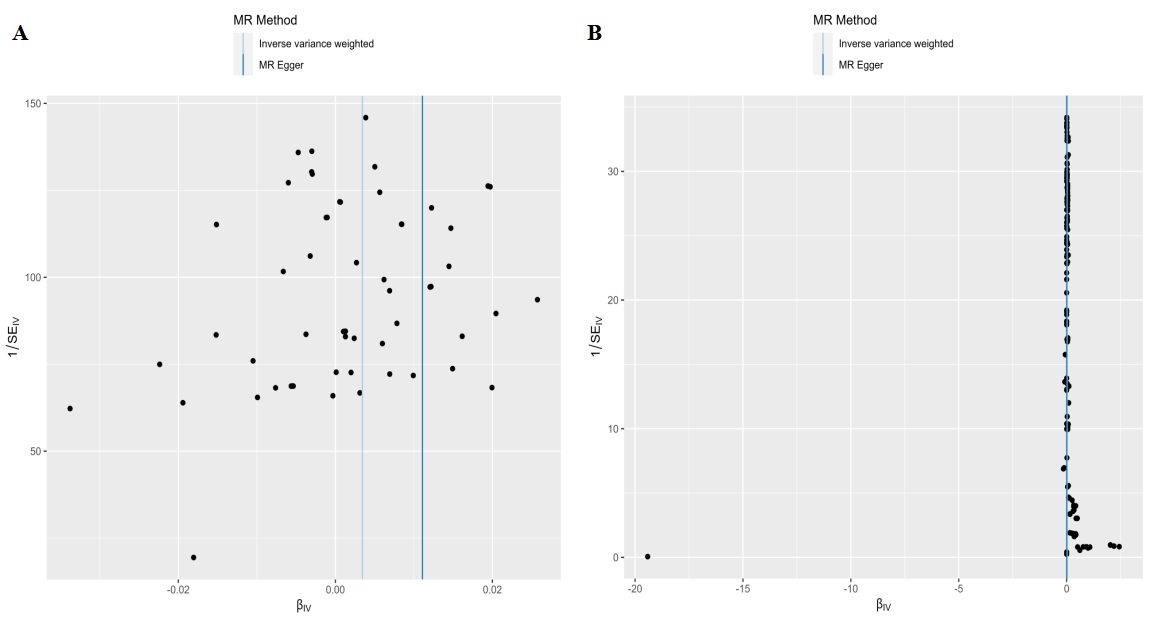
Supplementary Figure.9** Funnel plots of MR analysis. Except for some points that deviate significantly from the population, the funnel chart is relatively symmetrical overall. **(A)** Funnel plot when T2D is exposure and NAFLD is outcome. **(B)** Funnel plot when NAFLD is exposure and T2D is outcome.

**Supplementary Table 8** Summary of the results of the leave-one-out analysis

| exposure | outcome |  | SNP | b | se | p |  | SNP | b | se | p |
| --- | --- | --- | --- | --- | --- | --- | --- | --- | --- | --- | --- |
| T2D | NAFLD | 1 | rs10109673 | 0.003232577 | 0.001306254 | 0.013335131 | 33 | rs28676653 | 0.003517909 | 0.001302209 | 0.006902875 |
|  |  | 2 | rs11098582 | 0.002993706 | 0.001312201 | 0.02252249 | 34 | rs4678270 | 0.003446697 | 0.001306501 | 0.008336888 |
|  |  | 3 | rs11112963 | 0.003695572 | 0.001312475 | 0.004866691 | 35 | rs57287442 | 0.003294242 | 0.001304934 | 0.011587901 |
|  |  | 4 | rs111590801 | 0.003560532 | 0.001306948 | 0.006443598 | 36 | rs61748140 | 0.003444781 | 0.001301995 | 0.008150513 |
|  |  | 5 | rs112002252 | 0.003319208 | 0.001309218 | 0.011236372 | 37 | rs6814725 | 0.003293300 | 0.001304941 | 0.011612172 |
|  |  | 6 | rs113151537 | 0.003319510 | 0.001309215 | 0.011228796 | 38 | rs6837458 | 0.003293249 | 0.001304941 | 0.011613486 |
|  |  | 7 | rs113338256 | 0.003318304 | 0.001309210 | 0.011258058 | 39 | rs7244734 | 0.003376628 | 0.001302799 | 0.009546834 |
|  |  | 8 | rs114586446 | 0.003302868 | 0.001299645 | 0.011042144 | 40 | rs72676351 | 0.002987431 | 0.001312141 | 0.022800532 |
|  |  | 9 | rs115207334 | 0.003376403 | 0.001300179 | 0.009407597 | 41 | rs72683798 | 0.003460390 | 0.001299299 | 0.007738424 |
|  |  | 10 | rs115396842 | 0.003506247 | 0.001310930 | 0.007481277 | 42 | rs72801822 | 0.003378228 | 0.001304700 | 0.009617813 |
|  |  | 11 | rs115514201 | 0.003652619 | 0.001302177 | 0.005031499 | 43 | rs72877832 | 0.003462592 | 0.001300330 | 0.007748043 |
|  |  | 12 | rs117875240 | 0.003676087 | 0.001298782 | 0.004648819 | 44 | rs73555497 | 0.003386415 | 0.001305397 | 0.009482116 |
|  |  | 13 | rs11836202 | 0.003385216 | 0.001313816 | 0.009976923 | 45 | rs74450534 | 0.003291736 | 0.001304954 | 0.011652693 |
|  |  | 14 | rs12524857 | 0.003458891 | 0.001302371 | 0.007911062 | 46 | rs74592648 | 0.003284226 | 0.001302103 | 0.011660764 |
|  |  | 15 | rs138003231 | 0.003373701 | 0.001311693 | 0.010110716 | 47 | rs74816822 | 0.003505132 | 0.001299710 | 0.006999708 |
|  |  | 16 | rs140333000 | 0.003457915 | 0.001302085 | 0.007914977 | 48 | rs75196931 | 0.003200813 | 0.001303354 | 0.014056056 |
|  |  | 17 | rs140787298 | 0.003529394 | 0.001299231 | 0.006597117 | 49 | rs75230981 | 0.003446414 | 0.001294968 | 0.007781885 |
|  |  | 18 | rs142237662 | 0.003402124 | 0.001300244 | 0.00888294 | 50 | rs75350769 | 0.003503711 | 0.001310910 | 0.007523616 |
|  |  | 19 | rs145638372 | 0.003693692 | 0.001315069 | 0.0049735 | 51 | rs75937979 | 0.003639378 | 0.001315176 | 0.005653699 |
|  |  | 20 | rs145859924 | 0.003590359 | 0.001299013 | 0.005711272 | 52 | rs76598278 | 0.003503557 | 0.001299715 | 0.007025474 |
|  |  | 21 | rs146626413 | 0.003435182 | 0.001299419 | 0.008202242 | 53 | rs77535244 | 0.003505047 | 0.001299709 | 0.007001081 |
|  |  | 22 | rs149322389 | 0.003445681 | 0.001300317 | 0.008052117 | 54 | rs77977739 | 0.003505026 | 0.001299709 | 0.007001414 |
|  |  | 23 | rs150757913 | 0.003327221 | 0.001300494 | 0.010514635 | 55 | rs7824129 | 0.003215030 | 0.001310458 | 0.014152748 |
|  |  | 24 | rs150968658 | 0.003100832 | 0.001304160 | 0.017423485 | 56 | rs7828348 | 0.003214958 | 0.001310458 | 0.014154923 |
|  |  | 25 | rs16913137 | 0.003181319 | 0.001308922 | 0.015078556 | 57 | rs78384482 | 0.003678193 | 0.001300698 | 0.004686007 |
|  |  | 26 | rs16928989 | 0.003416819 | 0.001318288 | 0.009545839 | 58 | rs7875450 | 0.003621971 | 0.001313381 | 0.005820139 |
|  |  | 27 | rs182015146 | 0.003855158 | 0.001309190 | 0.003232783 | 59 | rs7962944 | 0.003404480 | 0.001301724 | 0.008913302 |
|  |  | 28 | rs182723908 | 0.003617671 | 0.001313198 | 0.005871802 | 60 | rs80102451 | 0.003502947 | 0.001299711 | 0.007035177 |
|  |  | 29 | rs185225050 | 0.003538259 | 0.001309723 | 0.006902067 | 61 | rs80325234 | 0.003610331 | 0.001305918 | 0.005699393 |
|  |  | 30 | rs186581176 | 0.003568731 | 0.001300863 | 0.006081465 | 62 | rs9666362 | 0.003519655 | 0.001299635 | 0.006765186 |
|  |  | 31 | rs192259602 | 0.003541304 | 0.001309713 | 0.006853559 | 63 | All | 0.003432841 | 0.001294559 | 0.008007674 |
|  |  | 32 | rs28372717 | 0.003461983 | 0.001302359 | 0.007854971 |  |  |  |  |  |
| NAFLD | T2D | 1 | rs1007863 | 0.024110790 | 0.003021051 | 1.45E-15 | 110 | rs2954025 | 0.024144589 | 0.003019695 | 1.29E-15 |
|  |  | 2 | rs1010022 | 0.024096758 | 0.003017398 | 1.39E-15 | 111 | rs2954026 | 0.024194020 | 0.003029741 | 1.40E-15 |
|  |  | 3 | rs1010023 | 0.024096758 | 0.003017398 | 1.39E-15 | 112 | rs2954028 | 0.024191943 | 0.003029536 | 1.40E-15 |
|  |  | 4 | rs10401969 | 0.024135075 | 0.003017193 | 1.25E-15 | 113 | rs2954033 | 0.024119339 | 0.003029741 | 1.71E-15 |
|  |  | 5 | rs10408875 | 0.024131991 | 0.003017222 | 1.26E-15 | 114 | rs2954038 | 0.024163511 | 0.003018667 | 1.20E-15 |
|  |  | 6 | rs10415849 | 0.024188152 | 0.003030139 | 1.43E-15 | 115 | rs2980853 | 0.024220691 | 0.003022237 | 1.11E-15 |
|  |  | 7 | rs10424702 | 0.024135510 | 0.003017235 | 1.25E-15 | 116 | rs2980854 | 0.024266089 | 0.003025068 | 1.04E-15 |
|  |  | 8 | rs10500212 | 0.024188744 | 0.003026205 | 1.32E-15 | 117 | rs2980855 | 0.024214038 | 0.003021822 | 1.12E-15 |
|  |  | 9 | rs10808546 | 0.023994672 | 0.003026138 | 2.21E-15 | 118 | rs2980858 | 0.024116932 | 0.003029401 | 1.71E-15 |
|  |  | 10 | rs11090617 | 0.024193424 | 0.003029506 | 1.39E-15 | 119 | rs2980860 | 0.024211825 | 0.003021684 | 1.12E-15 |
|  |  | 11 | rs11090620 | 0.024050698 | 0.003027848 | 1.97E-15 | 120 | rs2980867 | 0.024198314 | 0.003030162 | 1.40E-15 |
|  |  | 12 | rs112875651 | 0.023925864 | 0.003028086 | 2.76E-15 | 121 | rs2980868 | 0.024196400 | 0.003024808 | 1.25E-15 |
|  |  | 13 | rs113365218 | 0.024328951 | 0.003028984 | 9.59E-16 | 122 | rs2980869 | 0.024196400 | 0.003024808 | 1.25E-15 |
|  |  | 14 | rs11668386 | 0.024130623 | 0.003017232 | 1.27E-15 | 123 | rs2980871 | 0.024060197 | 0.003021189 | 1.67E-15 |
|  |  | 15 | rs11704562 | 0.024321168 | 0.003032791 | 1.06E-15 | 124 | rs2980875 | 0.024213147 | 0.003021766 | 1.12E-15 |
|  |  | 16 | rs11705218 | 0.024135251 | 0.003017599 | 1.26E-15 | 125 | rs2980876 | 0.024139053 | 0.003018832 | 1.28E-15 |
|  |  | 17 | rs11912828 | 0.024232577 | 0.003031687 | 1.32E-15 | 126 | rs2980880 | 0.024139053 | 0.003018832 | 1.28E-15 |
|  |  | 18 | rs12166587 | 0.024139829 | 0.003017188 | 1.24E-15 | 127 | rs2980882 | 0.024220207 | 0.003022207 | 1.11E-15 |
|  |  | 19 | rs12167845 | 0.024105263 | 0.003017312 | 1.36E-15 | 128 | rs2980886 | 0.024271919 | 0.003025431 | 1.04E-15 |
[truncated: 12,256 more chars]
